# Retinal dysfunction in Huntington’s disease mouse models concurs with local gliosis and microglia activation

**DOI:** 10.1038/s41598-024-54347-8

**Published:** 2024-02-20

**Authors:** Fátima Cano-Cano, Francisco Martín-Loro, Andrea Gallardo-Orihuela, María del Carmen González-Montelongo, Samanta Ortuño-Miquel, Irati Hervás-Corpión, Pedro de la Villa, Lucía Ramón-Marco, Jorge Navarro-Calvo, Laura Gómez-Jaramillo, Ana I. Arroba, Luis M. Valor

**Affiliations:** 1 Instituto de Investigación e Innovación Biomédica de Cádiz (INiBICA), Unidad de Investigación, Hospital Universitario Puerta del Mar, Av. Ana de Viya 21, 11009 Cádiz, Spain; 2 Instituto de Investigación Sanitaria y Biomédica de Alicante (ISABIAL), Unidad de Bioinformática, Hospital General Universitario Dr. Balmis, 03010 Alicante, Spain; 3 Departamento de Biología de Sistemas, Universidad de Alcalá de Henares, 28871 Alcalá de Henares, Spain; 4 Instituto Ramón y Cajal de Investigación Sanitaria (IRYCIS), 28034 Madrid, Spain; 5 Laboratorio de Investigación, Diagnostics Building, Instituto de Investigación Sanitaria y Biomédica de Alicante (ISABIAL), Hospital General Universitario Dr. Balmis, Av. Pintor Baeza 12, 03010 Alicante, Spain; 6 Instituto de Investigación, Desarrollo e Innovación en Biotecnología Sanitaria de Elche (IDiBE), 03202 Elche, Spain; 7 Present Address: Programa de Tumores Sólidos, Centro de Investigación Médica Aplicada (CIMA), Departamento de Pediatría, Clínica Universidad de Navarra, Instituto de Investigación Sanitaria de Navarra (IdiSNA), 31008 Pamplona, Spain

**Keywords:** Neuroscience, Diseases of the nervous system, Huntington's disease

## Abstract

Huntington’s disease (HD) is caused by an aberrant expansion of CAG repeats in the *HTT* gene that mainly affects basal ganglia. Although striatal dysfunction has been widely studied in HD mouse models, other brain areas can also be relevant to the pathology. In this sense, we have special interest on the retina as this is the most exposed part of the central nervous system that enable health monitoring of patients using noninvasive techniques. To establish the retina as an appropriate tissue for HD studies, we need to correlate the retinal alterations with those in the inner brain, i.e., striatum. We confirmed the malfunction of the transgenic R6/1 retinas, which underwent a rearrangement of their transcriptome as extensive as in the striatum. Although tissue-enriched genes were downregulated in both areas, a neuroinflammation signature was only clearly induced in the R6/1 retina in which the observed glial activation was reminiscent of the situation in HD patient’s brains. The retinal neuroinflammation was confirmed in the slow progressive knock-in zQ175 strain. Overall, these results demonstrated the suitability of the mouse retina as a research model for HD and its associated glial activation.

## Introduction

Huntington’s disease (HD) (OMIM #143100) is a fatal rare disorder without a cure, and its estimated prevalence is 1–15 per 100,000 people worldwide^1^. HD is caused by an aberrant expansion (fully penetrant with > 39) of CAG repeats at exon 1 of the *HTT* locus, which triggers a progressive symptomatology, usually starting in mid-adulthood (35–45 years old) that includes cognitive and motor impairments, psychiatric disorders and other symptoms (weight loss, sleep disturbance, etc.) until final death^2^. Although the most prominent signs are derived from malfunctioning and degeneration of the basal ganglia and the corticostriatal circuitry^3^, the involvement of other brain areas and peripheral tissues/cells plays a substantial role in the decline of quality of life, extending the repertoire of HD symptoms^4–8^. However, our current toolbox of biomarkers with potential application in clinics is still limited and cannot explain the large variability in the pleiotropic manifestation of symptoms^9–11^. This fact justifies the search for novel biomarkers with prognostic value in HD to enable the evaluation of therapeutic responses and to facilitate decision-making during clinical management.

The retina is a highly organized tissue of the central nervous system (CNS) characterized by a high cellular diversity arranged in discrete layers. This organization determines the functionality of the retina, in which photoreceptor neurons (rods and cones) transduce light stimuli into the complex network of interneurons (amacrine, bipolar and horizontal cells) to finally converge into ganglion cells, which axons form the optic nerve^12^; the retina also contains a specialized immune system with resident microglia and macroglia (astrocytes, Müller glia), which together with other nonneural type cells provide homeostatic and regulatory support to this network^13^. The retina can reproduce the disruption of key mechanisms that compromise cell function and viability of several neurodegenerative disorders, therefore the accessibility of this tissue to noninvasive techniques offers unique opportunities to infer the health status of inner regions of the CNS. In patients, the suitability of retinal imaging in diagnosis is being investigated in Alzheimer's disease, Parkinson's disease, Lewy body dementia, frontotemporal dementia and multiple sclerosis, with promising prospects^14,15^. HD is not an exception; there are selective abnormalities (e.g., thickness reduction of the temporal retinal nerve fibre layer (RNFL), colour vision impairment) that can be tentatively correlated with performance on the Unified Huntington’s Disease Rating Scale (UHDRS). These anomalies can be accompanied by additional alterations of the visual pathway, i.e., reduced amplitude of visual evoked potentials and impaired temporal contrast sensitivity (reviewed in Ref.^16^). In the case of HD mouse models, mutant retinas express mHTT, display deeply reduced photoexcitability responses and undergo a cellular remodelling^17–21^ that coincides with early stages of motor impairment: therefore, retinal dysfunction could be correlated with striatal impairment in HD.

However, the molecular mechanisms that compromise the retinal structure and visual function in HD mice and their corresponding correlates with inner brain events have not yet been investigated. This study reports for the first time a comprehensive analysis of the concomitant molecular alterations in the retina and striatum of HD mouse models, the transgenic R6/1 and the knock-in (KI) zQ175 strains, in which glial activation becomes a distinctive feature between both tissues during pathology progression.

## Materials and methods

### Animals

Transgenic R6/1^22^ and KI zQ175^23^, together with control wild-type (wt) animals, were maintained on a pure C57BL/6 J background under a 12-h light/dark cycle with food and water provided ad libitum. In all experiments, we pooled females and males in similar proportions (not exceeding 60% towards any sex), except for the RNA-seq analysis in which only males were used to avoid cost increase of the experiment. All animals were euthanized by cervical dislocation by well-trained personnel, followed by immediate tissue dissection for subsequent procedures. Experimental protocols were approved by the Comité de Ética de Experimentación Animal—Órgano Habilitado de la Universidad de Cádiz and authorized by the Dirección General de la Producción Agrícola y Ganadera de la Junta de Andalucía according to European and regional laws. This study was in compliance with the ARRIVE guidelines.

### Nucleic acid extraction and PCR assays

After sacrifice, the striatum and retina were immediately dissected and submerged in RNAlater (Thermo Fisher, Madrid, Spain) until processing. Total RNA and genomic DNA were sequentially extracted using TRIzol (Thermo Fisher, Madrid, Spain), and we next followed the procedures for RT-qPCR and CAG repeat analysis described in Ref.^24^ with minor modifications: qPCR was performed on the Rotor-Gene 6000 Detection System (Corbett, Hilden, Germany) and QuantStudio 12 K Flex (Thermo Fisher, Madrid, Spain), and each independent reaction was normalized to the level of *Tbp*, since its expression in the retinal samples was less variable across time points (coefficient of variation CV = 0.51) than other housekeeping genes (e.g., *Gapdh*, CV = 0.69; *Actb*, CV = 1.00); in addition, the latter genes were found to be significantly altered in the RNA-seq analysis (see Supplementary Material). Fold changes were estimated using the ΔΔC_T_ method. The sequences of all primer pairs are provided in Supplementary Table S1.

### RNA-seq analysis, external datasets and bioinformatics

After the TRIzol procedure, striatal and retinal RNA from the same R6/1 mice was pooled (3–4 samples per genotype) and further processed using the clean-up protocol of the RNeasy Mini Kit (Qiagen, Hilden, Germany), which also included on-column DNase I treatment. A total of three pools per genotype were analyzed. DNA libraries were produced for mRNA using the TruSeq Stranded mRNA kit (Illumina, San Diego, CA, USA) and subsequently sequenced using a NovaSeq apparatus (Illumina, San Diego, CA, USA) at STAB-VIDA facilities in a 150-bp paired-end configuration. The resulting reads (> 40 M/sample) were mapped onto the mouse genome GRCm38/mm10 using “Salmon” software^25^. Genes with < 10 counts in all samples were removed. The normalization of read counts and differential expression analysis was conducted using the “DEseq2” package^26^. Differentially expressed genes (DEGs) were filtered with an FDR threshold (adjusted *p-*value) of 0.05. See Supplementary Table S2 for complete results. To retrieve differentially spliced genes, we combined the results of three programs: vast-tools^27^ (dPSI > 0.95), rMATS^28^ (lncLevelDifference, FDR < 0.05) and SUPPA2^29^ (dPSI, *p*-value < 0.05). See Supplementary Table S3 for complete results. The RNA-seq data can be downloaded from the Gene Expression Omnibus (GEO) database using the accession number GSE216520.

To determine the most affected cell subtypes by the expression of mHTT in the differential expression between R6/1 and wt littermates, we used the cell-specific signatures obtained from mouse retinas in physiological conditions^30^, i.e., the genes that define the different subpopulations (clusters) of cells identified by scRNA-seq (Supplementary Table S4 of the original publication^30^). Amacrine and bipolar cells were defined by several clusters that we pooled for our study. Because rod cells constitute ~ 70% of the total retinal cells, contamination from rod cytoplasmic mRNA was present in nearly all clusters^31^; therefore, we filtered out the rod signature of the cell-specific signatures prior to the analysis of marker expression.

To investigate the presence of glial activation markers in our RNA-seq datasets, we used the most significant DEGs (top250): (i) between control and activated Aldh1h^+^-astrocytes (isolated from 1 to 7 days after different in vivo injuring protocols^32^), as calculated by applying the “affy”^33^ and “limma”^34^ packages, and (ii) among CD11b^+^-CD45^+^ microglial cells isolated from the neurodegenerative model CK-p25 at different time points after induction of the transgene (from immediately to 6 weeks), as presented in Supplementary Table S4 of the original publication^35^. Astrocytic panmarkers and specific markers for neurotoxic (A1) and neuroprotective (A2) astrocytes were obtained from a previous report^36^. Related to autophagy, we also used two classifications of autophagic-related genes as compiled by a recent study^37^: one consisted on “mTOR and upstream pathways”, “autophagy core”, “autophagy regulators”, “mitophagy”, “docking and fusion”, “lysosome” and “lysosome-related”, and another one consisted on “autophagy induction” and “lysosomal biogenesis” genes. Other additional bioinformatic tools included Venny (http://bioinfogp.cnb.csic.es/tools/venny/) for the identification of overlapping genes between multiple lists of genes, DAVID 2021 Update^38^ for overrepresentation analysis of Gene Ontology (GO) terms related to biological processes, Pscan^39^ for overrepresentation analysis of transcription factor binding sites (TFBS) at the promoter regions (-950/ + 50) from the Jaspar 2020_NR database, and the native R environment for statistical analysis (ANOVA and Student’s t-test).

### Western blotting assays

Whole retinas were homogenized in lysis buffer containing 125 mM Tris–HCl pH 6.9, 2% SDS, and 1 mM DTT supplemented with protease inhibitors (cOmplete EDTA-free, Sigma-Aldrich, Darmstadt, Germany). All debris was removed by centrifugation at 14,000 × g for 10 min at 4 °C and the protein concentration was quantified using the Bio-Rad protein assay with BSA as a standard. Equivalent amounts of protein were resolved using denaturing sodium dodecyl sulphate–polyacrylamide gel electrophoresis (SDS-PAGE), followed by transfer to PVDF membranes (Merck Millipore, Cork, Ireland). Membranes were blocked using 5% nonfat dried milk or 3% BSA in 10 mM Tris-HCl pH 7.5, 150 mM NaCl, and 0.1% Tween-20, and incubated overnight with several primary antibodies (1:1000) in fresh blocking solution. After incubation with secondary antibodies (1:5000), immunoreactive bands were visualized using enhanced chemiluminescence reagent (Bio-Rad, Hercules, CA, USA). The fold change relative to the basal condition is shown. Blots were quantified by scanning densitometry. Primary antibody against LC3 (#2775) were purchased from Cell Signaling Technology (Danvers, CA, USA); primary antibody against α-tubulin (T5168) and secondary antibodies (A0545, anti-rabbit; A9044, anti-mouse IgG-peroxidase) were purchased from Sigma-Aldrich (Darmstadt, Germany).

### Immunohistochemistry assays

Eye balls and whole retinal explants were fixed in 4% paraformaldehyde in 0.1 M phosphate buffer, pH 7.4, at 4 °C overnight and subsequently put in a solution of PBS with 25% sucrose for another 24 h and cryopreserved in Tissue-Tek (4583, Sakura Finetek, Barcelona, Spain) at − 80 °C until cryosectioning. For immunofluorescence analysis, cryosections were incubated with a permeabilization solution containing 0.1 M TBS, 2% Triton X-100, blocked for 2 h in TBS containing 3% BSA and 1% Triton X-100, and incubated overnight in a humid chamber at 4 °C with rabbit anti-glial fibrillary acidic protein (GFAP) antibody (1:500, Z0334, DAKO, Agilent, Glostrup, Denmark), rabbit anti-ionized calcium binding adaptor molecule 1 (Iba-1) (1:500, 019-19741, WAKO, FUJIFILM Cellular Dynamics, Madison, WI, USA) or mouse anti-huntingtin EM48 (1:300, Merck Millipore, Burlington, MA, USA) in blocking solution. Next, sections were washed and incubated for 2 h with secondary antibodies conjugated to Alexa-488 (1:2000; Molecular Probes, Thermo Fisher Scientific, Waltham, MA, USA). After washing, both the retinal explants and sections were mounted with medium (Fluoromount G, Southern Biotech, Thermo Fisher Scientific, Waltham, MA, USA) containing 4’-6-diamidino-2-phenylindole (DAPI). Staining was observed with an inverted laser confocal microscope Axio Observer LSM900 (Carl Zeiss Microscopy GmbH, Göttingen, Germany). Cell nuclei labelled with DAPI were counted in the outer nuclear layer (ONL) using ImageJ software on retinal section images at 40 × magnification. In each mouse, we measured a total of six retinal cross-sections made through the optic nerve head, and averaged for each animal. For the EM48 antibody we followed the Diaminobenzidene (DAB) staining produce described for the 3,3′-Diaminobenzidine (DAB) Liquid Substrate System tetrahydrochloride (Sigma-Aldrich, Darmstadt, Germany) following the manufacturer’s instructions.

### Electroretinogram recordings

All electroretinogram (ERG) procedures have been described previously^40^. Dark-adapted mice were anaesthetized with ketamine (95 mg/kg) and xylazine (5 mg/kg) under dim red light and the eyes were instilled as in the SD-OCT procedure. All mice were placed in a Faraday cage. Light intensity was measured in the eye with a photometer (Mavo Monitor USB, Nuremberg, Germany). Flash-induced ERG responses were recorded from the right eye in response to 4 to 64 consecutive light stimuli produced with a Ganzfeld stimulator: from − 4 to 1.5 log Cd × s × m^−2^ for rod-mediated responses, from − 1.5 to 0.5 log Cd × s × m^-2^ for mixed rod- and cone-mediated responses, from − 0.5 to 2 log Cd × s × m^−2^ on a rod saturating background of 30 Cd × s × m^−2^ for cone-mediated responses, with intervals between flashes of 10 s (dim flashes, scotopic condition), up to 60 s (high intensity flashes, scotopic condition) and 1 s (photopic condition). For recording, we used corneal electrodes, a reference electrode (in the mouth) and a ground electrode (in the tail) (Burian-Allen electrode, Hansen Ophthalmic Development Laboratory, Coralville, IA, USA). The recorded signals were amplified and band-filtered between 0.3 and 10.000 Hz with a Grass amplifier (CP511 AC amplifier; Grass Instruments, Quincy, MA, USA), and digitized at 10 kHz with a Power-Lab data acquisition board (AD Instruments, Chalgrove, UK). Wave amplitudes were calculated blinded to the animal genotype.

### Spectral-domain optical coherence tomography (SD-OCT)

R6/1 (25-week-old), zQ175 (12-month-old) and matched-age wt mice were anaesthetized and maintained on a heated pad at 37 °C. The eyes were instilled with a topical drop of 1% tropicamide (Colircusí Tropicamida, Alcon Cusí SA, Barcelona, Spain) for pupil dilation and 2% Methocel (Ciba Vision AG, Hetlingen, Switzerland). OCT images were obtained using a Micron IV rodent imaging system (Phoenix Research Labs, Pleasanton, CA, USA) as described elsewhere^41^. A B-scan including the maximal retinal thickness (in the centre of the retina) was segmented between the inner limiting membrane and the base of the retinal pigment epithelium using the Insight software package (Phoenix Research Labs). The thickness of both eyes (containing average raw data from 624 A-scans from each eye after removing the first and last 200 scans) was averaged per animal for statistical analysis.

## Results

### The retina and striatum of R6/1 mice showed extensive transcriptional dysregulation

**Figure 1:**
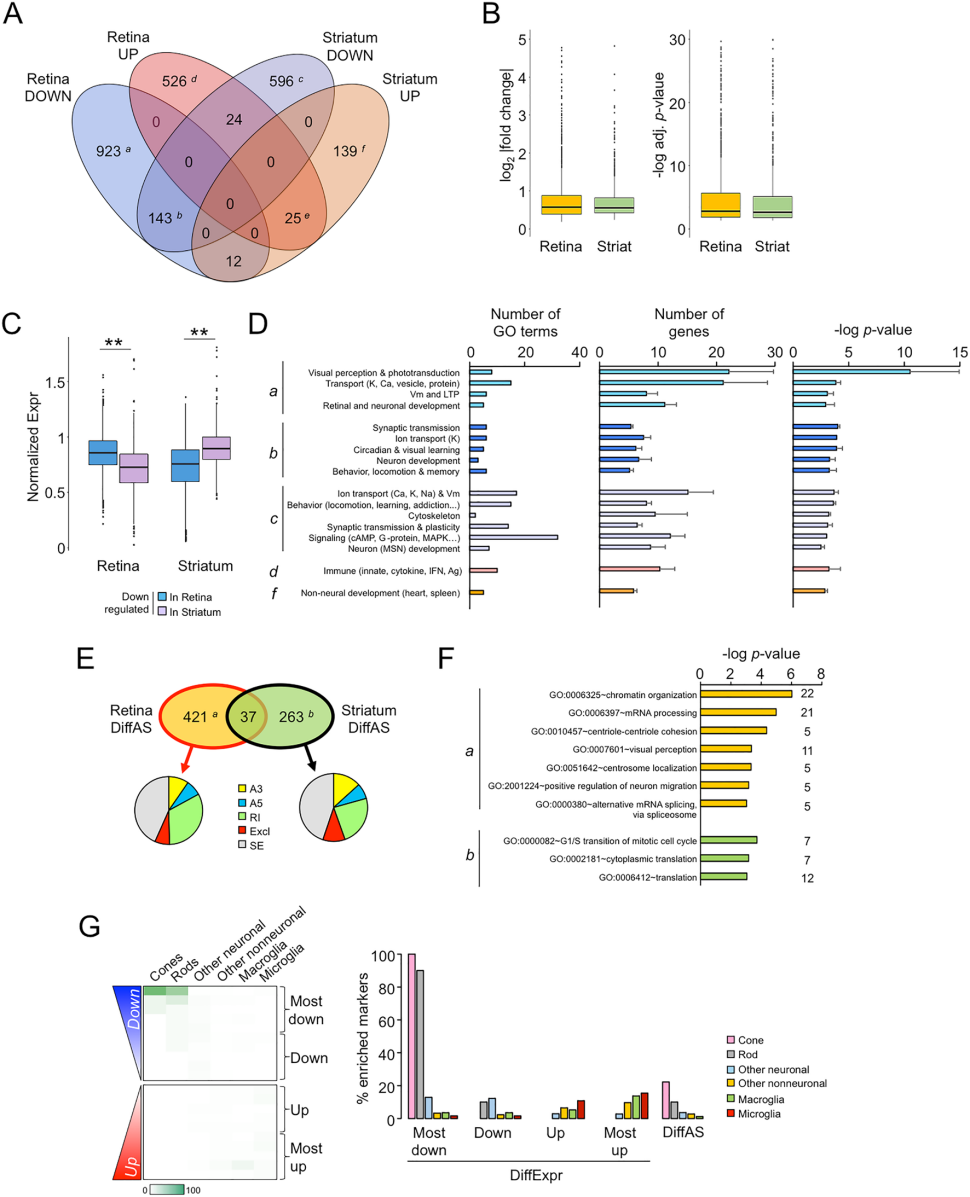
RNA-seq analysis in the retina and striatum of R6/1 mice. (**A**) Venn diagram showing the number of DEGs in pairwise comparisons between wt and R6/1 mice (adj. *p*-value < 0.05, n of pools = 3 per genotype). Downregulated genes were indicated as *a* (exclusive retinal), *b* (common to both tissues) and *c* (exclusive striatal), and upregulated genes as *d* (exclusive retinal), *e* (common) and *f* (exclusive striatal). (**B**) Absolute values of log_2_ fold change (left) and − log adj. *p*-value (right) in the R6/1 retina and striatum: outliers > 5 and > 30 were removed from each panel respectively to enable visualization of medians and quartiles of the values. (**C**) Normalized basal expression of DEGs in wt retinas and striata. **p-value < 0.005, Student’s *t*-test between tissues. (**D**) GO enrichment analysis of DEGs in the retina and the striatum of R6/1 mice (*p*-value < 0.001 (DAVID)); not significant results were retrieved for subset *e*. Letters indicate the subsets of genes defined in (**A**). Data are expressed as mean ± s.e.m. *Vm* membrane potential, *cAMP* cyclic AMP, *MSN* medium spiny neurons, *IFN* interferon, *Ag* antigen. (**E**) Venn diagram showing the genes affected by differential alternative splicing (diffAS) in R6/1 retina and striatum compared to wt littermates. Pie charts indicate the type of aberrant splicing compiled in vast-tools, rMATS and SUPPA2: A5, alternative 5’ splice-site; A3, alternative 3’ splice-site; RI, retained intron; Excl, excluded exon (mutually exclusive exons, alternative first and last exon); SE, skipped exon. (**F**) GO enrichment analysis of genes with diffAS in the retina and the striatum of R6/1 mice (*p*-value < 0.001 (DAVID)). Numbers besides bars indicate the number of genes contained in GO categories. (**G**) Retinal DEGs were ranked according to their significance and direction of change, and divided in bins of 100 genes. On the left, percentage of retinal cell-specific markers counted per bin. For “Other neuronal” we represent the average of counts from amacrine, bipolar and retinal ganglion cells, for “Macroglia” the averaged counts from Müller glia and astrocytes, and for “Other non-neuronal” the averaged counts from pericytes and perivascular fibroblasts. On the right, the same data were represented as grouped percentages in a bar graph, besides the genes affected by aberrant alternative splicing (diffAS).

Based on the retinal impairments reported in HD mouse models^17–21^, we examined the molecular alterations in the R6/1 retina and their relationships with the dysfunction of the most affected brain area in HD, the striatum. Basal ganglia are well documented to manifest the most dramatic transcriptional dysregulation in patients and HD mouse models compared to the rest of the brain^42,43^ but a direct comparison with retinal tissue is still lacking. Once demonstrated that R6/1 retina effectively expressed mHTT (Supplementary Fig. S1A) and showed an altered visual function (Supplementary Fig. S1B), we screened by RNA-seq the whole mRNA expression of the retinas and striata obtained from the same mutant and wt mice in an earlier pathological stage (13–15 weeks-old). The differential expression analysis (adjusted *p*-value < 0.05) revealed extensive dysregulation of the R6/1 retina transcriptome related to wt littermates (1078 downregulated and 575 upregulated genes), resulting in more DEGs compared to the striatum of the same animals (763 downregulated and 176 upregulated genes) although comparable in magnitude and significance (Fig. 1A,B). Using our significance cut-off, most of the DEGs in our RNA-seq analysis were specific to the retina and the striatum: 87.7% and 78.3%, respectively. In fact, downregulation generally affected genes that were highly expressed in the corresponding neural tissue: i.e., downregulated retinal genes were physiologically more highly expressed in the retina than in the striatum, and vice versa (Fig. 1C), in agreement with the strong tissue-enriched component of HD-associated transcriptional dysregulation (Refs.^44,45^ and references therein). This tissue specificity was also confirmed at the functional level (Fig. 1D), since we detected that downregulated genes in the R6/1 retina (subset *a*) were enriched in GO terms genes associated with visual perception, phototransduction and retinal development, whereas downregulated genes in the striatum (subset *c*) were enriched with genes linked to addiction, locomotion, and medium spiny neurons, among the expected pathways affected in HD (e.g., cAMP and G-protein-dependent). Notably, we retrieved circadian functions among the commonly altered genes in both tissues (subset *b*) that might be related to the circadian-like modulation of GABAergic interneurons and dopamine in diverse brain areas, including the retina and striatum^46,47^. This observation should be explored in future studies considering the circadian disruption in HD patients and mice^5,48^. All subsets of downregulated genes were enriched with neuronal functions (e.g., synaptic transmission, ion transport, neuronal development) (Fig. 1D). In contrast, upregulation was linked to distinctive phenomena in each tissue, as evidenced by the retrieval of nonoverlapping associated functions (i.e., immune response in retinal subset *d* and nonneural development in striatal subset *f*, Fig. 1D) and different putative regulatory mechanisms (Supplementary Fig. S2A,B). Since RNA splicing is also documented to be disturbed in HD^49,50^, we also investigated differential spliced genes between genotypes in our RNA-seq datasets. We observed a similar behaviour as in differential expression: high tissue specificity with a prominent retinal deregulation (Fig. 1E). The affected genes were enriched in mRNA processing functions (e.g., splicing in the retina and translation in the striatum) (Fig. 1F).

To explore in further detail the retinal transcriptional profile of the R6/1 strain, we examined the cell types that were the most affected according to our differential gene expression analysis. To this end, we used the markers obtained from a scRNA-seq analysis performed in the mouse retina that were assigned to specific neuronal and nonneuronal cells^30^ (see “Materials and methods” for further details). The presence of these markers among the DEGs inferred the prominent alterations of photoreceptor cells because specific genes of cones (e.g., *Opn1sw*, *Arr3*) and rods (e.g., *Rho*, *Gnat1*) were the most downregulated genes in R6/1 retinas compared to wt littermates, followed by the upregulation of microglia (e.g., *Trf*, *H2-K1*, *Ctss*) and macroglia markers (e.g., *Gfap*) (Fig. 1G), in agreement with the GO enrichment of innate immune response in Fig. 1D. Aberrant splicing was less selective but still showed a trend towards photoreceptor cells (Fig. 1G).

### Progressive molecular alterations in the R6/1 retina and striatum

**Figure 2:**
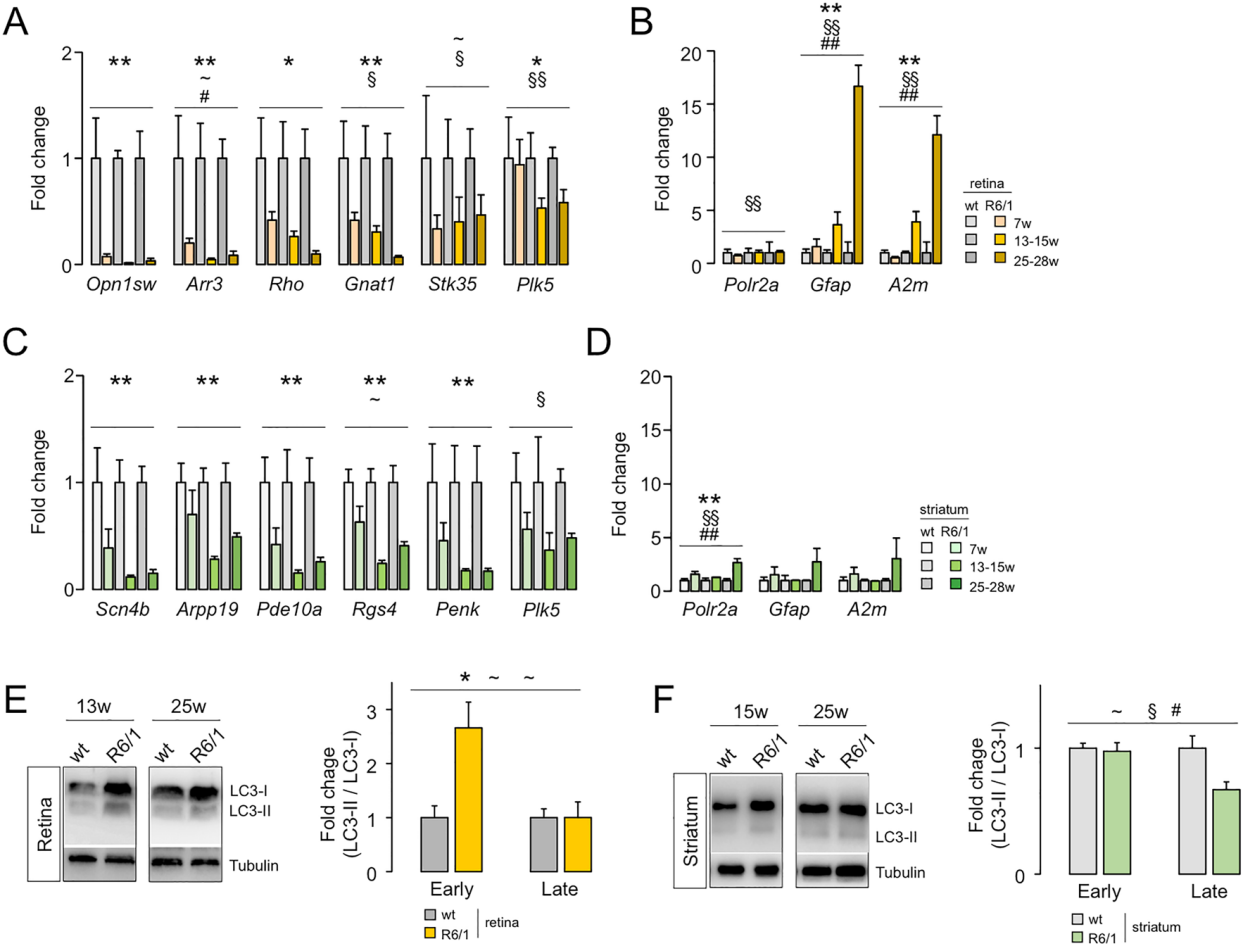
Time-course profiling of molecular alterations in the R6/1 retina and striatum. (**A**,**B**) Time-course analysis of the downregulation (**A**) and upregulation (**B**) of selected genes in the R6/1 retina compared to wild-type littermates. 7 weeks, n = 6 (wt) and n = 8 (R6/1); 13–15 weeks, n = 5 (wt) and n = 7 (R6/1); 25–28 weeks, n = 3 per genotype. (**C**,**D**) The same analysis in the R6/1 and wild-type striata. 7 weeks, n = 7 (wt) and n = 8 (R6/1); 13–15 weeks, n = 7 (wt) and n = 9 (R6/1); 25–28 weeks, n = 8 per genotype. Data are expressed as mean ± s.e.m. *p < 0.05, **p < 0.005, genotype effect; ^§^*p* < 0.05, ^§§^*p* < 0.005, age effect; ^#^*p* < 0.05, ^##^*p* < 0.005, interaction effect; ^~^*p* < 0.1 in any effect, ANOVA test. (**E**,**F**) Protein extracts from the retina (**E**) and striatum (**F**) of R6/1 and wt littermates were analyzed by western blotting assays with antibodies against LC3II/I and α-tubulin as loading control. Retina early, 7/9/15-week-old, n = 4 (wt) and n = 6 (R6/1); Retina late, 21/25-week-old, n = 3 (wt) and n = 4 (R6/1); striatum early, 5/13-week-old, n = 6 (wt) and n = 9 (R6/1); striatum late, 25-week-old, n = 4 (wt) and n = 5 (R6/1). Data are expressed as mean ± s.e.m. *p < 0.05 genotype effect; ^§^*p* < 0.05 age effect; ^#^*p* < 0.05 interaction; ^~^*p* < 0.1 in any effect, ANOVA test in R6/1 and wt littermates. More blots are shown in Supplementary Fig. S3.

To establish the time-frame of molecular impairments in the R6/1 retinas, we further analysed the transcript levels of photoreceptors and gliosis markers together with other down- and upregulated genes at different stages of the pathology: prodromal (7 weeks old), symptomatic (13–15 weeks old) and advanced symptomatic (25–28 weeks old). In general, downregulation preceded upregulation in the R6/1 retina; nonetheless, all examined genes were significantly altered in the intermediate age of sampling (Fig. 2A,B). A similar behaviour was observed in the R6/1 striatum, except for the lack of upregulation of the gliosis-related genes *A2m* and *Gfap* compared to wt striatum (Fig. 2C,D), confirming previous results from our laboratory^24^ at the timings of sampling.

On the basis that autophagy is known to be altered in HD^51^ and is involved in inflammation resolution due to its clearance role^52,53^, we compared the dysfunctions in autophagy processes between the retina and the striatum of R6/1 mice and their wt littermates. To this end, we analysed by western blotting assays the conversion of the cytosolic form of LC3 (LC3-I) into the LC3-phosphatidylethanolamine conjugate (LC3-II), which is recruited to autophagosomal membranes, as a marker of autophagy activation. Based on their similarity, we grouped the results for R6/1 at early (5–15 weeks old) and advance stages (21–25 weeks old). We detected a significant increase in the LC3-II / LC3-I ratio in the retinas from early R6/1 mice compared with age-matched wt mice in response to the mHTT insult, but the LC3-II / LC3-I ratio was later similar in advanced R6/1 mice (Fig. 2E and Supplementary Fig. S3A), indicating that autophagic influx was delayed^54^. The inability to activate the autophagic influx in the mutant striatum was already observed in early stages, leading to the accumulation of LC3-I in advanced stages (Fig. 2F and Supplementary Fig. S3B) in agreement with other reports^55,56^. After inspecting the RNA-seq results, we confirmed the significant deregulation of several components of the autophagic system in nearly all the categories summarized in Ref.^37^ in both the retina and the striatum of 13–15 weeks-old R6/1 mice, although the most notable difference was observed in genes involved in lysosomal biogenesis that were significantly upregulated in the R6/1 retina in comparison with the striatum of the same mice (Supplementary Fig. S4).

Overall, these results indicated that the progression of the neuronal alterations was similar between the retina and striatum of the R6/1 mice whereas neuroinflammation and autophagy was differentially impaired between both tissues.

### R6/1 retinas showed glial activation

Our previous analyses suggested that local inflammatory processes took place in the retina, but not in the striatum, of symptomatic R6/1 mice, in agreement with a TFBS prediction in which DNA motifs for inflammatory transcription factors (i.e., IRFs and STATs)^57^ were highly enriched in the genes that were upregulated in the R6/1 retina (Supplementary Fig. S2C). This retina-accelerated neuroinflammation was not due to either a higher CAG instability of the transgene (Supplementary Fig. S5A) or higher expression of the R6/1 transgene in the mutant retina related to the striatum (Supplementary Fig. S5B), which might exacerbate the progression of the pathology in the former tissue.

**Figure 3:**
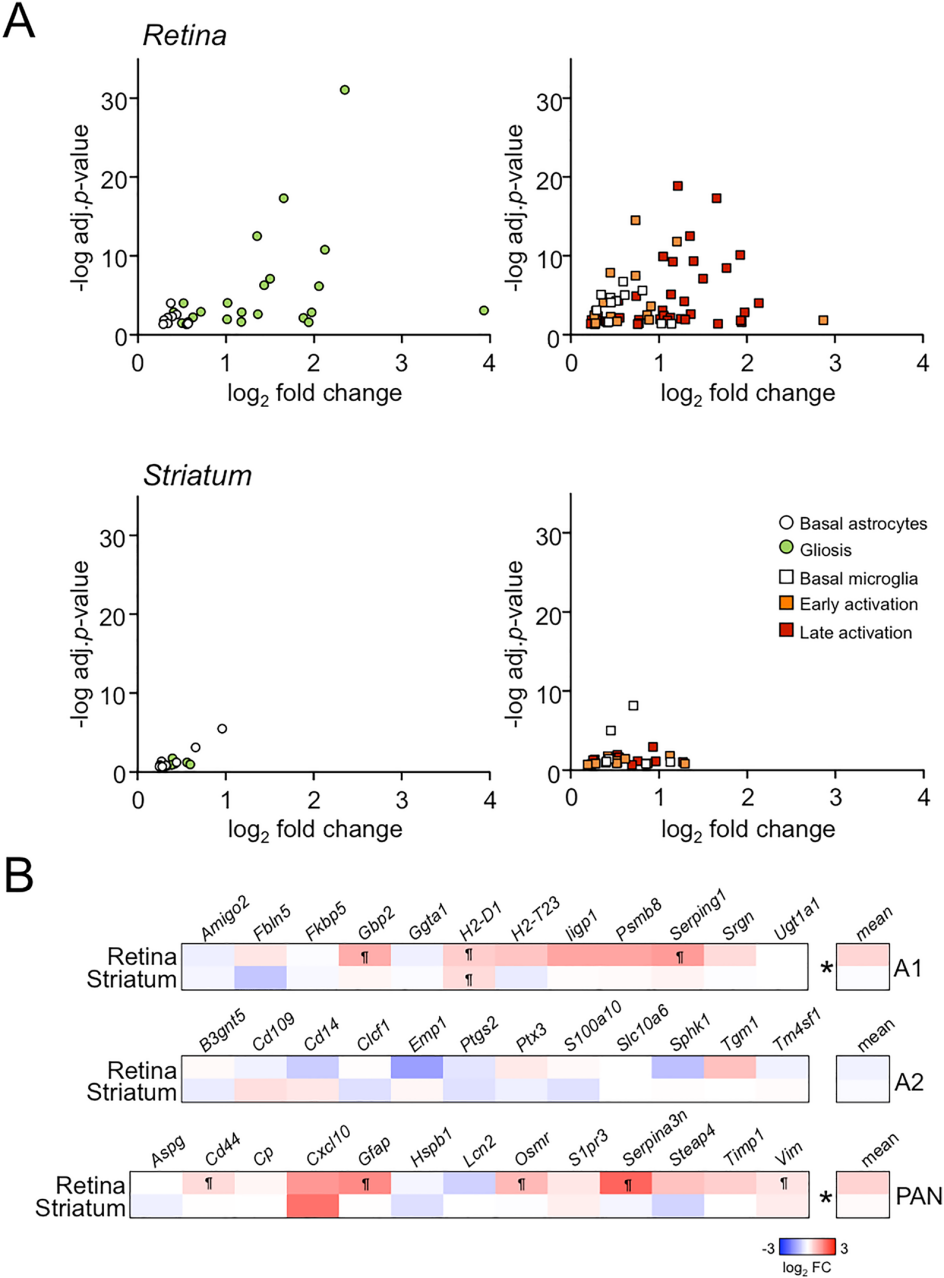
A glial activation signature is only present in the R6/1 retina. (**A**) Plots showing the upregulated genes in 13–15 weeks-old R6/1 retina (top panels) and striatum (bottom panels) according to magnitude (log_2_ fold change) and significance (− log adj. *p*-value) of change in the RNA-seq analysis that are markers for astrocytes (left panels) and microglia (right panels) at different stages of activation (see text for further details). For striatal genes no significance cut-off was applied to permit the analysis of the same number of genes as in retina to facilitate the comparison between both tissues. (**B**) Heatmap plot of the fold changes of DEGs belonging to the A1, A2 and panastrocytic signatures (see text). ¶, adjusted *p*-value < 0.05 from the RNA-seq analysis. *p-value < 0.05, Student’s *t*-test between the fold changes means of both tissues.

Since retinal markers used in Fig. 1G were retrieved in the basal state, this analysis did not differentiate the activation states of resident glial cells. To determine whether the retinal neuroinflammation was associated with glial activation, we used external datasets to obtain the most significant genes (top250) in the pairwise comparison between isolated astrocytes (Aldh1l1^+^) from control and injured mice (combining LPS injection and middle cerebral artery occlusion^32^): whereas the upregulation signature was linked to astrocytic activation and gliosis, downregulated genes indicated overexpression in resting astrocytes; these markers might be shared between retinal astrocytes and Müller glia as they trigger similar molecular changes after injury^58^. In a similar manner, we also obtained the top250 genes from isolated microglia (CD11b^+^-CD45^+^) at different stages of neurodegeneration triggered by p25 induction; thus, we retrieved the signatures for homeostatic/basal, early and late activated microglia under ongoing neurodegenerative processes^35^. We identified examples of all sets of genes related to basal and activated glia to be significantly upregulated in the R6/1 retina compared to wt littermates, suggesting the coexistence of different stages of glial activation (Fig. 3A, top panels) in HD progression. In contrast, the R6/1 striatum did not show any increase in glial markers (Fig. 3A, bottom panels). To gain further insights regarding the type of glial activation, we examined the changes occurring in markers ascribed to two recently described subtypes of reactive astrocytes: neurotoxic A1 and neuroprotective A2^36^. We found in the retina of R6/1 mice the activation of 25% of A1-astrocytic and 38.5% pan-astrocytic markers, indicating that the detected gliosis was associated with an inflammatory deleterious response (Fig. 3B).

Retinal neuroinflammation was apparently restricted to glial cells since additional inflammatory markers were not induced in our RNA-seq data. For example, most adaptive immune markers for T- and B cells were not detected in the retinal transcriptomes or were expressed at low levels, independently of the genotype (Supplementary Fig. S6A). In addition, other genes associated with inflammation were also lowly expressed (such as the classical proinflammatory cytokines *Il1b*, *Il6* and *Tnf*) or did not show differences between genotypes (such as the inducible heme oxygenase by mitochondrial dysfunction *Hmox1*), as confirmed by RT-qPCR assays in independent samples (Supplementary Fig. S6B).

**Figure 4:**
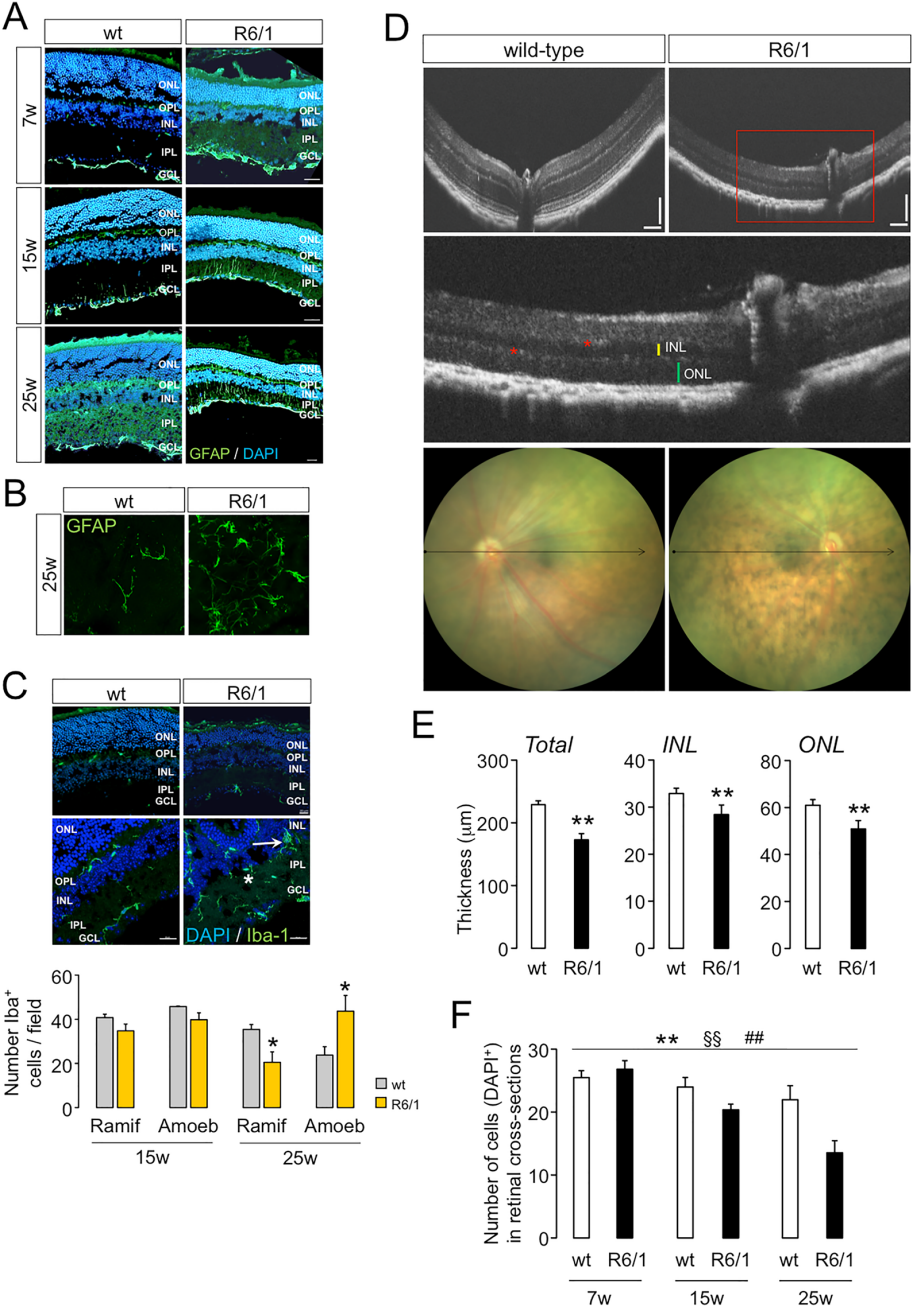
Morphological alterations associated with neuroinflammation in the R6/1 retinas. (**A**) Representative immunofluorescence stainings across the indicated time points of the gliosis marker GFAP in R6/1 and wt littermates. (**B**) Representative whole mounts of R6/1 and wt retinas with GFAP staining. (**C**) Upper panels, representative immunofluorescence staining of the microglia marker Iba-1 for R6/1 and wt littermates. Lower panel, quantification of the Iba-1^+^ cells distinguishing their morphology in ramified and amoeboid in R6/1 retinas compared to wt littermates. N = 2–4 per genotype, age and model; n = 3–5 slices per animal, n = 3 fields per slice; field area = 84,100 μm^2^. Data are expressed as mean ± SD. *p-value < 0.05, Student’s *t*-test between Iba-1^+^ cell subtypes. Green, glial marker; blue, DAPI staining. Scale = 20 μm. *ONL* outer nuclear layer, *OPL* outer plexiform layer, *INL* inner nuclear layer, *IPL* inner plexiform layer, *GCL* ganglion cell layer, *Ramif* ramified, *Amoeb* amoeboid. (**D**) Representative SD-OCT images from the fundus of a R6/1 mouse and wt littermates. Red inset, magnified image indicating hyperreactive spots (*) and thicknesses of INL and ONL (yellow and green lines, respectively). Arrows denote the B-scanned transects for thickness calculations. Scale bars = 100 μm. (**E**) Quantifications of the retinal thickness in 25-week-old R6/1 (n = 5) and matched-age wt (n = 5). Data are shown as mean ± SD. *p < 0.05; **p < 0.005, Student’s *t*-test between genotypes. (**F**) Quantification of DAPI-stained cells in the retinal ONL of mutant mice compared to wt across different time points, N = 3–4 for each genotype, n = 2–5 slices per animal. Data are shown as mean ± SD. **p < 0.005 genotype effect; ^§§^*p* < 0.005 age effect; ^##^*p* < 0.005 interaction effect from ANOVA test in R6/1 and wt littermates.

Because our gene expression analyses were performed using bulk homogenates, we investigated the expression of GFAP by immunofluorescence to obtain spatial sensitivity of Müller cells and astrocytes activation (Fig. 4A,B). Reactive GFAP was detected in retinal sections from R6/1 mice beginning at seven weeks of age with increased labelling in later time points, first in the feet of the ganglion cell layer (GCL), and later extended into the inner nuclear (INL) and inner plexiform layers (IPL) as glial reactivity increased in advance stages (Fig. 4A). We also analysed the microglial activation, as a potential contributor in retinal neuroinflammation detected in R6/1 mice, by determining the time-course staining pattern of the specific reactive microglial marker Iba-1. Immunopositive Iba-1 cells were detected mainly in both plexiform retinal layers of the HD mouse model (Fig. 4C). We did not observed a significant increase in the total number of microglial cells during pathology progression but, interestingly, there was a prominent shift towards an amoeboid-migrating phenotype detriment to ramified-resting morphology in the retinas of 25-week-old R6/1 mice, indicating ongoing progression in microglial activation (Fig. 4C) associated to neuroinflammatory responses.

These results, together with those retrieved from the gene expression analysis (Fig. 2,3), determined that older R6/1 mice showed an increased retinal neuroinflammation. This is in agreement with the presence of aberrant fundus images due to the presence of bright white or hyperreflective spots, as already reported^20^, which were indicative of inflammatory processes^59–61^ (Fig. 4D). This finding was accompanied by a reduction in the total retinal thickness in R6/1 mice (Fig. 4E), and a decrease in the number of nuclei compared to matched-age controls (Fig. 4F). In addition, the localization of activated glial cells identified the OPL as the most damaged layer, potentially explaining the loss of synaptic connection between the ONL and the INL, as determined by ERG recordings (Supplementary Fig. S1B).

### The zQ175 strain also shows retinal neuroinflammation

**Figure 5:**
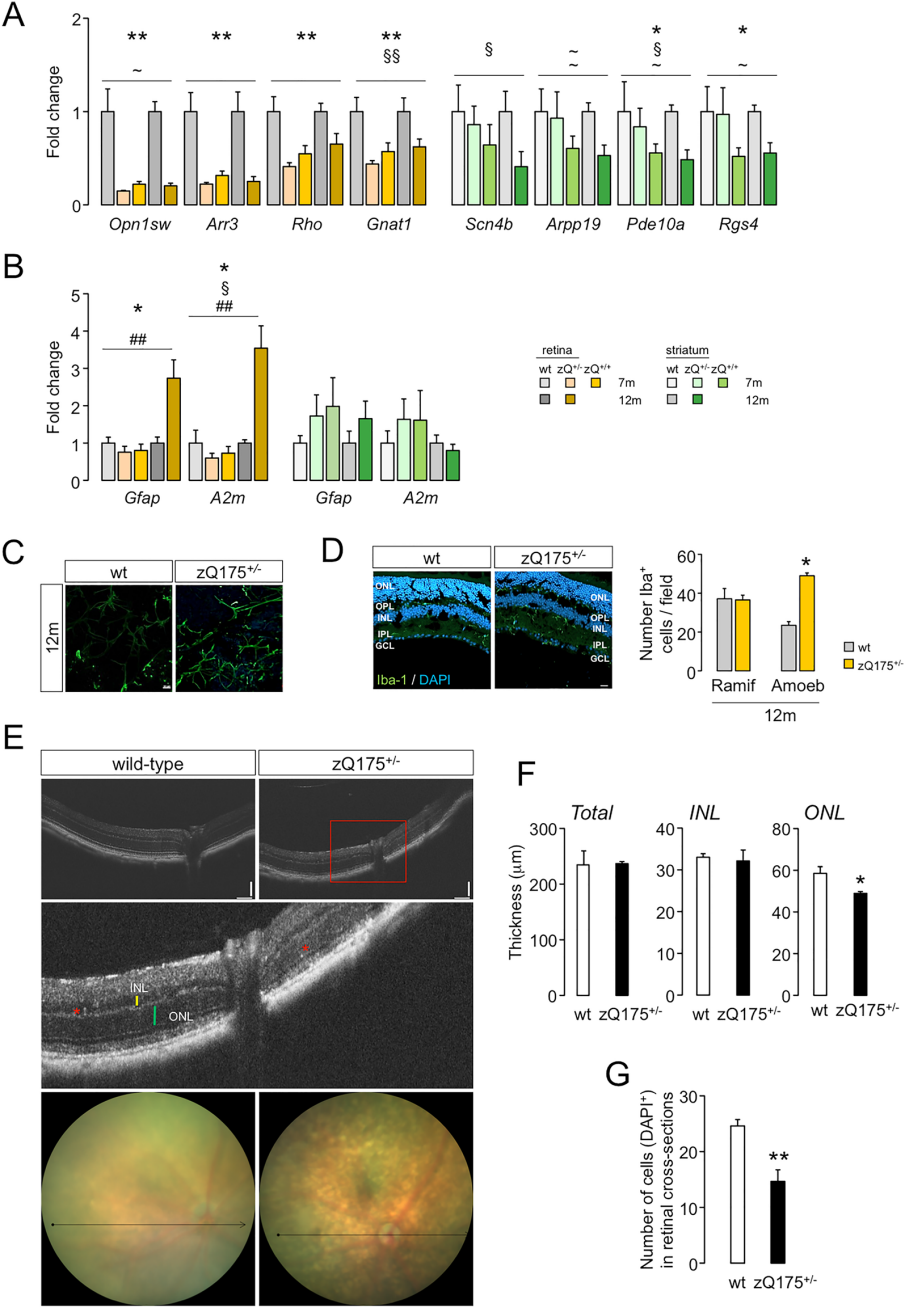
Retinal neuroinflammation is also detected in the zQ175 retinas. (**A**,**B**) The same analysis as in Fig. 2A and B for zQ175 and wt littermates. At the early time point (7 months) we included homozygous mice for the CAG expansion to check whether there was a possible exacerbation of the transcriptional dysregulation that was only observed for downregulated striatal genes. 7 months in each tissue, n = 5 (wt), n = 4 (zQ175^+/−^) and n = 4 (zQ175^+/+^); 12 months in striatum, n = 6 per genotype; in retina, n = 5 (wt) and n = 7 (zQ175^+/−^). Data are expressed as mean ± s.e.m. *p < 0.05, **p < 0.005, genotype effect; ^§^*p* < 0.05, ^§§^*p* < 0.005, age effect; ^##^*p* < 0.005, interaction effect; ~ , *p* < 0.1 in any effect, ANOVA test. (**C**) Representative whole mounts of zQ175^+/−^ and wt retinas with GFAP staining. (**D**) The same analysis as in Fig. 4C for zQ175 and wt littermates. N = 2 per genotype, age and model; n = 3–5 slices per animal, n = 3 fields per slice; field area = 84,100 μm^2^. Data are expressed as mean ± SD.*, *p*-value < 0.05, Student’s *t*-test between Iba-1^+^ cell subtypes. Green, microglial marker; blue, DAPI staining. Scale = 20 μm. *ONL* outer nuclear layer, *OPL* outer plexiform layer, *INL* inner nuclear layer, *IPL* inner plexiform layer; *GCL* ganglion cell layer, *Ramif* ramified, *Amoeb* amoeboid. (**E**) Representative SD-OCT images from the fundus of a zQ175 mouse and wt littermate. Red inset, magnified image indicating hyperreactive spots (*) and thicknesses of INL and ONL (yellow and green lines, respectively). Arrows denote the B-scanned transects for thickness calculations. Scale bars = 100 μm. (**F**) Quantifications of the retinal thickness in 12-month-old zQ175 (n = 2) and matched-age wt (n = 4). Data are shown as mean ± SD. *p < 0.05; **p < 0.005, Student’s *t*-test between genotypes. (**G**) Quantification of DAPI-stained cells in the retinal ONL of 12-month-old mutant mice compared to wt. N = 3 for each genotype, n = 2–4 slices per animal. Data are shown as mean ± SD. **p < 0.005, Student’s *t*-test between wt and zQ175 mice.

The R6/1 strain is a rapidly progressive model that may exhibit extreme pathological phenotypical traits. To demonstrate that retinal inflammation is not a particularity of this transgenic model, we extended our analysis to heterozygous zQ175 KI mice, which better resemble the pathology in humans since the disease progression is much slower and comprises the loss-of-function component^23^. We demonstrated a significant downregulation of neuronal genes in both tissues but a specific induction of gliosis-related markers in the zQ175 retina, in which changes in 12-month-old mice were comparable to those occurring in R6/1 mice 13–15 weeks old (Fig. 5A,B); this induction was corroborated in immunohistochemistry assays against GFAP (Fig. 5C). In addition, microglia also exhibited a prominent amoeboid phenotype at the same age (Fig. 5D), together with aberrant hyperreactive spots in the retinal fundus (Fig. 5E) and a remarkable thinning (Fig. 5F) and nuclei loss (Fig. 5G) of the ONL, where the cell bodies of photoreceptors reside, as observed in the R6/1 retinas. Overall, both symptomatic zQ175 and R6/1 mice developed a similar behaviour of inflammatory markers in their retinas and striata, suggesting that our observations can be generalized to other HD mouse models.

## Discussion

The present work defines for the first time the correlation between the molecular alterations in the retina and striatum in HD mouse models. Retinopathy in HD has been described as a late event in the pathology, based on the normal fundus and minimal ERG anomalies in young R6/1 animals^17,20^. However, degeneration of specific cell types and altered retinal responses to light can occur in the R6/2 model prior to the onset of motor impairment and body weight loss^18,19^. Another polyQ disorder caused by an aberrant expansion of CAG triplets in the *ATXN7* gene, spinocerebellar ataxia 7 (SCA7), is characterized by retinal degeneration and dystrophy, leading to visual anomalies preceding motor symptoms^62^. HD and SCA7 animal models display common features of retinal anomalies at the level of morphology, light response and gene expression^20,63,64^, retinal dysfunction may share similar progression in both disorders and might manifest in early symptomatology. Notably, our gene expression analysis in the KI zQ175 strain suggested that photoreceptor genes can be affected prior to striatal-specific genes (Fig. 5).

Although gliosis has been previously reported in the R6/1 retina^17^, we provided for the first time evidence of microglial activation in the retina of two different HD models as a marker of neuroinflammation. Microglia comprise the resident phagocyte population in the CNS that can have both protective and deleterious effects, with anti-inflammatory and repair responses during the early phase of neurodegeneration but with harmful effects on neurons in prolonged activation in chronic inflammation^65–68^. In our RNA-seq analysis, we were able to detect different states of microglial activation, consistent with the hyperreactivity reported in microglia derived from pluripotent stem cells and blood-isolated myeloid cells of HD patients^69,70^. Reactive microglia induce the proinflammatory A1 astrocyte subtype, which is found in patients’ brains affected by different neurodegenerative disorders including HD^36^. In the R6/1 retina, we observed the induction of some of these astrocytic-related markers that mostly occurred in Müller cells, considering the GFAP staining pattern observed in the HD retinas. In any case, glial diversity falls beyond the dichotomous classification in A1 and A2, as reported in patients^71^.

Our striatal results were in agreement with the absence of overt neuroinflammation in HD models^24,72^. However, in HD patients activated glia and induction of inflammatory markers are well detected in cortical and basal ganglia regions^43,73,74^. This discrepancy is exemplified in a recent study that compared the gene expression profiles of murine R6/1 and zQ175 striata with those of human caudate nuclei from HD patients at a single nucleus resolution^75^. The induction of pan- and A1-astrocytic markers in human HD samples was reminiscent of our own results in the retina of R6/1 mice, suggesting that mHTT-expressing retinas might better reproduce the conditions of HD-associated neuroinflammatory processes, or at least might serve as experimental settings to study the HD-associated neuroinflammation thanks to the acceleration of these processes. Tightly connected to inflammatory processes, autophagy delivers aberrant organelles and macromolecules in double-membrane vesicles to lysosomes for degradation and recycling^54^. Deficits in this process can trigger neuroinflammation and cellular damage^76^ by modulating the functionality of immune and glial cells^77^. In HD autophagy becomes severely disrupted at several steps (e.g., cargo recognition, autophagosome formation, maturation and fusion to lysosomes), not only due to the toxic effects of the polyQ peptide but also because of the reduced regulatory activity of physiological HTT over autophagy, affecting the clearance of mHTT and contributing to its accumulation in cells^51^. We confirmed that autophagy was altered in the retina and striatum of R6/1 mice, contributing to the worsening of the disease, but apparently showed different rates of autophagosome accumulation, in which autophagic influx was stopped earlier in the R6/1 striatum than in the retina. The latter was transiently able to respond to mHTT. Whether the upregulation of lysosomal components (including the microglial cathepsin *Ctss*) is part of a compensatory mechanism for the HD-associated deficiency inautophagydeserves further exploration.

Based on our results, we propose that the retinas of HD mouse models can serve as a model to analyse HD-linked neuroinflammation, allowing for the use of ex vivo retina cultures from HD mouse models to elucidate mechanisms of neurodegeneration and to evaluate potential therapeutic approaches^78^, as research models that are evolutionarily closer than HD *Drosophila* ommatidia^79–82^.

In conclusion, the retinas of HD mouse models show profound morphological and functional abnormalities that are accompanied by a dramatic transcriptional dysregulation and glial activation, suggesting that retinal dysfunction may be of higher relevance in HD than envisaged. Studying this activation can provide novel insights regarding the role of macroglia and microglia during HD neuroinflammation.

### Supplementary Information

Supplementary Information.

## Data Availability

The RNA-seq data can be downloaded from the Gene Expression Omnibus (GEO) database using the accession number GSE216520.

## Abbreviations

*A2m*: Alpha-2-macroglobulin
*Actb*: Actin beta
*Arr3*: Arrestin 3
cAMP: Cyclic adenosine monophosphate
CNS: Central nervous system
*Ctss*: Cathepsin S
DAPI: 4’-6-Diamidino-2-phenylindole
DEG: Differentially expressed gene
ERG: Electroretinogram
FDR: False discovery rate
*Gapdh*: Glyceraldehyde-3-phosphate dehydrogenase
GCL: Ganglion cell layer
GFAP: Glial fibrillary acidic protein
*Gnat1*: G-Protein subunit alpha transducin 1
*H2-K1*: H-2 class I histocompatibility antigen, K-B alpha chain
HD: Huntington’s disease
*Hmox1*: Heme oxygenase 1
HTT: Huntingtin
Iba-1: Ionized calcium binding adaptor molecule 1
*Il1b*: Interleukin 1b
*Il6*: Interleukin 6
INL: Inner nuclear layer
IPL: Inner plexiform layer
IRF: Interferon regulator factor
KI: Knock-in
ONL: Outer nuclear layer
OPL: Outer plexiform layer
*Opn1sw*: Opsin 1, short wave sensitive
*Rho*: Rhodopsin
RNFL: Retinal nerve fibre layer
RT-qPCR: Retrotranscription and quantitative PCR
scRNA-seq: Single cell RNA sequencing
SD-OCT: Spectral domain optical coherence tomography
STAT: Signal transducer and activator of transcription
*Tbp*: TATA-box binding protein
TFBS: Transcription factor binding site
*Tnf*: Tumor necrosis factor
*Trf*: Transferrin
UHDRS: Unified Huntington’s disease rating scale
wt: Wild-type
