# Supplementary material for: Retinal dysfunction in Huntington’s disease mouse models concurs with local gliosis and microglia activation
Source: Sci Rep. 2024 Feb 20;14:4176. doi: 10.1038/s41598-024-54347-8 (PMC10879138; doi:10.1038/s41598-024-54347-8)
Supplement: Supplementary file 1 — Supplementary Information. [file 41598_2024_54347_MOESM1_ESM.pdf]

Supplementary material for "Retinal dysfunction in Huntington's disease mouse models concurs with local gliosis and microglia activation"

Fátima Cano-Cano, Francisco Martín-Loro, Andrea Gallardo-Orihuela, Maria del Carmen González-Montelongo, Samanta Ortuño-Miquel, Irati Hervás-Corpión, Pedro de la Villa, Lucía Ramón-Marco, Jorge Navarro-Calvo, Laura Gómez-Jaramillo, Ana I. Arroba, Luis M. Valor

Supplementary Figure S1. R6/1 retinas express mHTT and become functionally altered. *A*, Representative immunohistochemistry images of 25-week-old wt and R6/1 retinas against mHTT (EM48 antibody): whereas no signal was observed in wt slices, R6/1 exhibited strong signals at cellular layers. Scale = 50 µm. *B*, Representative ERG recordings of a wt and R6/1 mouse, depicting a-and b-wave amplitudes. n = 4 for each genotype.

Supplementary Figure S2. Prediction analysis of DNA motifs at the promoters of R6/1 deregulated genes. *A*, Pscan enrichment of Jaspar-based TFBS at the -950/+50 promoter regions in the subsets of DEG as defined in Figure 2A. TFBS in all subsets were ranked according to the Z-scores obtained for exclusive retinal DEG (*a* and *d* subsets). Inset denotes the Spearman correlation coefficients (ρ) between subsets of DEG. *B*, Sum of the numbers of significant TFBS ($p< 0.05$, Pscan) that were specific or common (overlapping) to any subset of DEG, after comparing *a*, *b*, *c* (down) or *d*, *e*, *f* (up). Note that the differential result between down- and up-regulated genes was not influenced by the numbers of TFBS as they were similar between both groups of DEG. *C*, Z-scores from the enrichment analysis of TFBS related to inflammation (IRF, STAT) in the subsets of DEG. *, *p*-value < 0.05; **, *p*-value < 0.005 (Pscan).

Supplementary Figure S3. Raw western blots of Figure 2. *A*, retina, *B*, striatum.

Supplementary Figure S4. Gene expression changes in autophagy of the R6/1 retina and striatum. *A*, Proportion of autophagic-related genes belonging to the categories "mTOR and upstream pathways", "autophagy core", "autophagy regulators", "mitophagy", "docking and fusion", "lysosome" and "lysosome-related" in the RNA-seq data from 13-15 weeks-old R6/1 mice, considering the number of genes within each category and within DEGs in R6/1 tissues. Numbers indicate the number of DEG (adj. p-value < 0.05). The only significant difference was observed between retinal down- and up-regulated genes in the lysosomal category, indicated by * (χ^2= 12.13, d.f. = 5, p-value = 0.033). *B*, Heatmap plot of the fold changes of DEG belonging to the "Lysosome biogenesis" signature described in the text. ¶, adjusted p-value < 0.05 from the RNA-seq analysis. *, p-value < 0.05, Student's t-test between the means of both tissues.

Supplementary Figure S5. Number of CAG repeats and R6/1 transgene expression in the R6/1 retina and striatum. *A*, Representative electropherograms of DNA fragment analysis in the striatum and the retina of a mutant mouse (left panel). Quantification of the results from ten R6/1 mice (right panel): only a net single CAG repeat was different between both tissues (Δ). *B*, RT-qPCR assays of R6/1 retinas and striata (n =13 for each tissue) from 5 and 7-week-old mice, prior to any potential cell loss that might influence the result. Transgene expression was normalized by *Eef2* values as this gene was equally expressed in both tissues in the RNA-seq data (Supplementary Fig. S5). Data are expressed as mean ± s.e.m.

Supplementary Figure S6. Gene expression analysis of additional inflammatory markers. *A*, Table showing the normalized counts of all samples (baseMeans of Deseq2 results) for specific markers for B-, T, and NK-cells. The housekeeping *Eef2* is indicated as an example of a well expressed gene. NA, not available due to very low

expression. *B*, Unnormalized C_T values of the qPCR for *Il1b*, *Il6*, *Tnf* and *Hmox1* in the retina and the striatum of R6/1 and wild-type littermates, using the same threshold; only *Hmox1* was well expressed beyond noise, and fold change between genotypes was calculated. Data are expressed as mean ± s.e.m.

Supplementary Figure S1. R6/1 retinas express mHTT and becdome functionally affected

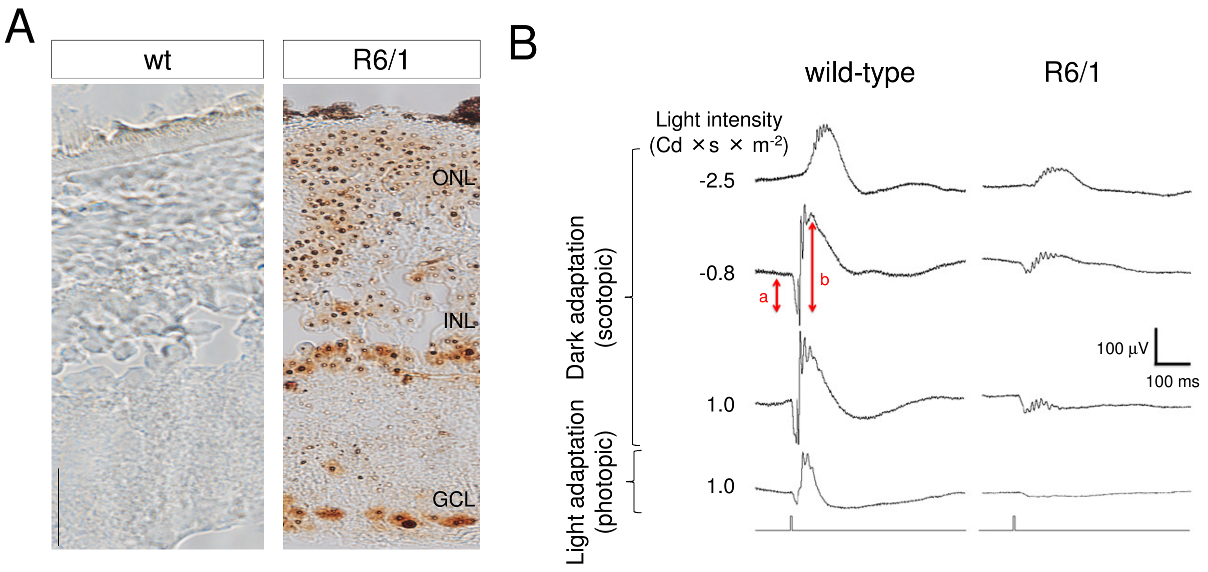

Supplementary Figure S2. Prediction analysis of DNA motifs at the promoters of R6/1 deregulated genes

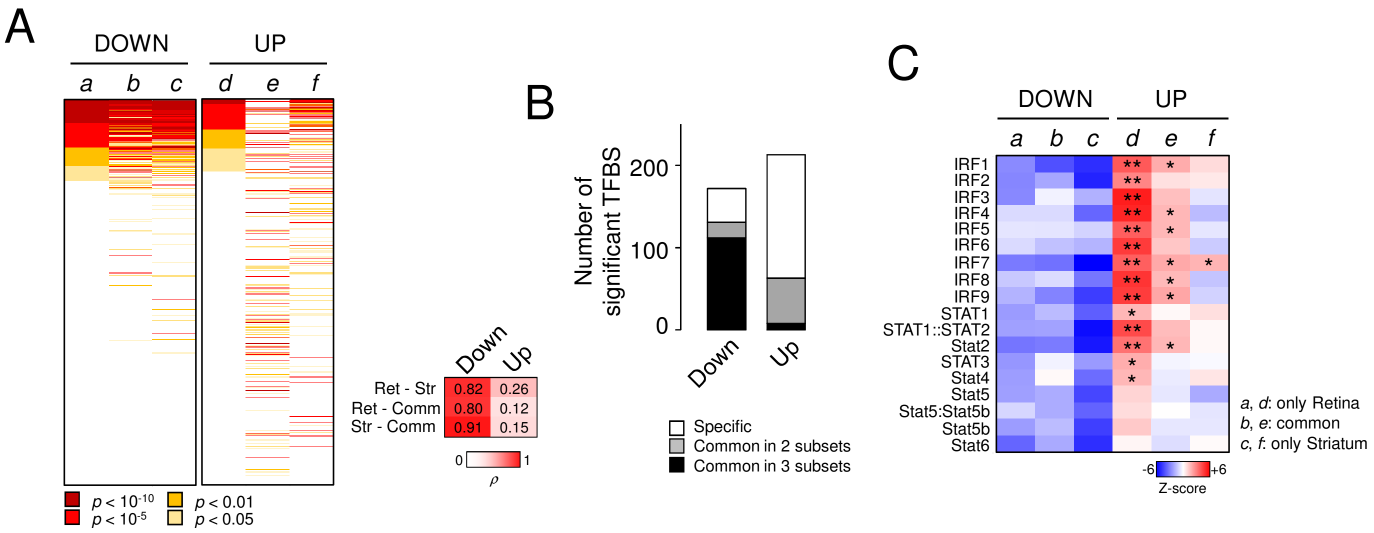

Supplementary Figure S3. Raw western blots of Figure 2

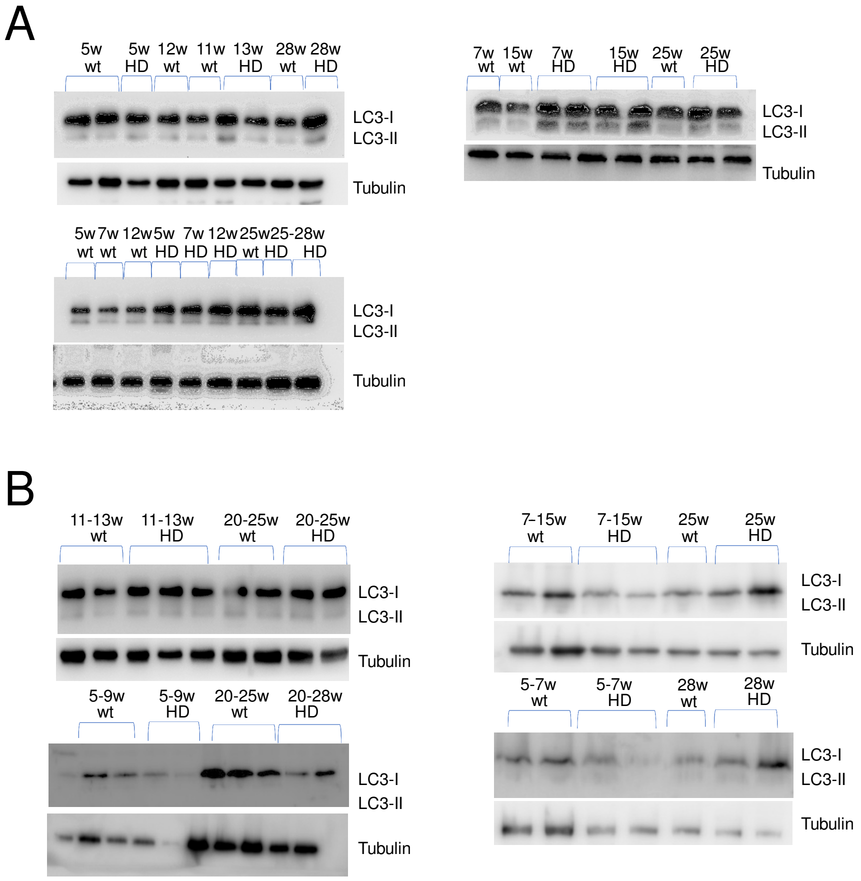

Supplementary Figure S4. Gene expression changes in the autophagy of R6/1 retina and striatum

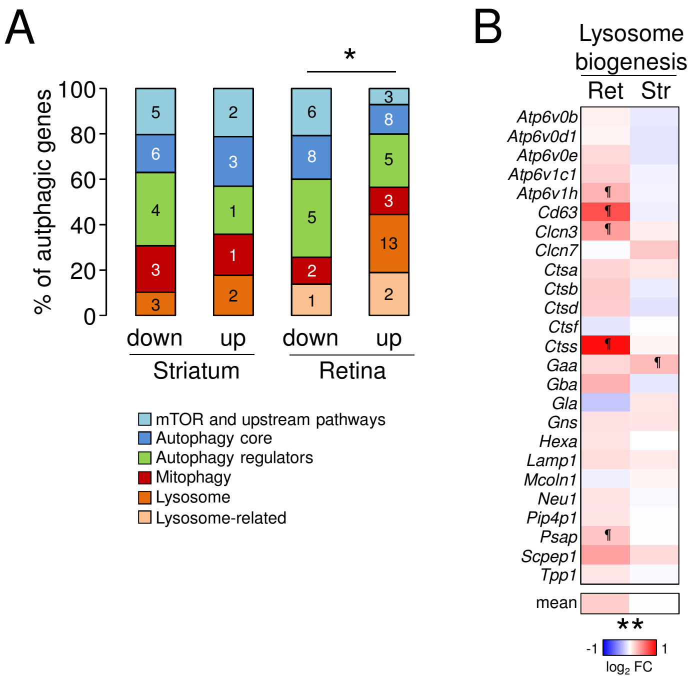

Supplementary Figure S5. Number of CAG repeats and R6/1 transgene expression in the R6/1 retina and striatum

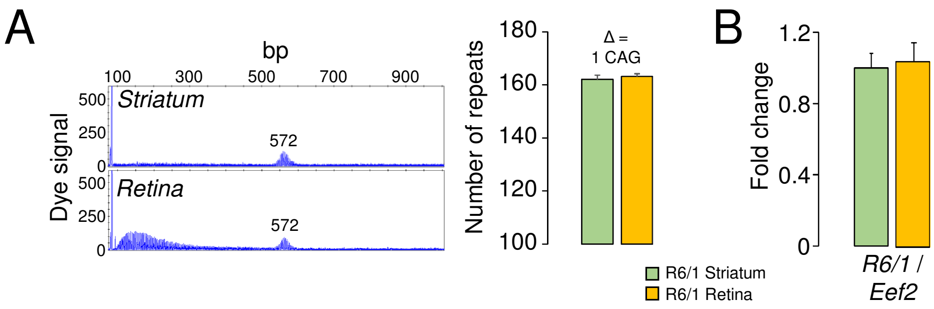

Supplementary Figure S6. Gene expression analysis of additional inflammatory markers

A

Gene	Marker	Retina	Striatum
Cd22	B-cell	NA	6.7
Cd80	B-cell	NA	7.5
Cd86	B-cell	4.6	46.0
Cd180	B-cell	10.5	83.8
Cr2	B-cell	5.3	10.6
Fcer2a/CD23	B-cell	NA	NA
Ms4a1/CD20	B-cell	NA	2.3
Tnfsf4/CD252	B-cell	NA	NA
Cd3d	NK & T-cells	NA	NA
Cd3e	NK & T-cells	NA	NA
Cd3g	NK & T-cells	NA	NA
Cd247	NK & T-cells	2.9	2.3
Btla	T-cell (CD4+)	12.5	18.9
Cd4	T-cell (CD4+)	1.9	550.9
Cd40lg	T-cell (CD4+)	NA	NA
Cd69	T-cell (CD4+)	NA	NA
Cd7	T-cell (CD4+)	NA	NA
Ctla4	T-cell (CD4+)	NA	2.0
Icos	T-cell (CD4+)	NA	NA
Il2ra/CD25	T-cell (CD4+)	NA	6.6
Il7r/CD127	T-cell (CD4+)	NA	9.7
Pdcd1	T-cell (CD4+)	NA	NA
Sell/CD62l	T-cell (CD4+)	2.1	3.3
Tnfrsf9/CD137	T-cell (CD4+)	7.7	15.1
Tnfrsf4/CD134	T-cell (CD4+)	8.3	8.9
Cd5	T-cell (CD8+)	NA	NA
Cd8b1	T-cell (CD8+)	NA	NA
Cd8b2	T-cell (CD8+)	NA	NA
Cd27	T-cell (CD8+)	NA	NA
Cd28	T-cell (CD8+)	2.5	4.3
Eef2	Housekeep.	17345.4	17449.8

B

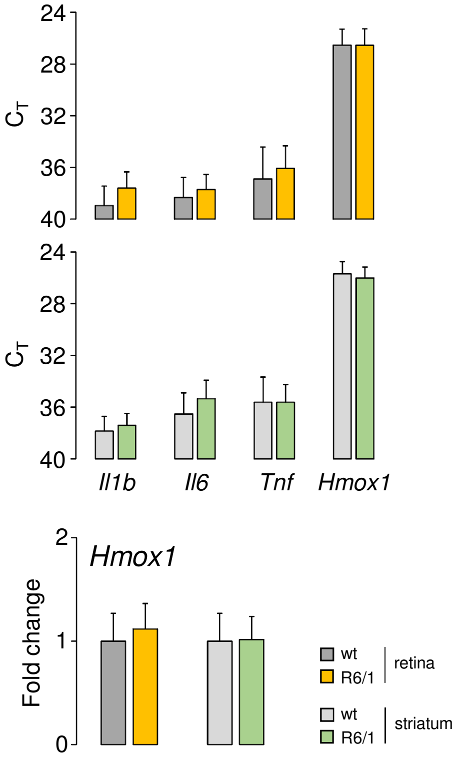

Supplementary Table S1. Sequences of the primers used in the present study.

Gene	Primer	Sequence (5' → 3')
Opn1sw	Forward<br>Reverse	TCATCTGTAAACCCTTCGGCA<br>CCAGGTGTAGTACTCGCTTCG
Arr3	Forward<br>Reverse	AAGAAGACTAGCTCCAATGGG<br>TCCAAGTCATCACGGCCATAG
Rho	Forward<br>Reverse	CCAACTTCGGCCCCATCTT<br>CAGAGGCGTCGTCATCTCC
Gnat1	Forward<br>Reverse	CCACTGAGCAGGACGTGTT<br>GATGCACGTCACACCCTCAAA
Stk35	Forward<br>Reverse	CTCTGCAGACAAGCCCGG<br>CTTCTCGCACAGTACCCGC
Plk5	Forward<br>Reverse	CACCTGTGTTTGCCTTTCCC<br>TTTTGAGGTAAAGGGACAAGGTG
Scn4b	Forward<br>Reverse	CATGGTTTAGTCCTCTGGCTTGG<br>CACGGCCCACCACTGTATTG
Arpp19	Forward<br>Reverse	GCAAAGTTAAAGGCAAGGTATCC<br>CTCTGTCTTATCCGGGGCTG
Pde10a	Forward<br>Reverse	TAACAATGCGAGTTGCTTCC<br>CAGCCACTTTTCCACAGTCTC
Rgs4	Forward<br>Reverse	TCTCTCCTCGCTAAGAATCCCT<br>GTTTTCCAACGATTCAGCCCA
Penk	Forward<br>Reverse	GGCGTGCACACTGGAATGT<br>TCCCAGATTTTGAAAGAAGGCA
Polr2a	Forward<br>Reverse	GAGTTTCGGCTGAGTGGAGAG<br>AGCGTCACATTCTTGGCAGAT
Gfap	Forward<br>Reverse	GGACAACTTTGCACAGGACCTC<br>TCCAAATCCACACGAGCCA
A2m	Forward<br>Reverse	GCAGAATTTCCGCTTAGAGGG<br>GAAAGCCAGCGATTCTTCACC
Il1b	Forward<br>Reverse	CAGGCAGGCAGTATCACTCAT<br>CTAATGGGAACGTCACACACC
Il6	Forward<br>Reverse	GAGGATACCACTCCCAACAGACC<br>AAGTGCATATCGTTGTTCATACA
Tnf	Forward<br>Reverse	CATCTTCTCAAAATTCGAGTGACAA<br>TGGGAGTAGACAAGGTACAACCC
Hmox1	Forward<br>Reverse	CACAGATGGCGTCACTTCGTC<br>GTGAGGACCCACTGGAGGAG
Tbp	Forward<br>Reverse	TAAGAGAGCCACGGACAACTG<br>CAAGGCCTTCCAGCCTTATAG
Eef2	Forward<br>Reverse	TCACAATCAAATCCACCGCC<br>ATGGCCTGGAGAGTCGATGA

Supplementary Table S2. Differential expression (RNA-seq results) of the retina and the striatum of 13-15-month-old R6/1 and wildtype littermates.

		Retina					
Ensembl Gene ID	Gene symbol	baseMean	log2FoldChange	lfcSE	stat	pvalue	padj
ENSMUSG00000060890	Arr3	1576.200	-3.734	0.134	-27.783	6.9E-170	1.0E-165
ENSMUSG00000058831	Opn1sw	3449.240	-8.793	0.342	-25.680	2.0E-145	1.5E-141
ENSMUSG00000030324	Rho	360755.633	-1.619	0.072	-22.374	7.1E-111	3.5E-107
ENSMUSG00000060044	Tmem26	428.680	4.780	0.222	21.536	7.2E-103	2.6E-99
ENSMUSG00000009108	Gnat2	1925.390	-2.043	0.103	-19.847	1.2E-87	3.4E-84
ENSMUSG00000037885	Stk35	8193.659	-1.397	0.073	-19.134	1.3E-81	3.2E-78
ENSMUSG00000031450	Grk1	34790.547	-1.219	0.067	-18.240	2.5E-74	5.2E-71
ENSMUSG00000035504	Reep6	8050.684	-1.783	0.101	-17.637	1.3E-69	2.4E-66
ENSMUSG00000113924	Gm48493	3347.553	-1.404	0.082	-17.203	2.5E-66	4.1E-63
ENSMUSG00000056043	Rgs9bp	14878.964	-1.202	0.070	-17.192	3.0E-66	4.5E-63
ENSMUSG00000025340	Rabgef1	6547.888	-1.650	0.098	-16.811	2.0E-63	2.7E-60
ENSMUSG00000034837	Gnat1	113352.312	-1.467	0.091	-16.157	1.0E-58	1.2E-55
ENSMUSG00000096740	Lbhd1	5551.329	-1.320	0.084	-15.810	2.7E-56	3.0E-53
ENSMUSG00000050556	Kcnb1	22346.377	-1.010	0.064	-15.733	8.9E-56	9.3E-53
ENSMUSG00000056055	Sag	52344.019	-1.373	0.089	-15.510	3.0E-54	2.9E-51
ENSMUSG00000033066	Gas7	3045.495	-1.363	0.091	-14.985	9.3E-51	8.5E-48
ENSMUSG00000023978	Prph2	50248.206	-1.184	0.080	-14.869	5.2E-50	4.5E-47
ENSMUSG00000026409	Pfkfb2	6383.635	-1.145	0.077	-14.791	1.7E-49	1.4E-46
ENSMUSG00000029672	Fam3c	5678.668	-1.257	0.085	-14.733	4.0E-49	3.1E-46
ENSMUSG00000096351	Samd11	4793.326	-1.351	0.093	-14.584	3.6E-48	2.6E-45
ENSMUSG00000020886	Dlg4	9926.741	-1.018	0.070	-14.485	1.5E-47	1.1E-44
ENSMUSG00000039672	Kcne2	575.186	-2.243	0.156	-14.421	3.8E-47	2.5E-44
ENSMUSG00000021255	Esrrb	3629.959	-1.164	0.083	-14.088	4.5E-45	2.9E-42
ENSMUSG00000025860	Xiap	12650.828	-0.928	0.066	-13.990	1.8E-44	1.1E-41
ENSMUSG00000021221	Dpf3	1629.206	-1.351	0.097	-13.980	2.1E-44	1.2E-41
ENSMUSG00000024992	Pde6c	1600.562	-1.542	0.111	-13.915	5.1E-44	2.9E-41
ENSMUSG00000006007	Pdc	40810.022	-1.130	0.081	-13.860	1.1E-43	6.0E-41
ENSMUSG00000029064	Gnb1	78007.525	-1.249	0.090	-13.808	2.3E-43	1.2E-40
ENSMUSG00000036030	Prtg	467.184	2.639	0.194	13.585	4.9E-42	2.5E-39
ENSMUSG00000040543	Pitpnm3	4797.091	-1.135	0.085	-13.395	6.4E-41	3.1E-38
ENSMUSG00000054792	Klhl18	4492.072	-1.038	0.078	-13.394	6.5E-41	3.1E-38
ENSMUSG00000024575	Pde6a	51058.095	-0.858	0.065	-13.236	5.4E-40	2.5E-37
ENSMUSG00000034829	Nxnl1	3453.637	-1.245	0.094	-13.202	8.5E-40	3.8E-37
ENSMUSG00000027360	Hdc	515.389	-2.505	0.190	-13.167	1.4E-39	5.9E-37
ENSMUSG00000005583	Mef2c	3467.954	-1.176	0.090	-13.122	2.5E-39	1.0E-36
ENSMUSG00000001211	Agpat3	21326.453	-0.891	0.068	-13.100	3.3E-39	1.3E-36
ENSMUSG00000030096	Slc6a6	22844.862	-0.847	0.066	-12.856	8.0E-38	3.2E-35
ENSMUSG00000034320	Slc26a2	2013.565	-1.182	0.093	-12.751	3.1E-37	1.2E-34
ENSMUSG00000022836	Mylk	1221.897	-1.364	0.107	-12.725	4.3E-37	1.6E-34
ENSMUSG00000024227	Pdzph1	6673.833	-1.008	0.080	-12.631	1.4E-36	5.2E-34
ENSMUSG00000021991	Cacna2d3	1046.649	-1.469	0.117	-12.599	2.1E-36	7.7E-34
ENSMUSG00000089917	Uckl1	2202.875	-1.193	0.095	-12.577	2.8E-36	9.8E-34
ENSMUSG00000036395	Glb1l2	3164.738	-1.120	0.090	-12.459	1.3E-35	4.3E-33
ENSMUSG00000014164	Klhl3	1214.559	-1.326	0.107	-12.379	3.4E-35	1.1E-32
ENSMUSG00000025900	Rp1	50134.517	-0.781	0.064	-12.265	1.4E-34	4.5E-32
ENSMUSG00000003657	Calb2	2349.212	-1.083	0.089	-12.215	2.6E-34	8.3E-32
ENSMUSG00000030111	A2m	531.721	2.349	0.192	12.208	2.8E-34	8.8E-32
ENSMUSG00000023979	Guca1b	11330.705	-1.097	0.090	-12.202	3.0E-34	9.2E-32
ENSMUSG00000041920	Slc16a6	3699.366	-1.093	0.090	-12.139	6.5E-34	2.0E-31
ENSMUSG00000025437	Usp33	6727.867	-0.855	0.070	-12.124	7.9E-34	2.3E-31
ENSMUSG00000024901	Peli3	1273.945	-1.425	0.118	-12.049	2.0E-33	5.5E-31
ENSMUSG00000031293	Rs1	42074.926	-0.859	0.071	-12.051	1.9E-33	5.5E-31

ENSMUSG00000054717	Hmgb2	979.710	-1.595	0.134	-11.938	7.5E-33	2.1E-30
ENSMUSG00000041308	Sntb2	3239.416	-0.996	0.085	-11.756	6.6E-32	1.8E-29
ENSMUSG00000025076	Casp7	476.795	-1.894	0.161	-11.749	7.1E-32	1.9E-29
ENSMUSG00000079550	Mpp4	7765.019	-0.848	0.072	-11.703	1.2E-31	3.2E-29
ENSMUSG00000003500	Impdh1	4942.017	-1.011	0.086	-11.701	1.3E-31	3.3E-29
ENSMUSG00000032554	Trf	2278.298	1.237	0.106	11.692	1.4E-31	3.6E-29
ENSMUSG00000058966	Tlcd3b	8113.972	-1.036	0.090	-11.573	5.6E-31	1.4E-28
ENSMUSG00000031966	Glb1l3	343.320	-2.686	0.233	-11.511	1.2E-30	2.8E-28
ENSMUSG00000071984	Fndc1	356.934	-2.391	0.208	-11.470	1.9E-30	4.5E-28
ENSMUSG00000078588	Ccdc24	1875.777	-1.178	0.103	-11.433	2.9E-30	6.7E-28
ENSMUSG00000079173	Zan	147.136	-3.290	0.291	-11.315	1.1E-29	2.6E-27
ENSMUSG00000054150	Syne3	1574.068	-1.116	0.099	-11.305	1.2E-29	2.8E-27
ENSMUSG00000031491	Chrna6	1434.540	-1.129	0.101	-11.161	6.3E-29	1.4E-26
ENSMUSG00000055865	Tafa3	1232.669	-1.226	0.110	-11.158	6.6E-29	1.5E-26
ENSMUSG00000005124	Ccn4	993.270	-1.297	0.117	-11.060	2.0E-28	4.3E-26
ENSMUSG00000030956	Fam53b	1959.083	-1.174	0.106	-11.054	2.1E-28	4.5E-26
ENSMUSG00000034452	Slc24a1	26273.046	-0.818	0.074	-10.999	3.9E-28	8.2E-26
ENSMUSG00000029491	Pde6b	36033.634	-0.834	0.076	-10.980	4.8E-28	1.0E-25
ENSMUSG00000118515	Gm53026	1505.552	-1.171	0.107	-10.967	5.5E-28	1.1E-25
ENSMUSG00000021123	Rdh12	5728.527	-1.055	0.097	-10.864	1.7E-27	3.5E-25
ENSMUSG00000058743	Kcnj14	6636.279	-0.840	0.078	-10.832	2.4E-27	4.9E-25
ENSMUSG00000002058	Unc119	10189.062	-0.883	0.082	-10.810	3.1E-27	6.1E-25
ENSMUSG00000004040	Stat3	1261.827	1.186	0.111	10.731	7.3E-27	1.4E-24
ENSMUSG00000027589	Pcmtd2	16352.145	-0.690	0.064	-10.718	8.4E-27	1.6E-24
ENSMUSG00000079042	Apela	324.809	2.010	0.188	10.683	1.2E-26	2.3E-24
ENSMUSG00000067220	Cnga1	17921.973	-0.908	0.085	-10.637	2.0E-26	3.8E-24
ENSMUSG00000022055	Nefl	6319.921	-0.785	0.074	-10.538	5.8E-26	1.1E-23
ENSMUSG00000018362	Kpna2	4547.260	-1.062	0.101	-10.493	9.3E-26	1.7E-23
ENSMUSG00000043987	Cep164	3807.959	-0.820	0.078	-10.480	1.1E-25	1.9E-23
ENSMUSG00000035181	Heatr5a	1797.646	-1.068	0.102	-10.445	1.5E-25	2.8E-23
ENSMUSG00000032413	Rasa2	1804.297	1.068	0.103	10.377	3.1E-25	5.6E-23
ENSMUSG00000023982	Guca1a	8554.442	-0.940	0.091	-10.338	4.8E-25	8.3E-23
ENSMUSG00000024026	Glo1	6144.870	0.898	0.087	10.336	4.8E-25	8.3E-23
ENSMUSG00000027797	Dclk1	1727.755	-1.039	0.101	-10.325	5.4E-25	9.3E-23
ENSMUSG00000057337	Chst3	1017.848	-1.316	0.128	-10.318	5.8E-25	9.8E-23
ENSMUSG00000020890	Gucy2e	7870.589	-0.899	0.088	-10.188	2.3E-24	3.8E-22
ENSMUSG00000038886	Man2a2	9730.347	-0.676	0.066	-10.161	3.0E-24	4.9E-22
ENSMUSG00000038963	Slco4a1	1445.193	-1.018	0.101	-10.114	4.8E-24	7.8E-22
ENSMUSG00000019867	Gje1	99.369	4.552	0.452	10.075	7.2E-24	1.2E-21
ENSMUSG00000021364	Elovl2	2509.977	0.853	0.085	10.003	1.5E-23	2.4E-21
ENSMUSG00000019970	Sgk1	5854.895	-0.813	0.081	-9.992	1.7E-23	2.6E-21
ENSMUSG00000026457	Adipor1	6578.264	-0.852	0.086	-9.960	2.3E-23	3.6E-21
ENSMUSG00000004655	Aqp1	1259.314	-1.181	0.120	-9.871	5.5E-23	8.4E-21
ENSMUSG00000031394	Opn1mw	871.852	-10.756	1.090	-9.872	5.5E-23	8.4E-21
ENSMUSG00000018076	Med13l	3689.999	-0.775	0.079	-9.861	6.1E-23	9.3E-21
ENSMUSG00000039307	Hexdc	1076.536	-1.076	0.112	-9.611	7.2E-22	1.1E-19
ENSMUSG00000034714	Ttyh2	793.110	1.215	0.127	9.585	9.3E-22	1.4E-19
ENSMUSG00000041528	Rnf123	2152.964	-0.897	0.094	-9.571	1.1E-21	1.6E-19
ENSMUSG00000019978	Epb41l2	20917.700	-0.618	0.065	-9.452	3.3E-21	4.8E-19
ENSMUSG00000048617	Rtbdn	4672.763	-0.828	0.088	-9.446	3.5E-21	5.0E-19
ENSMUSG00000032249	Anp32a	9453.490	-0.682	0.072	-9.438	3.8E-21	5.4E-19
ENSMUSG00000029410	Ppef2	2552.906	-0.820	0.087	-9.425	4.3E-21	6.0E-19
ENSMUSG00000044724	Gpr152	1371.135	-1.019	0.108	-9.412	4.9E-21	6.8E-19
ENSMUSG00000053205	Styx	2475.844	-0.890	0.095	-9.386	6.2E-21	8.6E-19
ENSMUSG00000056752	Dnah9	316.400	-1.843	0.197	-9.377	6.8E-21	9.3E-19
ENSMUSG00000041578	Crx	17373.723	-0.598	0.064	-9.343	9.4E-21	1.3E-18

ENSMUSG00000039285	Azi2	2993.335	-0.796	0.086	-9.271	1.9E-20	2.5E-18
ENSMUSG00000032087	Dscaml1	2161.750	-0.957	0.103	-9.266	1.9E-20	2.6E-18
ENSMUSG00000028015	Ctso	794.125	1.178	0.127	9.246	2.3E-20	3.1E-18
ENSMUSG00000035517	Tdrd7	4672.847	-0.860	0.093	-9.225	2.8E-20	3.7E-18
ENSMUSG00000037318	Traf3ip3	259.798	-1.952	0.212	-9.223	2.9E-20	3.7E-18
ENSMUSG00000039629	Strip2	1678.052	-0.990	0.107	-9.220	3.0E-20	3.8E-18
ENSMUSG00000063681	Crb1	9616.252	-0.618	0.067	-9.220	3.0E-20	3.8E-18
ENSMUSG00000020889	Nr1d1	1705.668	-0.911	0.099	-9.193	3.8E-20	4.8E-18
ENSMUSG00000020932	Gfap	391.133	1.654	0.180	9.187	4.0E-20	5.1E-18
ENSMUSG00000052609	Plekhg3	1066.811	-1.047	0.114	-9.150	5.7E-20	7.1E-18
ENSMUSG00000037446	Tulp1	14876.028	-0.670	0.073	-9.137	6.4E-20	7.9E-18
ENSMUSG00000032936	Camkv	851.552	-1.106	0.121	-9.102	8.9E-20	1.1E-17
ENSMUSG00000025386	Pde6g	16531.741	-0.876	0.096	-9.089	1.0E-19	1.2E-17
ENSMUSG00000023439	Gnb3	4559.770	0.739	0.081	9.086	1.0E-19	1.2E-17
ENSMUSG00000041642	Kif21b	5512.533	-0.669	0.074	-9.083	1.1E-19	1.3E-17
ENSMUSG00000041817	Fam169a	16382.892	-0.594	0.065	-9.077	1.1E-19	1.3E-17
ENSMUSG00000096887	Gm20594	11184.238	0.680	0.075	9.071	1.2E-19	1.4E-17
ENSMUSG00000050965	Prkca	5772.441	-0.728	0.080	-9.053	1.4E-19	1.6E-17
ENSMUSG00000052698	Tln2	2976.126	-0.763	0.084	-9.040	1.6E-19	1.8E-17
ENSMUSG00000017344	Vtn	7648.941	-0.801	0.089	-9.038	1.6E-19	1.8E-17
ENSMUSG00000032192	Gnb5	9748.759	-0.653	0.072	-9.023	1.8E-19	2.1E-17
ENSMUSG00000026819	Slc25a25	1782.807	-0.944	0.105	-8.996	2.3E-19	2.6E-17
ENSMUSG00000029338	Antxr2	509.920	1.458	0.162	8.995	2.4E-19	2.6E-17
ENSMUSG00000061288	Taok3	2741.193	-0.744	0.083	-8.978	2.8E-19	3.1E-17
ENSMUSG00000099032	Tcf24	239.068	1.911	0.214	8.923	4.6E-19	5.0E-17
ENSMUSG00000047298	Kcnv2	10944.042	-0.705	0.079	-8.918	4.7E-19	5.2E-17
ENSMUSG00000015243	Abca1	1941.941	0.919	0.104	8.815	1.2E-18	1.3E-16
ENSMUSG00000057469	E2f6	741.495	1.133	0.129	8.808	1.3E-18	1.4E-16
ENSMUSG00000028957	Per3	1673.367	-0.961	0.110	-8.774	1.7E-18	1.8E-16
ENSMUSG00000025582	Nptx1	648.422	-1.251	0.143	-8.757	2.0E-18	2.1E-16
ENSMUSG00000029769	Ccdc136	1289.891	-1.054	0.120	-8.754	2.1E-18	2.2E-16
ENSMUSG00000030283	St8sia1	3746.560	-0.672	0.077	-8.679	4.0E-18	4.2E-16
ENSMUSG00000054321	Taf4b	617.094	-1.349	0.156	-8.661	4.7E-18	4.9E-16
ENSMUSG00000031789	Cngb1	11592.819	-0.651	0.076	-8.609	7.4E-18	7.6E-16
ENSMUSG00000037325	Bbs7	3999.315	-0.681	0.079	-8.605	7.7E-18	7.8E-16
ENSMUSG00000028542	Slc6a9	1388.780	-0.875	0.102	-8.584	9.2E-18	9.3E-16
ENSMUSG00000027168	Pax6	4565.999	-0.640	0.075	-8.570	1.0E-17	1.0E-15
ENSMUSG00000002957	Ap2a2	4859.750	-0.663	0.077	-8.555	1.2E-17	1.2E-15
ENSMUSG00000027676	Ccdc39	788.546	1.163	0.136	8.528	1.5E-17	1.5E-15
ENSMUSG00000030402	Ppm1n	769.173	-1.106	0.130	-8.492	2.0E-17	2.0E-15
ENSMUSG00000030523	Trpm1	4657.485	-0.720	0.085	-8.445	3.0E-17	3.0E-15
ENSMUSG00000038515	Grtp1	1273.901	-1.091	0.129	-8.440	3.2E-17	3.1E-15
ENSMUSG00000030654	Arl6ip1	5057.866	0.734	0.087	8.438	3.2E-17	3.1E-15
ENSMUSG00000027674	Pex5l	7253.530	-0.643	0.076	-8.432	3.4E-17	3.3E-15
ENSMUSG00000046110	Serinc4	1124.303	-1.088	0.129	-8.412	4.0E-17	3.9E-15
ENSMUSG00000089669	Tnfsf13	362.156	-1.555	0.186	-8.382	5.2E-17	4.9E-15
ENSMUSG00000028222	Calb1	1088.432	-1.027	0.123	-8.377	5.4E-17	5.1E-15
ENSMUSG00000038370	Pcp4l1	1000.769	-0.962	0.115	-8.374	5.6E-17	5.2E-15
ENSMUSG00000022722	Arl6	3680.064	0.738	0.089	8.307	9.8E-17	9.2E-15
ENSMUSG00000004630	Pcp2	1274.968	-1.001	0.121	-8.302	1.0E-16	9.5E-15
ENSMUSG00000055368	Slc6a2	75.605	4.072	0.492	8.268	1.4E-16	1.3E-14
ENSMUSG00000031142	Cacna1f	6385.446	-0.648	0.079	-8.249	1.6E-16	1.5E-14
ENSMUSG00000041534	Rbp3	37354.305	-0.515	0.063	-8.211	2.2E-16	2.0E-14
ENSMUSG00000033416	Gucd1	895.367	-0.966	0.118	-8.164	3.2E-16	2.9E-14
ENSMUSG00000111551	Gm48128	2403.885	-0.795	0.098	-8.126	4.5E-16	4.0E-14
ENSMUSG00000020435	Osbp2	8262.383	-0.609	0.075	-8.123	4.6E-16	4.1E-14

ENSMUSG00000028649	Macf1	12254.262	-0.639	0.079	-8.068	7.1E-16	6.3E-14
ENSMUSG00000021384	Susd3	996.210	-0.970	0.120	-8.049	8.4E-16	7.4E-14
ENSMUSG00000024842	Cabp4	3119.289	-0.800	0.100	-8.028	9.9E-16	8.7E-14
ENSMUSG00000020907	Rcvrn	8292.140	-0.723	0.090	-8.027	1.0E-15	8.7E-14
ENSMUSG00000058498	Rnf207	2535.544	-0.831	0.104	-8.008	1.2E-15	1.0E-13
ENSMUSG00000067158	Col4a4	317.062	-1.838	0.230	-7.984	1.4E-15	1.2E-13
ENSMUSG00000034832	Tet3	3515.550	-0.656	0.082	-7.977	1.5E-15	1.3E-13
ENSMUSG00000028635	Edn2	103.382	2.862	0.360	7.961	1.7E-15	1.5E-13
ENSMUSG00000036473	Tbc1d24	3943.811	-0.662	0.083	-7.953	1.8E-15	1.5E-13
ENSMUSG00000026885	Ttll11	345.213	-1.654	0.208	-7.942	2.0E-15	1.7E-13
ENSMUSG00000051855	Mest	1335.476	0.817	0.103	7.918	2.4E-15	2.0E-13
ENSMUSG00000051977	Prdm9	783.759	-1.108	0.140	-7.913	2.5E-15	2.1E-13
ENSMUSG00000027715	Ccna2	581.550	-1.339	0.170	-7.893	2.9E-15	2.4E-13
ENSMUSG00000032599	Ip6k2	2016.207	-0.716	0.091	-7.890	3.0E-15	2.5E-13
ENSMUSG00000029561	Oasl2	375.263	1.353	0.172	7.858	3.9E-15	3.2E-13
ENSMUSG00000018427	Ypel2	3594.636	-0.837	0.107	-7.839	4.5E-15	3.7E-13
ENSMUSG00000042726	Trafd1	2786.414	-0.682	0.087	-7.819	5.3E-15	4.3E-13
ENSMUSG00000020212	Mdm1	3296.123	-0.626	0.080	-7.801	6.1E-15	4.9E-13
ENSMUSG00000044375	Pcare	5778.243	-0.591	0.076	-7.791	6.7E-15	5.3E-13
ENSMUSG00000079465	Col4a3	808.006	-3.032	0.390	-7.767	8.0E-15	6.4E-13
ENSMUSG00000021086	Ccdc175	72.550	3.441	0.443	7.763	8.3E-15	6.6E-13
ENSMUSG00000019772	Vip	107.057	-2.476	0.320	-7.749	9.3E-15	7.3E-13
ENSMUSG00000055447	Cd47	2575.052	0.684	0.088	7.741	9.8E-15	7.7E-13
ENSMUSG00000029762	Akr1b8	76.802	3.111	0.402	7.730	1.1E-14	8.4E-13
ENSMUSG00000035486	Plk5	1100.363	-0.878	0.114	-7.728	1.1E-14	8.5E-13
ENSMUSG00000020599	Rgs9	11936.641	-0.510	0.066	-7.724	1.1E-14	8.7E-13
ENSMUSG00000074259	Gramd2	1561.008	-0.885	0.115	-7.720	1.2E-14	9.0E-13
ENSMUSG00000071647	Eml3	2225.384	-0.922	0.120	-7.653	2.0E-14	1.5E-12
ENSMUSG00000061232	H2-K1	453.769	1.206	0.158	7.649	2.0E-14	1.5E-12
ENSMUSG00000064330	Pde6h	995.620	-2.376	0.312	-7.627	2.4E-14	1.8E-12
ENSMUSG00000090035	Galnt4	1574.517	-0.787	0.103	-7.621	2.5E-14	1.9E-12
ENSMUSG00000050619	Zscan29	1279.391	-0.800	0.105	-7.589	3.2E-14	2.4E-12
ENSMUSG00000025867	Cplx2	2573.157	-0.653	0.086	-7.579	3.5E-14	2.6E-12
ENSMUSG00000030256	Bhlhe41	1355.888	-0.800	0.106	-7.571	3.7E-14	2.7E-12
ENSMUSG00000005233	Spc25	1902.745	-0.757	0.100	-7.564	3.9E-14	2.9E-12
ENSMUSG00000041488	Stx3	14818.520	-0.514	0.068	-7.558	4.1E-14	3.0E-12
ENSMUSG00000061536	Sec22c	1321.469	0.811	0.107	7.558	4.1E-14	3.0E-12
ENSMUSG00000071646	Mta2	1937.682	-0.725	0.096	-7.551	4.3E-14	3.1E-12
ENSMUSG00000037224	Zfyve28	989.724	-0.879	0.117	-7.511	5.9E-14	4.2E-12
ENSMUSG00000026672	Optn	3210.959	-0.627	0.084	-7.501	6.3E-14	4.5E-12
ENSMUSG00000032855	Pkd1	2249.344	-0.776	0.103	-7.494	6.7E-14	4.8E-12
ENSMUSG00000074264	Amy1	3167.470	-0.623	0.083	-7.488	7.0E-14	5.0E-12
ENSMUSG00000022389	Tef	3104.204	-0.609	0.082	-7.473	7.8E-14	5.5E-12
ENSMUSG00000024654	Asrgl1	1961.980	-0.706	0.095	-7.471	7.9E-14	5.6E-12
ENSMUSG00000040681	Hmgn1	9684.380	-0.614	0.082	-7.472	7.9E-14	5.6E-12
ENSMUSG00000071653	1810009A15Rik	839.271	-0.948	0.127	-7.465	8.3E-14	5.8E-12
ENSMUSG00000046275	Trarg1	260.491	-1.503	0.202	-7.429	1.1E-13	7.6E-12
ENSMUSG00000057440	Mpp7	1046.319	-0.854	0.115	-7.405	1.3E-13	9.1E-12
ENSMUSG00000037593	Rskr	1582.466	-0.715	0.097	-7.355	1.9E-13	1.3E-11
ENSMUSG00000030265	Kras	3457.926	-0.608	0.083	-7.346	2.0E-13	1.4E-11
ENSMUSG00000021091	Serpina3n	163.888	2.124	0.290	7.322	2.4E-13	1.7E-11
ENSMUSG00000059434	Gckr	1147.368	-0.953	0.130	-7.317	2.5E-13	1.7E-11
ENSMUSG00000024424	Ttc39c	816.334	-0.898	0.123	-7.277	3.4E-13	2.3E-11
ENSMUSG00000038241	Cep250	2898.730	-0.627	0.086	-7.257	3.9E-13	2.7E-11
ENSMUSG00000028977	Casz1	3729.021	-0.657	0.091	-7.255	4.0E-13	2.7E-11
ENSMUSG00000019874	Fabp7	93.996	2.636	0.364	7.249	4.2E-13	2.8E-11

ENSMUSG00000118646		542.073	-1.099	0.152	-7.246	4.3E-13	2.8E-11
ENSMUSG00000066152	Slc31a2	1882.229	-0.710	0.098	-7.229	4.9E-13	3.2E-11
ENSMUSG00000017843	Ppp2r5c	4279.578	-0.607	0.084	-7.227	4.9E-13	3.2E-11
ENSMUSG00000023087	Noct	8860.832	0.507	0.071	7.168	7.6E-13	5.0E-11
ENSMUSG00000045672	Col27a1	260.330	-1.481	0.207	-7.168	7.6E-13	5.0E-11
ENSMUSG00000042282	Gucy2f	8760.039	-0.483	0.068	-7.156	8.3E-13	5.4E-11
ENSMUSG00000001103	Sebox	480.274	-1.148	0.161	-7.135	9.7E-13	6.3E-11
ENSMUSG00000022844	Pdia5	434.421	-1.292	0.181	-7.119	1.1E-12	7.0E-11
ENSMUSG00000034459	Ifit1	160.155	1.923	0.271	7.106	1.2E-12	7.7E-11
ENSMUSG00000024614	Tmx3	2299.036	0.636	0.090	7.103	1.2E-12	7.7E-11
ENSMUSG00000057421	Las1l	2232.900	-0.625	0.088	-7.103	1.2E-12	7.7E-11
ENSMUSG00000034311	Kif4	125.058	-2.557	0.361	-7.087	1.4E-12	8.7E-11
ENSMUSG00000051860	Samd7	3767.513	0.552	0.078	7.078	1.5E-12	9.2E-11
ENSMUSG00000026797	Stxbp1	10797.897	-0.468	0.066	-7.075	1.5E-12	9.4E-11
ENSMUSG00000070003	Ssbp4	842.672	-0.970	0.137	-7.066	1.6E-12	1.0E-10
ENSMUSG00000043557	Mdga1	484.228	-1.108	0.157	-7.061	1.7E-12	1.0E-10
ENSMUSG00000034274	Thoc5	1316.745	-0.731	0.104	-7.054	1.7E-12	1.1E-10
ENSMUSG00000048546	Tob2	2646.243	-0.705	0.100	-7.052	1.8E-12	1.1E-10
ENSMUSG00000005225	Plekha8	1895.846	-0.680	0.097	-7.046	1.8E-12	1.1E-10
ENSMUSG00000112972	Gm48417	464.842	-1.119	0.159	-7.046	1.8E-12	1.1E-10
ENSMUSG00000049969	Plekhf2	2017.460	-0.803	0.114	-7.038	1.9E-12	1.2E-10
ENSMUSG00000060802	B2m	533.754	1.048	0.149	7.032	2.0E-12	1.2E-10
ENSMUSG00000031824	6430548M08Rik	3739.879	-0.565	0.080	-7.028	2.1E-12	1.3E-10
ENSMUSG00000039714	Cplx3	3695.679	-0.562	0.080	-7.025	2.1E-12	1.3E-10
ENSMUSG00000019487	Trip10	355.924	-1.220	0.174	-7.021	2.2E-12	1.3E-10
ENSMUSG00000024743	Syt7	1076.170	-0.848	0.121	-7.017	2.3E-12	1.3E-10
ENSMUSG00000029055	Plch2	5143.998	-0.550	0.078	-7.008	2.4E-12	1.4E-10
ENSMUSG00000003810	Mast2	3195.146	-0.657	0.094	-7.006	2.5E-12	1.5E-10
ENSMUSG00000070327	Rnf213	427.259	1.137	0.162	6.998	2.6E-12	1.5E-10
ENSMUSG00000030657	Xylt1	1422.681	-0.779	0.111	-6.991	2.7E-12	1.6E-10
ENSMUSG00000016356	Col20a1	202.422	-1.739	0.249	-6.983	2.9E-12	1.7E-10
ENSMUSG00000041782	Lad1	84.299	2.717	0.389	6.980	3.0E-12	1.7E-10
ENSMUSG00000040860	Crocc	2852.694	-0.621	0.089	-6.976	3.0E-12	1.8E-10
ENSMUSG00000074794	Arrdc3	2303.648	-0.642	0.092	-6.961	3.4E-12	2.0E-10
ENSMUSG00000028125	Abca4	19831.888	-0.454	0.065	-6.946	3.8E-12	2.2E-10
ENSMUSG00000029208	Guf1	2216.539	0.611	0.088	6.946	3.8E-12	2.2E-10
ENSMUSG00000027634	Ndrg3	9090.912	-0.553	0.080	-6.926	4.3E-12	2.5E-10
ENSMUSG00000027367	Stard7	5904.226	-0.563	0.081	-6.910	4.8E-12	2.8E-10
ENSMUSG00000027309	Dnaaf9	1907.921	-0.685	0.099	-6.895	5.4E-12	3.1E-10
ENSMUSG00000037995	Igsf9	956.020	-0.889	0.129	-6.878	6.1E-12	3.4E-10
ENSMUSG00000038530	Rgs4	831.512	-0.878	0.128	-6.877	6.1E-12	3.4E-10
ENSMUSG00000027883	Gpsm2	4523.676	-0.540	0.079	-6.863	6.7E-12	3.8E-10
ENSMUSG00000013846	St3gal1	1577.139	-0.665	0.097	-6.857	7.0E-12	3.9E-10
ENSMUSG00000006649	Nphs1	66.667	-2.779	0.406	-6.839	8.0E-12	4.4E-10
ENSMUSG00000041609	Bicdl1	2620.480	-0.613	0.090	-6.835	8.2E-12	4.5E-10
ENSMUSG00000040296	Ddx58	261.716	1.393	0.204	6.830	8.5E-12	4.7E-10
ENSMUSG00000049811	Fam161a	5926.052	-0.512	0.075	-6.826	8.7E-12	4.8E-10
ENSMUSG00000042210	Abhd14a	570.648	-0.980	0.144	-6.826	8.7E-12	4.8E-10
ENSMUSG00000018752	Tnfsfm13	446.333	-1.122	0.164	-6.825	8.8E-12	4.8E-10
ENSMUSG00000111824	Gm47102	896.073	-0.802	0.118	-6.807	1.0E-11	5.4E-10
ENSMUSG00000074896	Ifit3	431.337	1.158	0.170	6.806	1.0E-11	5.4E-10
ENSMUSG00000095930	Nim1k	1805.340	-0.691	0.102	-6.801	1.0E-11	5.6E-10
ENSMUSG00000035314	Gdpd5	1128.201	-0.789	0.116	-6.774	1.3E-11	6.7E-10
ENSMUSG00000005649	Cabp5	1362.120	-0.706	0.104	-6.770	1.3E-11	6.9E-10
ENSMUSG00000032501	Trib1	645.250	0.942	0.139	6.764	1.3E-11	7.1E-10
ENSMUSG00000112349	Gm48132	1103.782	-0.743	0.110	-6.760	1.4E-11	7.3E-10

ENSMUSG00000022994	Adcy6	2207.743	-0.674	0.100	-6.758	1.4E-11	7.4E-10
ENSMUSG00000024112	Cacna1h	1328.526	-0.731	0.108	-6.754	1.4E-11	7.6E-10
ENSMUSG00000031558	Slit2	2217.405	-0.616	0.092	-6.696	2.1E-11	1.1E-09
ENSMUSG00000087141	Plcxd2	2622.070	-0.588	0.088	-6.690	2.2E-11	1.2E-09
ENSMUSG00000031028	Tub	5751.938	-0.514	0.077	-6.689	2.2E-11	1.2E-09
ENSMUSG00000046447	Camk2n1	2976.234	-0.546	0.082	-6.680	2.4E-11	1.2E-09
ENSMUSG00000038811	Gngt2	506.994	-1.044	0.156	-6.672	2.5E-11	1.3E-09
ENSMUSG00000050321	Neto1	485.247	-1.052	0.158	-6.659	2.8E-11	1.4E-09
ENSMUSG00000087569	Actb-ps1	33.701	-8.603	1.300	-6.615	3.7E-11	1.9E-09
ENSMUSG00000025927	Tfap2b	2429.483	-0.681	0.103	-6.606	3.9E-11	2.0E-09
ENSMUSG00000021928	Ebpl	1431.897	-0.705	0.107	-6.604	4.0E-11	2.0E-09
ENSMUSG00000074652	Myh7b	78.341	-2.432	0.369	-6.597	4.2E-11	2.1E-09
ENSMUSG00000049630	C1ql3	1020.803	0.829	0.126	6.562	5.3E-11	2.7E-09
ENSMUSG00000001642	Akr1b3	623.110	0.947	0.145	6.547	5.9E-11	3.0E-09
ENSMUSG00000075410	Prcd	1689.322	-0.777	0.119	-6.532	6.5E-11	3.3E-09
ENSMUSG00000109372	Gm19410	146.823	1.775	0.272	6.530	6.6E-11	3.3E-09
ENSMUSG00000021013	Ttc8	2096.232	-0.585	0.090	-6.527	6.7E-11	3.4E-09
ENSMUSG00000047034	Ankrd33	1319.162	-0.727	0.111	-6.525	6.8E-11	3.4E-09
ENSMUSG00000050132	Sarm1	1196.320	-0.822	0.126	-6.523	6.9E-11	3.4E-09
ENSMUSG00000035929	H2-Q4	158.938	1.767	0.271	6.516	7.2E-11	3.6E-09
ENSMUSG00000026475	Rgs16	401.355	-1.078	0.166	-6.503	7.8E-11	3.9E-09
ENSMUSG00000000308	Ckmt1	1194.308	-0.742	0.114	-6.502	7.9E-11	3.9E-09
ENSMUSG00000020718	Polg2	577.163	-1.002	0.154	-6.498	8.1E-11	4.0E-09
ENSMUSG00000027270	Lamp5	616.764	-0.905	0.139	-6.495	8.3E-11	4.1E-09
ENSMUSG00000041235	Chd7	5120.593	0.583	0.090	6.494	8.3E-11	4.1E-09
ENSMUSG00000011179	Odc1	2108.159	-0.676	0.104	-6.481	9.1E-11	4.4E-09
ENSMUSG00000048154	Kmt2d	6690.250	-0.449	0.069	-6.462	1.0E-10	5.0E-09
ENSMUSG00000034730	Adgrb1	679.046	-0.952	0.148	-6.431	1.3E-10	6.1E-09
ENSMUSG00000019850	Tnfaip3	5104.931	-0.579	0.090	-6.419	1.4E-10	6.6E-09
ENSMUSG00000031492	Chrnb3	1142.968	-0.796	0.124	-6.403	1.5E-10	7.3E-09
ENSMUSG00000000738	Spg7	1290.163	-0.662	0.104	-6.368	1.9E-10	9.1E-09
ENSMUSG00000075256	Cerkl	1243.274	0.803	0.127	6.338	2.3E-10	1.1E-08
ENSMUSG00000048416	Mlf1	204.847	1.438	0.227	6.322	2.6E-10	1.2E-08
ENSMUSG00000036061	Smug1	1953.650	-0.585	0.093	-6.317	2.7E-10	1.3E-08
ENSMUSG00000036995	Asap3	221.831	1.369	0.217	6.300	3.0E-10	1.4E-08
ENSMUSG00000015656	Hspa8	22848.271	0.448	0.071	6.297	3.0E-10	1.4E-08
ENSMUSG00000037936	Scarb1	1851.176	-0.631	0.101	-6.275	3.5E-10	1.6E-08
ENSMUSG00000018750	Zbtb4	3774.586	-0.514	0.082	-6.269	3.6E-10	1.7E-08
ENSMUSG00000038026	Kcnj9	1123.020	-0.701	0.112	-6.265	3.7E-10	1.7E-08
ENSMUSG00000010175	Prox1	3479.746	-0.588	0.094	-6.264	3.7E-10	1.7E-08
ENSMUSG00000059824	Dbp	1064.971	-0.715	0.114	-6.262	3.8E-10	1.8E-08
ENSMUSG00000041741	Pde3a	1436.998	-0.655	0.105	-6.258	3.9E-10	1.8E-08
ENSMUSG00000026193	Fn1	562.021	-0.976	0.156	-6.257	3.9E-10	1.8E-08
ENSMUSG00000035539	Ccdc180	74.974	-2.360	0.377	-6.251	4.1E-10	1.9E-08
ENSMUSG00000031600	Vps37a	5672.178	0.445	0.071	6.245	4.2E-10	1.9E-08
ENSMUSG00000028583	Pdpn	984.456	0.788	0.126	6.243	4.3E-10	2.0E-08
ENSMUSG00000023495	Pcbp4	3763.700	-0.510	0.082	-6.242	4.3E-10	2.0E-08
ENSMUSG00000028188	Spata1	1165.265	-0.786	0.126	-6.228	4.7E-10	2.1E-08
ENSMUSG00000039763	Dnajc28	822.985	-0.818	0.132	-6.216	5.1E-10	2.3E-08
ENSMUSG00000034295	Fhod3	1739.605	-0.586	0.094	-6.212	5.2E-10	2.4E-08
ENSMUSG00000116166	Gm49403	809.555	-0.999	0.161	-6.208	5.4E-10	2.4E-08
ENSMUSG00000041771	Slc24a4	840.865	-0.759	0.123	-6.188	6.1E-10	2.7E-08
ENSMUSG00000057286	St6galnac2	877.431	-0.749	0.121	-6.176	6.6E-10	2.9E-08
ENSMUSG00000020580	Rock2	3616.660	0.585	0.095	6.170	6.8E-10	3.0E-08
ENSMUSG00000040632	Nrl	14519.124	-1.193	0.193	-6.166	7.0E-10	3.1E-08
ENSMUSG00000048503	Tlcd5	4243.950	-0.523	0.085	-6.166	7.0E-10	3.1E-08

ENSMUSG00000063142	Kcnma1	5930.263	-0.484	0.079	-6.156	7.5E-10	3.3E-08
ENSMUSG00000042500	ago-04	781.400	0.779	0.127	6.155	7.5E-10	3.3E-08
ENSMUSG00000040084	Bub1b	906.559	0.734	0.119	6.154	7.6E-10	3.3E-08
ENSMUSG00000025650	Col7a1	366.260	-1.342	0.219	-6.122	9.3E-10	4.0E-08
ENSMUSG00000036054	Sugp2	1993.961	-0.604	0.099	-6.115	9.7E-10	4.2E-08
ENSMUSG00000017400	Stac2	819.689	-0.771	0.126	-6.100	1.1E-09	4.6E-08
ENSMUSG00000097886	Gsg1l2	186.346	-1.443	0.237	-6.094	1.1E-09	4.8E-08
ENSMUSG00000005198	Polr2a	5331.395	0.520	0.085	6.086	1.2E-09	5.0E-08
ENSMUSG00000021557	Agtpbp1	6062.330	0.479	0.079	6.080	1.2E-09	5.1E-08
ENSMUSG00000033849	B3galt2	3389.435	-0.504	0.083	-6.080	1.2E-09	5.1E-08
ENSMUSG00000104713	Gbp6	333.030	1.117	0.184	6.079	1.2E-09	5.2E-08
ENSMUSG00000079484	Phyhd1	230.464	-1.325	0.219	-6.057	1.4E-09	5.9E-08
ENSMUSG00000002055	Spag5	657.355	-1.038	0.172	-6.053	1.4E-09	6.0E-08
ENSMUSG00000022237	Ankrd33b	6699.864	-0.420	0.070	-6.042	1.5E-09	6.5E-08
ENSMUSG00000022037	Clu	6695.090	0.557	0.092	6.027	1.7E-09	7.1E-08
ENSMUSG00000037126	Psd	2225.224	-0.617	0.102	-6.025	1.7E-09	7.1E-08
ENSMUSG00000061411	Nol4l	1464.042	-0.682	0.113	-6.017	1.8E-09	7.5E-08
ENSMUSG00000050711	Scg2	7783.007	-0.451	0.075	-6.014	1.8E-09	7.6E-08
ENSMUSG00000079523	Tmsb10	1478.158	-0.641	0.107	-6.010	1.9E-09	7.8E-08
ENSMUSG00000046879	Irgm1	182.588	1.500	0.250	6.009	1.9E-09	7.8E-08
ENSMUSG00000020937	Plcd3	585.599	-0.912	0.152	-6.002	1.9E-09	8.1E-08
ENSMUSG00000040125	Gpr26	457.292	-1.008	0.168	-6.000	2.0E-09	8.2E-08
ENSMUSG00000039910	Cited2	1637.789	0.573	0.096	5.986	2.1E-09	8.9E-08
ENSMUSG00000031637	Lrp2bp	325.487	-1.110	0.186	-5.974	2.3E-09	9.6E-08
ENSMUSG00000039114	Nrn1	733.865	-0.773	0.130	-5.965	2.4E-09	1.0E-07
ENSMUSG00000021009	Ptpn21	1475.285	-0.592	0.100	-5.944	2.8E-09	1.1E-07
ENSMUSG00000072624	Gm5460	169.355	-1.576	0.266	-5.927	3.1E-09	1.3E-07
ENSMUSG00000050786	Ccdc126	3170.629	-0.611	0.103	-5.913	3.4E-09	1.4E-07
ENSMUSG00000060671	Atp8b2	671.410	0.934	0.158	5.911	3.4E-09	1.4E-07
ENSMUSG00000020069	Hnrnph3	2500.733	-0.528	0.090	-5.902	3.6E-09	1.4E-07
ENSMUSG00000074365	Crxos	278.407	-1.173	0.199	-5.888	3.9E-09	1.6E-07
ENSMUSG00000033419	Snap91	5170.378	-0.433	0.074	-5.879	4.1E-09	1.7E-07
ENSMUSG00000001120	Pcbp3	3830.044	-0.475	0.081	-5.878	4.2E-09	1.7E-07
ENSMUSG00000042671	Rgs8	2146.439	-0.523	0.089	-5.871	4.3E-09	1.7E-07
ENSMUSG00000056494	Cngb3	519.604	-0.887	0.151	-5.865	4.5E-09	1.8E-07
ENSMUSG00000025496	Drd4	833.250	-0.735	0.125	-5.865	4.5E-09	1.8E-07
ENSMUSG00000034248	Slc25a37	1426.253	0.593	0.101	5.859	4.6E-09	1.8E-07
ENSMUSG00000027070	Lrp2	574.930	0.851	0.145	5.854	4.8E-09	1.9E-07
ENSMUSG00000030787	Lyve1	166.006	-1.468	0.251	-5.852	4.8E-09	1.9E-07
ENSMUSG00000037225	Fgf2	1390.184	1.713	0.293	5.846	5.1E-09	2.0E-07
ENSMUSG00000014782	Plekhg4	134.644	-1.979	0.339	-5.843	5.1E-09	2.0E-07
ENSMUSG00000053420	Gm4792	740.364	-0.765	0.131	-5.833	5.4E-09	2.1E-07
ENSMUSG00000046049	Rp1l1	8965.095	-0.415	0.071	-5.826	5.7E-09	2.2E-07
ENSMUSG00000038828	Tmem214	1106.196	-0.679	0.117	-5.819	5.9E-09	2.3E-07
ENSMUSG00000035126	Dnai4	1647.635	-0.720	0.124	-5.814	6.1E-09	2.4E-07
ENSMUSG00000070880	Gad1	2965.099	-0.484	0.083	-5.805	6.4E-09	2.5E-07
ENSMUSG00000113393	Gm48415	1349.242	-0.601	0.104	-5.802	6.5E-09	2.5E-07
ENSMUSG00000028842	ago-03	2692.661	-0.534	0.092	-5.793	6.9E-09	2.7E-07
ENSMUSG00000007827	Ankrd26	1078.361	0.711	0.123	5.785	7.3E-09	2.8E-07
ENSMUSG00000038079	Tmem237	1536.581	0.625	0.108	5.781	7.4E-09	2.8E-07
ENSMUSG00000071648	Rom1	13663.632	-1.089	0.188	-5.781	7.4E-09	2.8E-07
ENSMUSG00000022594	Lynx1	1655.900	-0.549	0.095	-5.764	8.2E-09	3.1E-07
ENSMUSG00000087298	Gm9392	74.837	-2.371	0.413	-5.749	9.0E-09	3.4E-07
ENSMUSG00000054752	Fsd1l	5064.621	-0.509	0.089	-5.730	1.0E-08	3.8E-07
ENSMUSG00000049550	Clip1	2051.676	-0.571	0.100	-5.728	1.0E-08	3.8E-07
ENSMUSG00000020089	Ppa1	1428.842	-0.624	0.109	-5.725	1.0E-08	3.9E-07

ENSMUSG00000025609	Mkln1	3583.279	0.547	0.096	5.723	1.0E-08	3.9E-07
ENSMUSG00000069170	Adgrv1	4354.758	-0.462	0.081	-5.715	1.1E-08	4.1E-07
ENSMUSG00000053773	Rdh8	892.403	-0.719	0.126	-5.713	1.1E-08	4.1E-07
ENSMUSG00000038312	Edem2	556.283	-0.826	0.145	-5.706	1.2E-08	4.3E-07
ENSMUSG00000036304	Zdhhc23	184.339	1.495	0.263	5.694	1.2E-08	4.6E-07
ENSMUSG00000027284	Cdan1	1069.728	-0.674	0.118	-5.690	1.3E-08	4.7E-07
ENSMUSG00000034786	Gpsm3	212.140	-1.262	0.222	-5.688	1.3E-08	4.8E-07
ENSMUSG00000003038	Hmgn2	5137.224	-0.511	0.090	-5.687	1.3E-08	4.8E-07
ENSMUSG00000028630	Dyrk2	3981.801	-0.440	0.078	-5.673	1.4E-08	5.2E-07
ENSMUSG00000018378	Cuedc1	1762.492	-0.535	0.094	-5.671	1.4E-08	5.2E-07
ENSMUSG00000054074	Skida1	1980.340	-0.526	0.093	-5.669	1.4E-08	5.3E-07
ENSMUSG00000023224	Serping1	181.044	1.434	0.253	5.667	1.5E-08	5.3E-07
ENSMUSG00000038507	Parp12	333.985	1.054	0.186	5.665	1.5E-08	5.4E-07
ENSMUSG00000079055	Slc8a3	1086.724	-0.631	0.111	-5.665	1.5E-08	5.4E-07
ENSMUSG00000036854	Hspb6	858.144	-0.752	0.133	-5.658	1.5E-08	5.6E-07
ENSMUSG00000082361	Btc	495.188	0.863	0.153	5.655	1.6E-08	5.7E-07
ENSMUSG00000022462	Slc38a2	2641.805	0.525	0.093	5.648	1.6E-08	5.9E-07
ENSMUSG00000042826	Fgf11	707.616	-0.754	0.134	-5.630	1.8E-08	6.5E-07
ENSMUSG00000015217	Hmgb3	450.223	0.924	0.164	5.623	1.9E-08	6.8E-07
ENSMUSG00000071637	Cebpd	85.639	2.057	0.366	5.621	1.9E-08	6.8E-07
ENSMUSG00000001027	Scn4a	210.022	-1.275	0.227	-5.617	1.9E-08	6.9E-07
ENSMUSG00000025995	Wdr75	1047.519	0.640	0.114	5.607	2.1E-08	7.4E-07
ENSMUSG00000028766	Alpl	2872.154	-0.500	0.089	-5.606	2.1E-08	7.4E-07
ENSMUSG00000038390	Gpr162	1354.355	0.591	0.105	5.606	2.1E-08	7.4E-07
ENSMUSG00000029544	Cabp1	1147.889	-0.625	0.112	-5.588	2.3E-08	8.1E-07
ENSMUSG00000031441	Atp11a	6311.126	-0.463	0.083	-5.585	2.3E-08	8.3E-07
ENSMUSG00000059810	Rgs3	517.908	-0.828	0.148	-5.580	2.4E-08	8.5E-07
ENSMUSG00000013593	Ndufs2	2231.647	-0.519	0.093	-5.576	2.5E-08	8.6E-07
ENSMUSG00000020848	Doc2b	1952.073	0.506	0.091	5.573	2.5E-08	8.8E-07
ENSMUSG00000028906	Epb41	13774.341	-0.362	0.065	-5.570	2.5E-08	8.9E-07
ENSMUSG00000026473	Glul	35287.904	-0.369	0.066	-5.564	2.6E-08	9.2E-07
ENSMUSG00000037822	Smim14	2055.583	0.509	0.092	5.563	2.7E-08	9.3E-07
ENSMUSG00000031099	Smarca1	764.734	0.733	0.132	5.554	2.8E-08	9.7E-07
ENSMUSG00000068154	Insm1	492.481	0.855	0.154	5.546	2.9E-08	1.0E-06
ENSMUSG00000050229	Pigm	1156.969	-0.623	0.113	-5.538	3.1E-08	1.1E-06
ENSMUSG00000048376	F2r	239.168	-1.169	0.211	-5.535	3.1E-08	1.1E-06
ENSMUSG00000005204	Senp3	916.006	-0.667	0.121	-5.529	3.2E-08	1.1E-06
ENSMUSG00000028268	Gbp3	137.458	1.595	0.289	5.528	3.2E-08	1.1E-06
ENSMUSG00000063229	Ldha	11566.394	-0.431	0.078	-5.523	3.3E-08	1.1E-06
ENSMUSG00000028101	Pias3	1255.974	-0.633	0.115	-5.513	3.5E-08	1.2E-06
ENSMUSG00000053702	Nebl	1615.226	-0.574	0.104	-5.512	3.5E-08	1.2E-06
ENSMUSG00000039789	Zfp597	1121.462	-0.689	0.125	-5.508	3.6E-08	1.2E-06
ENSMUSG00000015214	Mtmr1	2228.099	0.482	0.087	5.508	3.6E-08	1.2E-06
ENSMUSG00000022040	Ephx2	620.389	0.768	0.140	5.505	3.7E-08	1.3E-06
ENSMUSG00000052305	Hbb-bs	267.342	-1.134	0.206	-5.505	3.7E-08	1.3E-06
ENSMUSG00000004319	Clcn3	8743.312	0.392	0.071	5.492	4.0E-08	1.3E-06
ENSMUSG00000037400	Atp11b	8008.999	-0.372	0.068	-5.471	4.5E-08	1.5E-06
ENSMUSG00000047747	Rnf150	3010.734	-0.453	0.083	-5.472	4.5E-08	1.5E-06
ENSMUSG00000026930	Gpsm1	1708.848	-0.542	0.099	-5.470	4.5E-08	1.5E-06
ENSMUSG00000043467	Zbtb37	2568.695	-0.574	0.105	-5.464	4.7E-08	1.6E-06
ENSMUSG00000021508	Cxcl14	310.123	-1.048	0.192	-5.461	4.7E-08	1.6E-06
ENSMUSG00000049775	Tmsb4x	1359.378	0.593	0.109	5.458	4.8E-08	1.6E-06
ENSMUSG00000081683	Fzd10	66.852	-2.304	0.423	-5.446	5.2E-08	1.7E-06
ENSMUSG00000035024	Ncapd3	1742.492	-0.513	0.094	-5.442	5.3E-08	1.7E-06
ENSMUSG00000036093	Arl5a	2579.340	0.464	0.085	5.440	5.3E-08	1.8E-06
ENSMUSG00000029608	Rph3a	1879.725	-0.516	0.095	-5.438	5.4E-08	1.8E-06

ENSMUSG00000112808	Gm4739	192.310	-1.444	0.266	-5.437	5.4E-08	1.8E-06
ENSMUSG00000042251	Pm20d1	99.225	-1.755	0.323	-5.436	5.5E-08	1.8E-06
ENSMUSG00000055567	Unc80	7540.572	0.412	0.076	5.434	5.5E-08	1.8E-06
ENSMUSG00000028255	Clca1	133.653	-1.495	0.276	-5.425	5.8E-08	1.9E-06
ENSMUSG00000020486	Septin4	1717.609	-0.537	0.099	-5.419	6.0E-08	2.0E-06
ENSMUSG00000030878	Cdr2	3030.342	-0.525	0.097	-5.416	6.1E-08	2.0E-06
ENSMUSG00000114123	Gm48416	1408.210	-0.550	0.102	-5.412	6.2E-08	2.0E-06
ENSMUSG00000079108	Srp54c	3420.438	-0.466	0.086	-5.409	6.4E-08	2.1E-06
ENSMUSG00000000305	Cdh4	364.638	-0.976	0.181	-5.395	6.9E-08	2.2E-06
ENSMUSG00000026141	Col19a1	206.051	-1.216	0.226	-5.392	7.0E-08	2.2E-06
ENSMUSG00000021730	Hcn1	6680.601	-0.436	0.081	-5.388	7.1E-08	2.3E-06
ENSMUSG00000026014	Raph1	1043.610	-0.613	0.114	-5.386	7.2E-08	2.3E-06
ENSMUSG00000021848	Otx2	5094.120	0.393	0.073	5.381	7.4E-08	2.4E-06
ENSMUSG00000037957	Wdr20	1080.077	-0.673	0.125	-5.375	7.7E-08	2.5E-06
ENSMUSG00000108218	Olfr1372-ps1	570.148	-0.816	0.152	-5.373	7.8E-08	2.5E-06
ENSMUSG00000052684	Jun	553.848	0.813	0.151	5.371	7.8E-08	2.5E-06
ENSMUSG00000041261	Car8	1431.293	-0.535	0.100	-5.367	8.0E-08	2.5E-06
ENSMUSG00000086583	Gm15500	3791.157	0.492	0.092	5.364	8.1E-08	2.6E-06
ENSMUSG00000020661	Dnmt3a	3436.600	-0.434	0.081	-5.357	8.5E-08	2.7E-06
ENSMUSG00000032883	Acsl3	7466.735	-0.408	0.077	-5.324	1.0E-07	3.2E-06
ENSMUSG00000038056	Kmt2c	6668.609	-0.401	0.075	-5.319	1.0E-07	3.3E-06
ENSMUSG00000026933	Camsap1	5307.410	-0.470	0.088	-5.313	1.1E-07	3.4E-06
ENSMUSG00000075706	Gpx4	3209.364	-0.515	0.097	-5.311	1.1E-07	3.4E-06
ENSMUSG00000024993	Dennd10	1754.967	-0.594	0.112	-5.302	1.1E-07	3.6E-06
ENSMUSG00000028456	Unc13b	11213.717	-0.393	0.074	-5.297	1.2E-07	3.7E-06
ENSMUSG00000032384	Csnk1g1	2273.251	-0.466	0.088	-5.290	1.2E-07	3.8E-06
ENSMUSG00000021775	Nr1d2	5240.797	-0.394	0.075	-5.282	1.3E-07	4.0E-06
ENSMUSG00000026667	Uhmk1	7960.796	-0.362	0.069	-5.255	1.5E-07	4.6E-06
ENSMUSG00000001419	Mef2d	5725.668	-0.391	0.075	-5.225	1.7E-07	5.4E-06
ENSMUSG00000037112	Sik2	896.999	-0.674	0.129	-5.224	1.8E-07	5.4E-06
ENSMUSG00000111485	Gm48443	844.795	-0.640	0.122	-5.224	1.8E-07	5.4E-06
ENSMUSG00000032332	Col12a1	176.574	-1.255	0.241	-5.216	1.8E-07	5.6E-06
ENSMUSG00000024079	Eif2ak2	238.674	1.103	0.212	5.215	1.8E-07	5.6E-06
ENSMUSG00000025035	Arl3	3093.248	-0.440	0.085	-5.208	1.9E-07	5.9E-06
ENSMUSG00000014786	Slc9a5	557.570	-0.785	0.151	-5.204	2.0E-07	6.0E-06
ENSMUSG00000039059	Hrh3	142.386	-1.410	0.271	-5.199	2.0E-07	6.1E-06
ENSMUSG00000025351	Cd63	795.087	0.692	0.133	5.198	2.0E-07	6.1E-06
ENSMUSG00000117877	Gm8184	212.857	-1.214	0.234	-5.197	2.0E-07	6.2E-06
ENSMUSG00000048458	Inka2	1303.458	-0.590	0.114	-5.195	2.0E-07	6.2E-06
ENSMUSG00000105207	Gm42927	1906.185	-0.484	0.093	-5.194	2.1E-07	6.2E-06
ENSMUSG00000114367	Gm48216	639.102	-0.715	0.138	-5.178	2.2E-07	6.8E-06
ENSMUSG00000087235	Gm4750	61.956	-2.160	0.417	-5.177	2.3E-07	6.8E-06
ENSMUSG00000025787	Tgm4	236.618	-1.127	0.218	-5.171	2.3E-07	7.0E-06
ENSMUSG00000041144	Dnah7b	1744.375	0.493	0.096	5.158	2.5E-07	7.5E-06
ENSMUSG00000113395	Gm48365	304.630	-0.957	0.186	-5.157	2.5E-07	7.5E-06
ENSMUSG00000038481	Cdk19	4487.424	0.395	0.077	5.153	2.6E-07	7.7E-06
ENSMUSG00000034422	Parp14	232.694	1.138	0.221	5.151	2.6E-07	7.7E-06
ENSMUSG00000090115	Usp49	884.349	-0.775	0.150	-5.151	2.6E-07	7.7E-06
ENSMUSG00000047496	Rnf152	1364.570	-0.533	0.103	-5.149	2.6E-07	7.8E-06
ENSMUSG00000008206	Cers4	4687.091	-0.409	0.079	-5.143	2.7E-07	8.0E-06
ENSMUSG00000024589	Nedd4l	1648.576	-0.502	0.098	-5.144	2.7E-07	8.0E-06
ENSMUSG00000032586	Traip	133.466	-1.449	0.282	-5.143	2.7E-07	8.0E-06
ENSMUSG00000020627	Klhl29	741.121	0.763	0.148	5.142	2.7E-07	8.0E-06
ENSMUSG00000043384	Gprasp1	10523.429	0.342	0.067	5.141	2.7E-07	8.0E-06
ENSMUSG00000040111	Gramd1b	9360.560	-0.371	0.072	-5.132	2.9E-07	8.4E-06
ENSMUSG00000039047	Pigk	1006.656	0.633	0.124	5.126	3.0E-07	8.6E-06

ENSMUSG00000053907	Mat2a	9354.618	-0.371	0.072	-5.126	3.0E-07	8.6E-06
ENSMUSG00000025921	Rdh10	883.706	0.611	0.120	5.113	3.2E-07	9.3E-06
ENSMUSG00000037266	Rsrp1	10054.138	-0.339	0.066	-5.112	3.2E-07	9.3E-06
ENSMUSG00000021803	Cdhr1	15201.233	-0.369	0.072	-5.104	3.3E-07	9.7E-06
ENSMUSG00000045776	Lrtm1	6882.041	-0.374	0.074	-5.084	3.7E-07	1.1E-05
ENSMUSG00000063077	Kif1b	23753.567	-0.344	0.068	-5.066	4.1E-07	1.2E-05
ENSMUSG00000037872	Ackr1	539.356	-0.737	0.146	-5.063	4.1E-07	1.2E-05
ENSMUSG00000051111	Sv2c	517.636	-0.786	0.155	-5.063	4.1E-07	1.2E-05
ENSMUSG00000070730	Rmdn3	1273.717	-0.624	0.123	-5.056	4.3E-07	1.2E-05
ENSMUSG00000027332	Ivd	1428.676	-0.518	0.102	-5.054	4.3E-07	1.2E-05
ENSMUSG00000032846	Zswim6	778.484	-0.651	0.129	-5.043	4.6E-07	1.3E-05
ENSMUSG00000023991	Foxp4	385.284	0.896	0.178	5.041	4.6E-07	1.3E-05
ENSMUSG00000073411	H2-D1	533.196	0.741	0.147	5.039	4.7E-07	1.3E-05
ENSMUSG00000003949	Hlf	3287.492	-0.411	0.082	-5.036	4.8E-07	1.4E-05
ENSMUSG00000036197	Gxylt1	1012.710	-0.580	0.116	-5.021	5.1E-07	1.5E-05
ENSMUSG00000037336	Mfsd2b	176.005	-1.204	0.240	-5.020	5.2E-07	1.5E-05
ENSMUSG00000059146	Ntrk3	664.793	-0.758	0.151	-5.014	5.3E-07	1.5E-05
ENSMUSG00000020279	Il9r	22.802	-4.268	0.853	-5.005	5.6E-07	1.6E-05
ENSMUSG00000025085	Ablim1	5078.560	-0.396	0.079	-5.003	5.6E-07	1.6E-05
ENSMUSG00000036902	Neto2	624.125	-0.678	0.135	-5.003	5.6E-07	1.6E-05
ENSMUSG00000034684	Sema3f	1347.528	-0.571	0.114	-4.997	5.8E-07	1.6E-05
ENSMUSG00000113795	Gm48119	364.711	-0.860	0.172	-4.995	5.9E-07	1.6E-05
ENSMUSG00000026074	Map4k4	1610.180	0.497	0.100	4.987	6.1E-07	1.7E-05
ENSMUSG00000021750	Fam107a	147.799	-1.347	0.270	-4.986	6.2E-07	1.7E-05
ENSMUSG00000051000	Fam160a1	1032.868	-0.555	0.112	-4.976	6.5E-07	1.8E-05
ENSMUSG00000030905	Crym	543.507	0.756	0.152	4.974	6.6E-07	1.8E-05
ENSMUSG00000030310	Slc6a1	3469.246	-0.407	0.082	-4.966	6.8E-07	1.9E-05
ENSMUSG00000052085	Dock8	105.249	-1.636	0.330	-4.964	6.9E-07	1.9E-05
ENSMUSG00000072966	Gprasp2	2110.623	0.450	0.091	4.962	7.0E-07	1.9E-05
ENSMUSG00000025190	Got1	4064.174	0.420	0.085	4.961	7.0E-07	1.9E-05
ENSMUSG00000021270	Hsp90aa1	45953.606	-0.400	0.081	-4.961	7.0E-07	1.9E-05
ENSMUSG00000019232	Etnppl	308.557	-1.002	0.202	-4.959	7.1E-07	1.9E-05
ENSMUSG00000047766	Lrrc49	1713.186	0.492	0.099	4.954	7.3E-07	2.0E-05
ENSMUSG00000032902	Slc16a1	4090.906	-0.411	0.083	-4.953	7.3E-07	2.0E-05
ENSMUSG00000024431	Nr3c1	2688.458	0.423	0.086	4.948	7.5E-07	2.1E-05
ENSMUSG00000032292	Nr2e3	7328.910	-0.994	0.201	-4.941	7.8E-07	2.1E-05
ENSMUSG00000095595	Fam177a	1648.519	-0.480	0.097	-4.940	7.8E-07	2.1E-05
ENSMUSG00000022564	Grina	4358.786	-0.376	0.076	-4.939	7.9E-07	2.1E-05
ENSMUSG00000025268	Maged2	709.926	0.645	0.131	4.935	8.0E-07	2.2E-05
ENSMUSG00000038642	Ctss	295.633	0.948	0.192	4.934	8.0E-07	2.2E-05
ENSMUSG00000025417	Pip4k2c	2135.914	-0.443	0.090	-4.933	8.1E-07	2.2E-05
ENSMUSG00000115279	Gm49273	460.589	-0.866	0.176	-4.930	8.2E-07	2.2E-05
ENSMUSG00000026669	Mcm10	85.265	-1.718	0.349	-4.928	8.3E-07	2.2E-05
ENSMUSG00000031530	Dusp4	406.494	-0.845	0.171	-4.927	8.3E-07	2.2E-05
ENSMUSG00000032495	Lrrc2	950.604	0.712	0.145	4.924	8.5E-07	2.3E-05
ENSMUSG00000040722	Scamp5	4481.654	-0.386	0.079	-4.922	8.6E-07	2.3E-05
ENSMUSG00000041794	Myrip	990.438	-0.570	0.116	-4.914	8.9E-07	2.4E-05
ENSMUSG00000050708	Ftl1	2343.647	0.457	0.093	4.903	9.4E-07	2.5E-05
ENSMUSG00000035283	Adrb1	178.313	-1.220	0.249	-4.901	9.5E-07	2.5E-05
ENSMUSG00000054604	Cggbp1	2512.489	0.446	0.091	4.895	9.8E-07	2.6E-05
ENSMUSG00000074968	Ano3	398.933	-0.803	0.164	-4.891	1.0E-06	2.7E-05
ENSMUSG00000053113	Socs3	67.281	2.082	0.426	4.889	1.0E-06	2.7E-05
ENSMUSG00000002249	Tead3	392.660	-0.815	0.167	-4.881	1.1E-06	2.8E-05
ENSMUSG00000029651	Mtus2	1024.140	-0.573	0.117	-4.880	1.1E-06	2.8E-05
ENSMUSG00000048978	Nrsn1	1267.412	-0.507	0.104	-4.878	1.1E-06	2.8E-05
ENSMUSG00000040896	Kcnd3	1367.997	-0.510	0.105	-4.876	1.1E-06	2.9E-05

ENSMUSG00000009828	Cilk1	1418.114	-0.490	0.101	-4.872	1.1E-06	2.9E-05
ENSMUSG00000040761	Spen	2129.239	-0.531	0.109	-4.870	1.1E-06	2.9E-05
ENSMUSG00000027981	Rnpc3	4303.593	-0.373	0.077	-4.869	1.1E-06	2.9E-05
ENSMUSG00000097328	Tnfsf12	1010.670	-0.587	0.121	-4.869	1.1E-06	2.9E-05
ENSMUSG00000018574	Acadvl	1246.329	-0.536	0.110	-4.865	1.1E-06	3.0E-05
ENSMUSG00000000617	Grm6	1579.838	-0.476	0.098	-4.858	1.2E-06	3.1E-05
ENSMUSG00000007570	Fance	393.124	-0.858	0.177	-4.856	1.2E-06	3.1E-05
ENSMUSG00000046593	Tmem215	862.279	-0.592	0.122	-4.851	1.2E-06	3.2E-05
ENSMUSG00000030064	Frmd4b	4139.198	-0.371	0.076	-4.850	1.2E-06	3.2E-05
ENSMUSG00000031586	Rbpms	264.642	-1.066	0.220	-4.842	1.3E-06	3.3E-05
ENSMUSG00000035403	Crb2	1218.658	-0.551	0.114	-4.834	1.3E-06	3.5E-05
ENSMUSG00000033080	Vsx1	415.112	-0.788	0.163	-4.832	1.4E-06	3.5E-05
ENSMUSG00000019889	Ptprk	753.255	-0.709	0.147	-4.830	1.4E-06	3.5E-05
ENSMUSG00000042258	Isl1	1473.489	-0.501	0.104	-4.827	1.4E-06	3.6E-05
ENSMUSG00000060371	Caln1	579.546	-0.676	0.140	-4.823	1.4E-06	3.6E-05
ENSMUSG00000023927	Satb1	567.955	0.720	0.149	4.821	1.4E-06	3.7E-05
ENSMUSG00000022723	Crybg3	1274.373	0.542	0.113	4.812	1.5E-06	3.8E-05
ENSMUSG00000064179	Tnnt1	218.616	-1.098	0.228	-4.811	1.5E-06	3.8E-05
ENSMUSG00000045817	Zfp36l2	1944.092	-0.449	0.094	-4.794	1.6E-06	4.2E-05
ENSMUSG00000037627	Rgs22	131.114	1.449	0.302	4.793	1.6E-06	4.2E-05
ENSMUSG00000029713	Gnb2	701.062	0.625	0.131	4.790	1.7E-06	4.2E-05
ENSMUSG00000026028	Trak2	5958.343	0.342	0.071	4.784	1.7E-06	4.3E-05
ENSMUSG00000036834	Plch1	2621.891	-0.399	0.084	-4.770	1.8E-06	4.7E-05
ENSMUSG00000028909	Ptpru	451.774	-0.841	0.176	-4.768	1.9E-06	4.7E-05
ENSMUSG00000025092	Hspa12a	3959.372	-0.385	0.081	-4.763	1.9E-06	4.8E-05
ENSMUSG00000026104	Stat1	1080.073	0.539	0.113	4.763	1.9E-06	4.8E-05
ENSMUSG00000030513	Pcsk6	251.659	-1.111	0.234	-4.751	2.0E-06	5.1E-05
ENSMUSG00000057897	Camk2b	2291.999	0.433	0.091	4.751	2.0E-06	5.1E-05
ENSMUSG00000054263	Lifr	2139.700	0.515	0.108	4.750	2.0E-06	5.1E-05
ENSMUSG00000020021	Fgd6	525.006	-0.785	0.166	-4.744	2.1E-06	5.2E-05
ENSMUSG00000022935	Grik1	1981.078	-0.434	0.091	-4.744	2.1E-06	5.2E-05
ENSMUSG00000020053	Igf1	845.130	-0.623	0.131	-4.741	2.1E-06	5.3E-05
ENSMUSG00000019951	Uhrf1bp1l	976.814	0.542	0.114	4.740	2.1E-06	5.3E-05
ENSMUSG00000020523	Fam114a2	1724.778	0.449	0.095	4.740	2.1E-06	5.3E-05
ENSMUSG00000030527	Crtc3	645.020	-0.685	0.145	-4.738	2.2E-06	5.3E-05
ENSMUSG00000019139	Isyna1	151.012	-1.234	0.261	-4.733	2.2E-06	5.5E-05
ENSMUSG00000020893	Per1	732.896	-0.614	0.130	-4.732	2.2E-06	5.5E-05
ENSMUSG00000062519	Zfp398	1522.000	-0.480	0.102	-4.727	2.3E-06	5.6E-05
ENSMUSG00000048540	Nhlh2	760.962	-0.592	0.125	-4.727	2.3E-06	5.6E-05
ENSMUSG00000087370	Tmem170b	2212.342	-0.414	0.087	-4.726	2.3E-06	5.6E-05
ENSMUSG00000029030	Tprgl	1167.801	0.522	0.110	4.723	2.3E-06	5.7E-05
ENSMUSG00000037921	Ddx60	147.771	1.288	0.273	4.722	2.3E-06	5.7E-05
ENSMUSG00000018593	Sparc	2309.673	0.453	0.096	4.718	2.4E-06	5.8E-05
ENSMUSG00000036192	Rorb	6634.648	-0.387	0.082	-4.707	2.5E-06	6.2E-05
ENSMUSG00000001175	Calm1	18475.185	-0.323	0.069	-4.701	2.6E-06	6.3E-05
ENSMUSG00000021196	Pfkp	14013.913	-0.337	0.072	-4.696	2.7E-06	6.5E-05
ENSMUSG00000026114	Cnga3	125.262	-1.503	0.320	-4.692	2.7E-06	6.6E-05
ENSMUSG00000043004	Gng2	1363.279	-0.478	0.102	-4.689	2.7E-06	6.7E-05
ENSMUSG00000048015	Neurod4	4391.491	-0.388	0.083	-4.684	2.8E-06	6.8E-05
ENSMUSG00000000276	Dgke	7176.606	-0.335	0.072	-4.664	3.1E-06	7.5E-05
ENSMUSG00000044033	Ccdc141	682.574	0.642	0.138	4.655	3.2E-06	7.8E-05
ENSMUSG00000020577	Tspan13	1648.027	0.454	0.098	4.648	3.3E-06	8.1E-05
ENSMUSG00000029298	Gbp9	142.845	1.257	0.271	4.646	3.4E-06	8.2E-05
ENSMUSG00000023944	Hsp90ab1	7232.171	0.370	0.080	4.643	3.4E-06	8.3E-05
ENSMUSG00000033460	Armcx1	1283.706	0.483	0.104	4.640	3.5E-06	8.4E-05
ENSMUSG00000114004	Gm48552	522.870	-0.775	0.167	-4.639	3.5E-06	8.4E-05

ENSMUSG00000094472	Gm21897	635.237	-0.657	0.142	-4.630	3.7E-06	8.7E-05
ENSMUSG00000063450	Syne2	4847.273	-0.384	0.083	-4.630	3.7E-06	8.7E-05
ENSMUSG00000030321	Efcab12	144.653	1.244	0.269	4.628	3.7E-06	8.8E-05
ENSMUSG00000034796	Cpne7	238.632	-0.991	0.214	-4.627	3.7E-06	8.8E-05
ENSMUSG00000020431	Adcy1	7371.493	-0.332	0.072	-4.624	3.8E-06	8.9E-05
ENSMUSG00000038319	Kcnh2	568.882	-0.705	0.153	-4.622	3.8E-06	9.0E-05
ENSMUSG00000045349	Sh2d5	389.649	-0.785	0.170	-4.622	3.8E-06	9.0E-05
ENSMUSG00000032557	Uba5	1051.327	0.515	0.111	4.619	3.9E-06	9.1E-05
ENSMUSG00000042650	Alkbh5	2269.045	-0.402	0.087	-4.616	3.9E-06	9.2E-05
ENSMUSG00000022146	Osmr	331.533	1.016	0.220	4.613	4.0E-06	9.3E-05
ENSMUSG00000016918	Sulf1	1712.774	0.516	0.112	4.607	4.1E-06	9.5E-05
ENSMUSG00000020108	Ddit4	801.877	-0.621	0.135	-4.608	4.1E-06	9.5E-05
ENSMUSG00000029166	Mapre3	2995.273	-0.436	0.095	-4.607	4.1E-06	9.5E-05
ENSMUSG00000047547	Cltb	1912.723	0.438	0.095	4.607	4.1E-06	9.5E-05
ENSMUSG00000017390	Aldoc	4825.474	-0.345	0.075	-4.606	4.1E-06	9.6E-05
ENSMUSG00000042834	Nrep	4234.150	0.373	0.081	4.606	4.1E-06	9.6E-05
ENSMUSG00000030921	Trim30a	49.349	2.135	0.464	4.603	4.2E-06	9.7E-05
ENSMUSG00000020912	Krt12	26.926	-3.639	0.791	-4.598	4.3E-06	9.9E-05
ENSMUSG00000020640	Itsn2	3904.058	-0.391	0.085	-4.597	4.3E-06	9.9E-05
ENSMUSG00000025323	Sp4	3797.100	-0.353	0.077	-4.595	4.3E-06	1.0E-04
ENSMUSG00000035615	Frmpd1	3104.369	-0.420	0.091	-4.593	4.4E-06	1.0E-04
ENSMUSG00000049092	Gpr137c	1429.518	0.460	0.100	4.584	4.6E-06	1.1E-04
ENSMUSG00000052534	Pbx1	3588.242	-0.371	0.081	-4.581	4.6E-06	1.1E-04
ENSMUSG00000024647	Cbln2	439.903	-0.766	0.167	-4.580	4.6E-06	1.1E-04
ENSMUSG00000085328	Gm17131	618.185	-0.648	0.142	-4.576	4.7E-06	1.1E-04
ENSMUSG00000067369	Trmt2b	712.905	0.603	0.132	4.568	4.9E-06	1.1E-04
ENSMUSG00000040724	Kcna2	1862.670	-0.482	0.106	-4.565	5.0E-06	1.1E-04
ENSMUSG00000020262	Adarb1	2681.503	-0.434	0.095	-4.560	5.1E-06	1.2E-04
ENSMUSG00000010064	Slc38a3	6989.966	-0.315	0.069	-4.559	5.1E-06	1.2E-04
ENSMUSG00000032845	Alpk2	179.655	1.153	0.253	4.556	5.2E-06	1.2E-04
ENSMUSG00000007950	Abhd8	1300.472	-0.510	0.112	-4.555	5.2E-06	1.2E-04
ENSMUSG00000021957	Tkt	2139.716	-0.415	0.091	-4.545	5.5E-06	1.2E-04
ENSMUSG00000002028	Kmt2a	10391.976	-0.402	0.089	-4.538	5.7E-06	1.3E-04
ENSMUSG00000039065	Atpsckmt	875.825	-0.567	0.125	-4.537	5.7E-06	1.3E-04
ENSMUSG00000027562	Car2	8258.184	0.355	0.078	4.536	5.7E-06	1.3E-04
ENSMUSG00000022999	Lmbr1l	570.928	-0.662	0.146	-4.534	5.8E-06	1.3E-04
ENSMUSG00000024186	Rgs11	492.726	-0.682	0.151	-4.531	5.9E-06	1.3E-04
ENSMUSG00000039728	Slc6a5	345.274	-0.807	0.178	-4.523	6.1E-06	1.4E-04
ENSMUSG00000074925	Ptar1	3432.327	-0.368	0.081	-4.521	6.2E-06	1.4E-04
ENSMUSG00000027488	Snta1	451.554	-0.717	0.159	-4.515	6.3E-06	1.4E-04
ENSMUSG00000029088	Kcnip4	912.779	-0.549	0.122	-4.513	6.4E-06	1.4E-04
ENSMUSG00000038658	Ric1	3048.626	-0.369	0.082	-4.513	6.4E-06	1.4E-04
ENSMUSG00000029420	Rimbp2	531.803	-0.662	0.147	-4.512	6.4E-06	1.4E-04
ENSMUSG00000028876	Epha10	318.392	-0.835	0.185	-4.510	6.5E-06	1.4E-04
ENSMUSG00000093865	Lrit3	864.642	-0.576	0.128	-4.509	6.5E-06	1.5E-04
ENSMUSG00000035476	Tab3	1319.541	-0.509	0.113	-4.502	6.7E-06	1.5E-04
ENSMUSG00000002228	Ppm1j	96.905	-1.499	0.333	-4.499	6.8E-06	1.5E-04
ENSMUSG00000036964	Trim17	386.775	-0.800	0.178	-4.499	6.8E-06	1.5E-04
ENSMUSG00000062151	Unc13c	1817.072	-0.417	0.093	-4.498	6.9E-06	1.5E-04
ENSMUSG00000032131	Abcg4	1531.096	-0.458	0.102	-4.489	7.2E-06	1.6E-04
ENSMUSG00000021313	Ryr2	1619.213	-0.462	0.103	-4.488	7.2E-06	1.6E-04
ENSMUSG00000033871	Ppargc1b	550.958	-0.642	0.143	-4.480	7.5E-06	1.6E-04
ENSMUSG00000070683	Lactbl1	2171.294	0.422	0.094	4.470	7.8E-06	1.7E-04
ENSMUSG00000024286	Ccny	1368.629	0.454	0.102	4.465	8.0E-06	1.8E-04
ENSMUSG00000029471	Camkk2	930.168	-0.518	0.116	-4.465	8.0E-06	1.8E-04
ENSMUSG00000033319	Fem1c	1629.820	-0.439	0.098	-4.465	8.0E-06	1.8E-04

ENSMUSG00000021190	Lgmn	2770.831	0.373	0.084	4.461	8.2E-06	1.8E-04
ENSMUSG00000025195	Dnmbp	839.438	-0.573	0.128	-4.456	8.3E-06	1.8E-04
ENSMUSG00000030103	Bhlhe40	542.740	-0.670	0.150	-4.456	8.3E-06	1.8E-04
ENSMUSG00000024008	Cpne5	188.299	-1.095	0.246	-4.454	8.4E-06	1.8E-04
ENSMUSG00000024900	Cpt1a	3934.477	-0.350	0.079	-4.453	8.5E-06	1.8E-04
ENSMUSG00000030276	Ttll3	176.999	-1.072	0.241	-4.453	8.5E-06	1.8E-04
ENSMUSG00000066892	Fbxl12	782.993	-0.550	0.124	-4.445	8.8E-06	1.9E-04
ENSMUSG00000064357	mt-Atp6	140214.640	0.296	0.067	4.437	9.1E-06	2.0E-04
ENSMUSG00000026004	Kansl1l	1228.739	0.485	0.110	4.427	9.6E-06	2.1E-04
ENSMUSG00000094595	Fsbp	511.044	-0.653	0.148	-4.426	9.6E-06	2.1E-04
ENSMUSG00000024210	Ip6k3	52.869	-2.018	0.457	-4.414	1.0E-05	2.2E-04
ENSMUSG00000039158	Akna	519.065	-0.690	0.156	-4.411	1.0E-05	2.2E-04
ENSMUSG00000029720	Gm20605	779.382	-0.600	0.136	-4.410	1.0E-05	2.2E-04
ENSMUSG00000024835	Coro1b	1105.835	-0.485	0.110	-4.410	1.0E-05	2.2E-04
ENSMUSG00000042535	Gtpbp1	2010.975	-0.414	0.094	-4.403	1.1E-05	2.3E-04
ENSMUSG00000021647	Cartpt	345.730	-0.771	0.175	-4.402	1.1E-05	2.3E-04
ENSMUSG00000020866	Cacna1g	767.158	-0.640	0.146	-4.392	1.1E-05	2.4E-04
ENSMUSG00000028312	Smc2	243.109	0.909	0.207	4.385	1.2E-05	2.5E-04
ENSMUSG00000062488	Ifit3b	189.263	1.040	0.237	4.385	1.2E-05	2.5E-04
ENSMUSG00000021706	Zfyve16	1014.054	0.526	0.120	4.384	1.2E-05	2.5E-04
ENSMUSG00000048874	Phf3	3464.320	0.402	0.092	4.377	1.2E-05	2.6E-04
ENSMUSG00000022641	Bbx	1290.679	0.462	0.106	4.375	1.2E-05	2.6E-04
ENSMUSG00000024924	Vldlr	7870.415	-0.309	0.071	-4.376	1.2E-05	2.6E-04
ENSMUSG00000025515	Muc2	136.764	-1.253	0.286	-4.376	1.2E-05	2.6E-04
ENSMUSG00000028137	Celf3	1253.481	-0.464	0.106	-4.375	1.2E-05	2.6E-04
ENSMUSG00000028832	Stmn1	1274.865	-0.474	0.108	-4.371	1.2E-05	2.6E-04
ENSMUSG00000027238	Frmd5	1632.192	-0.431	0.099	-4.367	1.3E-05	2.7E-04
ENSMUSG00000037012	Hk1	10816.396	-0.287	0.066	-4.361	1.3E-05	2.7E-04
ENSMUSG00000032042	Srpr	1319.491	0.459	0.105	4.354	1.3E-05	2.8E-04
ENSMUSG00000040865	Ino80d	2024.910	-0.431	0.099	-4.353	1.3E-05	2.8E-04
ENSMUSG00000031166	Wdr13	1339.627	0.446	0.103	4.344	1.4E-05	2.9E-04
ENSMUSG00000068323	Slc4a5	449.647	-0.679	0.156	-4.341	1.4E-05	3.0E-04
ENSMUSG00000040364	Sec1	31.364	-2.801	0.646	-4.335	1.5E-05	3.0E-04
ENSMUSG00000059991	Nptx2	223.307	-0.924	0.213	-4.334	1.5E-05	3.1E-04
ENSMUSG00000019789	Hey2	324.837	0.784	0.181	4.333	1.5E-05	3.1E-04
ENSMUSG00000027763	Mbnl1	5920.445	0.358	0.083	4.332	1.5E-05	3.1E-04
ENSMUSG00000004631	Sgce	432.291	0.738	0.170	4.330	1.5E-05	3.1E-04
ENSMUSG00000032120	C2cd2l	4132.553	-0.349	0.081	-4.319	1.6E-05	3.2E-04
ENSMUSG00000032294	Pkm	34993.019	-0.270	0.063	-4.319	1.6E-05	3.2E-04
ENSMUSG00000054057	A930004D18Rik	268.198	-0.904	0.209	-4.319	1.6E-05	3.2E-04
ENSMUSG00000002459	Rgs20	1053.961	-0.569	0.132	-4.311	1.6E-05	3.4E-04
ENSMUSG00000029276	Glmn	2466.749	-0.444	0.103	-4.298	1.7E-05	3.6E-04
ENSMUSG00000090223	Pcp4	1421.361	-0.445	0.104	-4.296	1.7E-05	3.6E-04
ENSMUSG00000046404	Yod1	1048.419	-0.482	0.112	-4.288	1.8E-05	3.7E-04
ENSMUSG00000038174	Fam126b	4185.690	-0.332	0.077	-4.287	1.8E-05	3.7E-04
ENSMUSG00000054280	Prr14l	934.724	-0.509	0.119	-4.287	1.8E-05	3.7E-04
ENSMUSG00000022416	Cacna1i	426.460	-0.696	0.163	-4.281	1.9E-05	3.8E-04
ENSMUSG00000025171	Ubtd1	364.856	-0.798	0.187	-4.262	2.0E-05	4.1E-04
ENSMUSG00000047842	Diras2	3217.914	-0.340	0.080	-4.259	2.1E-05	4.2E-04
ENSMUSG00000039205	Ciz1	1020.054	-0.506	0.119	-4.257	2.1E-05	4.2E-04
ENSMUSG00000016921	Srsf6	4963.191	0.315	0.074	4.255	2.1E-05	4.3E-04
ENSMUSG00000018474	Chd3	3945.367	0.370	0.087	4.252	2.1E-05	4.3E-04
ENSMUSG00000024519	Cplx4	11105.384	-0.323	0.076	-4.238	2.3E-05	4.6E-04
ENSMUSG00000037742	Eef1a1	39366.264	0.316	0.075	4.236	2.3E-05	4.6E-04
ENSMUSG00000048706	Lurap1l	891.342	0.511	0.121	4.234	2.3E-05	4.7E-04
ENSMUSG00000030055	Rab43	514.538	-0.684	0.162	-4.230	2.3E-05	4.7E-04

ENSMUSG00000022708	Zbtb20	3561.140	-0.478	0.113	-4.227	2.4E-05	4.8E-04
ENSMUSG00000007655	Cav1	1625.069	0.507	0.120	4.225	2.4E-05	4.8E-04
ENSMUSG00000071654	Uqcc3	438.513	-0.697	0.165	-4.218	2.5E-05	5.0E-04
ENSMUSG00000022358	Fbxo32	439.553	-0.692	0.164	-4.213	2.5E-05	5.1E-04
ENSMUSG00000054580	Pla2r1	6475.373	-0.318	0.076	-4.205	2.6E-05	5.2E-04
ENSMUSG00000007682	Dio2	627.602	-0.630	0.150	-4.205	2.6E-05	5.2E-04
ENSMUSG00000027276	Jag1	2495.092	-0.402	0.096	-4.203	2.6E-05	5.3E-04
ENSMUSG00000048483	Zdhhc22	453.840	-0.674	0.161	-4.198	2.7E-05	5.4E-04
ENSMUSG00000032279	Idh3a	2750.747	0.372	0.089	4.195	2.7E-05	5.5E-04
ENSMUSG00000021832	Psmc6	2600.853	-0.355	0.085	-4.194	2.7E-05	5.5E-04
ENSMUSG00000057132	Rpgrip1	27363.601	-0.304	0.073	-4.193	2.7E-05	5.5E-04
ENSMUSG00000030761	Myo7a	477.995	0.725	0.173	4.192	2.8E-05	5.5E-04
ENSMUSG00000053519	Kcnip1	459.764	-0.676	0.161	-4.188	2.8E-05	5.6E-04
ENSMUSG00000034997	Htr2a	92.130	-1.453	0.347	-4.182	2.9E-05	5.7E-04
ENSMUSG00000058443	Rpl10-ps3	1566.910	0.494	0.118	4.181	2.9E-05	5.8E-04
ENSMUSG00000031613	Hpgd	169.333	-1.015	0.243	-4.172	3.0E-05	6.0E-04
ENSMUSG00000032064	Dixdc1	3574.379	-0.324	0.078	-4.162	3.2E-05	6.2E-04
ENSMUSG00000024556	Me2	689.158	-0.575	0.138	-4.161	3.2E-05	6.3E-04
ENSMUSG00000051413	Plagl2	1146.335	-0.477	0.115	-4.160	3.2E-05	6.3E-04
ENSMUSG00000063524	Eno1	24335.340	-0.272	0.065	-4.157	3.2E-05	6.3E-04
ENSMUSG00000042453	Reln	1924.079	-0.418	0.101	-4.157	3.2E-05	6.4E-04
ENSMUSG00000031393	Mecp2	4370.254	-0.347	0.083	-4.155	3.2E-05	6.4E-04
ENSMUSG00000021796	Bmpr1a	3939.592	-0.332	0.080	-4.155	3.3E-05	6.4E-04
ENSMUSG00000040250	Ints13	1577.383	-0.458	0.110	-4.154	3.3E-05	6.4E-04
ENSMUSG00000064356	mt-Atp8	14506.468	0.305	0.073	4.153	3.3E-05	6.4E-04
ENSMUSG00000016664	Pacsin2	3459.247	-0.325	0.078	-4.148	3.4E-05	6.5E-04
ENSMUSG00000021583	Erap1	700.994	0.546	0.132	4.148	3.4E-05	6.5E-04
ENSMUSG00000095241	Gm5478	56.399	-1.784	0.430	-4.148	3.4E-05	6.5E-04
ENSMUSG00000046456	Tmem150b	11.679	-6.158	1.487	-4.142	3.4E-05	6.7E-04
ENSMUSG00000020062	Slc5a8	202.296	-0.947	0.229	-4.140	3.5E-05	6.7E-04
ENSMUSG00000025733	Rhot2	2347.574	-0.439	0.106	-4.140	3.5E-05	6.7E-04
ENSMUSG00000026163	Sphkap	6496.606	-0.309	0.075	-4.141	3.5E-05	6.7E-04
ENSMUSG00000030287	Itpr2	330.281	-0.795	0.192	-4.141	3.5E-05	6.7E-04
ENSMUSG00000032965	Ift57	600.287	0.587	0.142	4.141	3.5E-05	6.7E-04
ENSMUSG00000059674	Cdh24	100.745	-1.302	0.314	-4.139	3.5E-05	6.7E-04
ENSMUSG00000023050	Map3k12	1168.878	-0.443	0.107	-4.138	3.5E-05	6.7E-04
ENSMUSG00000023267	Gabrr2	901.536	-0.488	0.118	-4.138	3.5E-05	6.7E-04
ENSMUSG00000036990	Otud4	2974.329	-0.339	0.082	-4.138	3.5E-05	6.7E-04
ENSMUSG00000049537	Tecrl	31.455	-2.384	0.576	-4.138	3.5E-05	6.7E-04
ENSMUSG00000113262	Gm48551	777.386	-0.598	0.145	-4.136	3.5E-05	6.8E-04
ENSMUSG00000089736	Tgfbr3l	298.715	-0.825	0.200	-4.128	3.7E-05	7.0E-04
ENSMUSG00000042581	Thsd7b	107.639	-1.268	0.307	-4.126	3.7E-05	7.1E-04
ENSMUSG00000111662	Gm7435	146.671	-1.162	0.282	-4.125	3.7E-05	7.1E-04
ENSMUSG00000019256	Ahr	1053.024	0.460	0.112	4.119	3.8E-05	7.3E-04
ENSMUSG00000055407	Map6	1142.546	-0.455	0.111	-4.117	3.8E-05	7.3E-04
ENSMUSG00000070570	Slc17a7	15636.033	-0.321	0.078	-4.116	3.9E-05	7.3E-04
ENSMUSG00000048281	Dleu7	38.346	-2.168	0.527	-4.112	3.9E-05	7.4E-04
ENSMUSG00000001270	Ckb	6899.262	0.291	0.071	4.106	4.0E-05	7.6E-04
ENSMUSG00000021831	Ero1a	1404.737	-0.441	0.108	-4.102	4.1E-05	7.8E-04
ENSMUSG00000024120	Lrpprc	1864.075	-0.391	0.095	-4.102	4.1E-05	7.8E-04
ENSMUSG00000033526	Ppip5k1	4379.942	-0.341	0.083	-4.099	4.1E-05	7.8E-04
ENSMUSG00000031367	Ap1s2	982.093	0.476	0.116	4.095	4.2E-05	8.0E-04
ENSMUSG00000110218	Tincr	83.052	-1.440	0.352	-4.093	4.3E-05	8.0E-04
ENSMUSG00000060227	Golm2	1378.935	0.445	0.109	4.092	4.3E-05	8.1E-04
ENSMUSG00000020230	Prmt2	396.071	0.675	0.165	4.091	4.3E-05	8.1E-04
ENSMUSG00000022906	Parp9	188.788	1.042	0.255	4.090	4.3E-05	8.1E-04

ENSMUSG00000039954	Stk32a	156.560	-1.066	0.261	-4.090	4.3E-05	8.1E-04
ENSMUSG00000024304	Cdh2	2513.746	0.370	0.091	4.088	4.3E-05	8.1E-04
ENSMUSG00000066258	Trim12a	113.013	1.260	0.308	4.085	4.4E-05	8.2E-04
ENSMUSG00000096696	Zfp960	768.212	-0.550	0.135	-4.085	4.4E-05	8.2E-04
ENSMUSG00000024420	Zfp521	884.901	0.484	0.119	4.083	4.4E-05	8.3E-04
ENSMUSG00000030189	Ybx3	5771.444	-0.333	0.082	-4.080	4.5E-05	8.4E-04
ENSMUSG00000045193	Cirbp	2880.286	-0.344	0.084	-4.080	4.5E-05	8.4E-04
ENSMUSG00000053175	Bcl3	18.460	3.932	0.964	4.080	4.5E-05	8.4E-04
ENSMUSG00000071748	Gm14698	129.720	-1.298	0.318	-4.080	4.5E-05	8.4E-04
ENSMUSG00000018199	Ro60	5246.516	-0.299	0.073	-4.078	4.5E-05	8.4E-04
ENSMUSG00000052151	Plpp2	400.682	-0.683	0.168	-4.072	4.7E-05	8.6E-04
ENSMUSG00000052572	Dlg2	4674.955	-0.398	0.098	-4.068	4.7E-05	8.8E-04
ENSMUSG00000042804	Gpr153	565.940	-0.581	0.143	-4.065	4.8E-05	8.9E-04
ENSMUSG00000043460	Elfn2	1034.731	-0.515	0.127	-4.064	4.8E-05	8.9E-04
ENSMUSG00000039662	Icmt	9113.810	-0.311	0.077	-4.061	4.9E-05	9.0E-04
ENSMUSG00000019920	Lims1	1036.239	-0.481	0.119	-4.059	4.9E-05	9.1E-04
ENSMUSG00000044835	Ankrd45	222.955	0.864	0.213	4.049	5.1E-05	9.5E-04
ENSMUSG00000002109	Ddb2	460.624	-0.624	0.154	-4.046	5.2E-05	9.6E-04
ENSMUSG00000073557	Ppp1r12b	1506.254	-0.460	0.114	-4.045	5.2E-05	9.6E-04
ENSMUSG00000021359	Tfap2a	360.375	-0.695	0.172	-4.043	5.3E-05	9.7E-04
ENSMUSG00000026275	Ppp1r7	2454.572	0.386	0.095	4.039	5.4E-05	9.8E-04
ENSMUSG00000000708	Kat2b	3361.364	0.362	0.090	4.038	5.4E-05	9.8E-04
ENSMUSG00000038276	Asic3	301.579	-0.774	0.192	-4.030	5.6E-05	1.0E-03
ENSMUSG00000111928	Gm48082	367.001	-0.687	0.171	-4.026	5.7E-05	1.0E-03
ENSMUSG00000106631	Gm42669	865.424	-0.482	0.120	-4.020	5.8E-05	1.1E-03
ENSMUSG00000041193	Pla2g5	1330.517	-0.472	0.118	-4.014	6.0E-05	1.1E-03
ENSMUSG00000020290	Xpo1	4048.508	-0.331	0.083	-4.012	6.0E-05	1.1E-03
ENSMUSG00000036585	Fgf1	727.434	-0.527	0.131	-4.012	6.0E-05	1.1E-03
ENSMUSG00000029822	Osbpl3	272.440	-0.784	0.196	-4.006	6.2E-05	1.1E-03
ENSMUSG00000027799	Nbea	5758.642	-0.285	0.071	-4.004	6.2E-05	1.1E-03
ENSMUSG00000022054	Nefm	1553.890	-0.425	0.106	-3.998	6.4E-05	1.2E-03
ENSMUSG00000027963	Extl2	2260.981	0.359	0.090	3.998	6.4E-05	1.2E-03
ENSMUSG00000032010	Usp2	1439.633	-0.409	0.102	-3.998	6.4E-05	1.2E-03
ENSMUSG00000112049	Gm48269	310.211	-0.754	0.188	-3.998	6.4E-05	1.2E-03
ENSMUSG00000048878	Hexim1	856.514	0.480	0.120	3.995	6.5E-05	1.2E-03
ENSMUSG00000019845	Tube1	1088.326	0.445	0.111	3.995	6.5E-05	1.2E-03
ENSMUSG00000002985	Apoe	6595.159	0.283	0.071	3.991	6.6E-05	1.2E-03
ENSMUSG00000029524	Sirt4	550.393	-0.577	0.145	-3.987	6.7E-05	1.2E-03
ENSMUSG00000110622	Iqcn	17.750	-3.266	0.819	-3.986	6.7E-05	1.2E-03
ENSMUSG00000002325	Irf9	330.155	0.714	0.180	3.975	7.1E-05	1.3E-03
ENSMUSG00000020672	Sntg2	467.738	-0.614	0.155	-3.973	7.1E-05	1.3E-03
ENSMUSG00000091844	Gm8251	79.954	-1.401	0.353	-3.973	7.1E-05	1.3E-03
ENSMUSG00000026456	Cyb5r1	758.766	-0.528	0.133	-3.969	7.2E-05	1.3E-03
ENSMUSG00000024045	Akap8	3947.362	-0.317	0.080	-3.966	7.3E-05	1.3E-03
ENSMUSG00000029439	Sfswap	1929.065	-0.376	0.095	-3.958	7.5E-05	1.3E-03
ENSMUSG00000012422	Tmem167	2651.524	-0.330	0.083	-3.956	7.6E-05	1.4E-03
ENSMUSG00000033161	Atp1a1	3690.857	0.333	0.084	3.954	7.7E-05	1.4E-03
ENSMUSG00000021613	Hapln1	50.087	-1.838	0.465	-3.954	7.7E-05	1.4E-03
ENSMUSG00000032463	Faim	2173.185	-0.400	0.101	-3.952	7.8E-05	1.4E-03
ENSMUSG00000028133	Rwdd3	147.494	1.034	0.262	3.947	7.9E-05	1.4E-03
ENSMUSG00000026728	Vim	1382.051	0.405	0.103	3.945	8.0E-05	1.4E-03
ENSMUSG00000028270	Gbp2	115.753	1.178	0.299	3.945	8.0E-05	1.4E-03
ENSMUSG00000042606	Hirip3	880.409	-0.485	0.123	-3.944	8.0E-05	1.4E-03
ENSMUSG00000042807	Hecw2	344.316	0.818	0.207	3.944	8.0E-05	1.4E-03
ENSMUSG00000017737	Mmp9	94.776	-1.332	0.338	-3.944	8.0E-05	1.4E-03
ENSMUSG00000021500	Ddx46	3964.958	0.341	0.086	3.943	8.0E-05	1.4E-03

ENSMUSG00000029797	Sspo	42.348	-2.030	0.515	-3.940	8.2E-05	1.4E-03
ENSMUSG00000040554	Aipl1	9239.079	-0.305	0.077	-3.939	8.2E-05	1.4E-03
ENSMUSG00000006930	Hap1	1134.317	0.435	0.110	3.937	8.2E-05	1.4E-03
ENSMUSG00000033295	Ptprf	807.188	-0.501	0.127	-3.934	8.4E-05	1.5E-03
ENSMUSG00000049076	Acap2	1574.447	-0.385	0.098	-3.933	8.4E-05	1.5E-03
ENSMUSG00000073418	C4b	305.026	1.973	0.502	3.933	8.4E-05	1.5E-03
ENSMUSG00000034187	Nsf	7551.627	-0.270	0.069	-3.932	8.4E-05	1.5E-03
ENSMUSG00000029053	Prkcz	1517.010	-0.479	0.122	-3.930	8.5E-05	1.5E-03
ENSMUSG00000033578	Tmem35a	777.408	0.510	0.130	3.929	8.5E-05	1.5E-03
ENSMUSG00000031626	Sorbs2	1671.738	0.386	0.098	3.925	8.7E-05	1.5E-03
ENSMUSG00000069372	Ctxn3	83.604	-1.498	0.382	-3.924	8.7E-05	1.5E-03
ENSMUSG00000030533	Unc45a	1008.625	-0.444	0.113	-3.924	8.7E-05	1.5E-03
ENSMUSG00000030824	Nucb1	566.016	0.569	0.145	3.923	8.7E-05	1.5E-03
ENSMUSG00000023094	Msrb2	81.327	1.388	0.354	3.921	8.8E-05	1.5E-03
ENSMUSG00000060739	Nsa2	1013.554	0.440	0.112	3.918	8.9E-05	1.5E-03
ENSMUSG00000113388	Gm48111	633.526	-0.558	0.142	-3.918	8.9E-05	1.5E-03
ENSMUSG00000032890	Rims3	1093.021	-0.455	0.116	-3.914	9.1E-05	1.6E-03
ENSMUSG00000040037	Negr1	3305.304	0.321	0.082	3.914	9.1E-05	1.6E-03
ENSMUSG00000071649	B3gat3	765.901	0.500	0.128	3.913	9.1E-05	1.6E-03
ENSMUSG00000022272	Myo10	1085.604	0.502	0.128	3.912	9.2E-05	1.6E-03
ENSMUSG00000025050	Pcgf6	715.110	0.503	0.129	3.911	9.2E-05	1.6E-03
ENSMUSG00000033530	Ttc7b	1663.217	-0.403	0.103	-3.910	9.2E-05	1.6E-03
ENSMUSG00000032525	Nktr	7243.621	-0.353	0.090	-3.907	9.3E-05	1.6E-03
ENSMUSG00000061331	Gm17132	409.217	-0.634	0.162	-3.907	9.4E-05	1.6E-03
ENSMUSG00000015291	Gdi1	5823.028	0.278	0.071	3.900	9.6E-05	1.6E-03
ENSMUSG00000027259	Adal	1437.451	-0.416	0.107	-3.898	9.7E-05	1.7E-03
ENSMUSG00000078619	Smarcd2	452.615	-0.608	0.156	-3.897	9.7E-05	1.7E-03
ENSMUSG00000025665	Rps6ka6	297.586	0.771	0.198	3.896	9.8E-05	1.7E-03
ENSMUSG00000035513	Ntng2	1565.826	-0.377	0.097	-3.890	1.0E-04	1.7E-03
ENSMUSG00000032743	Katnip	3151.886	-0.326	0.084	-3.888	1.0E-04	1.7E-03
ENSMUSG00000038552	Fndc4	770.311	0.521	0.134	3.888	1.0E-04	1.7E-03
ENSMUSG00000056596	Trnp1	1471.116	-0.410	0.105	-3.887	1.0E-04	1.7E-03
ENSMUSG00000024982	Zdhhc6	985.288	-0.449	0.116	-3.884	1.0E-04	1.7E-03
ENSMUSG00000024066	Xdh	485.671	0.604	0.156	3.880	1.0E-04	1.8E-03
ENSMUSG00000037161	Mgarp	11259.183	-0.288	0.074	-3.880	1.0E-04	1.8E-03
ENSMUSG00000044783	Hjurp	1012.534	0.512	0.132	3.879	1.1E-04	1.8E-03
ENSMUSG00000029108	Pcdh7	1110.235	-0.431	0.111	-3.876	1.1E-04	1.8E-03
ENSMUSG00000001604	Tcea3	91.835	1.285	0.332	3.874	1.1E-04	1.8E-03
ENSMUSG00000032589	Bsn	1844.014	-0.397	0.103	-3.867	1.1E-04	1.8E-03
ENSMUSG00000039601	Rcan2	1168.949	-0.438	0.113	-3.863	1.1E-04	1.9E-03
ENSMUSG00000025597	Klhl4	249.845	0.808	0.209	3.861	1.1E-04	1.9E-03
ENSMUSG00000007021	Syngr3	316.436	0.704	0.183	3.852	1.2E-04	2.0E-03
ENSMUSG00000025019	Lcor	1075.576	-0.475	0.123	-3.852	1.2E-04	2.0E-03
ENSMUSG00000029304	Spp1	201.742	-0.886	0.230	-3.852	1.2E-04	2.0E-03
ENSMUSG00000032116	Stt3a	1260.758	0.481	0.125	3.852	1.2E-04	2.0E-03
ENSMUSG00000034472	Rasd2	117.085	-1.139	0.296	-3.851	1.2E-04	2.0E-03
ENSMUSG00000043903	Zfp469	56.926	-1.657	0.430	-3.850	1.2E-04	2.0E-03
ENSMUSG00000069919	Hba-a1	158.880	-0.987	0.256	-3.849	1.2E-04	2.0E-03
ENSMUSG00000106864	Gtf3c2	1829.556	-0.380	0.099	-3.846	1.2E-04	2.0E-03
ENSMUSG00000079056	Kcnip3	551.299	-0.554	0.144	-3.845	1.2E-04	2.0E-03
ENSMUSG00000048142	Nat8l	496.941	-0.745	0.194	-3.844	1.2E-04	2.0E-03
ENSMUSG00000027677	Ttc14	8515.909	-0.267	0.070	-3.842	1.2E-04	2.0E-03
ENSMUSG00000021983	Atp8a2	4934.223	-0.286	0.074	-3.840	1.2E-04	2.0E-03
ENSMUSG00000019894	Slc6a15	1164.636	0.447	0.117	3.837	1.2E-04	2.0E-03
ENSMUSG00000009731	Kcnd1	119.183	-1.196	0.312	-3.834	1.3E-04	2.1E-03
ENSMUSG00000101348	Gm7135	8.750	6.131	1.600	3.831	1.3E-04	2.1E-03

ENSMUSG00000018796	Acsl1	2016.790	0.344	0.090	3.827	1.3E-04	2.1E-03
ENSMUSG00000027071	P2rx3	32.034	-2.387	0.624	-3.826	1.3E-04	2.1E-03
ENSMUSG00000021870	Slmap	4513.849	-0.287	0.075	-3.825	1.3E-04	2.1E-03
ENSMUSG00000030522	Mtmr10	686.041	-0.507	0.133	-3.822	1.3E-04	2.2E-03
ENSMUSG00000046876	Atxn1	2462.166	-0.325	0.085	-3.820	1.3E-04	2.2E-03
ENSMUSG00000047921	Trappc9	1293.408	-0.406	0.106	-3.820	1.3E-04	2.2E-03
ENSMUSG00000072214	Septin5	1194.855	-0.416	0.109	-3.816	1.4E-04	2.2E-03
ENSMUSG00000034936	Arl4d	1180.569	-0.421	0.110	-3.815	1.4E-04	2.2E-03
ENSMUSG00000041670	Rims1	1572.075	-0.380	0.100	-3.813	1.4E-04	2.2E-03
ENSMUSG00000033174	Mgll	820.756	-0.481	0.126	-3.812	1.4E-04	2.2E-03
ENSMUSG00000048612	Myof	262.652	0.813	0.214	3.809	1.4E-04	2.3E-03
ENSMUSG00000027499	Pkia	1980.335	0.368	0.097	3.809	1.4E-04	2.3E-03
ENSMUSG00000031812	Map1lc3b	2160.556	0.347	0.091	3.808	1.4E-04	2.3E-03
ENSMUSG00000037977	6430571L13Rik	402.372	-0.630	0.166	-3.807	1.4E-04	2.3E-03
ENSMUSG00000038766	Gabpb2	2041.344	-0.355	0.093	-3.807	1.4E-04	2.3E-03
ENSMUSG00000013367	Iglon5	460.537	-0.608	0.160	-3.803	1.4E-04	2.3E-03
ENSMUSG00000038550	Ciart	141.725	-1.005	0.265	-3.796	1.5E-04	2.4E-03
ENSMUSG00000039661	Dusp26	882.969	0.467	0.123	3.796	1.5E-04	2.4E-03
ENSMUSG00000046743	Fat4	578.335	-0.575	0.151	-3.797	1.5E-04	2.4E-03
ENSMUSG00000029106	Add1	4240.577	-0.301	0.079	-3.791	1.5E-04	2.4E-03
ENSMUSG00000025492	Ifitm3	83.396	1.361	0.359	3.790	1.5E-04	2.4E-03
ENSMUSG00000023572	Ccndbp1	1836.953	-0.375	0.099	-3.784	1.5E-04	2.5E-03
ENSMUSG00000012187	Mogat1	38.065	-1.970	0.521	-3.783	1.5E-04	2.5E-03
ENSMUSG00000037341	Slc9a7	622.127	-0.542	0.143	-3.781	1.6E-04	2.5E-03
ENSMUSG00000061527	Krt5	18.766	-3.493	0.925	-3.775	1.6E-04	2.6E-03
ENSMUSG00000023913	Pla2g7	3317.620	0.355	0.094	3.773	1.6E-04	2.6E-03
ENSMUSG00000031292	Cdkl5	1491.179	0.433	0.115	3.771	1.6E-04	2.6E-03
ENSMUSG00000064341	mt-Nd1	141200.477	0.252	0.067	3.770	1.6E-04	2.6E-03
ENSMUSG00000042743	Sgtb	1884.554	-0.351	0.093	-3.769	1.6E-04	2.6E-03
ENSMUSG00000028556	Dock7	1504.742	0.373	0.099	3.769	1.6E-04	2.6E-03
ENSMUSG00000051483	Cbr1	545.648	0.543	0.144	3.763	1.7E-04	2.7E-03
ENSMUSG00000037143	Cfap61	541.110	0.571	0.152	3.762	1.7E-04	2.7E-03
ENSMUSG00000000184	Ccnd2	105.592	1.158	0.308	3.761	1.7E-04	2.7E-03
ENSMUSG00000024339	Tap2	147.055	0.984	0.262	3.761	1.7E-04	2.7E-03
ENSMUSG00000029663	Gngt1	20235.445	-0.649	0.173	-3.761	1.7E-04	2.7E-03
ENSMUSG00000053825	Ppfia2	3337.600	0.304	0.081	3.759	1.7E-04	2.7E-03
ENSMUSG00000038538	Ubn2	4706.042	-0.335	0.089	-3.758	1.7E-04	2.7E-03
ENSMUSG00000082415	Gm12486	11.607	-4.707	1.254	-3.753	1.7E-04	2.8E-03
ENSMUSG00000073079	Srp54a	4254.287	-0.300	0.080	-3.751	1.8E-04	2.8E-03
ENSMUSG00000025221	Kcnip2	73.403	-1.400	0.373	-3.749	1.8E-04	2.8E-03
ENSMUSG00000056870	Gulp1	987.788	0.440	0.117	3.749	1.8E-04	2.8E-03
ENSMUSG00000118667	Ahnak2	131.468	-1.070	0.285	-3.748	1.8E-04	2.8E-03
ENSMUSG00000112557	Gm47626	387.352	-0.631	0.168	-3.745	1.8E-04	2.8E-03
ENSMUSG00000005871	Apc	8013.662	-0.311	0.083	-3.745	1.8E-04	2.8E-03
ENSMUSG00000037613	Tnfrsf23	15.796	-3.288	0.878	-3.744	1.8E-04	2.8E-03
ENSMUSG00000031755	Bbs2	1943.193	-0.408	0.109	-3.743	1.8E-04	2.8E-03
ENSMUSG00000041879	Ipo9	3340.570	-0.347	0.093	-3.740	1.8E-04	2.9E-03
ENSMUSG00000050822	Slc29a4	116.526	-1.171	0.313	-3.739	1.8E-04	2.9E-03
ENSMUSG00000022790	Igsf11	1226.928	0.431	0.115	3.734	1.9E-04	2.9E-03
ENSMUSG00000025739	Gng13	526.311	-0.590	0.158	-3.731	1.9E-04	3.0E-03
ENSMUSG00000027293	Ehd4	543.072	-0.589	0.158	-3.730	1.9E-04	3.0E-03
ENSMUSG00000026031	Cflar	1239.415	0.418	0.112	3.727	1.9E-04	3.0E-03
ENSMUSG00000029442	Wdr66	393.861	-0.653	0.175	-3.725	2.0E-04	3.0E-03
ENSMUSG00000030350	Prmt8	352.803	0.664	0.178	3.723	2.0E-04	3.0E-03
ENSMUSG00000040373	Cacng5	364.923	-0.639	0.172	-3.720	2.0E-04	3.1E-03
ENSMUSG00000038524	Fchsd1	97.935	1.212	0.326	3.719	2.0E-04	3.1E-03

ENSMUSG00000037876	Jmjd1c	5830.772	0.285	0.077	3.717	2.0E-04	3.1E-03
ENSMUSG00000025332	Kdm5c	2365.122	-0.338	0.091	-3.712	2.1E-04	3.2E-03
ENSMUSG00000019837	Gtf3c6	597.970	0.542	0.146	3.707	2.1E-04	3.2E-03
ENSMUSG00000025025	Mxi1	1176.525	0.417	0.112	3.707	2.1E-04	3.2E-03
ENSMUSG00000002748	Baz1b	3888.508	-0.292	0.079	-3.705	2.1E-04	3.3E-03
ENSMUSG00000034488	Edil3	3136.801	0.320	0.086	3.702	2.1E-04	3.3E-03
ENSMUSG00000033880	Lgals3bp	195.494	0.866	0.234	3.700	2.2E-04	3.3E-03
ENSMUSG00000075600	Zc3h3	403.726	-0.675	0.183	-3.696	2.2E-04	3.4E-03
ENSMUSG00000024077	Strn	1449.442	-0.418	0.113	-3.695	2.2E-04	3.4E-03
ENSMUSG00000066551	Hmgb1	4758.779	0.296	0.080	3.691	2.2E-04	3.4E-03
ENSMUSG00000074862	BC025920	456.660	-0.594	0.161	-3.691	2.2E-04	3.4E-03
ENSMUSG00000048834	Vstm2a	1110.014	-0.408	0.111	-3.689	2.3E-04	3.4E-03
ENSMUSG00000069917	Hba-a2	169.269	-0.953	0.258	-3.689	2.3E-04	3.4E-03
ENSMUSG00000041598	Cdc42ep4	562.944	-0.538	0.146	-3.685	2.3E-04	3.5E-03
ENSMUSG00000042225	Ammecr1	888.214	-0.437	0.119	-3.685	2.3E-04	3.5E-03
ENSMUSG00000027347	Rasgrp1	338.393	-0.676	0.184	-3.683	2.3E-04	3.5E-03
ENSMUSG00000030554	Synm	817.042	-0.560	0.152	-3.683	2.3E-04	3.5E-03
ENSMUSG00000023456	Tpi1	9303.852	0.266	0.072	3.680	2.3E-04	3.5E-03
ENSMUSG00000028364	Tnc	191.917	-0.920	0.250	-3.679	2.3E-04	3.6E-03
ENSMUSG00000068748	Ptprz1	1465.379	-0.382	0.104	-3.678	2.4E-04	3.6E-03
ENSMUSG00000039860	Srrm3	1100.313	-0.419	0.114	-3.677	2.4E-04	3.6E-03
ENSMUSG00000046020	Pofut1	1161.784	-0.404	0.110	-3.677	2.4E-04	3.6E-03
ENSMUSG00000025777	Gdap1	2029.515	0.334	0.091	3.674	2.4E-04	3.6E-03
ENSMUSG00000062044	Lmtk3	523.452	0.589	0.160	3.674	2.4E-04	3.6E-03
ENSMUSG00000030341	Tnfrsf1a	93.916	1.196	0.326	3.673	2.4E-04	3.6E-03
ENSMUSG00000090667	Mdfic2	63.431	1.492	0.407	3.667	2.5E-04	3.7E-03
ENSMUSG00000015401	Cltrn	89.569	1.262	0.345	3.662	2.5E-04	3.8E-03
ENSMUSG00000021125	Arg2	244.960	-0.755	0.206	-3.661	2.5E-04	3.8E-03
ENSMUSG00000027605	Acss2	749.948	-0.492	0.135	-3.652	2.6E-04	3.9E-03
ENSMUSG00000059772	Slx1b	534.068	-0.532	0.146	-3.649	2.6E-04	3.9E-03
ENSMUSG00000098950	Gm28036	676.103	0.639	0.175	3.647	2.6E-04	4.0E-03
ENSMUSG00000020255	Nopchap1	3071.292	-0.335	0.092	-3.647	2.7E-04	4.0E-03
ENSMUSG00000046805	Mpeg1	118.543	1.054	0.289	3.645	2.7E-04	4.0E-03
ENSMUSG00000021670	Hmgcr	2780.788	-0.303	0.083	-3.642	2.7E-04	4.0E-03
ENSMUSG00000041592	Sdk2	395.452	-0.605	0.166	-3.641	2.7E-04	4.0E-03
ENSMUSG00000029804	Herc3	9154.601	-0.275	0.075	-3.640	2.7E-04	4.1E-03
ENSMUSG00000093909	Gm3883	63.128	1.580	0.435	3.637	2.8E-04	4.1E-03
ENSMUSG00000041258	Zfp236	1950.127	-0.347	0.095	-3.635	2.8E-04	4.1E-03
ENSMUSG00000042050	Dync2i1	1096.795	-0.404	0.111	-3.635	2.8E-04	4.1E-03
ENSMUSG00000055026	Gabrg3	272.186	-0.752	0.207	-3.634	2.8E-04	4.1E-03
ENSMUSG00000074272	Ceacam1	58.304	-1.551	0.427	-3.633	2.8E-04	4.1E-03
ENSMUSG00000070368	Prok1	58.674	-1.577	0.434	-3.631	2.8E-04	4.2E-03
ENSMUSG00000018379	Srsf1	9906.523	-0.243	0.067	-3.630	2.8E-04	4.2E-03
ENSMUSG00000029701	Rbm28	1249.775	0.393	0.108	3.628	2.9E-04	4.2E-03
ENSMUSG00000042444	Mindy2	3538.102	0.302	0.083	3.627	2.9E-04	4.2E-03
ENSMUSG00000029076	Sdf4	2912.877	0.305	0.084	3.624	2.9E-04	4.3E-03
ENSMUSG00000031245	Hmgn5	640.675	0.511	0.141	3.624	2.9E-04	4.3E-03
ENSMUSG00000059866	Tnip2	512.345	-0.587	0.162	-3.622	2.9E-04	4.3E-03
ENSMUSG00000035274	Tpbg	187.756	-0.876	0.242	-3.620	2.9E-04	4.3E-03
ENSMUSG00000041351	Rap1gap	1142.424	-0.394	0.109	-3.620	2.9E-04	4.3E-03
ENSMUSG00000023156	Rpp14	413.390	0.600	0.166	3.619	3.0E-04	4.3E-03
ENSMUSG00000043036	Ccdc63	90.080	-1.213	0.335	-3.619	3.0E-04	4.3E-03
ENSMUSG00000027221	Chst1	222.619	-0.794	0.220	-3.616	3.0E-04	4.4E-03
ENSMUSG00000026915	Strbp	4851.158	-0.272	0.075	-3.615	3.0E-04	4.4E-03
ENSMUSG00000025239	Limd1	275.471	0.716	0.198	3.608	3.1E-04	4.5E-03
ENSMUSG00000036339	Tmem260	1562.331	-0.362	0.100	-3.607	3.1E-04	4.5E-03

ENSMUSG00000020953	Coch	219.106	-0.788	0.219	-3.602	3.2E-04	4.6E-03
ENSMUSG00000029452	Tmem116	38.893	-1.814	0.504	-3.597	3.2E-04	4.7E-03
ENSMUSG00000032489	Kif9	170.135	0.917	0.255	3.594	3.3E-04	4.7E-03
ENSMUSG00000047036	Zfp445	9481.202	-0.240	0.067	-3.592	3.3E-04	4.8E-03
ENSMUSG00000036275	9530068E07Rik	1245.907	0.389	0.108	3.591	3.3E-04	4.8E-03
ENSMUSG00000024642	Tle4	1227.961	0.388	0.108	3.582	3.4E-04	4.9E-03
ENSMUSG00000031636	Pdlim3	249.394	0.887	0.248	3.576	3.5E-04	5.1E-03
ENSMUSG00000033900	Map9	2093.514	0.336	0.094	3.573	3.5E-04	5.1E-03
ENSMUSG00000022490	Ppp1r1a	265.620	-0.703	0.197	-3.573	3.5E-04	5.1E-03
ENSMUSG00000029309	Sparcl1	4646.328	-0.269	0.075	-3.572	3.5E-04	5.1E-03
ENSMUSG00000050930	Map10	588.422	-0.498	0.139	-3.570	3.6E-04	5.1E-03
ENSMUSG00000030342	Cd9	781.511	0.453	0.127	3.567	3.6E-04	5.2E-03
ENSMUSG00000042046	Dstyk	1929.534	0.335	0.094	3.567	3.6E-04	5.2E-03
ENSMUSG00000029090	Adgra3	188.834	0.828	0.232	3.565	3.6E-04	5.2E-03
ENSMUSG00000029632	Ndufa4	1685.396	-0.418	0.117	-3.565	3.6E-04	5.2E-03
ENSMUSG00000037712	Fermt2	791.911	0.469	0.132	3.563	3.7E-04	5.3E-03
ENSMUSG00000055639	Dach1	875.453	0.428	0.120	3.562	3.7E-04	5.3E-03
ENSMUSG00000074909	Ranbp6	2422.165	-0.342	0.096	-3.561	3.7E-04	5.3E-03
ENSMUSG00000036760	Kcnk9	336.542	-0.691	0.194	-3.561	3.7E-04	5.3E-03
ENSMUSG00000022855	Senp2	2507.251	-0.340	0.095	-3.560	3.7E-04	5.3E-03
ENSMUSG00000029718	Pcolce	99.645	1.199	0.337	3.560	3.7E-04	5.3E-03
ENSMUSG00000095041		2898.540	0.294	0.083	3.557	3.8E-04	5.4E-03
ENSMUSG00000058975	Kcnc1	1252.738	-0.438	0.123	-3.554	3.8E-04	5.4E-03
ENSMUSG00000028661	Epha8	316.005	-0.647	0.182	-3.552	3.8E-04	5.4E-03
ENSMUSG00000020340	Cyfip2	4076.629	-0.297	0.084	-3.551	3.8E-04	5.5E-03
ENSMUSG00000035914	Cd276	339.925	0.775	0.218	3.550	3.8E-04	5.5E-03
ENSMUSG00000037148	Arhgap10	809.591	-0.434	0.122	-3.550	3.8E-04	5.5E-03
ENSMUSG00000019189	Rnf145	2951.599	0.295	0.083	3.549	3.9E-04	5.5E-03
ENSMUSG00000047888	Tnrc6b	7920.887	-0.670	0.189	-3.549	3.9E-04	5.5E-03
ENSMUSG00000014355	Anapc1	3662.804	0.356	0.100	3.548	3.9E-04	5.5E-03
ENSMUSG00000019082	Slc25a22	1714.591	-0.338	0.095	-3.546	3.9E-04	5.5E-03
ENSMUSG00000037857	Nufip2	2782.450	-0.293	0.083	-3.546	3.9E-04	5.5E-03
ENSMUSG00000107476	Zfp862-ps	405.848	-0.615	0.173	-3.546	3.9E-04	5.5E-03
ENSMUSG00000060733	Ipmk	3156.626	-0.306	0.086	-3.545	3.9E-04	5.5E-03
ENSMUSG00000026365	Cfh	496.199	0.542	0.153	3.543	4.0E-04	5.6E-03
ENSMUSG00000032702	Kank1	921.250	-0.442	0.125	-3.543	4.0E-04	5.6E-03
ENSMUSG00000059003	Grin2a	285.130	-0.691	0.195	-3.542	4.0E-04	5.6E-03
ENSMUSG00000038070	Cntln	1293.330	-0.365	0.103	-3.542	4.0E-04	5.6E-03
ENSMUSG00000034462	Pkd2	1050.210	0.426	0.120	3.541	4.0E-04	5.6E-03
ENSMUSG00000074037	Mc1r	40.900	-1.877	0.530	-3.541	4.0E-04	5.6E-03
ENSMUSG00000022257	Laptm4b	5681.797	-0.282	0.080	-3.539	4.0E-04	5.6E-03
ENSMUSG00000060923	Acyp2	150.735	1.015	0.287	3.539	4.0E-04	5.6E-03
ENSMUSG00000028777	Gnat3	16.418	2.999	0.848	3.538	4.0E-04	5.6E-03
ENSMUSG00000020263	Appl2	3331.085	-0.298	0.084	-3.537	4.0E-04	5.7E-03
ENSMUSG00000028461	Ccdc107	445.320	-0.584	0.165	-3.533	4.1E-04	5.7E-03
ENSMUSG00000022508	Bcl6	304.843	0.664	0.188	3.530	4.1E-04	5.8E-03
ENSMUSG00000025159	Mms19	1127.210	-0.427	0.121	-3.530	4.2E-04	5.8E-03
ENSMUSG00000050343	Olfr1378	12.109	-3.510	0.994	-3.530	4.2E-04	5.8E-03
ENSMUSG00000079083	Jrkl	852.792	-0.441	0.125	-3.529	4.2E-04	5.8E-03
ENSMUSG00000006057	Atp5g1	672.857	0.480	0.136	3.526	4.2E-04	5.9E-03
ENSMUSG00000025420	Katnal2	283.579	-0.689	0.196	-3.524	4.2E-04	5.9E-03
ENSMUSG00000027273	Snap25	21090.885	-0.229	0.065	-3.523	4.3E-04	5.9E-03
ENSMUSG00000054256	Msi1	5484.098	-0.264	0.075	-3.520	4.3E-04	6.0E-03
ENSMUSG00000075700	Selenot	5055.339	0.284	0.081	3.519	4.3E-04	6.0E-03
ENSMUSG00000040363	Bcor	1438.658	0.350	0.100	3.518	4.3E-04	6.0E-03
ENSMUSG00000028910	Mecr	300.852	-0.656	0.187	-3.515	4.4E-04	6.1E-03

ENSMUSG00000051242	Pcdhb9	341.123	0.624	0.178	3.514	4.4E-04	6.1E-03
ENSMUSG00000026637	Traf5	361.682	-0.603	0.172	-3.511	4.5E-04	6.2E-03
ENSMUSG00000042082	Arsb	790.264	0.440	0.125	3.505	4.6E-04	6.3E-03
ENSMUSG00000009739	Pou6f1	1479.997	-0.376	0.107	-3.504	4.6E-04	6.3E-03
ENSMUSG00000049744	Arhgap15	96.011	-1.142	0.326	-3.500	4.6E-04	6.4E-03
ENSMUSG00000022661	Cd200	725.815	0.453	0.129	3.497	4.7E-04	6.5E-03
ENSMUSG00000025380	Fscn2	1428.384	-0.369	0.105	-3.497	4.7E-04	6.5E-03
ENSMUSG00000032067	Pts	909.840	0.439	0.125	3.497	4.7E-04	6.5E-03
ENSMUSG00000014551	Mrps25	516.708	0.668	0.191	3.496	4.7E-04	6.5E-03
ENSMUSG00000024735	Prpf19	1466.189	0.385	0.110	3.490	4.8E-04	6.6E-03
ENSMUSG00000022797	Tfrc	5077.377	0.282	0.081	3.486	4.9E-04	6.7E-03
ENSMUSG00000036046	5031439G07Rik	1030.961	-0.390	0.112	-3.486	4.9E-04	6.7E-03
ENSMUSG00000021327	Zkscan3	1666.914	-0.348	0.100	-3.486	4.9E-04	6.7E-03
ENSMUSG00000031904	Slc7a6	539.063	0.526	0.151	3.486	4.9E-04	6.7E-03
ENSMUSG00000078853	Igtp	123.658	0.985	0.283	3.485	4.9E-04	6.7E-03
ENSMUSG00000030270	Cpne9	137.376	-1.062	0.305	-3.484	4.9E-04	6.7E-03
ENSMUSG00000021555	Naa35	1255.833	0.379	0.109	3.483	5.0E-04	6.7E-03
ENSMUSG00000002010	Idh3g	1219.286	0.414	0.119	3.482	5.0E-04	6.8E-03
ENSMUSG00000029580	Actb	4580.753	0.258	0.074	3.482	5.0E-04	6.8E-03
ENSMUSG00000032458	Copb2	2929.480	0.286	0.082	3.480	5.0E-04	6.8E-03
ENSMUSG00000022186	Oxct1	2055.662	0.335	0.096	3.479	5.0E-04	6.8E-03
ENSMUSG00000063952	Brpf3	553.110	0.524	0.151	3.478	5.0E-04	6.8E-03
ENSMUSG00000023805	Synj2	459.309	-0.569	0.164	-3.476	5.1E-04	6.9E-03
ENSMUSG00000016477	E2f3	502.977	0.534	0.154	3.475	5.1E-04	6.9E-03
ENSMUSG00000054404	Slfn5	105.823	1.130	0.325	3.475	5.1E-04	6.9E-03
ENSMUSG00000021219	Rgs6	862.129	-0.415	0.120	-3.472	5.2E-04	7.0E-03
ENSMUSG00000014813	Stc1	130.460	-1.015	0.292	-3.471	5.2E-04	7.0E-03
ENSMUSG00000025151	Maged1	4812.967	0.256	0.074	3.471	5.2E-04	7.0E-03
ENSMUSG00000030757	Zkscan2	2357.266	-0.333	0.096	-3.467	5.3E-04	7.1E-03
ENSMUSG00000031805	Jak3	40.692	1.879	0.542	3.464	5.3E-04	7.1E-03
ENSMUSG00000029086	Prom1	13978.085	-0.226	0.065	-3.462	5.4E-04	7.2E-03
ENSMUSG00000039879	Heca	733.784	0.549	0.159	3.462	5.4E-04	7.2E-03
ENSMUSG00000023041	Krt6b	13.594	-3.737	1.080	-3.461	5.4E-04	7.2E-03
ENSMUSG00000072437	Nanos1	267.126	-0.702	0.203	-3.461	5.4E-04	7.2E-03
ENSMUSG00000034813	Grip1	771.534	-0.454	0.131	-3.461	5.4E-04	7.2E-03
ENSMUSG00000045994	B3gat1	1050.672	-0.414	0.120	-3.457	5.5E-04	7.3E-03
ENSMUSG00000066643	Wdr35	1467.723	0.427	0.123	3.457	5.5E-04	7.3E-03
ENSMUSG00000071350	Setdb2	346.227	0.679	0.197	3.455	5.5E-04	7.3E-03
ENSMUSG00000043621	Ubxn10	95.347	1.134	0.329	3.452	5.6E-04	7.4E-03
ENSMUSG00000032570	Atp2c1	4268.284	0.259	0.075	3.449	5.6E-04	7.5E-03
ENSMUSG00000040253	Gbp7	247.435	0.709	0.206	3.449	5.6E-04	7.5E-03
ENSMUSG00000022216	Psme1	421.832	0.553	0.160	3.447	5.7E-04	7.5E-03
ENSMUSG00000020610	Amz2	1485.250	-0.365	0.106	-3.447	5.7E-04	7.5E-03
ENSMUSG00000023010	Tmbim6	3933.729	-0.270	0.078	-3.447	5.7E-04	7.5E-03
ENSMUSG00000061603	Akap6	2074.430	-0.358	0.104	-3.444	5.7E-04	7.6E-03
ENSMUSG00000071379	Hpcal1	552.534	-0.515	0.150	-3.439	5.8E-04	7.7E-03
ENSMUSG00000040188	Scamp2	470.516	-0.536	0.156	-3.435	5.9E-04	7.8E-03
ENSMUSG00000039543	Cfap70	81.651	-1.199	0.349	-3.431	6.0E-04	7.9E-03
ENSMUSG00000032959	Pebp1	2272.117	0.326	0.095	3.431	6.0E-04	7.9E-03
ENSMUSG00000003031	Cdkn1b	2283.822	0.304	0.089	3.431	6.0E-04	7.9E-03
ENSMUSG00000035270	Impg2	11756.524	0.225	0.066	3.430	6.0E-04	7.9E-03
ENSMUSG00000000915	Hip1r	571.592	0.517	0.151	3.430	6.0E-04	7.9E-03
ENSMUSG00000042686	Jph1	72.695	1.336	0.390	3.428	6.1E-04	8.0E-03
ENSMUSG00000032336	Nptn	4441.128	-0.259	0.076	-3.426	6.1E-04	8.1E-03
ENSMUSG00000028709	Mob3c	210.563	0.801	0.234	3.420	6.3E-04	8.2E-03
ENSMUSG00000072889	Nfxl1	463.115	0.558	0.163	3.419	6.3E-04	8.2E-03

ENSMUSG00000037343	Taf2	1203.265	0.362	0.106	3.417	6.3E-04	8.3E-03
ENSMUSG00000069044	Usp9y	12.665	4.419	1.295	3.413	6.4E-04	8.4E-03
ENSMUSG00000053693	Mast1	1100.787	-0.376	0.110	-3.410	6.5E-04	8.5E-03
ENSMUSG00000016763	Scube1	31.118	1.995	0.586	3.402	6.7E-04	8.7E-03
ENSMUSG00000062190	Lancl2	991.230	0.390	0.115	3.398	6.8E-04	8.9E-03
ENSMUSG00000038816	Ctnnal1	628.792	0.558	0.164	3.397	6.8E-04	8.9E-03
ENSMUSG00000031709	Tbc1d9	1051.055	0.383	0.113	3.396	6.8E-04	8.9E-03
ENSMUSG00000038628	Polr3k	1712.579	0.333	0.098	3.396	6.8E-04	8.9E-03
ENSMUSG00000035413	Tmem98	134.531	0.937	0.276	3.394	6.9E-04	9.0E-03
ENSMUSG00000027940	Tpm3	1505.871	-0.339	0.100	-3.393	6.9E-04	9.0E-03
ENSMUSG00000038418	Egr1	540.143	1.474	0.434	3.392	6.9E-04	9.0E-03
ENSMUSG00000037234	Hook3	7075.855	-0.253	0.075	-3.391	7.0E-04	9.0E-03
ENSMUSG00000037541	Shank2	1139.490	-0.454	0.134	-3.388	7.0E-04	9.1E-03
ENSMUSG00000000120	Ngfr	294.204	-0.636	0.188	-3.387	7.1E-04	9.2E-03
ENSMUSG00000024558	Mapk4	186.322	-0.805	0.238	-3.386	7.1E-04	9.2E-03
ENSMUSG00000006932	Ctnnb1	5712.917	0.260	0.077	3.384	7.1E-04	9.2E-03
ENSMUSG00000023033	Scn8a	2942.620	0.326	0.096	3.384	7.2E-04	9.2E-03
ENSMUSG00000024096	Ralbp1	1176.244	0.362	0.107	3.384	7.2E-04	9.2E-03
ENSMUSG00000022540	Rogdi	1043.566	-0.376	0.111	-3.383	7.2E-04	9.3E-03
ENSMUSG00000025066	Sfr1	1710.474	0.344	0.102	3.383	7.2E-04	9.3E-03
ENSMUSG00000032202	Rab27a	320.204	0.660	0.195	3.382	7.2E-04	9.3E-03
ENSMUSG00000071866	Ppia	4497.598	0.303	0.090	3.380	7.2E-04	9.3E-03
ENSMUSG00000019996	Map7	5716.331	-0.291	0.086	-3.380	7.3E-04	9.3E-03
ENSMUSG00000044712	Slc38a6	1506.126	-0.382	0.113	-3.378	7.3E-04	9.4E-03
ENSMUSG00000036887	C1qa	78.551	1.251	0.371	3.374	7.4E-04	9.5E-03
ENSMUSG00000020585	Laptm4a	2145.613	0.307	0.091	3.374	7.4E-04	9.5E-03
ENSMUSG00000028639	Ybx1	6780.503	-0.263	0.078	-3.371	7.5E-04	9.6E-03
ENSMUSG00000024978	Gpam	1557.894	-0.348	0.103	-3.369	7.5E-04	9.7E-03
ENSMUSG00000027210	Meis2	2092.214	-0.333	0.099	-3.368	7.6E-04	9.7E-03
ENSMUSG00000020048	Hsp90b1	11026.735	-0.239	0.071	-3.365	7.7E-04	9.8E-03
ENSMUSG00000010608	Rbm25	8824.556	-0.247	0.074	-3.363	7.7E-04	9.9E-03
ENSMUSG00000056917	Sipa1	114.938	-1.020	0.304	-3.361	7.8E-04	9.9E-03
ENSMUSG00000000751	Rpa1	1978.726	-0.319	0.095	-3.360	7.8E-04	9.9E-03
ENSMUSG00000037492	Zmat4	774.786	-0.424	0.126	-3.357	7.9E-04	1.0E-02
ENSMUSG00000027668	Mfn1	1581.650	0.329	0.098	3.355	7.9E-04	1.0E-02
ENSMUSG00000029910	Mad2l1	482.299	-0.529	0.158	-3.355	7.9E-04	1.0E-02
ENSMUSG00000034121	Mks1	291.080	-0.641	0.191	-3.355	7.9E-04	1.0E-02
ENSMUSG00000024507	Hsd17b4	986.060	0.406	0.121	3.353	8.0E-04	1.0E-02
ENSMUSG00000043336	Filip1l	557.723	-0.487	0.145	-3.352	8.0E-04	1.0E-02
ENSMUSG00000048108	Tmem72	1172.069	-0.400	0.119	-3.351	8.1E-04	1.0E-02
ENSMUSG00000060098	Prmt7	683.166	-0.468	0.140	-3.350	8.1E-04	1.0E-02
ENSMUSG00000062933	Gm10123	2352.226	0.335	0.100	3.347	8.2E-04	1.0E-02
ENSMUSG00000005514	Por	698.944	-0.454	0.136	-3.342	8.3E-04	1.0E-02
ENSMUSG00000023186	Vwa5a	354.124	0.589	0.176	3.341	8.3E-04	1.1E-02
ENSMUSG00000064215	Ifi27	85.357	1.197	0.358	3.341	8.4E-04	1.1E-02
ENSMUSG00000026565	Pou2f1	2326.881	-0.357	0.107	-3.338	8.4E-04	1.1E-02
ENSMUSG00000028246	Faxc	912.971	-0.411	0.123	-3.338	8.4E-04	1.1E-02
ENSMUSG00000034573	Ptpn13	410.991	0.564	0.169	3.338	8.4E-04	1.1E-02
ENSMUSG00000029826	Zc3hav1	117.052	1.013	0.304	3.335	8.5E-04	1.1E-02
ENSMUSG00000000168	Dlat	1040.906	0.384	0.115	3.334	8.6E-04	1.1E-02
ENSMUSG00000020121	Srgap1	283.185	-0.696	0.209	-3.332	8.6E-04	1.1E-02
ENSMUSG00000060206	Zfp462	3262.437	0.331	0.099	3.331	8.6E-04	1.1E-02
ENSMUSG00000045903	Npas4	65.424	-1.336	0.401	-3.329	8.7E-04	1.1E-02
ENSMUSG00000056458	Mok	407.920	-0.553	0.166	-3.329	8.7E-04	1.1E-02
ENSMUSG00000025893	Kbtbd3	406.614	-0.547	0.164	-3.328	8.8E-04	1.1E-02
ENSMUSG00000032076	Cadm1	6767.087	-0.259	0.078	-3.326	8.8E-04	1.1E-02

ENSMUSG00000050628	Ubald2	269.102	0.688	0.207	3.326	8.8E-04	1.1E-02
ENSMUSG00000040760	Appl1	3272.226	-0.266	0.080	-3.326	8.8E-04	1.1E-02
ENSMUSG00000029209	Gnpda2	738.062	0.463	0.139	3.325	8.8E-04	1.1E-02
ENSMUSG00000027893	Ahcyl1	8894.302	-0.267	0.080	-3.320	9.0E-04	1.1E-02
ENSMUSG00000055116	Arntl	2104.499	0.295	0.089	3.320	9.0E-04	1.1E-02
ENSMUSG00000022474	Pmm1	1140.828	-0.372	0.112	-3.318	9.1E-04	1.1E-02
ENSMUSG00000082676	Gm11843	9.162	-4.388	1.323	-3.316	9.1E-04	1.1E-02
ENSMUSG00000023236	Scg5	3186.078	0.290	0.087	3.315	9.2E-04	1.1E-02
ENSMUSG00000030279	C2cd5	2613.313	-0.314	0.095	-3.314	9.2E-04	1.1E-02
ENSMUSG00000035578	Iqcg	376.689	-0.656	0.198	-3.314	9.2E-04	1.1E-02
ENSMUSG00000026204	Ptprn	1445.425	0.441	0.133	3.314	9.2E-04	1.1E-02
ENSMUSG00000054423	Cadps	5001.148	-0.265	0.080	-3.312	9.3E-04	1.1E-02
ENSMUSG00000025184	R3hcc1l	1104.205	0.459	0.139	3.307	9.4E-04	1.2E-02
ENSMUSG00000071636	Rimbp3	111.829	-1.032	0.312	-3.306	9.5E-04	1.2E-02
ENSMUSG00000028214	Gem	333.519	-0.586	0.177	-3.306	9.5E-04	1.2E-02
ENSMUSG00000078851	H2aw	1941.309	-0.303	0.092	-3.306	9.5E-04	1.2E-02
ENSMUSG00000038773	Kdm3b	3215.949	-0.266	0.080	-3.305	9.5E-04	1.2E-02
ENSMUSG00000030302	Atp2b2	1637.053	-0.332	0.100	-3.302	9.6E-04	1.2E-02
ENSMUSG00000032840	Spring1	981.873	-0.395	0.120	-3.302	9.6E-04	1.2E-02
ENSMUSG00000110685	Gm5373	279.252	-0.694	0.210	-3.301	9.6E-04	1.2E-02
ENSMUSG00000031488	Rab11fip1	139.683	-0.909	0.275	-3.299	9.7E-04	1.2E-02
ENSMUSG00000038805	Six3	948.906	-0.389	0.118	-3.299	9.7E-04	1.2E-02
ENSMUSG00000066798	Zbtb6	1475.743	-0.346	0.105	-3.300	9.7E-04	1.2E-02
ENSMUSG00000067149	Jchain	205.899	-0.764	0.232	-3.300	9.7E-04	1.2E-02
ENSMUSG00000091264	Smim13	3529.396	0.266	0.081	3.299	9.7E-04	1.2E-02
ENSMUSG00000001774	Chordc1	1606.406	0.330	0.100	3.298	9.7E-04	1.2E-02
ENSMUSG00000059742	Kcnh7	658.193	-0.445	0.135	-3.296	9.8E-04	1.2E-02
ENSMUSG00000001995	Sipa1l2	858.669	-0.411	0.125	-3.293	9.9E-04	1.2E-02
ENSMUSG00000029229	Chic2	627.040	0.455	0.138	3.293	9.9E-04	1.2E-02
ENSMUSG00000021518	Ptdss1	698.635	0.426	0.129	3.292	9.9E-04	1.2E-02
ENSMUSG00000029038	Ssu72	1291.166	-0.369	0.112	-3.291	1.0E-03	1.2E-02
ENSMUSG00000046324	Ermp1	556.655	0.520	0.158	3.291	1.0E-03	1.2E-02
ENSMUSG00000017778	Cox7c	1024.027	0.377	0.115	3.290	1.0E-03	1.2E-02
ENSMUSG00000019122	Ccl9	29.647	-2.095	0.637	-3.290	1.0E-03	1.2E-02
ENSMUSG00000017167	Cntnap1	745.654	-0.417	0.127	-3.289	1.0E-03	1.2E-02
ENSMUSG00000022957	Itsn1	2887.992	-0.307	0.093	-3.289	1.0E-03	1.2E-02
ENSMUSG00000036896	C1qc	63.250	1.299	0.395	3.286	1.0E-03	1.2E-02
ENSMUSG00000039630	Hnrnpu	20453.649	-0.208	0.063	-3.286	1.0E-03	1.2E-02
ENSMUSG00000052562	Slc22a30	45.967	-1.530	0.466	-3.286	1.0E-03	1.2E-02
ENSMUSG00000033705	Stard9	291.482	-0.667	0.203	-3.285	1.0E-03	1.2E-02
ENSMUSG00000046841	Ckap4	436.859	0.535	0.163	3.282	1.0E-03	1.2E-02
ENSMUSG00000057130	Txnl4a	231.341	-0.806	0.245	-3.282	1.0E-03	1.2E-02
ENSMUSG00000027615	Hps3	1185.077	-0.361	0.110	-3.281	1.0E-03	1.2E-02
ENSMUSG00000032383	Ppib	1440.895	0.343	0.105	3.279	1.0E-03	1.3E-02
ENSMUSG00000061882	Ccdc62	196.319	-0.766	0.233	-3.279	1.0E-03	1.3E-02
ENSMUSG00000028995	Fam126a	481.042	-0.494	0.151	-3.279	1.0E-03	1.3E-02
ENSMUSG00000007279	Scube2	82.200	-1.155	0.352	-3.278	1.0E-03	1.3E-02
ENSMUSG00000068551	Zfp467	363.392	-0.584	0.178	-3.276	1.1E-03	1.3E-02
ENSMUSG00000027742	Cog6	1099.215	0.365	0.111	3.275	1.1E-03	1.3E-02
ENSMUSG00000053963	Stum	104.774	-1.117	0.341	-3.274	1.1E-03	1.3E-02
ENSMUSG00000031333	Abcb7	2253.899	0.293	0.090	3.273	1.1E-03	1.3E-02
ENSMUSG00000024430	Cabyr	55.052	-1.411	0.431	-3.272	1.1E-03	1.3E-02
ENSMUSG00000029283	Cdc7	834.091	-0.396	0.121	-3.271	1.1E-03	1.3E-02
ENSMUSG00000005161	Prdx2	1861.846	0.309	0.095	3.270	1.1E-03	1.3E-02
ENSMUSG00000006567	Atp7b	334.508	-0.592	0.181	-3.270	1.1E-03	1.3E-02
ENSMUSG00000039913	Pak5	1237.972	-0.350	0.107	-3.270	1.1E-03	1.3E-02

ENSMUSG00000031517	Gpm6a	9434.878	0.267	0.082	3.269	1.1E-03	1.3E-02
ENSMUSG00000032034	Kcnj5	212.987	-0.743	0.227	-3.269	1.1E-03	1.3E-02
ENSMUSG00000024935	Slc1a1	877.161	0.403	0.123	3.267	1.1E-03	1.3E-02
ENSMUSG00000044700	Tmem201	1171.978	-0.358	0.110	-3.267	1.1E-03	1.3E-02
ENSMUSG00000028931	Kcnab2	3234.116	-0.283	0.087	-3.265	1.1E-03	1.3E-02
ENSMUSG00000032396	Dis3l	448.379	0.529	0.162	3.265	1.1E-03	1.3E-02
ENSMUSG00000029816	Gpnmb	179.403	0.772	0.237	3.263	1.1E-03	1.3E-02
ENSMUSG00000031837	Necab2	231.038	-0.706	0.217	-3.263	1.1E-03	1.3E-02
ENSMUSG00000027475	Kif3b	2241.692	-0.289	0.089	-3.262	1.1E-03	1.3E-02
ENSMUSG00000025534	Gusb	140.046	0.888	0.272	3.259	1.1E-03	1.3E-02
ENSMUSG00000017210	Med24	1606.222	0.330	0.101	3.258	1.1E-03	1.3E-02
ENSMUSG00000053716	Dusp7	782.289	-0.419	0.129	-3.256	1.1E-03	1.3E-02
ENSMUSG00000038011	Dnah10	11.668	-3.185	0.978	-3.255	1.1E-03	1.3E-02
ENSMUSG00000024287	Thoc1	1378.490	0.333	0.102	3.254	1.1E-03	1.3E-02
ENSMUSG00000027010	Slc25a12	1808.328	0.360	0.111	3.253	1.1E-03	1.3E-02
ENSMUSG00000041653	Pnpla3	301.125	-0.687	0.211	-3.252	1.1E-03	1.3E-02
ENSMUSG00000004207	Psap	6641.396	0.235	0.072	3.251	1.1E-03	1.4E-02
ENSMUSG00000005417	Mprip	4326.140	0.245	0.075	3.250	1.2E-03	1.4E-02
ENSMUSG00000068699	Flnc	41.668	2.075	0.638	3.250	1.2E-03	1.4E-02
ENSMUSG00000024276	Zfp397	2447.794	-0.287	0.088	-3.246	1.2E-03	1.4E-02
ENSMUSG00000054728	Phactr1	2234.482	0.296	0.091	3.246	1.2E-03	1.4E-02
ENSMUSG00000110206	Flt3l	102.996	1.024	0.316	3.245	1.2E-03	1.4E-02
ENSMUSG00000073139	Tmem185a	1287.172	0.339	0.104	3.245	1.2E-03	1.4E-02
ENSMUSG00000030199	Etv6	532.750	0.474	0.146	3.245	1.2E-03	1.4E-02
ENSMUSG00000021936	Mapk8	2606.862	0.278	0.086	3.242	1.2E-03	1.4E-02
ENSMUSG00000026662	Sephs1	1002.483	0.379	0.117	3.239	1.2E-03	1.4E-02
ENSMUSG00000020362	Cnot6	6370.503	-0.242	0.075	-3.237	1.2E-03	1.4E-02
ENSMUSG00000056258	Kcnq3	259.866	-0.656	0.203	-3.237	1.2E-03	1.4E-02
ENSMUSG00000032456	Nmnat3	87.670	1.121	0.346	3.236	1.2E-03	1.4E-02
ENSMUSG00000029313	Aff1	4436.476	-0.242	0.075	-3.235	1.2E-03	1.4E-02
ENSMUSG00000032739	Pram1	90.869	-1.111	0.344	-3.235	1.2E-03	1.4E-02
ENSMUSG00000016534	Lamp2	2171.749	0.286	0.089	3.233	1.2E-03	1.4E-02
ENSMUSG00000031570	Plpp5	386.847	0.584	0.181	3.230	1.2E-03	1.4E-02
ENSMUSG00000022208	Jph4	364.518	-0.610	0.189	-3.229	1.2E-03	1.4E-02
ENSMUSG00000042015	Wdr41	763.772	0.409	0.127	3.229	1.2E-03	1.4E-02
ENSMUSG00000031483	Erlin2	2143.071	0.286	0.089	3.228	1.2E-03	1.4E-02
ENSMUSG00000022911	Arl13b	1736.020	-0.341	0.106	-3.227	1.3E-03	1.4E-02
ENSMUSG00000018567	Gabarap	1160.148	0.370	0.115	3.225	1.3E-03	1.5E-02
ENSMUSG00000044201	Cdc25c	20.538	-2.334	0.723	-3.225	1.3E-03	1.5E-02
ENSMUSG00000036699	Zcchc12	218.069	0.696	0.216	3.225	1.3E-03	1.5E-02
ENSMUSG00000021478	Drd1	704.352	-0.445	0.138	-3.224	1.3E-03	1.5E-02
ENSMUSG00000091402	Rd3l	1507.325	0.336	0.104	3.224	1.3E-03	1.5E-02
ENSMUSG00000033768	Nrxn2	1245.542	-0.401	0.125	-3.220	1.3E-03	1.5E-02
ENSMUSG00000034427	Myo15b	20.169	-2.266	0.704	-3.220	1.3E-03	1.5E-02
ENSMUSG00000071076	Jund	469.344	0.495	0.154	3.220	1.3E-03	1.5E-02
ENSMUSG00000040276	Pacsin1	3882.434	-0.268	0.083	-3.219	1.3E-03	1.5E-02
ENSMUSG00000001473	Tubb6	16.442	2.871	0.892	3.218	1.3E-03	1.5E-02
ENSMUSG00000028820	Sfpq	6137.558	-0.258	0.080	-3.214	1.3E-03	1.5E-02
ENSMUSG00000022148	Fyb	16.137	2.589	0.806	3.212	1.3E-03	1.5E-02
ENSMUSG00000018486	Wnt9b	10.442	-3.867	1.205	-3.208	1.3E-03	1.5E-02
ENSMUSG00000068011	Mkrn2os	102.095	1.103	0.344	3.208	1.3E-03	1.5E-02
ENSMUSG00000031748	Gnao1	5449.357	-0.233	0.073	-3.206	1.3E-03	1.5E-02
ENSMUSG00000017286	Glod4	915.566	0.378	0.118	3.205	1.4E-03	1.5E-02
ENSMUSG00000025178	Pi4k2a	830.237	0.392	0.122	3.205	1.4E-03	1.5E-02
ENSMUSG00000040483	Xaf1	89.038	1.097	0.343	3.204	1.4E-03	1.5E-02
ENSMUSG00000062794	Zfp599	65.666	-1.239	0.387	-3.204	1.4E-03	1.5E-02

ENSMUSG00000046718	Bst2	28.671	1.928	0.602	3.202	1.4E-03	1.6E-02
ENSMUSG00000031314	Taf1	3268.161	0.261	0.082	3.201	1.4E-03	1.6E-02
ENSMUSG00000074457	S100a16	284.932	0.620	0.194	3.197	1.4E-03	1.6E-02
ENSMUSG00000022012	Enox1	1056.901	0.361	0.113	3.195	1.4E-03	1.6E-02
ENSMUSG00000051920	Rspo2	55.476	1.383	0.433	3.193	1.4E-03	1.6E-02
ENSMUSG00000053414	Hunk	554.629	-0.464	0.146	-3.191	1.4E-03	1.6E-02
ENSMUSG00000017561	Crlf3	1399.886	-0.337	0.106	-3.190	1.4E-03	1.6E-02
ENSMUSG00000039989	Cbx4	670.768	0.441	0.138	3.189	1.4E-03	1.6E-02
ENSMUSG00000053012	Krcc1	510.883	0.495	0.156	3.186	1.4E-03	1.6E-02
ENSMUSG00000109718	Trim61	19.981	2.405	0.755	3.185	1.4E-03	1.6E-02
ENSMUSG00000024335	Brd2	3358.940	0.263	0.083	3.182	1.5E-03	1.7E-02
ENSMUSG00000025157	Zdhhc16	848.739	0.385	0.121	3.180	1.5E-03	1.7E-02
ENSMUSG00000026374	Tsn	2118.469	0.307	0.096	3.179	1.5E-03	1.7E-02
ENSMUSG00000101939	Gm28438	7234.392	1.520	0.478	3.179	1.5E-03	1.7E-02
ENSMUSG00000004771	Rab11a	1304.435	0.337	0.106	3.178	1.5E-03	1.7E-02
ENSMUSG00000040652	Oaz2	1476.598	0.333	0.105	3.178	1.5E-03	1.7E-02
ENSMUSG00000022949	Clic6	151.386	-0.887	0.279	-3.176	1.5E-03	1.7E-02
ENSMUSG00000023723	Mrps23	310.072	0.592	0.186	3.175	1.5E-03	1.7E-02
ENSMUSG00000034109	Golim4	1318.000	0.347	0.109	3.174	1.5E-03	1.7E-02
ENSMUSG00000028692	Akr1a1	2574.444	0.292	0.092	3.173	1.5E-03	1.7E-02
ENSMUSG00000060568	Fam78b	1052.888	0.363	0.114	3.172	1.5E-03	1.7E-02
ENSMUSG00000025608	Podxl	2988.836	-0.261	0.082	-3.171	1.5E-03	1.7E-02
ENSMUSG00000015829	Tnr	1935.501	-0.336	0.106	-3.166	1.5E-03	1.7E-02
ENSMUSG00000022956	Atp5o	1289.607	0.353	0.112	3.166	1.5E-03	1.7E-02
ENSMUSG00000010476	Ebf3	498.149	-0.486	0.154	-3.165	1.5E-03	1.7E-02
ENSMUSG00000068742	Cry2	1335.715	-0.330	0.104	-3.164	1.6E-03	1.7E-02
ENSMUSG00000030000	Add2	1059.598	-0.350	0.111	-3.164	1.6E-03	1.7E-02
ENSMUSG00000061322	Dnai1	30.183	-2.112	0.668	-3.163	1.6E-03	1.7E-02
ENSMUSG00000022212	Cpne6	358.551	-0.544	0.172	-3.163	1.6E-03	1.7E-02
ENSMUSG00000001930	Vwf	92.308	1.112	0.352	3.161	1.6E-03	1.7E-02
ENSMUSG00000008153	Clstn3	1243.108	0.381	0.120	3.161	1.6E-03	1.7E-02
ENSMUSG00000032262	Elovl4	12638.092	-0.245	0.078	-3.159	1.6E-03	1.8E-02
ENSMUSG00000029123	Stk32b	61.184	-1.288	0.408	-3.158	1.6E-03	1.8E-02
ENSMUSG00000028524	Sgip1	6057.306	-0.254	0.080	-3.158	1.6E-03	1.8E-02
ENSMUSG00000030061	Uba3	1867.541	0.298	0.094	3.156	1.6E-03	1.8E-02
ENSMUSG00000091476	Catspere2	150.111	0.842	0.267	3.155	1.6E-03	1.8E-02
ENSMUSG00000033111	3830406C13Rik	463.596	0.559	0.178	3.152	1.6E-03	1.8E-02
ENSMUSG00000042121	Ssh1	821.404	-0.403	0.128	-3.150	1.6E-03	1.8E-02
ENSMUSG00000023852	Chd1	1872.808	-0.312	0.099	-3.148	1.6E-03	1.8E-02
ENSMUSG00000002346	Slc25a42	421.603	-0.520	0.165	-3.145	1.7E-03	1.8E-02
ENSMUSG00000053581	Zfand2a	529.502	0.504	0.160	3.145	1.7E-03	1.8E-02
ENSMUSG00000022756	Slc7a4	150.936	0.835	0.266	3.144	1.7E-03	1.8E-02
ENSMUSG00000032289	Thsd4	119.181	-1.035	0.329	-3.144	1.7E-03	1.8E-02
ENSMUSG00000001729	Akt1	1244.366	0.340	0.108	3.143	1.7E-03	1.8E-02
ENSMUSG00000049690	Nckap5	302.793	-0.725	0.231	-3.142	1.7E-03	1.9E-02
ENSMUSG00000033717	Adra2a	288.926	-0.626	0.199	-3.140	1.7E-03	1.9E-02
ENSMUSG00000059851	Kmt5c	655.578	-0.420	0.134	-3.140	1.7E-03	1.9E-02
ENSMUSG00000026415	Fcamr	61.956	1.368	0.436	3.139	1.7E-03	1.9E-02
ENSMUSG00000021109	Hif1a	15068.541	-0.210	0.067	-3.136	1.7E-03	1.9E-02
ENSMUSG00000033793	Atp6v1h	1805.844	0.305	0.097	3.135	1.7E-03	1.9E-02
ENSMUSG00000010663	Fads1	960.030	-0.360	0.115	-3.135	1.7E-03	1.9E-02
ENSMUSG00000025868	Higd2a	413.590	0.523	0.167	3.134	1.7E-03	1.9E-02
ENSMUSG00000038457	Tmem255b	23.281	-2.158	0.689	-3.132	1.7E-03	1.9E-02
ENSMUSG00000060261	Gtf2i	3474.738	0.266	0.085	3.131	1.7E-03	1.9E-02
ENSMUSG00000027630	Tbl1xr1	1003.078	0.357	0.114	3.130	1.7E-03	1.9E-02
ENSMUSG00000034467	Dynlrb2	14.548	2.931	0.938	3.124	1.8E-03	2.0E-02

ENSMUSG00000026730	Pter	252.789	0.685	0.219	3.123	1.8E-03	2.0E-02
ENSMUSG00000073158	9030624G23Rik	117.299	-0.917	0.294	-3.122	1.8E-03	2.0E-02
ENSMUSG00000000197	Nalcn	1360.793	0.329	0.105	3.121	1.8E-03	2.0E-02
ENSMUSG00000026407	Cacna1s	378.086	-0.603	0.193	-3.121	1.8E-03	2.0E-02
ENSMUSG00000024298	Zfp871	5698.113	-0.265	0.085	-3.117	1.8E-03	2.0E-02
ENSMUSG00000035885	Cox8a	1151.583	0.371	0.119	3.117	1.8E-03	2.0E-02
ENSMUSG00000027282	Mtch2	1043.043	0.349	0.112	3.116	1.8E-03	2.0E-02
ENSMUSG00000063919	Srrm4	2110.953	0.305	0.098	3.113	1.9E-03	2.0E-02
ENSMUSG00000061751	Kalrn	825.877	-0.467	0.150	-3.112	1.9E-03	2.0E-02
ENSMUSG00000016256	Ctsz	400.737	0.526	0.169	3.105	1.9E-03	2.1E-02
ENSMUSG00000025967	Eef1b2	969.694	0.398	0.128	3.105	1.9E-03	2.1E-02
ENSMUSG00000039347	Atp6v0e2	4140.612	-0.256	0.082	-3.104	1.9E-03	2.1E-02
ENSMUSG00000021684	Pde8b	1682.215	-0.337	0.109	-3.103	1.9E-03	2.1E-02
ENSMUSG00000031139	Mcf2	60.840	-1.263	0.407	-3.102	1.9E-03	2.1E-02
ENSMUSG00000030380	Mzf1	170.578	-0.746	0.240	-3.101	1.9E-03	2.1E-02
ENSMUSG00000034040	Galnt17	428.585	-0.495	0.160	-3.100	1.9E-03	2.1E-02
ENSMUSG00000033863	Klf9	2513.077	-0.279	0.090	-3.098	2.0E-03	2.1E-02
ENSMUSG00000042570	Mier2	530.462	-0.452	0.146	-3.097	2.0E-03	2.1E-02
ENSMUSG00000026491	Ahctf1	2426.824	-0.273	0.088	-3.095	2.0E-03	2.1E-02
ENSMUSG00000046836	Brox	1420.850	0.319	0.103	3.095	2.0E-03	2.1E-02
ENSMUSG00000054459	Vsnl1	1545.271	-0.309	0.100	-3.095	2.0E-03	2.1E-02
ENSMUSG00000020451	Limk2	1912.132	-0.289	0.093	-3.093	2.0E-03	2.1E-02
ENSMUSG00000022092	Ppp3cc	2348.833	-0.276	0.089	-3.092	2.0E-03	2.1E-02
ENSMUSG00000026773	Pfkfb3	468.067	-0.497	0.161	-3.092	2.0E-03	2.1E-02
ENSMUSG00000054134	Umodl1	17.943	-2.798	0.905	-3.091	2.0E-03	2.1E-02
ENSMUSG00000016481	Cr1l	254.750	0.659	0.213	3.090	2.0E-03	2.1E-02
ENSMUSG00000019774	Mtrf1l	139.851	0.821	0.266	3.090	2.0E-03	2.1E-02
ENSMUSG00000020926	Adam11	516.442	-0.478	0.155	-3.086	2.0E-03	2.2E-02
ENSMUSG00000038342	Mlxip	360.004	0.604	0.196	3.085	2.0E-03	2.2E-02
ENSMUSG00000052551	Adarb2	228.312	-0.717	0.232	-3.086	2.0E-03	2.2E-02
ENSMUSG00000000711	Rab5b	2575.242	0.259	0.084	3.083	2.0E-03	2.2E-02
ENSMUSG00000032030	Cul5	3189.058	-0.248	0.080	-3.083	2.0E-03	2.2E-02
ENSMUSG00000069237	Fam8a1	1790.240	0.305	0.099	3.082	2.1E-03	2.2E-02
ENSMUSG00000019923	Zwint	5906.191	0.248	0.081	3.082	2.1E-03	2.2E-02
ENSMUSG00000040478	Prdm13	163.131	-0.791	0.257	-3.082	2.1E-03	2.2E-02
ENSMUSG00000052557	Gan	793.575	-0.426	0.139	-3.077	2.1E-03	2.2E-02
ENSMUSG00000037344	Slc12a9	298.045	-0.609	0.198	-3.077	2.1E-03	2.2E-02
ENSMUSG00000038762	Abcf1	1552.074	-0.316	0.103	-3.076	2.1E-03	2.2E-02
ENSMUSG00000042763	Maneal	1116.905	-0.351	0.114	-3.075	2.1E-03	2.2E-02
ENSMUSG00000034706	Dnai2	126.103	0.913	0.297	3.073	2.1E-03	2.2E-02
ENSMUSG00000021754	Map3k1	1406.242	0.361	0.118	3.072	2.1E-03	2.3E-02
ENSMUSG00000024242	Map4k3	3623.588	-0.242	0.079	-3.071	2.1E-03	2.3E-02
ENSMUSG00000031645	F11	26.430	-1.875	0.610	-3.071	2.1E-03	2.3E-02
ENSMUSG00000040859	Bsdc1	620.931	0.446	0.145	3.071	2.1E-03	2.3E-02
ENSMUSG00000036334	Igsf10	85.439	1.063	0.346	3.068	2.2E-03	2.3E-02
ENSMUSG00000036026	Tmem63b	1693.647	-0.289	0.094	-3.067	2.2E-03	2.3E-02
ENSMUSG00000056553	Ptprn2	902.258	0.363	0.119	3.064	2.2E-03	2.3E-02
ENSMUSG00000004366	Sst	17.542	-2.357	0.770	-3.063	2.2E-03	2.3E-02
ENSMUSG00000037818	Abhd18	898.027	-0.377	0.123	-3.062	2.2E-03	2.3E-02
ENSMUSG00000004085	Map3k20	116.355	1.173	0.383	3.062	2.2E-03	2.3E-02
ENSMUSG00000004113	Cacna1b	570.542	0.434	0.142	3.061	2.2E-03	2.3E-02
ENSMUSG00000096808		97.750	-0.980	0.321	-3.058	2.2E-03	2.3E-02
ENSMUSG00000031622	Sin3b	1075.468	0.354	0.116	3.057	2.2E-03	2.4E-02
ENSMUSG00000040938	Slc16a11	40.684	-1.494	0.489	-3.056	2.2E-03	2.4E-02
ENSMUSG00000022261	Sdc2	1046.444	0.379	0.124	3.054	2.3E-03	2.4E-02
ENSMUSG00000020140	Lgr5	620.524	-0.478	0.157	-3.053	2.3E-03	2.4E-02

ENSMUSG00000110170	St6galnac2	179.005	-0.741	0.243	-3.053	2.3E-03	2.4E-02
ENSMUSG00000034333	Zbed4	698.882	-0.403	0.132	-3.052	2.3E-03	2.4E-02
ENSMUSG00000110444	Gm10033	629.519	-0.460	0.151	-3.051	2.3E-03	2.4E-02
ENSMUSG00000004668	Abca13	357.433	-0.582	0.191	-3.043	2.3E-03	2.4E-02
ENSMUSG00000020917	Acly	3657.834	-0.241	0.079	-3.043	2.3E-03	2.4E-02
ENSMUSG00000022604	Cep97	871.308	0.406	0.133	3.044	2.3E-03	2.4E-02
ENSMUSG00000078515	Ddi2	2314.755	0.271	0.089	3.043	2.3E-03	2.4E-02
ENSMUSG00000023022	Lima1	2205.970	0.279	0.092	3.043	2.3E-03	2.4E-02
ENSMUSG00000031144	Syp	14467.166	0.197	0.065	3.041	2.4E-03	2.5E-02
ENSMUSG00000025202	Scd3	104.301	0.984	0.324	3.040	2.4E-03	2.5E-02
ENSMUSG00000032932	Hspa13	1163.412	0.346	0.114	3.040	2.4E-03	2.5E-02
ENSMUSG00000033105	Lss	1063.820	-0.353	0.116	-3.040	2.4E-03	2.5E-02
ENSMUSG00000046994	Mars2	934.972	-0.370	0.122	-3.040	2.4E-03	2.5E-02
ENSMUSG00000045532	C1ql1	346.841	-0.562	0.185	-3.039	2.4E-03	2.5E-02
ENSMUSG00000059336	Slc14a1	370.207	0.568	0.187	3.038	2.4E-03	2.5E-02
ENSMUSG00000034868	Myl12b	1188.753	0.341	0.112	3.038	2.4E-03	2.5E-02
ENSMUSG00000048148	Nwd1	403.808	-0.517	0.170	-3.034	2.4E-03	2.5E-02
ENSMUSG00000015143	Actn1	620.300	0.427	0.141	3.034	2.4E-03	2.5E-02
ENSMUSG00000078490	Cfap74	256.791	0.607	0.200	3.032	2.4E-03	2.5E-02
ENSMUSG00000041992	Rapgef5	4958.903	-0.222	0.073	-3.031	2.4E-03	2.5E-02
ENSMUSG00000021748	Pdhb	2378.945	0.268	0.088	3.029	2.5E-03	2.5E-02
ENSMUSG00000030107	Usp18	29.750	1.940	0.641	3.028	2.5E-03	2.5E-02
ENSMUSG00000058318	Phf21a	943.207	0.361	0.119	3.028	2.5E-03	2.5E-02
ENSMUSG00000032715	Trib3	57.494	-1.280	0.423	-3.025	2.5E-03	2.6E-02
ENSMUSG00000107928	Gm45140	202.710	0.915	0.303	3.017	2.5E-03	2.6E-02
ENSMUSG00000113976	Gm18243	9.419	3.863	1.280	3.017	2.5E-03	2.6E-02
ENSMUSG00000036580	Spg20	663.014	0.415	0.138	3.017	2.6E-03	2.6E-02
ENSMUSG00000045427	Hnrnph2	3668.653	0.242	0.080	3.017	2.6E-03	2.6E-02
ENSMUSG00000058392	Rrp1b	1829.021	-0.313	0.104	-3.016	2.6E-03	2.6E-02
ENSMUSG00000028127	Abcd3	1431.444	0.319	0.106	3.016	2.6E-03	2.6E-02
ENSMUSG00000042208	Sanbr	857.482	0.384	0.127	3.014	2.6E-03	2.6E-02
ENSMUSG00000003184	Irf3	647.858	-0.411	0.136	-3.013	2.6E-03	2.7E-02
ENSMUSG00000025645	Ccdc51	205.694	-0.667	0.221	-3.012	2.6E-03	2.7E-02
ENSMUSG00000013622	Atraid	776.148	0.395	0.131	3.012	2.6E-03	2.7E-02
ENSMUSG00000039000	Ube3c	1873.275	0.283	0.094	3.009	2.6E-03	2.7E-02
ENSMUSG00000039671	Zmynd8	3007.068	-0.245	0.081	-3.009	2.6E-03	2.7E-02
ENSMUSG00000057329	Bcl2	491.965	-0.456	0.152	-3.007	2.6E-03	2.7E-02
ENSMUSG00000042429	Adora1	742.172	-0.398	0.133	-3.002	2.7E-03	2.7E-02
ENSMUSG00000037197	Rbm17	1007.722	0.339	0.113	3.001	2.7E-03	2.7E-02
ENSMUSG00000049502	Dtx3l	138.035	0.831	0.277	2.998	2.7E-03	2.8E-02
ENSMUSG00000031539	Ap3m2	2278.799	0.266	0.089	2.998	2.7E-03	2.8E-02
ENSMUSG00000079659	Tmem243	737.229	0.411	0.137	2.995	2.7E-03	2.8E-02
ENSMUSG00000029992	Gfpt1	2667.727	-0.262	0.088	-2.991	2.8E-03	2.8E-02
ENSMUSG00000042109	Csdc2	540.890	-0.429	0.144	-2.991	2.8E-03	2.8E-02
ENSMUSG00000042320	Prox2	63.016	-1.176	0.393	-2.990	2.8E-03	2.8E-02
ENSMUSG00000016346	Kcnq2	1451.287	-0.346	0.116	-2.988	2.8E-03	2.9E-02
ENSMUSG00000036478	Btg1	1715.799	0.296	0.099	2.988	2.8E-03	2.9E-02
ENSMUSG00000034450	Gulo	283.213	0.587	0.197	2.987	2.8E-03	2.9E-02
ENSMUSG00000021400	Wrnip1	826.670	0.371	0.124	2.987	2.8E-03	2.9E-02
ENSMUSG00000014846	Tppp3	513.017	-0.454	0.152	-2.985	2.8E-03	2.9E-02
ENSMUSG00000029862	Clcn1	99.745	-0.948	0.318	-2.985	2.8E-03	2.9E-02
ENSMUSG00000070498	Tmem132b	357.759	0.612	0.205	2.985	2.8E-03	2.9E-02
ENSMUSG00000000740	Rpl13	2936.549	0.302	0.101	2.984	2.8E-03	2.9E-02
ENSMUSG00000044934	Zfp367	614.858	0.414	0.139	2.984	2.8E-03	2.9E-02
ENSMUSG00000003660	Snrnp200	4311.995	-0.243	0.082	-2.983	2.9E-03	2.9E-02
ENSMUSG00000050545	Fam228b	115.841	-1.014	0.341	-2.977	2.9E-03	2.9E-02

ENSMUSG00000003923	Tfam	515.994	0.492	0.165	2.974	2.9E-03	3.0E-02
ENSMUSG00000029763	Exoc4	1259.861	0.339	0.114	2.974	2.9E-03	3.0E-02
ENSMUSG00000064105	Cnnm2	597.010	0.441	0.148	2.973	2.9E-03	3.0E-02
ENSMUSG00000041329	Atp1b2	18980.113	0.188	0.063	2.972	3.0E-03	3.0E-02
ENSMUSG00000021604	Irx4	26.424	-1.834	0.617	-2.971	3.0E-03	3.0E-02
ENSMUSG00000056536	Pign	1105.471	0.362	0.122	2.968	3.0E-03	3.0E-02
ENSMUSG00000020807	4933427D14Rik	336.614	0.583	0.196	2.966	3.0E-03	3.0E-02
ENSMUSG00000048349	Pou4f1	236.707	-0.624	0.210	-2.966	3.0E-03	3.0E-02
ENSMUSG00000037110	Ralgapa2	1110.493	-0.393	0.133	-2.964	3.0E-03	3.1E-02
ENSMUSG00000030850	Ate1	2867.204	0.255	0.086	2.961	3.1E-03	3.1E-02
ENSMUSG00000061740	Cyp2d22	297.735	-0.553	0.187	-2.961	3.1E-03	3.1E-02
ENSMUSG00000000628	Hk2	5369.231	-0.258	0.087	-2.960	3.1E-03	3.1E-02
ENSMUSG00000015647	Lama5	226.344	0.703	0.237	2.960	3.1E-03	3.1E-02
ENSMUSG00000025400	Tac2	79.651	-1.029	0.348	-2.959	3.1E-03	3.1E-02
ENSMUSG00000026670	Uap1	1166.288	-0.354	0.120	-2.957	3.1E-03	3.1E-02
ENSMUSG00000007415	Gatad1	879.263	0.364	0.123	2.957	3.1E-03	3.1E-02
ENSMUSG00000030337	Vamp1	1173.880	-0.341	0.115	-2.957	3.1E-03	3.1E-02
ENSMUSG00000034867	Ankrd27	1009.672	-0.382	0.129	-2.957	3.1E-03	3.1E-02
ENSMUSG00000023886	Smoc2	28.707	-1.884	0.638	-2.955	3.1E-03	3.1E-02
ENSMUSG00000027849	Syt6	182.768	-0.702	0.238	-2.954	3.1E-03	3.1E-02
ENSMUSG00000026594	Ralgps2	2272.209	0.318	0.108	2.954	3.1E-03	3.1E-02
ENSMUSG00000071454	Dtnb	472.641	-0.477	0.161	-2.953	3.1E-03	3.1E-02
ENSMUSG00000036698	ago-02	2627.952	-0.283	0.096	-2.952	3.2E-03	3.1E-02
ENSMUSG00000032601	Prkar2a	2515.355	-0.249	0.084	-2.952	3.2E-03	3.1E-02
ENSMUSG00000019699	Akt3	3423.030	-0.237	0.080	-2.951	3.2E-03	3.1E-02
ENSMUSG00000041147	Brca2	308.394	-0.540	0.183	-2.947	3.2E-03	3.2E-02
ENSMUSG00000021335	Slc17a1	46.492	-1.344	0.456	-2.946	3.2E-03	3.2E-02
ENSMUSG00000029063	Nadk	507.424	0.436	0.148	2.944	3.2E-03	3.2E-02
ENSMUSG00000041360	Pum3	562.363	0.499	0.169	2.944	3.2E-03	3.2E-02
ENSMUSG00000021884	Hacl1	226.904	-0.670	0.228	-2.943	3.2E-03	3.2E-02
ENSMUSG00000009470	Tnpo1	12904.221	-0.211	0.072	-2.941	3.3E-03	3.2E-02
ENSMUSG00000027378	Nphp1	487.252	0.452	0.154	2.939	3.3E-03	3.3E-02
ENSMUSG00000062203	Gspt1	2258.503	0.291	0.099	2.939	3.3E-03	3.3E-02
ENSMUSG00000056579	Tug1	3907.744	-0.232	0.079	-2.939	3.3E-03	3.3E-02
ENSMUSG00000020782	Llgl2	844.245	-0.397	0.135	-2.933	3.4E-03	3.3E-02
ENSMUSG00000032123	Dpagt1	179.144	0.720	0.245	2.934	3.4E-03	3.3E-02
ENSMUSG00000038208	Pgap3	456.627	0.459	0.157	2.933	3.4E-03	3.3E-02
ENSMUSG00000036928	Stag3	141.958	-0.773	0.264	-2.932	3.4E-03	3.3E-02
ENSMUSG00000051469	Zfp24	2554.506	0.262	0.089	2.932	3.4E-03	3.3E-02
ENSMUSG00000020982	Nemf	2517.275	-0.260	0.089	-2.930	3.4E-03	3.3E-02
ENSMUSG00000082090	Gm16481	10.902	-3.488	1.191	-2.928	3.4E-03	3.3E-02
ENSMUSG00000034263	Ints14	572.514	0.415	0.142	2.926	3.4E-03	3.4E-02
ENSMUSG00000021703	Serinc5	787.031	0.395	0.135	2.925	3.4E-03	3.4E-02
ENSMUSG00000020857	Nme2	199.726	0.660	0.226	2.925	3.4E-03	3.4E-02
ENSMUSG00000039375	Wdr17	7205.374	-0.217	0.074	-2.923	3.5E-03	3.4E-02
ENSMUSG00000021665	Hexb	487.856	0.438	0.150	2.921	3.5E-03	3.4E-02
ENSMUSG00000027381	Bcl2l11	297.914	-0.578	0.198	-2.920	3.5E-03	3.4E-02
ENSMUSG00000059729	Olfr1385	141.094	-0.783	0.268	-2.920	3.5E-03	3.4E-02
ENSMUSG00000109764	Klkb1	122.443	-0.854	0.293	-2.920	3.5E-03	3.4E-02
ENSMUSG00000004364	Cul3	6556.206	-0.223	0.076	-2.919	3.5E-03	3.4E-02
ENSMUSG00000012405	Rpl15	2895.075	0.279	0.096	2.917	3.5E-03	3.4E-02
ENSMUSG00000028274	Rngtt	717.611	0.454	0.156	2.917	3.5E-03	3.4E-02
ENSMUSG00000035594	Chrna5	166.958	0.734	0.252	2.915	3.6E-03	3.5E-02
ENSMUSG00000067578	Cbln4	72.183	-1.070	0.367	-2.916	3.6E-03	3.5E-02
ENSMUSG00000029798	Herc6	149.639	0.773	0.265	2.913	3.6E-03	3.5E-02
ENSMUSG00000039263	Npepl1	155.373	0.782	0.268	2.911	3.6E-03	3.5E-02

ENSMUSG00000026315	Serpinb8	21.033	-2.035	0.700	-2.909	3.6E-03	3.5E-02
ENSMUSG00000030452	Nipa2	659.660	0.395	0.136	2.907	3.6E-03	3.5E-02
ENSMUSG00000056812	St8sia3	1033.909	0.325	0.112	2.906	3.7E-03	3.6E-02
ENSMUSG00000021239	Vsx2	2322.995	-0.279	0.096	-2.905	3.7E-03	3.6E-02
ENSMUSG00000016409	Nkap	432.244	0.513	0.177	2.904	3.7E-03	3.6E-02
ENSMUSG00000034799	Unc13a	1807.034	-0.283	0.097	-2.904	3.7E-03	3.6E-02
ENSMUSG00000030806	Stx1b	2033.288	-0.264	0.091	-2.902	3.7E-03	3.6E-02
ENSMUSG00000037736	Limch1	1796.193	0.787	0.271	2.901	3.7E-03	3.6E-02
ENSMUSG00000055733	Nap1l3	513.772	0.436	0.150	2.901	3.7E-03	3.6E-02
ENSMUSG00000038127	Ccdc50	1511.351	0.286	0.099	2.898	3.8E-03	3.6E-02
ENSMUSG00000057666	Gapdh	44234.270	-0.188	0.065	-2.898	3.8E-03	3.6E-02
ENSMUSG00000029505	Ep400	1932.657	-0.272	0.094	-2.897	3.8E-03	3.6E-02
ENSMUSG00000028759	Hp1bp3	4655.053	0.216	0.074	2.897	3.8E-03	3.6E-02
ENSMUSG00000075254	Heg1	3195.190	0.308	0.106	2.896	3.8E-03	3.6E-02
ENSMUSG00000025372	Baiap2	144.533	-0.762	0.263	-2.895	3.8E-03	3.6E-02
ENSMUSG00000036905	C1qb	72.420	1.084	0.374	2.895	3.8E-03	3.6E-02
ENSMUSG00000000131	Xpo6	1844.740	-0.267	0.092	-2.894	3.8E-03	3.7E-02
ENSMUSG00000055531	Cpsf6	4413.431	-0.230	0.079	-2.891	3.8E-03	3.7E-02
ENSMUSG00000030760	Acer3	509.282	-0.443	0.153	-2.891	3.8E-03	3.7E-02
ENSMUSG00000041355	Ssr2	905.478	0.348	0.120	2.888	3.9E-03	3.7E-02
ENSMUSG00000048347	Pcdhb18	386.880	0.481	0.166	2.888	3.9E-03	3.7E-02
ENSMUSG00000021379	Id4	602.083	-0.414	0.143	-2.887	3.9E-03	3.7E-02
ENSMUSG00000030862	Cpxm2	65.213	1.138	0.394	2.887	3.9E-03	3.7E-02
ENSMUSG00000068206	Pick1	595.708	0.399	0.138	2.886	3.9E-03	3.7E-02
ENSMUSG00000000420	Galnt1	1529.383	0.282	0.098	2.881	4.0E-03	3.8E-02
ENSMUSG00000001773	Folh1	274.762	-0.607	0.211	-2.879	4.0E-03	3.8E-02
ENSMUSG00000021982	Cdadc1	1109.867	0.338	0.117	2.880	4.0E-03	3.8E-02
ENSMUSG00000028826	Maco1	1849.953	-0.276	0.096	-2.879	4.0E-03	3.8E-02
ENSMUSG00000090100	Ttbk2	2935.449	-0.240	0.083	-2.880	4.0E-03	3.8E-02
ENSMUSG00000097929	Tunar	181.045	-0.692	0.240	-2.879	4.0E-03	3.8E-02
ENSMUSG00000022811	Zfp148	3102.726	0.232	0.081	2.877	4.0E-03	3.8E-02
ENSMUSG00000021756	Il6st	2508.095	0.276	0.096	2.875	4.0E-03	3.8E-02
ENSMUSG00000020704	Asic2	312.336	-0.524	0.182	-2.875	4.0E-03	3.8E-02
ENSMUSG00000041112	Elmo1	872.111	-0.348	0.121	-2.874	4.0E-03	3.8E-02
ENSMUSG00000044461	Shisa2	321.372	0.569	0.198	2.872	4.1E-03	3.9E-02
ENSMUSG00000028926	Cdk14	2418.492	-0.245	0.085	-2.871	4.1E-03	3.9E-02
ENSMUSG00000032551	1110059G10Rik	371.818	0.506	0.176	2.871	4.1E-03	3.9E-02
ENSMUSG00000027014	Cwc22	794.172	-0.360	0.125	-2.870	4.1E-03	3.9E-02
ENSMUSG00000021222	Dcaf4	367.993	0.537	0.187	2.869	4.1E-03	3.9E-02
ENSMUSG00000021608	Lpcat1	2506.808	-0.249	0.087	-2.869	4.1E-03	3.9E-02
ENSMUSG00000032172	Olfm2	253.673	0.584	0.203	2.869	4.1E-03	3.9E-02
ENSMUSG00000048832	Vps37c	596.841	-0.422	0.147	-2.869	4.1E-03	3.9E-02
ENSMUSG00000022519	Srl	105.853	1.027	0.358	2.867	4.2E-03	3.9E-02
ENSMUSG00000069911	Insyn2b	161.954	-0.798	0.279	-2.865	4.2E-03	3.9E-02
ENSMUSG00000031442	Mcf2l	2268.776	-0.282	0.099	-2.862	4.2E-03	4.0E-02
ENSMUSG00000039057	Myo16	187.814	-0.662	0.231	-2.862	4.2E-03	4.0E-02
ENSMUSG00000036646	Man1b1	1148.597	0.330	0.115	2.861	4.2E-03	4.0E-02
ENSMUSG00000113146	Gm47406	166.790	-0.720	0.252	-2.861	4.2E-03	4.0E-02
ENSMUSG00000028438	Kif24	74.872	-1.044	0.365	-2.859	4.3E-03	4.0E-02
ENSMUSG00000032382	Snx1	1097.246	0.314	0.110	2.859	4.3E-03	4.0E-02
ENSMUSG00000015981	Stk32c	133.467	-0.789	0.276	-2.858	4.3E-03	4.0E-02
ENSMUSG00000039199	Zdhhc1	390.208	0.491	0.172	2.857	4.3E-03	4.0E-02
ENSMUSG00000061904	Slc25a3	3342.884	0.226	0.079	2.857	4.3E-03	4.0E-02
ENSMUSG00000034780	B3galt1	751.688	0.382	0.134	2.857	4.3E-03	4.0E-02
ENSMUSG00000025064	Col17a1	45.293	-1.437	0.503	-2.855	4.3E-03	4.0E-02
ENSMUSG00000054793	Cadm4	510.529	-0.426	0.149	-2.855	4.3E-03	4.0E-02

ENSMUSG00000078429	Ctdsp2	1100.791	0.322	0.113	2.855	4.3E-03	4.0E-02
ENSMUSG00000033355	Rtp4	30.780	1.672	0.586	2.855	4.3E-03	4.0E-02
ENSMUSG00000038213	Tapbpl	85.248	1.007	0.353	2.853	4.3E-03	4.0E-02
ENSMUSG00000035649	Zcchc7	2588.885	-0.244	0.085	-2.852	4.3E-03	4.1E-02
ENSMUSG00000111163	Gm46136	99.363	0.941	0.331	2.846	4.4E-03	4.1E-02
ENSMUSG00000038451	Spsb2	37.124	1.513	0.532	2.846	4.4E-03	4.1E-02
ENSMUSG00000109378	Gm49396	289.550	-0.536	0.188	-2.845	4.4E-03	4.1E-02
ENSMUSG00000034173	Zbed5	64.807	1.142	0.402	2.844	4.5E-03	4.2E-02
ENSMUSG00000051056	Gja10	45.405	-1.356	0.477	-2.844	4.5E-03	4.2E-02
ENSMUSG00000080893	Ndufa12-ps	19.198	-2.065	0.726	-2.844	4.5E-03	4.2E-02
ENSMUSG00000034783	Cd207	10.296	-2.991	1.052	-2.843	4.5E-03	4.2E-02
ENSMUSG00000031239	Itm2a	237.304	0.597	0.210	2.842	4.5E-03	4.2E-02
ENSMUSG00000000948	Gm38393	949.602	-0.365	0.128	-2.841	4.5E-03	4.2E-02
ENSMUSG00000001942	Siae	182.510	0.678	0.239	2.841	4.5E-03	4.2E-02
ENSMUSG00000025938	Slco5a1	467.022	-0.474	0.167	-2.839	4.5E-03	4.2E-02
ENSMUSG00000080316	Spaca6	393.838	0.492	0.173	2.838	4.5E-03	4.2E-02
ENSMUSG00000018604	Tbx3	157.563	-0.729	0.257	-2.836	4.6E-03	4.2E-02
ENSMUSG00000028525	Pde4b	1225.838	-0.303	0.107	-2.836	4.6E-03	4.2E-02
ENSMUSG00000052397	Ezr	228.267	0.616	0.217	2.836	4.6E-03	4.2E-02
ENSMUSG00000028541	B4galt2	861.201	-0.344	0.121	-2.835	4.6E-03	4.2E-02
ENSMUSG00000019984	Med23	1245.522	0.301	0.106	2.834	4.6E-03	4.2E-02
ENSMUSG00000040170	Fmo2	26.507	2.270	0.801	2.834	4.6E-03	4.2E-02
ENSMUSG00000034107	Ano7	13.278	-2.799	0.988	-2.834	4.6E-03	4.2E-02
ENSMUSG00000096764	Gm21985	116.668	1.113	0.393	2.833	4.6E-03	4.3E-02
ENSMUSG00000039046	Usp6nl	2621.783	-0.239	0.085	-2.828	4.7E-03	4.3E-02
ENSMUSG00000052373	Mpp3	235.609	-0.643	0.228	-2.827	4.7E-03	4.3E-02
ENSMUSG00000052912	Smarca5-ps	114.923	-0.822	0.291	-2.827	4.7E-03	4.3E-02
ENSMUSG00000001025	S100a6	59.969	1.168	0.413	2.825	4.7E-03	4.3E-02
ENSMUSG00000017314	Mpp2	945.192	-0.346	0.123	-2.825	4.7E-03	4.4E-02
ENSMUSG00000017548	Suz12	3499.902	-0.227	0.081	-2.823	4.8E-03	4.4E-02
ENSMUSG00000048988	Elfn1	1146.732	-0.315	0.112	-2.823	4.8E-03	4.4E-02
ENSMUSG00000049940	Pgrmc2	1231.534	0.296	0.105	2.821	4.8E-03	4.4E-02
ENSMUSG00000043822	Adamtsl5	131.984	-0.767	0.272	-2.819	4.8E-03	4.4E-02
ENSMUSG00000024766	Lipo3	405.649	0.478	0.170	2.818	4.8E-03	4.4E-02
ENSMUSG00000053119	Chmp3	1284.899	0.308	0.109	2.818	4.8E-03	4.4E-02
ENSMUSG00000052632	Asap2	909.436	-0.339	0.120	-2.817	4.8E-03	4.4E-02
ENSMUSG00000026553	Copa	5292.765	0.215	0.076	2.815	4.9E-03	4.5E-02
ENSMUSG00000113889	Gm48501	155.586	-0.769	0.273	-2.815	4.9E-03	4.5E-02
ENSMUSG00000021428	Riok1	621.722	0.395	0.140	2.815	4.9E-03	4.5E-02
ENSMUSG00000026798	Coq4	90.966	0.979	0.348	2.815	4.9E-03	4.5E-02
ENSMUSG00000038145	Snrk	2169.961	-0.263	0.094	-2.813	4.9E-03	4.5E-02
ENSMUSG00000038936	Sccpdh	1087.849	0.331	0.118	2.813	4.9E-03	4.5E-02
ENSMUSG00000026596	Abl2	1464.715	-0.297	0.106	-2.812	4.9E-03	4.5E-02
ENSMUSG00000035161	Ints6	1050.970	0.327	0.116	2.811	4.9E-03	4.5E-02
ENSMUSG00000035901	Dennd5a	4008.700	0.218	0.078	2.810	5.0E-03	4.5E-02
ENSMUSG00000031109	Enox2	281.856	0.562	0.200	2.809	5.0E-03	4.5E-02
ENSMUSG00000055782	Abcd2	1450.845	0.320	0.114	2.807	5.0E-03	4.5E-02
ENSMUSG00000025968	Ndufs1	2618.486	0.257	0.092	2.806	5.0E-03	4.6E-02
ENSMUSG00000025240	Sacm1l	1705.933	0.284	0.101	2.805	5.0E-03	4.6E-02
ENSMUSG00000034912	Mdga2	1252.576	-0.346	0.123	-2.805	5.0E-03	4.6E-02
ENSMUSG00000014075	Dynlt2b	303.492	-0.523	0.187	-2.804	5.1E-03	4.6E-02
ENSMUSG00000026527	Rgs7	949.362	-0.350	0.125	-2.803	5.1E-03	4.6E-02
ENSMUSG00000029267	Mtf2	1386.239	0.288	0.103	2.801	5.1E-03	4.6E-02
ENSMUSG00000034342	Cbl	1549.636	-0.345	0.123	-2.801	5.1E-03	4.6E-02
ENSMUSG00000021820	Camk2g	1693.293	0.288	0.103	2.799	5.1E-03	4.6E-02
ENSMUSG00000054808	Actn4	1866.808	-0.292	0.104	-2.798	5.1E-03	4.6E-02

ENSMUSG00000014313	Cox6c	2033.839	0.276	0.099	2.798	5.1E-03	4.6E-02
ENSMUSG00000031828	Klhl36	439.508	-0.444	0.159	-2.798	5.1E-03	4.6E-02
ENSMUSG00000038187	Btbd10	1061.186	0.310	0.111	2.795	5.2E-03	4.7E-02
ENSMUSG00000072969	Armcx5	374.225	0.473	0.169	2.795	5.2E-03	4.7E-02
ENSMUSG00000019877	Serinc1	12037.024	0.200	0.071	2.793	5.2E-03	4.7E-02
ENSMUSG00000047735	Samd9l	136.200	0.760	0.272	2.792	5.2E-03	4.7E-02
ENSMUSG00000020130	Tbc1d15	1056.458	0.397	0.142	2.791	5.3E-03	4.7E-02
ENSMUSG00000039145	Camk1d	3967.503	-0.214	0.076	-2.791	5.3E-03	4.7E-02
ENSMUSG00000073940	Hbb-bt	35.680	-1.471	0.527	-2.791	5.3E-03	4.7E-02
ENSMUSG00000020770	Unk	495.795	-0.441	0.158	-2.791	5.3E-03	4.7E-02
ENSMUSG00000036751	Cox6b1	1092.588	0.361	0.129	2.790	5.3E-03	4.7E-02
ENSMUSG00000026442	Nfasc	5972.667	-0.222	0.079	-2.789	5.3E-03	4.7E-02
ENSMUSG00000042363	Lgalsl	6397.311	-0.212	0.076	-2.787	5.3E-03	4.8E-02
ENSMUSG00000049176	Frmpd4	228.996	-0.607	0.218	-2.787	5.3E-03	4.8E-02
ENSMUSG00000032564	Cpne4	191.530	-0.641	0.230	-2.786	5.3E-03	4.8E-02
ENSMUSG00000030505	Prmt3	315.091	0.522	0.187	2.785	5.4E-03	4.8E-02
ENSMUSG00000072964	Bhlhb9	1082.902	0.305	0.110	2.784	5.4E-03	4.8E-02
ENSMUSG00000031024	Denn2b	302.489	-0.547	0.196	-2.783	5.4E-03	4.8E-02
ENSMUSG00000038619	Ensa	3602.902	0.224	0.081	2.780	5.4E-03	4.9E-02
ENSMUSG00000066189	Cacng3	358.708	-0.542	0.195	-2.778	5.5E-03	4.9E-02
ENSMUSG00000108423	Gm44856	28.489	-1.651	0.595	-2.777	5.5E-03	4.9E-02
ENSMUSG00000020131	Pcsk4	105.022	-0.842	0.303	-2.777	5.5E-03	4.9E-02
ENSMUSG00000035711	Dok3	73.136	-1.110	0.400	-2.777	5.5E-03	4.9E-02
ENSMUSG00000040270	Bach2	214.418	0.738	0.266	2.775	5.5E-03	4.9E-02
ENSMUSG00000006576	Slc4a3	1092.592	-0.338	0.122	-2.774	5.5E-03	4.9E-02
ENSMUSG00000047881	Rell1	177.310	0.711	0.256	2.775	5.5E-03	4.9E-02
ENSMUSG00000079057	Cyp4v3	260.417	-0.560	0.202	-2.774	5.5E-03	4.9E-02
ENSMUSG00000003721	Insig2	926.961	0.334	0.121	2.774	5.5E-03	4.9E-02
ENSMUSG00000005087	Cd44	307.991	0.552	0.199	2.774	5.5E-03	4.9E-02
ENSMUSG00000021510	Zfp729a	1155.935	-0.340	0.123	-2.771	5.6E-03	5.0E-02
ENSMUSG00000062591	Tubb4a	1465.129	-0.281	0.102	-2.771	5.6E-03	5.0E-02
ENSMUSG00000027636	Sla2	13.489	-2.784	1.005	-2.770	5.6E-03	5.0E-02
ENSMUSG00000031818	Cox4i1	2319.340	0.273	0.098	2.770	5.6E-03	5.0E-02

		Estriado					
Ensembl Gene ID	Gene symbol	baseMean	log2FoldChange	lfcSE	stat	pvalue	padj
ENSMUSG00000046480	Scn4b	8313.326	-2.553	0.111	-23.078	7.7E-118	1.1E-113
ENSMUSG00000045573	Penk	8106.515	-1.978	0.111	-17.871	2.0E-71	1.4E-67
ENSMUSG00000007656	Arpp19	6365.376	-1.428	0.086	-16.687	1.6E-62	7.5E-59
ENSMUSG00000038530	Rgs4	7834.600	-1.475	0.096	-15.440	8.8E-54	3.0E-50
ENSMUSG00000059213	Ddn	12021.171	-1.235	0.092	-13.384	7.5E-41	2.1E-37
ENSMUSG00000020178	Adora2a	1946.453	-1.637	0.123	-13.305	2.2E-40	5.0E-37
ENSMUSG00000032503	Arpp21	16267.790	-1.353	0.102	-13.260	4.0E-40	7.8E-37
ENSMUSG00000022840	Adcy5	11137.291	-1.164	0.092	-12.629	1.5E-36	2.5E-33
ENSMUSG00000090223	Pcp4	4885.937	-1.248	0.102	-12.182	3.9E-34	5.9E-31
ENSMUSG00000046447	Camk2n1	23892.639	-1.158	0.096	-12.119	8.4E-34	1.2E-30
ENSMUSG00000030592	Ryr1	500.128	-1.971	0.163	-12.103	1.0E-33	1.3E-30
ENSMUSG00000032373	Car12	575.697	-1.971	0.167	-11.832	2.7E-32	3.1E-29
ENSMUSG00000020599	Rgs9	4522.182	-1.431	0.124	-11.514	1.1E-30	1.2E-27
ENSMUSG00000021478	Drd1	2842.281	-1.284	0.112	-11.442	2.6E-30	2.6E-27
ENSMUSG00000007617	Homer1	4147.070	-1.013	0.089	-11.421	3.3E-30	3.0E-27
ENSMUSG00000046182	Gsg1l	1285.369	-1.530	0.136	-11.289	1.5E-29	1.3E-26
ENSMUSG00000090291	Lrrc10b	1316.733	-1.746	0.155	-11.257	2.1E-29	1.7E-26
ENSMUSG00000037679	Inf2	3407.337	-1.102	0.099	-11.121	9.9E-29	7.6E-26
ENSMUSG00000046818	Ddit4l	264.462	-2.384	0.217	-11.006	3.6E-28	2.6E-25
ENSMUSG00000052889	Prkcb	9848.955	-0.949	0.087	-10.971	5.3E-28	3.6E-25
ENSMUSG00000025738	Fbxl16	12147.087	-0.962	0.088	-10.908	1.1E-27	6.9E-25
ENSMUSG00000028161	Ppp3ca	35616.111	-0.858	0.080	-10.763	5.2E-27	3.2E-24
ENSMUSG00000020953	Coch	716.962	-1.579	0.147	-10.753	5.8E-27	3.5E-24
ENSMUSG00000074968	Ano3	5846.305	-1.082	0.101	-10.747	6.2E-27	3.5E-24
ENSMUSG00000028785	Hpca	10442.904	-1.047	0.098	-10.722	8.0E-27	4.4E-24
ENSMUSG00000062296	Trank1	8036.427	-1.076	0.101	-10.625	2.3E-26	1.2E-23
ENSMUSG00000031932	Gpr83	986.495	-1.302	0.124	-10.539	5.7E-26	2.9E-23
ENSMUSG00000030102	Itpr1	16780.129	-0.946	0.090	-10.527	6.5E-26	3.2E-23
ENSMUSG00000044288	Cnr1	4387.835	-1.199	0.114	-10.485	1.0E-25	4.8E-23
ENSMUSG00000019943	Atp2b1	23137.619	-0.816	0.078	-10.463	1.3E-25	5.9E-23
ENSMUSG00000029054	Gabrd	845.418	-1.388	0.134	-10.364	3.6E-25	1.6E-22
ENSMUSG00000033730	Egr3	2087.468	-1.064	0.103	-10.319	5.8E-25	2.5E-22
ENSMUSG00000053310	Nrgn	7891.595	-0.861	0.084	-10.263	1.0E-24	4.3E-22
ENSMUSG00000037754	Ppp1r16b	4566.387	-0.906	0.089	-10.192	2.2E-24	8.7E-22
ENSMUSG00000038976	Ppp1r9b	12863.197	-0.870	0.086	-10.085	6.5E-24	2.6E-21
ENSMUSG00000054728	Phactr1	12823.679	-0.860	0.087	-9.893	4.5E-23	1.7E-20
ENSMUSG00000039059	Hrh3	679.314	-1.552	0.162	-9.577	1.0E-21	3.7E-19
ENSMUSG00000022489	Pde1b	10081.893	-1.046	0.111	-9.458	3.1E-21	1.1E-18
ENSMUSG00000041324	Inhba	390.580	-1.680	0.178	-9.445	3.5E-21	1.3E-18
ENSMUSG00000038128	Camk4	1531.886	-1.023	0.109	-9.377	6.8E-21	2.4E-18
ENSMUSG00000023274	Cd4	550.936	-1.682	0.180	-9.345	9.2E-21	3.1E-18
ENSMUSG00000026834	Acvr1c	1331.999	-1.111	0.119	-9.319	1.2E-20	3.9E-18
ENSMUSG00000025221	Kcnip2	2236.596	-0.951	0.102	-9.307	1.3E-20	4.2E-18
ENSMUSG00000032259	Drd2	1748.467	-1.321	0.143	-9.270	1.9E-20	5.9E-18
ENSMUSG00000109372	Gm19410	531.196	-1.469	0.159	-9.217	3.1E-20	9.4E-18
ENSMUSG00000036902	Neto2	2609.483	-0.944	0.104	-9.119	7.6E-20	2.3E-17
ENSMUSG00000027296	Itpka	876.692	-1.202	0.133	-9.070	1.2E-19	3.5E-17
ENSMUSG00000034472	Rasd2	4837.555	-1.124	0.124	-9.048	1.5E-19	4.2E-17
ENSMUSG00000056486	Chn1	11994.754	-0.851	0.097	-8.777	1.7E-18	4.7E-16
ENSMUSG00000026259	Ngef	4569.022	-0.882	0.101	-8.753	2.1E-18	5.6E-16
ENSMUSG00000097993	Ptprv	186.316	-2.781	0.318	-8.753	2.1E-18	5.6E-16
ENSMUSG00000003273	Car11	1745.362	-0.936	0.107	-8.727	2.6E-18	7.0E-16

ENSMUSG00000025867	Cplx2	26165.874	-0.696	0.080	-8.681	3.9E-18	1.0E-15
ENSMUSG00000038400	Pmepa1	846.967	-1.122	0.129	-8.675	4.1E-18	1.1E-15
ENSMUSG00000020684	Rasl10b	5366.288	-0.809	0.094	-8.575	9.9E-18	2.5E-15
ENSMUSG00000078307	AI593442	4465.000	-0.823	0.096	-8.561	1.1E-17	2.8E-15
ENSMUSG00000031778	Cx3cl1	7359.451	-0.738	0.087	-8.534	1.4E-17	3.4E-15
ENSMUSG00000026077	Npas2	739.380	-1.221	0.143	-8.532	1.4E-17	3.4E-15
ENSMUSG00000048644	Ctxn1	2674.175	-0.832	0.098	-8.507	1.8E-17	4.2E-15
ENSMUSG00000042388	Dlgap3	4997.929	-0.823	0.097	-8.477	2.3E-17	5.3E-15
ENSMUSG00000033960	Jcad	4893.234	-0.816	0.097	-8.403	4.3E-17	9.8E-15
ENSMUSG00000086040	Wipf3	3066.892	-0.790	0.096	-8.226	1.9E-16	4.3E-14
ENSMUSG00000032366	Tpm1	3475.297	-0.761	0.093	-8.181	2.8E-16	6.2E-14
ENSMUSG00000045349	Sh2d5	1616.584	-0.936	0.115	-8.123	4.5E-16	9.7E-14
ENSMUSG00000056812	St8sia3	6906.523	-0.816	0.100	-8.122	4.6E-16	9.7E-14
ENSMUSG00000046607	Hrk	653.794	-1.443	0.179	-8.053	8.1E-16	1.7E-13
ENSMUSG00000021719	Rgs7bp	10153.764	-0.669	0.083	-8.032	9.6E-16	2.0E-13
ENSMUSG00000029471	Camkk2	3626.535	-0.865	0.108	-8.013	1.1E-15	2.3E-13
ENSMUSG00000022197	Pdzd2	1768.919	-0.895	0.113	-7.911	2.5E-15	5.1E-13
ENSMUSG00000029359	Tesc	497.109	-1.279	0.163	-7.833	4.8E-15	9.4E-13
ENSMUSG00000030123	Plxnd1	1528.941	-0.949	0.124	-7.679	1.6E-14	3.1E-12
ENSMUSG00000067889	Sptbn2	12985.529	-0.630	0.082	-7.636	2.2E-14	4.3E-12
ENSMUSG00000032946	Rasgrp2	1559.394	-0.973	0.129	-7.526	5.2E-14	9.9E-12
ENSMUSG00000044216	Kcnj4	1620.325	-0.887	0.118	-7.507	6.1E-14	1.1E-11
ENSMUSG00000027400	Pdyn	1079.876	-0.952	0.127	-7.502	6.3E-14	1.1E-11
ENSMUSG00000036882	Arhgap33	2302.771	-0.751	0.100	-7.503	6.2E-14	1.1E-11
ENSMUSG00000027827	Kcnab1	4973.166	-0.810	0.109	-7.435	1.0E-13	1.9E-11
ENSMUSG00000022490	Ppp1r1a	578.609	-1.307	0.177	-7.394	1.4E-13	2.5E-11
ENSMUSG00000037126	Psd	3604.512	-0.667	0.090	-7.375	1.6E-13	2.9E-11
ENSMUSG00000072214	Septin5	5909.155	-0.797	0.108	-7.359	1.8E-13	3.2E-11
ENSMUSG00000118401	Gpr52	677.167	-1.180	0.161	-7.342	2.1E-13	3.6E-11
ENSMUSG00000051177	Plcb1	6899.882	-0.618	0.084	-7.332	2.3E-13	3.8E-11
ENSMUSG00000030302	Atp2b2	11955.258	-0.661	0.090	-7.303	2.8E-13	4.7E-11
ENSMUSG00000022602	Arc	881.563	-1.010	0.138	-7.297	3.0E-13	4.8E-11
ENSMUSG00000030854	Ptpn5	5041.411	-0.690	0.095	-7.290	3.1E-13	5.0E-11
ENSMUSG00000030376	Slc8a2	2869.208	-0.714	0.098	-7.259	3.9E-13	6.2E-11
ENSMUSG00000037703	Lzts3	3297.553	-0.763	0.105	-7.255	4.0E-13	6.4E-11
ENSMUSG00000052087	Rgs14	463.350	-1.240	0.171	-7.252	4.1E-13	6.5E-11
ENSMUSG00000029406	Pitpnm2	2613.327	-0.812	0.112	-7.226	5.0E-13	7.7E-11
ENSMUSG00000042700	Sipa1l1	7976.282	-0.592	0.082	-7.205	5.8E-13	8.9E-11
ENSMUSG00000023945	Slc5a7	553.610	-1.111	0.155	-7.186	6.7E-13	1.0E-10
ENSMUSG00000032936	Camkv	6253.901	-0.632	0.088	-7.176	7.2E-13	1.1E-10
ENSMUSG00000045967	Gpr158	4605.679	-0.631	0.088	-7.176	7.2E-13	1.1E-10
ENSMUSG00000021750	Fam107a	4738.196	-0.893	0.125	-7.162	7.9E-13	1.2E-10
ENSMUSG00000052374	Actn2	850.113	-1.166	0.163	-7.151	8.6E-13	1.2E-10
ENSMUSG00000030134	Rasgef1a	5267.482	-0.643	0.090	-7.142	9.2E-13	1.3E-10
ENSMUSG00000026090	Cracdl	4333.539	-0.640	0.090	-7.142	9.2E-13	1.3E-10
ENSMUSG00000003134	Tbc1d8	968.391	-0.911	0.128	-7.139	9.4E-13	1.3E-10
ENSMUSG00000027797	Dclk1	12262.907	-0.754	0.106	-7.106	1.2E-12	1.7E-10
ENSMUSG00000022537	Tmem44	1205.967	-0.855	0.121	-7.078	1.5E-12	2.0E-10
ENSMUSG00000027347	Rasgrp1	10450.583	-0.617	0.087	-7.056	1.7E-12	2.3E-10
ENSMUSG00000035355	Kcnh4	275.159	-1.424	0.202	-7.051	1.8E-12	2.4E-10
ENSMUSG00000023868	Pde10a	14161.777	-1.575	0.224	-7.048	1.8E-12	2.4E-10
ENSMUSG00000021991	Cacna2d3	2863.225	-0.702	0.100	-7.029	2.1E-12	2.7E-10
ENSMUSG00000041351	Rap1gap	3976.774	-0.640	0.091	-7.028	2.1E-12	2.7E-10
ENSMUSG00000047842	Diras2	4346.537	-0.732	0.104	-7.026	2.1E-12	2.8E-10
ENSMUSG00000020823	Sec14l1	3003.743	-0.717	0.102	-7.020	2.2E-12	2.9E-10
ENSMUSG00000033063	Cntnap3	496.647	-1.194	0.170	-7.017	2.3E-12	2.9E-10

ENSMUSG00000046922	Gpr6	371.012	-1.468	0.209	-7.014	2.3E-12	2.9E-10
ENSMUSG00000021062	Rab15	3348.687	-0.708	0.101	-7.012	2.4E-12	3.0E-10
ENSMUSG00000038418	Egr1	2792.979	-0.767	0.110	-6.992	2.7E-12	3.4E-10
ENSMUSG00000094626	Tmem121b	733.086	-0.961	0.139	-6.934	4.1E-12	5.0E-10
ENSMUSG00000021115	Vrk1	568.499	-1.148	0.166	-6.933	4.1E-12	5.0E-10
ENSMUSG00000038264	Sema7a	1330.903	-0.986	0.143	-6.913	4.7E-12	5.7E-10
ENSMUSG00000061911	Myt1l	6042.992	-0.593	0.086	-6.911	4.8E-12	5.8E-10
ENSMUSG00000049225	Pdp1	2672.060	-0.733	0.107	-6.867	6.6E-12	7.8E-10
ENSMUSG00000024077	Strn	5131.799	-0.664	0.097	-6.826	8.8E-12	1.0E-09
ENSMUSG00000051067	Lingo3	781.145	-0.969	0.142	-6.823	8.9E-12	1.0E-09
ENSMUSG00000037855	Zfp365	10137.435	-0.624	0.092	-6.804	1.0E-11	1.2E-09
ENSMUSG00000037579	Kcnh3	963.397	-0.903	0.134	-6.750	1.5E-11	1.7E-09
ENSMUSG00000032586	Traip	383.521	-1.235	0.184	-6.723	1.8E-11	2.0E-09
ENSMUSG00000009681	Bcr	4997.938	-0.636	0.095	-6.710	1.9E-11	2.2E-09
ENSMUSG00000014786	Slc9a5	504.557	-1.057	0.158	-6.704	2.0E-11	2.3E-09
ENSMUSG00000033287	Kctd17	1776.144	-0.699	0.104	-6.701	2.1E-11	2.3E-09
ENSMUSG00000045441	Gprin3	1831.472	-0.768	0.115	-6.677	2.4E-11	2.7E-09
ENSMUSG00000025372	Baiap2	3747.884	-0.675	0.102	-6.651	2.9E-11	3.2E-09
ENSMUSG00000042489	Clspn	184.191	-1.994	0.300	-6.648	3.0E-11	3.2E-09
ENSMUSG00000001270	Ckb	9142.688	-0.576	0.087	-6.622	3.5E-11	3.8E-09
ENSMUSG00000059456	Ptk2b	6969.341	-0.550	0.083	-6.615	3.7E-11	4.0E-09
ENSMUSG00000057897	Camk2b	14102.681	-0.528	0.080	-6.613	3.8E-11	4.0E-09
ENSMUSG00000006205	Htra1	1499.471	-0.792	0.120	-6.608	3.9E-11	4.1E-09
ENSMUSG00000022075	Rhobtb2	1898.365	-0.691	0.105	-6.584	4.6E-11	4.8E-09
ENSMUSG00000004849	Ap1s1	2165.859	-0.707	0.108	-6.561	5.4E-11	5.6E-09
ENSMUSG00000066197	Gpr139	149.378	-1.823	0.278	-6.549	5.8E-11	6.0E-09
ENSMUSG00000004366	Sst	1023.770	-0.849	0.130	-6.533	6.5E-11	6.6E-09
ENSMUSG00000072966	Gprasp2	3596.596	0.710	0.109	6.524	6.8E-11	6.9E-09
ENSMUSG00000011877	Git1	3734.790	-0.677	0.104	-6.523	6.9E-11	6.9E-09
ENSMUSG00000000530	Acvrl1	338.665	-1.213	0.187	-6.480	9.2E-11	9.2E-09
ENSMUSG00000038370	Pcp4l1	4518.906	-0.728	0.112	-6.479	9.2E-11	9.2E-09
ENSMUSG00000022623	Shank3	3722.053	-0.617	0.095	-6.473	9.6E-11	9.5E-09
ENSMUSG00000028033	Kcnq5	2075.102	-0.667	0.103	-6.458	1.1E-10	1.0E-08
ENSMUSG00000055805	Fmnl1	922.141	-0.839	0.130	-6.452	1.1E-10	1.1E-08
ENSMUSG00000018849	Wwc1	1466.562	-0.819	0.128	-6.397	1.6E-10	1.5E-08
ENSMUSG00000024883	Rin1	873.514	-0.891	0.140	-6.369	1.9E-10	1.8E-08
ENSMUSG00000018648	Dusp14	417.949	-1.096	0.172	-6.368	1.9E-10	1.8E-08
ENSMUSG00000037624	Kcnk2	2029.330	-0.710	0.112	-6.342	2.3E-10	2.1E-08
ENSMUSG00000040495	Chrm4	374.783	-1.129	0.178	-6.342	2.3E-10	2.1E-08
ENSMUSG00000027298	Tyro3	2361.653	-0.627	0.099	-6.335	2.4E-10	2.2E-08
ENSMUSG00000050511	Oprd1	284.213	-1.262	0.199	-6.333	2.4E-10	2.2E-08
ENSMUSG00000061751	Kalrn	5638.335	-0.700	0.111	-6.284	3.3E-10	3.0E-08
ENSMUSG00000020189	Osbpl8	4694.067	-0.551	0.088	-6.273	3.5E-10	3.2E-08
ENSMUSG00000051323	Pcdh19	1026.696	0.861	0.137	6.272	3.6E-10	3.2E-08
ENSMUSG00000023019	Gpd1	842.941	-0.916	0.146	-6.264	3.7E-10	3.4E-08
ENSMUSG00000023150	Ivns1abp	5939.076	-0.582	0.093	-6.260	3.9E-10	3.5E-08
ENSMUSG00000028278	Rragd	4256.126	-0.568	0.091	-6.246	4.2E-10	3.8E-08
ENSMUSG00000040385	Ppp1ca	3403.676	-0.626	0.101	-6.227	4.8E-10	4.2E-08
ENSMUSG00000028328	Tmod1	1050.514	-0.774	0.124	-6.225	4.8E-10	4.2E-08
ENSMUSG00000021182	Ccdc88c	1327.826	-0.744	0.120	-6.215	5.1E-10	4.5E-08
ENSMUSG00000003469	Phyhip	5262.614	-0.532	0.086	-6.208	5.4E-10	4.7E-08
ENSMUSG00000024617	Camk2a	29997.504	-0.614	0.099	-6.204	5.5E-10	4.7E-08
ENSMUSG00000085042	Abhd11os	36.508	-4.079	0.658	-6.201	5.6E-10	4.8E-08
ENSMUSG00000051747	Ttn	47.891	3.237	0.522	6.202	5.6E-10	4.8E-08
ENSMUSG00000021870	Slmap	3574.974	-0.558	0.090	-6.195	5.8E-10	4.9E-08
ENSMUSG00000048240	Gng7	11991.184	-0.763	0.123	-6.195	5.8E-10	4.9E-08

ENSMUSG00000048458	Inka2	1389.227	-0.765	0.124	-6.190	6.0E-10	5.0E-08
ENSMUSG00000021379	Id4	3899.085	-0.592	0.096	-6.171	6.8E-10	5.6E-08
ENSMUSG00000021209	Ppp4r4	980.633	-0.771	0.125	-6.170	6.8E-10	5.6E-08
ENSMUSG00000030688	Stard10	634.251	-0.878	0.143	-6.148	7.8E-10	6.4E-08
ENSMUSG00000017390	Aldoc	7650.354	-0.670	0.110	-6.106	1.0E-09	8.4E-08
ENSMUSG00000014782	Plekhg4	42.280	-3.654	0.603	-6.061	1.4E-09	1.1E-07
ENSMUSG00000031342	Gpm6b	25606.732	-0.534	0.089	-6.019	1.8E-09	1.4E-07
ENSMUSG00000101878	Gm8203	25.959	8.035	1.335	6.020	1.7E-09	1.4E-07
ENSMUSG00000027419	Pcsk2	5598.664	-0.569	0.095	-5.989	2.1E-09	1.7E-07
ENSMUSG00000031906	Smpd3	2586.271	-0.665	0.111	-5.982	2.2E-09	1.8E-07
ENSMUSG00000052812	Atad2b	911.763	0.960	0.161	5.960	2.5E-09	2.0E-07
ENSMUSG00000029348	Asphd2	1185.142	-0.702	0.118	-5.955	2.6E-09	2.0E-07
ENSMUSG00000025499	Hras	1162.125	-0.701	0.118	-5.922	3.2E-09	2.5E-07
ENSMUSG00000056073	Grik2	2666.517	-0.603	0.102	-5.903	3.6E-09	2.8E-07
ENSMUSG00000014355	Anapc1	2775.309	0.745	0.126	5.895	3.7E-09	2.9E-07
ENSMUSG00000036225	Kctd1	1403.840	-0.685	0.116	-5.895	3.8E-09	2.9E-07
ENSMUSG00000033174	Mgll	2208.815	-0.636	0.108	-5.892	3.8E-09	2.9E-07
ENSMUSG00000089809	Rasgef1b	1302.510	-0.687	0.117	-5.889	3.9E-09	3.0E-07
ENSMUSG00000021259	Cyp46a1	1987.902	-0.599	0.102	-5.887	3.9E-09	3.0E-07
ENSMUSG00000041986	Elmod1	5549.743	-0.548	0.093	-5.882	4.0E-09	3.0E-07
ENSMUSG00000047888	Tnrc6b	3091.486	0.586	0.100	5.876	4.2E-09	3.1E-07
ENSMUSG00000033953	Ppp3r1	16469.587	-0.504	0.086	-5.864	4.5E-09	3.4E-07
ENSMUSG00000023034	Nr4a1	650.875	-0.864	0.147	-5.861	4.6E-09	3.4E-07
ENSMUSG00000066607	Insyn1	1863.565	-0.662	0.114	-5.830	5.5E-09	4.1E-07
ENSMUSG00000047507	Baiap3	602.185	0.960	0.165	5.826	5.7E-09	4.2E-07
ENSMUSG00000021057	Akap5	4896.520	-0.601	0.103	-5.820	5.9E-09	4.3E-07
ENSMUSG00000043439	Epop	221.011	-1.507	0.259	-5.814	6.1E-09	4.4E-07
ENSMUSG00000024186	Rgs11	246.209	-1.286	0.223	-5.775	7.7E-09	5.5E-07
ENSMUSG00000034997	Htr2a	494.700	-1.155	0.200	-5.775	7.7E-09	5.5E-07
ENSMUSG00000027134	Lpcat4	957.368	-0.723	0.126	-5.750	8.9E-09	6.3E-07
ENSMUSG00000037224	Zfyve28	707.088	-0.815	0.142	-5.741	9.4E-09	6.7E-07
ENSMUSG00000057457	Phex	60.908	-2.630	0.458	-5.736	9.7E-09	6.8E-07
ENSMUSG00000020723	Cacng4	1568.634	-0.708	0.123	-5.735	9.8E-09	6.9E-07
ENSMUSG00000060371	Caln1	8569.785	-0.524	0.092	-5.693	1.2E-08	8.7E-07
ENSMUSG00000036095	Dgkb	9669.571	-0.555	0.098	-5.678	1.4E-08	9.5E-07
ENSMUSG00000049303	Syt12	457.693	-0.928	0.164	-5.670	1.4E-08	9.9E-07
ENSMUSG00000110195	Pde2a	7391.910	-0.556	0.098	-5.669	1.4E-08	9.9E-07
ENSMUSG00000026663	Atf6	1727.330	-0.635	0.112	-5.647	1.6E-08	1.1E-06
ENSMUSG00000021071	Trim9	4114.849	-0.532	0.094	-5.645	1.7E-08	1.1E-06
ENSMUSG00000025789	St8sia2	184.647	-1.450	0.257	-5.634	1.8E-08	1.2E-06
ENSMUSG00000005089	Slc1a2	66431.288	-0.517	0.092	-5.627	1.8E-08	1.2E-06
ENSMUSG00000061718	Ppp1r1b	9348.647	-1.388	0.247	-5.618	1.9E-08	1.3E-06
ENSMUSG00000049511	Htr1b	710.197	-0.882	0.157	-5.616	2.0E-08	1.3E-06
ENSMUSG00000031506	Ptpn7	53.865	-2.657	0.474	-5.606	2.1E-08	1.4E-06
ENSMUSG00000030270	Cpne9	235.762	-1.658	0.296	-5.603	2.1E-08	1.4E-06
ENSMUSG00000048978	Nrsn1	4753.492	-0.565	0.101	-5.589	2.3E-08	1.5E-06
ENSMUSG00000001175	Calm1	45760.501	-0.540	0.097	-5.589	2.3E-08	1.5E-06
ENSMUSG00000028656	Cap1	4444.170	-0.547	0.098	-5.591	2.3E-08	1.5E-06
ENSMUSG00000051650	B3gnt2	1079.907	-0.821	0.147	-5.591	2.3E-08	1.5E-06
ENSMUSG00000068373	D430041D05Ril	3220.323	-0.543	0.098	-5.568	2.6E-08	1.7E-06
ENSMUSG00000022842	Ece2	774.502	0.786	0.143	5.491	4.0E-08	2.6E-06
ENSMUSG00000032572	Col6a4	101.950	-1.845	0.336	-5.490	4.0E-08	2.6E-06
ENSMUSG00000020886	Dlg4	11643.942	-0.443	0.081	-5.476	4.4E-08	2.8E-06
ENSMUSG00000032698	Lmo2	494.769	-0.908	0.166	-5.465	4.6E-08	2.9E-06
ENSMUSG00000116594	Gm49601	772.132	-0.818	0.150	-5.465	4.6E-08	2.9E-06
ENSMUSG00000004056	Akt2	1723.522	-0.610	0.112	-5.448	5.1E-08	3.2E-06

ENSMUSG00000051617	Krt9	362.508	-1.051	0.193	-5.445	5.2E-08	3.2E-06
ENSMUSG00000029093	Sorcs2	1089.139	-0.708	0.130	-5.443	5.2E-08	3.3E-06
ENSMUSG00000015843	Rxrg	998.387	-0.959	0.176	-5.442	5.3E-08	3.3E-06
ENSMUSG00000041828	Abca8a	464.286	0.962	0.177	5.433	5.5E-08	3.4E-06
ENSMUSG00000021373	Cap2	3763.300	-0.489	0.090	-5.430	5.6E-08	3.5E-06
ENSMUSG00000043079	Synpo	2213.814	-0.537	0.099	-5.410	6.3E-08	3.9E-06
ENSMUSG00000005198	Polr2a	2176.710	0.750	0.139	5.409	6.3E-08	3.9E-06
ENSMUSG00000004341	Gpx6	23.954	-4.825	0.893	-5.404	6.5E-08	4.0E-06
ENSMUSG00000079056	Kcnip3	1523.870	-0.662	0.123	-5.399	6.7E-08	4.0E-06
ENSMUSG00000058589	Anks1b	7651.804	-0.496	0.092	-5.399	6.7E-08	4.0E-06
ENSMUSG00000025905	Oprk1	595.153	-0.843	0.156	-5.399	6.7E-08	4.0E-06
ENSMUSG00000090071	Cdk5r2	2389.286	-0.535	0.099	-5.384	7.3E-08	4.3E-06
ENSMUSG00000000555	Itga5	185.914	-1.398	0.261	-5.359	8.3E-08	4.9E-06
ENSMUSG00000054162	Spock3	6813.047	-0.506	0.095	-5.346	9.0E-08	5.3E-06
ENSMUSG00000056665	Them6	340.411	-1.050	0.196	-5.347	8.9E-08	5.3E-06
ENSMUSG00000022237	Ankrd33b	1111.473	-0.670	0.125	-5.344	9.1E-08	5.3E-06
ENSMUSG00000030605	Mfge8	1325.359	-0.677	0.127	-5.326	1.0E-07	5.8E-06
ENSMUSG00000090125	Pou3f1	537.651	-0.818	0.154	-5.303	1.1E-07	6.6E-06
ENSMUSG00000003949	Hlf	3470.980	-0.519	0.098	-5.287	1.2E-07	7.2E-06
ENSMUSG00000027221	Chst1	2826.688	-0.520	0.099	-5.277	1.3E-07	7.6E-06
ENSMUSG00000033161	Atp1a1	7941.811	-0.556	0.105	-5.275	1.3E-07	7.6E-06
ENSMUSG00000016346	Kcnq2	8939.582	-0.437	0.083	-5.272	1.4E-07	7.7E-06
ENSMUSG00000048038	Ccdc187	583.910	-0.774	0.147	-5.268	1.4E-07	7.8E-06
ENSMUSG00000017491	Rarb	1321.454	-0.758	0.144	-5.261	1.4E-07	8.1E-06
ENSMUSG00000029467	Atp2a2	15253.151	-0.454	0.087	-5.244	1.6E-07	8.9E-06
ENSMUSG00000058013	Septin11	2549.281	-0.522	0.100	-5.229	1.7E-07	9.5E-06
ENSMUSG00000021765	Fst	51.565	-2.471	0.472	-5.230	1.7E-07	9.5E-06
ENSMUSG00000043384	Gprasp1	16035.784	0.455	0.087	5.222	1.8E-07	9.8E-06
ENSMUSG00000036062	Phf24	5485.159	-0.526	0.101	-5.212	1.9E-07	1.0E-05
ENSMUSG00000062372	Otof	438.174	-0.947	0.182	-5.208	1.9E-07	1.1E-05
ENSMUSG00000015879	Fam184b	325.275	-0.969	0.186	-5.206	1.9E-07	1.1E-05
ENSMUSG00000022052	Ppp2r2a	2999.159	-0.511	0.098	-5.197	2.0E-07	1.1E-05
ENSMUSG00000026473	Glul	24740.332	-0.496	0.095	-5.192	2.1E-07	1.1E-05
ENSMUSG00000031841	Cdh13	1502.511	-0.581	0.112	-5.182	2.2E-07	1.2E-05
ENSMUSG00000000632	Sez6	4169.219	-0.535	0.103	-5.179	2.2E-07	1.2E-05
ENSMUSG00000027270	Lamp5	1272.839	-0.723	0.140	-5.179	2.2E-07	1.2E-05
ENSMUSG00000039115	Itga9	262.285	-1.110	0.214	-5.178	2.2E-07	1.2E-05
ENSMUSG00000030122	Ptms	3620.609	-0.491	0.095	-5.147	2.6E-07	1.4E-05
ENSMUSG00000032827	Ppp1r9a	11918.311	-0.448	0.087	-5.136	2.8E-07	1.5E-05
ENSMUSG00000045318	Adra2c	359.808	-0.948	0.185	-5.125	3.0E-07	1.6E-05
ENSMUSG00000007682	Dio2	1566.488	-0.607	0.119	-5.118	3.1E-07	1.6E-05
ENSMUSG00000033863	Klf9	5038.572	-0.483	0.095	-5.111	3.2E-07	1.7E-05
ENSMUSG00000032192	Gnb5	2886.842	-0.489	0.096	-5.106	3.3E-07	1.7E-05
ENSMUSG00000035314	Gdpd5	468.572	-0.856	0.168	-5.104	3.3E-07	1.7E-05
ENSMUSG00000022752	Tomm70a	7670.983	-0.450	0.089	-5.066	4.1E-07	2.1E-05
ENSMUSG00000044167	Foxo1	1605.570	-0.643	0.127	-5.052	4.4E-07	2.3E-05
ENSMUSG00000008658	Rbfox1	5630.843	-0.431	0.085	-5.046	4.5E-07	2.3E-05
ENSMUSG00000070000	Fcho1	1059.448	-0.630	0.125	-5.043	4.6E-07	2.4E-05
ENSMUSG00000044783	Hjurp	1593.142	0.616	0.122	5.037	4.7E-07	2.4E-05
ENSMUSG00000037428	Vgf	1660.486	-0.540	0.107	-5.035	4.8E-07	2.4E-05
ENSMUSG00000075316	Scn9a	443.449	1.074	0.213	5.032	4.9E-07	2.5E-05
ENSMUSG00000031765	Mt1	911.890	-0.658	0.131	-5.028	5.0E-07	2.5E-05
ENSMUSG00000019990	Pde7b	3057.777	-0.609	0.122	-5.002	5.7E-07	2.9E-05
ENSMUSG00000032517	Mobp	20811.622	-0.402	0.080	-5.001	5.7E-07	2.9E-05
ENSMUSG00000032174	Icam5	1560.472	-0.551	0.110	-5.000	5.7E-07	2.9E-05
ENSMUSG00000029608	Rph3a	4265.752	-0.592	0.118	-4.998	5.8E-07	2.9E-05

ENSMUSG00000029208	Guf1	1649.580	0.633	0.127	4.994	5.9E-07	3.0E-05
ENSMUSG00000032267	Usp28	826.454	-0.670	0.135	-4.970	6.7E-07	3.3E-05
ENSMUSG00000016179	Camk1g	197.351	-1.199	0.242	-4.958	7.1E-07	3.5E-05
ENSMUSG00000063239	Grm4	630.770	-0.719	0.145	-4.955	7.2E-07	3.6E-05
ENSMUSG00000003352	Cacnb3	1805.219	-0.637	0.129	-4.954	7.3E-07	3.6E-05
ENSMUSG00000085007	Smim43	173.460	-1.319	0.267	-4.946	7.6E-07	3.7E-05
ENSMUSG00000021701	Plk2	3124.042	-0.517	0.105	-4.946	7.6E-07	3.7E-05
ENSMUSG00000026185	Igfbp5	2683.620	-0.568	0.115	-4.940	7.8E-07	3.8E-05
ENSMUSG00000059991	Nptx2	293.660	-1.155	0.234	-4.938	7.9E-07	3.8E-05
ENSMUSG00000029364	Wsb2	2777.305	-0.481	0.098	-4.932	8.1E-07	3.9E-05
ENSMUSG00000061186	Sfmbt2	215.697	1.300	0.264	4.931	8.2E-07	3.9E-05
ENSMUSG00000039103	Nexn	1338.976	-0.734	0.149	-4.929	8.3E-07	4.0E-05
ENSMUSG00000025422	Agap2	13628.971	-0.423	0.086	-4.913	9.0E-07	4.3E-05
ENSMUSG00000022342	Kcnv1	1183.831	-0.657	0.134	-4.912	9.0E-07	4.3E-05
ENSMUSG00000031837	Necab2	1470.194	-0.610	0.124	-4.901	9.6E-07	4.5E-05
ENSMUSG00000042834	Nrep	828.943	-0.820	0.168	-4.892	1.0E-06	4.7E-05
ENSMUSG00000037795	N4bp2	607.980	0.857	0.175	4.890	1.0E-06	4.7E-05
ENSMUSG00000048251	Bcl11b	4748.480	-0.568	0.117	-4.870	1.1E-06	5.3E-05
ENSMUSG00000042429	Adora1	2695.139	-0.579	0.119	-4.866	1.1E-06	5.3E-05
ENSMUSG00000020827	Mink1	5566.922	-0.421	0.087	-4.862	1.2E-06	5.4E-05
ENSMUSG00000041729	Coro2b	2641.034	-0.471	0.097	-4.861	1.2E-06	5.4E-05
ENSMUSG00000025576	Rbfox3	3605.946	-0.438	0.090	-4.857	1.2E-06	5.5E-05
ENSMUSG00000023018	Smarcd1	2433.509	-0.495	0.102	-4.848	1.2E-06	5.8E-05
ENSMUSG00000032336	Nptn	9904.286	-0.399	0.082	-4.845	1.3E-06	5.8E-05
ENSMUSG00000022054	Nefm	3706.508	-0.594	0.123	-4.840	1.3E-06	5.9E-05
ENSMUSG00000019997	Ccn2	163.291	-1.518	0.314	-4.836	1.3E-06	6.0E-05
ENSMUSG00000029722	Agfg2	597.273	-0.708	0.147	-4.828	1.4E-06	6.3E-05
ENSMUSG00000017493	Igfbp4	451.857	-0.960	0.199	-4.815	1.5E-06	6.7E-05
ENSMUSG00000035397	Klf16	810.234	-0.642	0.134	-4.799	1.6E-06	7.2E-05
ENSMUSG00000030905	Crym	967.822	-0.675	0.141	-4.793	1.6E-06	7.4E-05
ENSMUSG00000037996	Slc24a2	12114.569	-0.504	0.105	-4.794	1.6E-06	7.4E-05
ENSMUSG00000026764	Kif5c	19284.848	-0.469	0.098	-4.792	1.7E-06	7.4E-05
ENSMUSG00000040640	Erc2	2860.642	-0.464	0.097	-4.785	1.7E-06	7.7E-05
ENSMUSG00000021124	Vti1b	1772.274	-0.507	0.106	-4.776	1.8E-06	8.0E-05
ENSMUSG00000020889	Nr1d1	1081.954	-0.616	0.129	-4.766	1.9E-06	8.3E-05
ENSMUSG00000050164	Mchr1	336.478	-0.922	0.194	-4.757	2.0E-06	8.7E-05
ENSMUSG00000028086	Fbxw7	2946.616	-0.597	0.126	-4.742	2.1E-06	9.3E-05
ENSMUSG00000025658	Cnksr2	4319.603	-0.418	0.088	-4.738	2.2E-06	9.5E-05
ENSMUSG00000025885	Myo5b	834.013	-0.642	0.136	-4.730	2.2E-06	9.9E-05
ENSMUSG00000000378	Ccm2	630.738	-0.678	0.144	-4.723	2.3E-06	1.0E-04
ENSMUSG00000024990	Rbp4	100.007	-1.513	0.321	-4.708	2.5E-06	1.1E-04
ENSMUSG00000070304	Scn2b	5687.846	-0.421	0.089	-4.700	2.6E-06	1.1E-04
ENSMUSG00000046329	Slc25a23	5923.522	-0.439	0.093	-4.698	2.6E-06	1.1E-04
ENSMUSG00000020642	Rnf144a	700.378	-0.676	0.144	-4.696	2.7E-06	1.1E-04
ENSMUSG00000033594	Spata2l	469.878	-0.815	0.174	-4.694	2.7E-06	1.2E-04
ENSMUSG00000013089	Etv5	1169.951	-0.551	0.118	-4.685	2.8E-06	1.2E-04
ENSMUSG00000031074	Fgf3	36.973	-2.538	0.542	-4.683	2.8E-06	1.2E-04
ENSMUSG00000050321	Neto1	3932.034	-0.455	0.097	-4.678	2.9E-06	1.2E-04
ENSMUSG00000052572	Dlg2	10743.587	-0.375	0.080	-4.665	3.1E-06	1.3E-04
ENSMUSG00000034799	Unc13a	4426.777	-0.454	0.098	-4.656	3.2E-06	1.4E-04
ENSMUSG00000003363	Pld3	5985.280	-0.392	0.085	-4.638	3.5E-06	1.5E-04
ENSMUSG00000070372	Capza1	1124.809	0.560	0.121	4.637	3.5E-06	1.5E-04
ENSMUSG00000043635	Adamts3	578.292	-0.695	0.151	-4.613	4.0E-06	1.7E-04
ENSMUSG00000040412	Elapor1	966.198	-0.592	0.128	-4.609	4.0E-06	1.7E-04
ENSMUSG00000033998	Kcnk1	1303.654	-0.530	0.115	-4.603	4.2E-06	1.7E-04
ENSMUSG00000029819	Npy	313.002	-0.925	0.201	-4.596	4.3E-06	1.8E-04

ENSMUSG00000036617	Etl4	1152.561	-0.612	0.133	-4.591	4.4E-06	1.8E-04
ENSMUSG00000006356	Crip2	888.783	-0.591	0.129	-4.590	4.4E-06	1.8E-04
ENSMUSG00000039474	Wfs1	1707.680	-0.496	0.108	-4.589	4.4E-06	1.8E-04
ENSMUSG00000030043	Tacr1	310.091	-0.912	0.199	-4.587	4.5E-06	1.8E-04
ENSMUSG00000040860	Crocc	670.913	-0.711	0.155	-4.578	4.7E-06	1.9E-04
ENSMUSG00000029120	Ppp2r2c	9298.150	-0.392	0.086	-4.575	4.8E-06	1.9E-04
ENSMUSG00000074796	Slc4a11	28.016	-3.062	0.669	-4.575	4.8E-06	1.9E-04
ENSMUSG00000019986	Ahi1	6808.427	0.401	0.088	4.575	4.8E-06	1.9E-04
ENSMUSG00000024558	Mapk4	1138.354	-0.545	0.119	-4.570	4.9E-06	2.0E-04
ENSMUSG00000030067	Foxp1	5458.949	-0.523	0.114	-4.571	4.8E-06	2.0E-04
ENSMUSG00000031833	Mast3	2869.031	-0.463	0.101	-4.570	4.9E-06	2.0E-04
ENSMUSG00000027457	Snph	3740.292	-0.472	0.103	-4.562	5.1E-06	2.0E-04
ENSMUSG00000023800	Tiam2	1420.158	-0.590	0.129	-4.561	5.1E-06	2.0E-04
ENSMUSG00000078591	Hs3st4	984.495	-0.610	0.134	-4.558	5.2E-06	2.1E-04
ENSMUSG00000030350	Prmt8	1149.106	-0.606	0.133	-4.553	5.3E-06	2.1E-04
ENSMUSG00000046056	Sbsn	257.239	-0.967	0.213	-4.551	5.3E-06	2.1E-04
ENSMUSG00000026452	Syt2	1015.150	-0.711	0.157	-4.524	6.1E-06	2.4E-04
ENSMUSG00000032846	Zswim6	1280.742	-0.572	0.127	-4.518	6.3E-06	2.5E-04
ENSMUSG00000066189	Cacng3	1200.587	-0.538	0.119	-4.513	6.4E-06	2.5E-04
ENSMUSG00000040606	Kazn	2371.754	-0.506	0.112	-4.513	6.4E-06	2.5E-04
ENSMUSG00000056091	St3gal5	1521.057	-0.526	0.116	-4.514	6.4E-06	2.5E-04
ENSMUSG00000019935	Slc17a8	228.695	-0.978	0.217	-4.499	6.8E-06	2.7E-04
ENSMUSG00000020882	Cacnb1	1670.408	-0.486	0.108	-4.497	6.9E-06	2.7E-04
ENSMUSG00000001986	Gria3	4320.352	-0.447	0.100	-4.490	7.1E-06	2.8E-04
ENSMUSG00000064368	mt-Nd6	6315.876	-0.444	0.099	-4.487	7.2E-06	2.8E-04
ENSMUSG00000016150	Tenm1	1816.402	0.478	0.107	4.478	7.5E-06	2.9E-04
ENSMUSG00000034570	Inpp5j	614.075	-0.658	0.147	-4.475	7.6E-06	2.9E-04
ENSMUSG00000032549	Rab6b	13944.819	-0.420	0.094	-4.471	7.8E-06	3.0E-04
ENSMUSG00000051853	Arf3	17219.016	-0.362	0.081	-4.466	8.0E-06	3.1E-04
ENSMUSG00000041773	Enc1	6376.684	-0.498	0.112	-4.463	8.1E-06	3.1E-04
ENSMUSG00000042350	Arel1	3530.652	-0.440	0.099	-4.463	8.1E-06	3.1E-04
ENSMUSG00000089997	1810020O05Rik	82.884	-1.566	0.351	-4.461	8.2E-06	3.1E-04
ENSMUSG00000010086	Rnf112	2728.822	-0.443	0.099	-4.457	8.3E-06	3.1E-04
ENSMUSG00000028782	Adgrb2	3703.852	-0.400	0.090	-4.457	8.3E-06	3.1E-04
ENSMUSG00000054871	Tmem158	1056.995	-0.664	0.149	-4.453	8.5E-06	3.2E-04
ENSMUSG00000034275	Igsf9b	1782.117	-0.523	0.117	-4.449	8.6E-06	3.2E-04
ENSMUSG00000035954	Dock4	2869.921	-0.434	0.098	-4.446	8.7E-06	3.3E-04
ENSMUSG00000056258	Kcnq3	5684.982	-0.389	0.088	-4.441	8.9E-06	3.3E-04
ENSMUSG00000042978	Sbk1	1050.981	-0.574	0.129	-4.434	9.2E-06	3.4E-04
ENSMUSG00000056296	Synpr	2562.931	-0.526	0.119	-4.433	9.3E-06	3.4E-04
ENSMUSG00000041380	Htr2c	2418.946	0.528	0.119	4.430	9.4E-06	3.5E-04
ENSMUSG00000030428	Ttyh1	6743.073	-0.402	0.091	-4.429	9.5E-06	3.5E-04
ENSMUSG00000027394	Ttl	1254.883	-0.519	0.117	-4.422	9.8E-06	3.6E-04
ENSMUSG00000052305	Hbb-bs	870.977	-0.654	0.148	-4.406	1.1E-05	3.9E-04
ENSMUSG00000057101	Zfp180	1377.303	-0.503	0.114	-4.401	1.1E-05	3.9E-04
ENSMUSG00000021360	Gcnt2	1078.863	-0.642	0.146	-4.401	1.1E-05	3.9E-04
ENSMUSG00000036620	Mgat4b	585.732	-0.641	0.146	-4.382	1.2E-05	4.3E-04
ENSMUSG00000066357	Wdr6	1678.860	0.479	0.109	4.372	1.2E-05	4.5E-04
ENSMUSG00000031604	Msmo1	1354.175	-0.599	0.137	-4.364	1.3E-05	4.6E-04
ENSMUSG00000020923	Ubtf	1982.009	-0.455	0.105	-4.350	1.4E-05	4.9E-04
ENSMUSG00000024486	Hbegf	233.728	-0.953	0.219	-4.344	1.4E-05	5.0E-04
ENSMUSG00000057378	Ryr3	3107.235	-0.530	0.122	-4.342	1.4E-05	5.1E-04
ENSMUSG00000054986	Sec14l3	33.709	-2.823	0.651	-4.335	1.5E-05	5.2E-04
ENSMUSG00000022463	Srebf2	2708.251	-0.433	0.100	-4.324	1.5E-05	5.5E-04
ENSMUSG00000020644	Id2	1193.993	-0.694	0.161	-4.319	1.6E-05	5.6E-04
ENSMUSG00000027346	Gpcpd1	2140.386	-0.484	0.112	-4.306	1.7E-05	5.9E-04

ENSMUSG00000047591	Mafa	38.751	-2.285	0.531	-4.303	1.7E-05	6.0E-04
ENSMUSG00000002341	Ncan	3224.951	-0.421	0.098	-4.303	1.7E-05	6.0E-04
ENSMUSG00000024008	Cpne5	2378.328	-0.518	0.121	-4.299	1.7E-05	6.1E-04
ENSMUSG00000021180	Rps6ka5	1212.770	-0.517	0.120	-4.299	1.7E-05	6.1E-04
ENSMUSG00000017167	Cntnap1	3518.331	-0.387	0.090	-4.296	1.7E-05	6.1E-04
ENSMUSG00000003974	Grm3	1512.627	-0.477	0.111	-4.293	1.8E-05	6.2E-04
ENSMUSG00000023067	Cdkn1a	147.343	-1.390	0.324	-4.292	1.8E-05	6.2E-04
ENSMUSG00000029427	Zcchc8	695.648	0.620	0.145	4.283	1.8E-05	6.4E-04
ENSMUSG00000028838	Extl1	429.341	-0.770	0.180	-4.275	1.9E-05	6.6E-04
ENSMUSG00000056692	Ilrun	3916.223	-0.394	0.092	-4.275	1.9E-05	6.6E-04
ENSMUSG00000022208	Jph4	5065.284	-0.370	0.087	-4.272	1.9E-05	6.7E-04
ENSMUSG00000034730	Adgrb1	3747.417	-0.402	0.094	-4.265	2.0E-05	6.9E-04
ENSMUSG00000020785	Camkk1	1073.404	-0.583	0.137	-4.258	2.1E-05	7.1E-04
ENSMUSG00000029436	Mmp17	1758.395	-0.483	0.114	-4.244	2.2E-05	7.6E-04
ENSMUSG00000056708	Ier5	574.272	-0.708	0.167	-4.243	2.2E-05	7.6E-04
ENSMUSG00000029361	Nos1	1023.468	-0.568	0.134	-4.240	2.2E-05	7.6E-04
ENSMUSG00000050505	Pcdh20	638.156	0.660	0.156	4.239	2.2E-05	7.7E-04
ENSMUSG00000032589	Bsn	5173.307	-0.464	0.109	-4.236	2.3E-05	7.7E-04
ENSMUSG00000017692	Rhbdl3	409.435	-0.718	0.170	-4.228	2.4E-05	8.0E-04
ENSMUSG00000027525	Phactr3	2545.221	-0.437	0.103	-4.228	2.4E-05	8.0E-04
ENSMUSG00000045294	Insig1	991.539	-0.654	0.155	-4.223	2.4E-05	8.1E-04
ENSMUSG00000026202	Tuba4a	4535.337	-0.465	0.111	-4.210	2.5E-05	8.6E-04
ENSMUSG00000049907	Rasl11b	323.384	-1.034	0.246	-4.205	2.6E-05	8.8E-04
ENSMUSG00000028351	Brinp1	2616.853	-0.456	0.109	-4.184	2.9E-05	9.6E-04
ENSMUSG00000015981	Stk32c	1633.247	-0.476	0.114	-4.181	2.9E-05	9.7E-04
ENSMUSG00000021596	Mctp1	2797.556	-0.443	0.106	-4.181	2.9E-05	9.7E-04
ENSMUSG00000070003	Ssbp4	753.992	-0.564	0.135	-4.178	2.9E-05	9.8E-04
ENSMUSG00000020340	Cyfip2	14266.837	-0.358	0.086	-4.177	2.9E-05	9.8E-04
ENSMUSG00000021919	Chat	128.900	-1.180	0.282	-4.177	3.0E-05	9.8E-04
ENSMUSG00000059824	Dbp	1050.427	-0.503	0.120	-4.175	3.0E-05	9.9E-04
ENSMUSG00000042500	ago-04	481.026	-0.680	0.163	-4.172	3.0E-05	1.0E-03
ENSMUSG00000025255	Zfhx4	662.487	0.635	0.152	4.171	3.0E-05	1.0E-03
ENSMUSG00000060260	Pwwp2b	287.858	-0.834	0.200	-4.162	3.1E-05	1.0E-03
ENSMUSG00000031626	Sorbs2	3469.901	-0.396	0.095	-4.161	3.2E-05	1.0E-03
ENSMUSG00000064345	mt-Nd2	#########	-0.333	0.080	-4.160	3.2E-05	1.0E-03
ENSMUSG00000047714	Ppp1r2	4134.708	-0.402	0.097	-4.149	3.3E-05	1.1E-03
ENSMUSG00000048895	Cdk5r1	4055.083	-0.387	0.093	-4.148	3.4E-05	1.1E-03
ENSMUSG00000030772	Dkk3	1813.094	-0.504	0.122	-4.147	3.4E-05	1.1E-03
ENSMUSG00000021676	Iqgap2	540.045	-0.657	0.159	-4.142	3.4E-05	1.1E-03
ENSMUSG00000028758	Kif17	542.841	-0.642	0.155	-4.139	3.5E-05	1.1E-03
ENSMUSG00000078137	Ankrd63	1642.186	-0.553	0.134	-4.137	3.5E-05	1.1E-03
ENSMUSG00000074896	Ifit3	251.768	0.932	0.225	4.133	3.6E-05	1.2E-03
ENSMUSG00000066036	Ubr4	4183.408	0.435	0.105	4.132	3.6E-05	1.2E-03
ENSMUSG00000035849	Krt222	654.438	-0.790	0.191	-4.129	3.6E-05	1.2E-03
ENSMUSG00000022533	Atp13a3	1787.648	0.427	0.104	4.113	3.9E-05	1.2E-03
ENSMUSG00000020734	Grin2c	488.100	-0.678	0.165	-4.101	4.1E-05	1.3E-03
ENSMUSG00000000308	Ckmt1	1593.098	-0.469	0.114	-4.097	4.2E-05	1.3E-03
ENSMUSG00000024661	Fth1	19226.961	-0.325	0.079	-4.095	4.2E-05	1.3E-03
ENSMUSG00000038630	Zkscan16	1350.229	0.477	0.117	4.095	4.2E-05	1.3E-03
ENSMUSG00000089762	Ier5l	101.829	-1.356	0.331	-4.094	4.2E-05	1.3E-03
ENSMUSG00000022176	Rem2	320.099	-0.906	0.221	-4.093	4.2E-05	1.3E-03
ENSMUSG00000051149	Adnp	1487.372	0.494	0.121	4.090	4.3E-05	1.4E-03
ENSMUSG00000102070	Gm28661	#########	-0.399	0.098	-4.084	4.4E-05	1.4E-03
ENSMUSG00000019146	Cacng2	1805.439	-0.438	0.108	-4.071	4.7E-05	1.5E-03
ENSMUSG00000055003	Lrtm2	936.016	-0.505	0.124	-4.069	4.7E-05	1.5E-03
ENSMUSG00000038173	Enpp6	513.945	0.663	0.163	4.066	4.8E-05	1.5E-03

ENSMUSG00000022604	Cep97	968.713	0.542	0.133	4.061	4.9E-05	1.5E-03
ENSMUSG00000036264	Fstl4	380.843	-0.731	0.180	-4.060	4.9E-05	1.5E-03
ENSMUSG00000003345	Csnk1g2	1677.657	-0.431	0.106	-4.057	5.0E-05	1.5E-03
ENSMUSG00000021068	Nin	1496.281	-0.438	0.108	-4.039	5.4E-05	1.7E-03
ENSMUSG00000021508	Cxcl14	884.392	-0.524	0.130	-4.034	5.5E-05	1.7E-03
ENSMUSG00000042064	Myo3b	117.656	-1.193	0.296	-4.033	5.5E-05	1.7E-03
ENSMUSG00000023994	Nfya	647.269	0.731	0.181	4.032	5.5E-05	1.7E-03
ENSMUSG00000022292	Rrm2b	1505.151	0.491	0.122	4.032	5.5E-05	1.7E-03
ENSMUSG00000035486	Plk5	111.229	-1.451	0.360	-4.029	5.6E-05	1.7E-03
ENSMUSG00000035112	Wnk4	60.096	-1.672	0.415	-4.027	5.7E-05	1.7E-03
ENSMUSG00000031292	Cdkl5	5011.214	-0.391	0.097	-4.019	5.9E-05	1.8E-03
ENSMUSG00000036564	Ndrg4	29719.783	-0.320	0.080	-4.017	5.9E-05	1.8E-03
ENSMUSG00000032528	Vipr1	317.972	-0.763	0.190	-4.013	6.0E-05	1.8E-03
ENSMUSG00000028266	Lmo4	1890.001	-0.423	0.105	-4.007	6.2E-05	1.9E-03
ENSMUSG00000037148	Arhgap10	430.113	-0.666	0.166	-4.004	6.2E-05	1.9E-03
ENSMUSG00000053093	Myh7	166.513	-1.043	0.261	-4.001	6.3E-05	1.9E-03
ENSMUSG00000032966	Fkbp1a	7253.944	-0.333	0.083	-4.001	6.3E-05	1.9E-03
ENSMUSG00000022594	Lynx1	3416.650	-1.021	0.255	-4.000	6.3E-05	1.9E-03
ENSMUSG00000031551	Ido1	182.691	-1.091	0.273	-4.000	6.3E-05	1.9E-03
ENSMUSG00000025199	Chuk	1296.621	0.492	0.123	3.994	6.5E-05	1.9E-03
ENSMUSG00000046449	Nexmif	1298.849	0.451	0.113	3.993	6.5E-05	1.9E-03
ENSMUSG00000025795	Rassf3	500.066	-0.652	0.164	-3.988	6.7E-05	2.0E-03
ENSMUSG00000052981	Ube2ql1	1092.312	-0.609	0.153	-3.984	6.8E-05	2.0E-03
ENSMUSG00000026247	Ecel1	271.443	-0.951	0.239	-3.984	6.8E-05	2.0E-03
ENSMUSG00000022099	Dmtn	3471.026	-0.366	0.092	-3.984	6.8E-05	2.0E-03
ENSMUSG00000043857	Mgat5b	1088.934	-0.492	0.124	-3.983	6.8E-05	2.0E-03
ENSMUSG00000037461	Ints7	684.214	-0.550	0.138	-3.983	6.8E-05	2.0E-03
ENSMUSG00000030103	Bhlhe40	719.200	-0.590	0.148	-3.979	6.9E-05	2.0E-03
ENSMUSG00000068747	Sort1	7623.112	-0.358	0.090	-3.979	6.9E-05	2.0E-03
ENSMUSG00000038257	Glra3	324.731	0.757	0.191	3.972	7.1E-05	2.1E-03
ENSMUSG00000033545	Znrf1	2090.269	-0.412	0.104	-3.971	7.2E-05	2.1E-03
ENSMUSG00000026640	Plxna2	2615.494	-0.393	0.099	-3.968	7.2E-05	2.1E-03
ENSMUSG00000069917	Hba-a2	551.497	-0.624	0.158	-3.960	7.5E-05	2.2E-03
ENSMUSG00000032010	Usp2	1002.541	-0.482	0.122	-3.959	7.5E-05	2.2E-03
ENSMUSG00000054405	Dnajc8	1459.282	-0.432	0.109	-3.956	7.6E-05	2.2E-03
ENSMUSG00000050556	Kcnb1	4708.015	-0.362	0.092	-3.949	7.9E-05	2.3E-03
ENSMUSG00000033208	S100b	2329.675	0.425	0.108	3.948	7.9E-05	2.3E-03
ENSMUSG00000001119	Col6a1	1490.135	-0.498	0.126	-3.947	7.9E-05	2.3E-03
ENSMUSG00000031605	Klhl2	4207.560	-0.375	0.095	-3.943	8.0E-05	2.3E-03
ENSMUSG00000024290	Rock1	1421.938	0.443	0.112	3.942	8.1E-05	2.3E-03
ENSMUSG00000049583	Grm5	7283.525	-0.326	0.083	-3.942	8.1E-05	2.3E-03
ENSMUSG00000051111	Sv2c	2995.662	-0.394	0.100	-3.938	8.2E-05	2.3E-03
ENSMUSG00000025272	Tro	3704.941	0.361	0.092	3.938	8.2E-05	2.3E-03
ENSMUSG00000033940	Brk1	1558.303	-0.437	0.111	-3.935	8.3E-05	2.4E-03
ENSMUSG00000002459	Rgs20	752.508	-0.537	0.137	-3.930	8.5E-05	2.4E-03
ENSMUSG00000059136	Olfr539	103.112	-1.790	0.456	-3.927	8.6E-05	2.4E-03
ENSMUSG00000024736	Tmem132a	1198.280	-0.511	0.130	-3.925	8.7E-05	2.4E-03
ENSMUSG00000059003	Grin2a	3243.749	-0.449	0.114	-3.921	8.8E-05	2.5E-03
ENSMUSG00000070462	Tlnrd1	437.585	-0.663	0.169	-3.921	8.8E-05	2.5E-03
ENSMUSG00000034801	Sos2	1162.018	0.509	0.130	3.911	9.2E-05	2.6E-03
ENSMUSG00000010175	Prox1	722.419	0.558	0.143	3.903	9.5E-05	2.7E-03
ENSMUSG00000039954	Stk32a	417.902	-0.722	0.185	-3.899	9.6E-05	2.7E-03
ENSMUSG00000050821	Fam131a	1550.911	-0.512	0.131	-3.899	9.7E-05	2.7E-03
ENSMUSG00000040857	Erf	604.565	-0.602	0.155	-3.891	1.0E-04	2.8E-03
ENSMUSG00000000531	Tamalin	430.417	-0.644	0.165	-3.891	1.0E-04	2.8E-03
ENSMUSG00000020458	Rtn4	12318.769	-0.369	0.095	-3.889	1.0E-04	2.8E-03

ENSMUSG00000022548	Apod	3862.774	0.423	0.109	3.888	1.0E-04	2.8E-03
ENSMUSG00000028152	Tspan5	1957.522	-0.467	0.120	-3.881	1.0E-04	2.9E-03
ENSMUSG00000046093	Hpcal4	12034.760	-0.387	0.100	-3.879	1.1E-04	2.9E-03
ENSMUSG00000029135	Fosl2	406.219	-0.673	0.174	-3.878	1.1E-04	2.9E-03
ENSMUSG00000043463	Rab9b	999.366	0.474	0.122	3.878	1.1E-04	2.9E-03
ENSMUSG00000036273	Lrrk2	2112.547	-1.052	0.272	-3.873	1.1E-04	2.9E-03
ENSMUSG00000058254	Tspan7	15141.400	-0.346	0.090	-3.869	1.1E-04	3.0E-03
ENSMUSG00000034248	Slc25a37	849.163	-0.494	0.128	-3.856	1.2E-04	3.1E-03
ENSMUSG00000035226	Rims4	1000.864	-0.513	0.133	-3.855	1.2E-04	3.1E-03
ENSMUSG00000038296	Galnt18	346.841	-0.726	0.188	-3.856	1.2E-04	3.1E-03
ENSMUSG00000026979	Psd4	42.470	2.030	0.527	3.855	1.2E-04	3.1E-03
ENSMUSG00000040289	Hey1	715.950	-0.524	0.136	-3.852	1.2E-04	3.2E-03
ENSMUSG00000043388	Tmem130	3560.192	0.366	0.095	3.849	1.2E-04	3.2E-03
ENSMUSG00000017417	Plxdc1	245.905	-0.827	0.215	-3.844	1.2E-04	3.3E-03
ENSMUSG00000024479	Mal2	2599.612	-0.379	0.099	-3.842	1.2E-04	3.3E-03
ENSMUSG00000045038	Prkce	8250.977	-0.357	0.093	-3.841	1.2E-04	3.3E-03
ENSMUSG00000032500	Dclk3	1385.662	-0.490	0.128	-3.833	1.3E-04	3.4E-03
ENSMUSG00000035864	Syt1	22637.561	-0.384	0.100	-3.830	1.3E-04	3.4E-03
ENSMUSG00000019194	Scn1b	2475.312	-0.475	0.124	-3.823	1.3E-04	3.5E-03
ENSMUSG00000068037	Mas1	135.291	-1.412	0.369	-3.823	1.3E-04	3.5E-03
ENSMUSG00000020015	Cdk17	4349.855	-0.357	0.093	-3.822	1.3E-04	3.5E-03
ENSMUSG00000050473	Slc35d3	507.142	-0.673	0.176	-3.815	1.4E-04	3.6E-03
ENSMUSG00000036915	Kirrel2	112.483	-1.242	0.326	-3.806	1.4E-04	3.7E-03
ENSMUSG00000024502	Jakmip2	1338.890	-0.440	0.116	-3.802	1.4E-04	3.8E-03
ENSMUSG00000053470	Kdm3a	1245.046	-0.432	0.114	-3.801	1.4E-04	3.8E-03
ENSMUSG00000021273	Fdft1	1107.334	-0.533	0.140	-3.801	1.4E-04	3.8E-03
ENSMUSG00000027955	Gask1b	211.858	-0.966	0.255	-3.796	1.5E-04	3.9E-03
ENSMUSG00000058135	Gstm1	1877.041	-0.402	0.106	-3.795	1.5E-04	3.9E-03
ENSMUSG00000032343	Impg1	34.734	-2.092	0.552	-3.792	1.5E-04	3.9E-03
ENSMUSG00000039126	Prune2	2451.712	0.368	0.097	3.789	1.5E-04	3.9E-03
ENSMUSG00000036202	Rif1	943.395	0.472	0.125	3.788	1.5E-04	3.9E-03
ENSMUSG00000045871	Slitrk6	205.982	1.024	0.271	3.786	1.5E-04	4.0E-03
ENSMUSG00000009216	Fam163b	1097.000	-0.589	0.156	-3.785	1.5E-04	4.0E-03
ENSMUSG00000030465	Psd3	11917.285	-0.327	0.086	-3.782	1.6E-04	4.0E-03
ENSMUSG00000038582	Pptc7	1837.306	-0.391	0.103	-3.782	1.6E-04	4.0E-03
ENSMUSG00000040943	Tet2	2113.617	0.448	0.119	3.778	1.6E-04	4.1E-03
ENSMUSG00000044835	Ankrd45	1526.059	0.413	0.109	3.777	1.6E-04	4.1E-03
ENSMUSG00000025920	Stau2	3023.263	-0.378	0.100	-3.770	1.6E-04	4.2E-03
ENSMUSG00000072572	Slc39a2	102.977	-1.206	0.320	-3.769	1.6E-04	4.2E-03
ENSMUSG00000034310	Tmem132d	378.546	-0.804	0.213	-3.767	1.7E-04	4.2E-03
ENSMUSG00000021140	Pcnx	3116.664	0.364	0.097	3.764	1.7E-04	4.3E-03
ENSMUSG00000071234	Syndig1l	1679.071	-1.176	0.313	-3.762	1.7E-04	4.3E-03
ENSMUSG00000054387	Mdm4	2921.390	0.378	0.100	3.762	1.7E-04	4.3E-03
ENSMUSG00000028059	Arhgef2	2581.079	-0.386	0.103	-3.760	1.7E-04	4.3E-03
ENSMUSG00000010505	Myt1	374.911	0.775	0.206	3.759	1.7E-04	4.3E-03
ENSMUSG00000029095	Ablim2	3371.780	-0.435	0.116	-3.755	1.7E-04	4.4E-03
ENSMUSG00000035735	Dagla	1156.362	-0.448	0.119	-3.752	1.8E-04	4.4E-03
ENSMUSG00000041771	Slc24a4	531.740	-0.600	0.160	-3.751	1.8E-04	4.4E-03
ENSMUSG00000019947	Arid5b	964.853	0.514	0.137	3.750	1.8E-04	4.4E-03
ENSMUSG00000051403	Ppp1r37	1439.607	-0.409	0.109	-3.744	1.8E-04	4.5E-03
ENSMUSG00000075012	Fjx1	572.998	-0.606	0.162	-3.745	1.8E-04	4.5E-03
ENSMUSG00000062397	Zfp706	3127.029	-0.412	0.110	-3.743	1.8E-04	4.6E-03
ENSMUSG00000057110	Cntrl	361.752	0.688	0.184	3.741	1.8E-04	4.6E-03
ENSMUSG00000010663	Fads1	3508.943	-0.381	0.102	-3.740	1.8E-04	4.6E-03
ENSMUSG00000030747	Dgat2	991.403	-0.483	0.129	-3.738	1.9E-04	4.6E-03
ENSMUSG00000038540	Tmc3	55.326	2.005	0.536	3.737	1.9E-04	4.6E-03

ENSMUSG00000039714	Cplx3	72.511	-1.909	0.511	-3.734	1.9E-04	4.7E-03
ENSMUSG00000035390	Brsk1	4707.548	-0.341	0.091	-3.731	1.9E-04	4.7E-03
ENSMUSG00000030518	Fam189a1	727.558	-0.567	0.152	-3.729	1.9E-04	4.7E-03
ENSMUSG00000019960	Dusp6	529.952	-0.566	0.152	-3.729	1.9E-04	4.7E-03
ENSMUSG00000063804	Lin28b	397.480	0.691	0.185	3.728	1.9E-04	4.7E-03
ENSMUSG00000049577	Zfpm1	300.818	-0.746	0.200	-3.727	1.9E-04	4.8E-03
ENSMUSG00000061462	Obscn	80.721	-1.352	0.363	-3.722	2.0E-04	4.8E-03
ENSMUSG00000027210	Meis2	6457.630	-0.427	0.115	-3.719	2.0E-04	4.9E-03
ENSMUSG00000036093	Arl5a	2448.443	0.364	0.098	3.712	2.1E-04	5.0E-03
ENSMUSG00000040972	Igsf21	310.041	-0.831	0.225	-3.700	2.2E-04	5.3E-03
ENSMUSG00000036676	Tmtc3	587.755	0.653	0.177	3.690	2.2E-04	5.5E-03
ENSMUSG00000039601	Rcan2	4828.601	-0.338	0.092	-3.689	2.3E-04	5.5E-03
ENSMUSG00000027206	Cops2	2627.412	0.401	0.109	3.686	2.3E-04	5.5E-03
ENSMUSG00000056069	Otulinl	414.862	-0.633	0.172	-3.685	2.3E-04	5.5E-03
ENSMUSG00000030120	Mlf2	4287.119	-0.322	0.088	-3.681	2.3E-04	5.6E-03
ENSMUSG00000025370	Cdh9	538.717	-0.593	0.161	-3.681	2.3E-04	5.6E-03
ENSMUSG00000059974	Ntm	4276.012	-0.391	0.106	-3.679	2.3E-04	5.6E-03
ENSMUSG00000019254	Ppp1r12c	1151.790	-0.430	0.117	-3.678	2.4E-04	5.7E-03
ENSMUSG00000029419	Ajm1	1359.614	-0.443	0.120	-3.676	2.4E-04	5.7E-03
ENSMUSG00000020393	Kremen1	387.117	-0.631	0.172	-3.673	2.4E-04	5.7E-03
ENSMUSG00000053199	Arhgap20	2541.321	-0.359	0.098	-3.672	2.4E-04	5.8E-03
ENSMUSG00000000631	Myo18a	2622.998	-0.399	0.109	-3.665	2.5E-04	5.9E-03
ENSMUSG00000020435	Osbp2	1267.765	-0.492	0.134	-3.662	2.5E-04	6.0E-03
ENSMUSG00000003872	Lin7b	215.145	-0.813	0.222	-3.661	2.5E-04	6.0E-03
ENSMUSG00000056629	Fkbp2	832.518	-0.484	0.132	-3.661	2.5E-04	6.0E-03
ENSMUSG00000040543	Pitpnm3	1076.748	-0.621	0.170	-3.657	2.6E-04	6.1E-03
ENSMUSG00000002985	Apoe	15882.138	-0.286	0.078	-3.654	2.6E-04	6.1E-03
ENSMUSG00000041205	Map6d1	893.734	-0.482	0.132	-3.653	2.6E-04	6.1E-03
ENSMUSG00000021647	Cartpt	57.373	-1.847	0.507	-3.643	2.7E-04	6.4E-03
ENSMUSG00000032773	Chrm1	1911.431	-0.381	0.105	-3.640	2.7E-04	6.4E-03
ENSMUSG00000019027	Dnah1	171.814	-1.150	0.316	-3.640	2.7E-04	6.4E-03
ENSMUSG00000051335	Gfod1	1471.439	-0.433	0.119	-3.640	2.7E-04	6.4E-03
ENSMUSG00000070327	Rnf213	455.133	0.594	0.163	3.633	2.8E-04	6.6E-03
ENSMUSG00000021431	Snrnp48	954.764	0.509	0.140	3.631	2.8E-04	6.6E-03
ENSMUSG00000036533	Cdc42ep3	425.409	-0.676	0.186	-3.630	2.8E-04	6.6E-03
ENSMUSG00000050608	Micos10	851.253	-0.471	0.130	-3.628	2.9E-04	6.7E-03
ENSMUSG00000022763	Aifm3	503.180	-0.690	0.191	-3.623	2.9E-04	6.8E-03
ENSMUSG00000021684	Pde8b	2167.086	-0.358	0.099	-3.620	2.9E-04	6.8E-03
ENSMUSG00000028788	Ptp4a2	3412.463	-0.337	0.093	-3.616	3.0E-04	6.9E-03
ENSMUSG00000061288	Taok3	1119.305	0.453	0.125	3.615	3.0E-04	7.0E-03
ENSMUSG00000118608	Gm4988	245.521	0.912	0.252	3.614	3.0E-04	7.0E-03
ENSMUSG00000041669	Prima1	89.875	-1.204	0.333	-3.611	3.0E-04	7.0E-03
ENSMUSG00000036438	Calm2	24965.715	-0.283	0.078	-3.611	3.0E-04	7.0E-03
ENSMUSG00000064341	mt-Nd1	#########	-0.287	0.079	-3.611	3.1E-04	7.0E-03
ENSMUSG00000033066	Gas7	9997.750	-0.295	0.082	-3.607	3.1E-04	7.1E-03
ENSMUSG00000037868	Egr2	70.113	-1.587	0.441	-3.601	3.2E-04	7.3E-03
ENSMUSG00000049470	Aff4	6491.974	0.320	0.089	3.599	3.2E-04	7.3E-03
ENSMUSG00000025533	Asl	227.232	0.830	0.231	3.594	3.3E-04	7.4E-03
ENSMUSG00000024213	Nudt3	3824.357	-0.333	0.093	-3.592	3.3E-04	7.5E-03
ENSMUSG00000033427	Upb1	40.002	-1.931	0.538	-3.591	3.3E-04	7.5E-03
ENSMUSG00000031382	Asb11	74.079	-1.426	0.397	-3.589	3.3E-04	7.5E-03
ENSMUSG00000041444	Arhgap32	7736.315	-0.300	0.083	-3.588	3.3E-04	7.6E-03
ENSMUSG00000027016	Zfp385b	937.845	-0.459	0.128	-3.585	3.4E-04	7.6E-03
ENSMUSG00000020681	Ace	320.073	-0.711	0.198	-3.585	3.4E-04	7.6E-03
ENSMUSG00000052133	Sema5b	313.456	-0.764	0.213	-3.583	3.4E-04	7.7E-03
ENSMUSG00000020176	Grb10	1382.010	0.399	0.111	3.581	3.4E-04	7.7E-03

ENSMUSG00000034771	Tle2	129.881	-1.025	0.286	-3.579	3.4E-04	7.8E-03
ENSMUSG00000022661	Cd200	1935.504	0.398	0.111	3.576	3.5E-04	7.8E-03
ENSMUSG00000021680	Crhbp	83.385	-1.320	0.369	-3.576	3.5E-04	7.8E-03
ENSMUSG00000052302	Tbc1d30	1287.558	-0.454	0.127	-3.575	3.5E-04	7.8E-03
ENSMUSG00000029999	Tgfa	2070.772	-0.463	0.130	-3.573	3.5E-04	7.9E-03
ENSMUSG00000025170	Rab40b	522.044	-0.587	0.164	-3.572	3.5E-04	7.9E-03
ENSMUSG00000034936	Arl4d	228.769	-0.772	0.216	-3.572	3.5E-04	7.9E-03
ENSMUSG00000043013	Onecut1	19.801	3.060	0.857	3.571	3.6E-04	7.9E-03
ENSMUSG00000032883	Acsl3	4093.067	-0.419	0.117	-3.569	3.6E-04	8.0E-03
ENSMUSG00000035722	Abca7	279.810	0.727	0.204	3.568	3.6E-04	8.0E-03
ENSMUSG00000071658	Gng3	2277.044	0.358	0.100	3.565	3.6E-04	8.1E-03
ENSMUSG00000060962	Dmkn	58.057	-1.489	0.418	-3.562	3.7E-04	8.1E-03
ENSMUSG00000037465	Klf10	329.090	-0.723	0.203	-3.562	3.7E-04	8.1E-03
ENSMUSG00000053716	Dusp7	719.627	-0.484	0.136	-3.559	3.7E-04	8.2E-03
ENSMUSG00000052373	Mpp3	535.417	-0.569	0.160	-3.556	3.8E-04	8.3E-03
ENSMUSG00000030092	Cntn6	538.156	0.596	0.168	3.550	3.8E-04	8.5E-03
ENSMUSG00000038481	Cdk19	2303.535	-0.350	0.099	-3.548	3.9E-04	8.5E-03
ENSMUSG00000019828	Grm1	1455.115	-0.419	0.118	-3.543	3.9E-04	8.6E-03
ENSMUSG00000036052	Dnajb5	1404.977	-0.438	0.123	-3.544	3.9E-04	8.6E-03
ENSMUSG00000000792	Slc5a5	192.019	0.860	0.243	3.544	3.9E-04	8.6E-03
ENSMUSG00000047205	Dusp18	448.915	-0.605	0.171	-3.541	4.0E-04	8.7E-03
ENSMUSG00000051351	Zfp46	896.687	-0.455	0.129	-3.539	4.0E-04	8.7E-03
ENSMUSG00000041308	Sntb2	651.614	-0.544	0.154	-3.538	4.0E-04	8.8E-03
ENSMUSG00000073591	Pcdhb22	439.331	0.594	0.168	3.536	4.1E-04	8.8E-03
ENSMUSG00000026885	Ttll11	467.529	-0.575	0.163	-3.532	4.1E-04	8.9E-03
ENSMUSG00000028800	Hdac1	520.121	0.551	0.156	3.528	4.2E-04	9.1E-03
ENSMUSG00000021990	Spata13	661.937	-0.518	0.147	-3.524	4.2E-04	9.2E-03
ENSMUSG00000096054	Syne1	11626.897	-0.286	0.081	-3.521	4.3E-04	9.3E-03
ENSMUSG00000061607	Mdc1	569.290	0.547	0.155	3.518	4.3E-04	9.4E-03
ENSMUSG00000029861	Fam131b	2249.470	-0.365	0.104	-3.510	4.5E-04	9.6E-03
ENSMUSG00000062110	Scfd2	271.546	-0.711	0.202	-3.510	4.5E-04	9.6E-03
ENSMUSG00000030844	Rgs10	336.113	-0.642	0.183	-3.510	4.5E-04	9.6E-03
ENSMUSG00000063430	Wscd2	957.614	-0.476	0.136	-3.508	4.5E-04	9.7E-03
ENSMUSG00000070498	Tmem132b	3111.697	-0.333	0.095	-3.504	4.6E-04	9.8E-03
ENSMUSG00000053550	Shisa7	3053.954	-0.330	0.094	-3.502	4.6E-04	9.9E-03
ENSMUSG00000065954	Tacc1	4769.683	-0.318	0.091	-3.500	4.7E-04	9.9E-03
ENSMUSG00000015501	Hivep2	4960.499	-0.320	0.092	-3.492	4.8E-04	1.0E-02
ENSMUSG00000046159	Chrm3	450.910	-0.750	0.215	-3.488	4.9E-04	1.0E-02
ENSMUSG00000100862	Gm10925	15951.299	-3.197	0.917	-3.486	4.9E-04	1.0E-02
ENSMUSG00000039735	Fnbp1l	4570.658	-0.329	0.094	-3.481	5.0E-04	1.1E-02
ENSMUSG00000041112	Elmo1	2386.245	-0.357	0.103	-3.475	5.1E-04	1.1E-02
ENSMUSG00000021846	Peli2	676.706	-0.546	0.157	-3.475	5.1E-04	1.1E-02
ENSMUSG00000009291	Pttg1ip	989.816	-0.423	0.122	-3.472	5.2E-04	1.1E-02
ENSMUSG00000047976	Kcna1	1481.628	-0.407	0.117	-3.472	5.2E-04	1.1E-02
ENSMUSG00000033590	Myo5c	52.202	-1.591	0.458	-3.471	5.2E-04	1.1E-02
ENSMUSG00000031749	St3gal2	1780.393	-0.373	0.107	-3.469	5.2E-04	1.1E-02
ENSMUSG00000030711	Sult1a1	44.398	-1.683	0.486	-3.467	5.3E-04	1.1E-02
ENSMUSG00000005973	Rcn1	927.005	-0.474	0.137	-3.460	5.4E-04	1.1E-02
ENSMUSG00000030806	Stx1b	8232.557	-0.294	0.085	-3.454	5.5E-04	1.2E-02
ENSMUSG00000035021	Baz1a	137.934	-1.096	0.317	-3.455	5.5E-04	1.2E-02
ENSMUSG00000020680	Taf15	1298.039	0.448	0.130	3.453	5.5E-04	1.2E-02
ENSMUSG00000040407	Akap9	4762.835	-0.360	0.104	-3.453	5.5E-04	1.2E-02
ENSMUSG00000039713	Plekhg5	520.129	-0.611	0.177	-3.452	5.6E-04	1.2E-02
ENSMUSG00000026104	Stat1	562.585	0.529	0.153	3.451	5.6E-04	1.2E-02
ENSMUSG00000042323	Pbrm1	1828.995	0.363	0.105	3.451	5.6E-04	1.2E-02
ENSMUSG00000040724	Kcna2	5837.554	-0.359	0.104	-3.449	5.6E-04	1.2E-02

ENSMUSG00000039145	Camk1d	3408.826	-0.316	0.092	-3.447	5.7E-04	1.2E-02
ENSMUSG00000070576	Mn1	679.936	-0.515	0.150	-3.446	5.7E-04	1.2E-02
ENSMUSG00000047495	Dlgap2	1170.267	-0.442	0.128	-3.443	5.7E-04	1.2E-02
ENSMUSG00000050751	Pgbd5	1639.274	-0.393	0.114	-3.436	5.9E-04	1.2E-02
ENSMUSG00000022723	Crybg3	729.923	-0.506	0.147	-3.434	5.9E-04	1.2E-02
ENSMUSG00000038780	Smurf1	871.145	-0.439	0.128	-3.433	6.0E-04	1.2E-02
ENSMUSG00000020674	Pxdn	1313.254	-0.462	0.135	-3.432	6.0E-04	1.2E-02
ENSMUSG00000069911	Insyn2b	190.200	0.834	0.243	3.430	6.0E-04	1.2E-02
ENSMUSG00000023046	Igfbp6	133.095	-0.949	0.277	-3.427	6.1E-04	1.2E-02
ENSMUSG00000096146	Kcnj11	469.225	-0.568	0.166	-3.427	6.1E-04	1.2E-02
ENSMUSG00000087408	Cers1	827.957	-0.443	0.129	-3.425	6.1E-04	1.2E-02
ENSMUSG00000073424	Cyp4f15	120.555	-1.019	0.298	-3.423	6.2E-04	1.3E-02
ENSMUSG00000020893	Per1	775.389	-0.470	0.138	-3.414	6.4E-04	1.3E-02
ENSMUSG00000031328	Flna	1693.329	-0.402	0.118	-3.412	6.5E-04	1.3E-02
ENSMUSG00000013275	Slc41a1	1098.347	-0.444	0.130	-3.412	6.5E-04	1.3E-02
ENSMUSG00000041115	Iqsec2	2257.782	-0.350	0.103	-3.405	6.6E-04	1.3E-02
ENSMUSG00000044254	Pcsk9	61.345	-1.377	0.405	-3.403	6.7E-04	1.3E-02
ENSMUSG00000018474	Chd3	15212.231	0.340	0.100	3.402	6.7E-04	1.3E-02
ENSMUSG00000021125	Arg2	77.420	-1.227	0.361	-3.402	6.7E-04	1.3E-02
ENSMUSG00000037104	Socs5	2788.246	-0.348	0.102	-3.401	6.7E-04	1.3E-02
ENSMUSG00000035268	Pkig	574.486	-0.503	0.148	-3.397	6.8E-04	1.4E-02
ENSMUSG00000040699	Limd2	476.335	-0.548	0.161	-3.397	6.8E-04	1.4E-02
ENSMUSG00000029817	Tra2a	2622.196	0.377	0.111	3.393	6.9E-04	1.4E-02
ENSMUSG00000072969	Armcx5	800.109	0.466	0.137	3.393	6.9E-04	1.4E-02
ENSMUSG00000045009	Prrt3	363.991	-0.642	0.190	-3.390	7.0E-04	1.4E-02
ENSMUSG00000094065	Ccl21d	33.102	-7.822	2.310	-3.387	7.1E-04	1.4E-02
ENSMUSG00000022095	Fam160b2	550.273	-0.512	0.151	-3.387	7.1E-04	1.4E-02
ENSMUSG00000050963	Kcns2	871.796	-0.468	0.138	-3.384	7.2E-04	1.4E-02
ENSMUSG00000047747	Rnf150	2339.560	-0.337	0.100	-3.382	7.2E-04	1.4E-02
ENSMUSG00000037872	Ackr1	316.374	-0.663	0.196	-3.380	7.2E-04	1.4E-02
ENSMUSG00000043154	Ppp2r3a	1453.997	0.376	0.111	3.378	7.3E-04	1.4E-02
ENSMUSG00000058258	Idi1	1035.880	-0.515	0.153	-3.372	7.5E-04	1.5E-02
ENSMUSG00000049804	Armcx4	1998.566	0.341	0.101	3.367	7.6E-04	1.5E-02
ENSMUSG00000055053	Nfic	1661.050	-0.412	0.122	-3.366	7.6E-04	1.5E-02
ENSMUSG00000016763	Scube1	282.867	-0.927	0.276	-3.362	7.7E-04	1.5E-02
ENSMUSG00000030209	Grin2b	10114.171	-0.314	0.093	-3.358	7.9E-04	1.5E-02
ENSMUSG00000027002	Nckap1	10935.696	-0.297	0.088	-3.357	7.9E-04	1.5E-02
ENSMUSG00000036565	Ttyh3	2056.913	-0.365	0.109	-3.352	8.0E-04	1.6E-02
ENSMUSG00000118668	Rps6ka4	449.092	-0.558	0.166	-3.352	8.0E-04	1.6E-02
ENSMUSG00000023017	Asic1	1191.585	-0.387	0.115	-3.352	8.0E-04	1.6E-02
ENSMUSG00000027469	Tpx2	106.032	1.124	0.336	3.348	8.2E-04	1.6E-02
ENSMUSG00000044176	Spink10	135.951	0.929	0.278	3.346	8.2E-04	1.6E-02
ENSMUSG00000074923	Pak6	871.460	-0.429	0.128	-3.340	8.4E-04	1.6E-02
ENSMUSG00000000305	Cdh4	480.009	-0.573	0.172	-3.339	8.4E-04	1.6E-02
ENSMUSG00000028039	Efna3	526.311	-0.520	0.156	-3.335	8.5E-04	1.7E-02
ENSMUSG00000037257	Aagab	684.809	0.496	0.149	3.331	8.7E-04	1.7E-02
ENSMUSG00000036777	Anln	1294.964	0.420	0.126	3.328	8.8E-04	1.7E-02
ENSMUSG00000027429	Sec23b	775.526	0.445	0.134	3.325	8.8E-04	1.7E-02
ENSMUSG00000007987	Ift22	678.706	0.478	0.144	3.323	8.9E-04	1.7E-02
ENSMUSG00000025656	Arhgef9	12534.224	-0.269	0.081	-3.317	9.1E-04	1.7E-02
ENSMUSG00000024524	Gnal	12264.319	-0.329	0.099	-3.314	9.2E-04	1.8E-02
ENSMUSG00000067369	Trmt2b	404.395	0.578	0.174	3.312	9.3E-04	1.8E-02
ENSMUSG00000004031	Brinp2	1008.424	-0.423	0.128	-3.312	9.3E-04	1.8E-02
ENSMUSG00000027849	Syt6	1811.454	-0.423	0.128	-3.311	9.3E-04	1.8E-02
ENSMUSG00000009013	Dynll1	2135.490	-0.375	0.113	-3.308	9.4E-04	1.8E-02
ENSMUSG00000031762	Mt2	690.165	-0.501	0.152	-3.306	9.5E-04	1.8E-02

ENSMUSG00000018412	Kansl1	1870.926	0.371	0.112	3.303	9.6E-04	1.8E-02
ENSMUSG00000038495	Otud7b	1718.489	0.371	0.112	3.300	9.7E-04	1.8E-02
ENSMUSG00000042743	Sgtb	2407.999	-0.354	0.107	-3.299	9.7E-04	1.8E-02
ENSMUSG00000030850	Ate1	1764.440	0.343	0.104	3.299	9.7E-04	1.8E-02
ENSMUSG00000028931	Kcnab2	4527.780	-0.306	0.093	-3.296	9.8E-04	1.9E-02
ENSMUSG00000024501	Dpysl3	1039.317	0.396	0.120	3.293	9.9E-04	1.9E-02
ENSMUSG00000060126	Tpt1	4196.286	-0.293	0.089	-3.293	9.9E-04	1.9E-02
ENSMUSG00000029153	Ociad2	1125.460	-0.427	0.130	-3.293	9.9E-04	1.9E-02
ENSMUSG00000017718	Afmid	36.858	2.366	0.719	3.291	1.0E-03	1.9E-02
ENSMUSG00000042942	Greb1l	209.937	0.760	0.231	3.290	1.0E-03	1.9E-02
ENSMUSG00000004383	Large1	1624.778	-0.413	0.126	-3.282	1.0E-03	1.9E-02
ENSMUSG00000057130	Txnl4a	258.401	-0.675	0.206	-3.281	1.0E-03	1.9E-02
ENSMUSG00000022296	Baalc	2460.638	-0.418	0.127	-3.279	1.0E-03	2.0E-02
ENSMUSG00000051726	Kcnf1	1259.484	-0.463	0.141	-3.277	1.0E-03	2.0E-02
ENSMUSG00000022801	Lrch3	939.473	0.420	0.128	3.277	1.1E-03	2.0E-02
ENSMUSG00000015291	Gdi1	9859.639	0.287	0.088	3.275	1.1E-03	2.0E-02
ENSMUSG00000093954	Gm16867	362.376	-0.610	0.186	-3.275	1.1E-03	2.0E-02
ENSMUSG00000074736	Syndig1	611.757	-0.492	0.150	-3.275	1.1E-03	2.0E-02
ENSMUSG00000020846	Rflnb	229.405	-0.886	0.271	-3.275	1.1E-03	2.0E-02
ENSMUSG00000028933	Xrcc2	156.820	0.916	0.280	3.275	1.1E-03	2.0E-02
ENSMUSG00000041556	Fbxo2	489.841	-0.523	0.160	-3.273	1.1E-03	2.0E-02
ENSMUSG00000036295	Lrrn3	1637.189	0.383	0.117	3.273	1.1E-03	2.0E-02
ENSMUSG00000027327	1700037H04Rik	459.137	-0.531	0.162	-3.272	1.1E-03	2.0E-02
ENSMUSG00000060780	Lrrtm1	631.111	-0.487	0.149	-3.271	1.1E-03	2.0E-02
ENSMUSG00000040631	Dok4	285.369	-0.660	0.202	-3.270	1.1E-03	2.0E-02
ENSMUSG00000055254	Ntrk2	11505.230	-0.313	0.096	-3.269	1.1E-03	2.0E-02
ENSMUSG00000021763	Cspg4b	123.954	1.055	0.323	3.268	1.1E-03	2.0E-02
ENSMUSG00000006435	Neurl1a	2537.972	-0.335	0.103	-3.263	1.1E-03	2.0E-02
ENSMUSG00000022521	Crebbp	1964.762	0.332	0.102	3.263	1.1E-03	2.0E-02
ENSMUSG00000020451	Limk2	1625.756	0.349	0.107	3.261	1.1E-03	2.0E-02
ENSMUSG00000034744	Nagk	424.951	0.555	0.170	3.259	1.1E-03	2.0E-02
ENSMUSG00000117286	Gm1043	726.890	-0.450	0.138	-3.258	1.1E-03	2.1E-02
ENSMUSG00000001138	Cnnm3	506.686	0.585	0.180	3.256	1.1E-03	2.1E-02
ENSMUSG00000022883	Robo1	1487.806	0.387	0.119	3.255	1.1E-03	2.1E-02
ENSMUSG00000040105	Plpp6	763.135	-0.495	0.152	-3.253	1.1E-03	2.1E-02
ENSMUSG00000050447	Lypd6	437.850	-0.570	0.175	-3.254	1.1E-03	2.1E-02
ENSMUSG00000038354	Ankrd35	173.354	-0.873	0.268	-3.254	1.1E-03	2.1E-02
ENSMUSG00000026360	Rgs2	1647.451	-0.455	0.140	-3.251	1.1E-03	2.1E-02
ENSMUSG00000021061	Sptb	4634.937	-0.315	0.097	-3.250	1.2E-03	2.1E-02
ENSMUSG00000041592	Sdk2	267.672	-0.777	0.239	-3.249	1.2E-03	2.1E-02
ENSMUSG00000053253	Ndfip2	1913.866	-0.380	0.117	-3.243	1.2E-03	2.1E-02
ENSMUSG00000045903	Npas4	236.252	-0.743	0.229	-3.241	1.2E-03	2.1E-02
ENSMUSG00000049792	Bag5	931.928	-0.419	0.129	-3.240	1.2E-03	2.2E-02
ENSMUSG00000024597	Slc12a2	3014.766	0.318	0.098	3.238	1.2E-03	2.2E-02
ENSMUSG00000027375	Mal	5141.334	-0.328	0.102	-3.234	1.2E-03	2.2E-02
ENSMUSG00000033487	Fndc3a	2053.689	0.367	0.114	3.229	1.2E-03	2.2E-02
ENSMUSG00000024544	Ldlrad4	481.950	-0.538	0.167	-3.228	1.2E-03	2.2E-02
ENSMUSG00000015944	Castor2	2856.164	0.323	0.100	3.227	1.2E-03	2.2E-02
ENSMUSG00000026740	Dnajc1	529.832	-0.537	0.166	-3.224	1.3E-03	2.3E-02
ENSMUSG00000029720	Gm20605	199.863	-0.798	0.248	-3.221	1.3E-03	2.3E-02
ENSMUSG00000001313	Rnd2	1038.523	-0.391	0.121	-3.219	1.3E-03	2.3E-02
ENSMUSG00000042502	Cd2bp2	1377.251	0.377	0.117	3.217	1.3E-03	2.3E-02
ENSMUSG00000075376	Rc3h2	2694.483	0.347	0.108	3.216	1.3E-03	2.3E-02
ENSMUSG00000029309	Sparcl1	24391.051	-0.312	0.097	-3.214	1.3E-03	2.3E-02
ENSMUSG00000038970	Lmtk2	2919.913	-0.300	0.093	-3.212	1.3E-03	2.3E-02
ENSMUSG00000034755	Pcdh11x	977.369	0.445	0.139	3.210	1.3E-03	2.4E-02

ENSMUSG00000050965	Prkca	3451.687	-0.423	0.132	-3.209	1.3E-03	2.4E-02
ENSMUSG00000027223	Mapk8ip1	4792.021	-0.283	0.088	-3.207	1.3E-03	2.4E-02
ENSMUSG00000001687	Ubl3	3182.053	-0.327	0.102	-3.205	1.4E-03	2.4E-02
ENSMUSG00000040836	Gpr161	241.722	0.699	0.218	3.203	1.4E-03	2.4E-02
ENSMUSG00000073411	H2-D1	474.457	0.547	0.171	3.199	1.4E-03	2.4E-02
ENSMUSG00000001467	Cyp51	1008.253	-0.461	0.144	-3.198	1.4E-03	2.4E-02
ENSMUSG00000015970	Chdh	193.126	0.896	0.280	3.198	1.4E-03	2.4E-02
ENSMUSG00000064225	Paqr9	1311.639	-0.378	0.118	-3.197	1.4E-03	2.4E-02
ENSMUSG00000021782	Dlg5	713.318	0.435	0.136	3.192	1.4E-03	2.5E-02
ENSMUSG00000029624	Ptcd1	397.648	-0.556	0.174	-3.191	1.4E-03	2.5E-02
ENSMUSG00000059146	Ntrk3	4406.552	-0.290	0.091	-3.187	1.4E-03	2.5E-02
ENSMUSG00000020716	Nf1	3455.583	0.304	0.095	3.184	1.5E-03	2.5E-02
ENSMUSG00000046020	Pofut1	581.033	0.516	0.162	3.180	1.5E-03	2.6E-02
ENSMUSG00000047181	Samd14	537.258	0.520	0.164	3.177	1.5E-03	2.6E-02
ENSMUSG00000020950	Foxg1	1856.309	-0.340	0.107	-3.171	1.5E-03	2.6E-02
ENSMUSG00000102543	Pcdhgc5	2249.540	-0.324	0.102	-3.170	1.5E-03	2.6E-02
ENSMUSG00000037972	Snn	3098.121	-0.305	0.096	-3.170	1.5E-03	2.6E-02
ENSMUSG00000058248	Kcnh1	1308.925	-0.396	0.125	-3.169	1.5E-03	2.6E-02
ENSMUSG00000007097	Atp1a2	16305.484	-0.291	0.092	-3.165	1.6E-03	2.7E-02
ENSMUSG00000039046	Usp6nl	724.960	0.451	0.143	3.163	1.6E-03	2.7E-02
ENSMUSG00000025937	Lactb2	559.790	0.470	0.149	3.162	1.6E-03	2.7E-02
ENSMUSG00000030096	Slc6a6	4042.523	-0.295	0.093	-3.161	1.6E-03	2.7E-02
ENSMUSG00000020580	Rock2	5793.270	-0.272	0.086	-3.161	1.6E-03	2.7E-02
ENSMUSG00000041078	Grid1	1272.226	-0.365	0.116	-3.161	1.6E-03	2.7E-02
ENSMUSG00000032518	Rpsa	5665.264	-0.273	0.086	-3.158	1.6E-03	2.7E-02
ENSMUSG00000037416	Dmxl1	2260.732	0.335	0.106	3.157	1.6E-03	2.7E-02
ENSMUSG00000044912	Syt16	1187.960	-0.410	0.130	-3.155	1.6E-03	2.7E-02
ENSMUSG00000022055	Nefl	5768.545	-0.330	0.105	-3.155	1.6E-03	2.7E-02
ENSMUSG00000033910	Gucy1a1	2989.950	-0.297	0.094	-3.154	1.6E-03	2.8E-02
ENSMUSG00000032402	Smad3	946.110	-0.400	0.127	-3.153	1.6E-03	2.8E-02
ENSMUSG00000020135	Apc2	2002.908	0.335	0.106	3.153	1.6E-03	2.8E-02
ENSMUSG00000070047	Fat1	1813.266	0.341	0.108	3.150	1.6E-03	2.8E-02
ENSMUSG00000036099	Vezt	1895.060	0.377	0.120	3.149	1.6E-03	2.8E-02
ENSMUSG00000069919	Hba-a1	520.300	-0.570	0.181	-3.146	1.7E-03	2.8E-02
ENSMUSG00000031441	Atp11a	1921.282	0.343	0.109	3.144	1.7E-03	2.8E-02
ENSMUSG00000068696	Gpr88	11488.854	-0.837	0.266	-3.143	1.7E-03	2.8E-02
ENSMUSG00000092329		320.117	9.726	3.096	3.141	1.7E-03	2.8E-02
ENSMUSG00000042804	Gpr153	267.217	-0.835	0.266	-3.140	1.7E-03	2.9E-02
ENSMUSG00000006575	Rundc3a	2580.549	-0.308	0.098	-3.139	1.7E-03	2.9E-02
ENSMUSG00000038936	Sccpdh	1326.690	0.363	0.116	3.138	1.7E-03	2.9E-02
ENSMUSG00000022332	Khdrbs3	1201.944	-0.465	0.148	-3.135	1.7E-03	2.9E-02
ENSMUSG00000038291	Snx25	1150.811	-0.371	0.118	-3.135	1.7E-03	2.9E-02
ENSMUSG00000022358	Fbxo32	969.609	-0.386	0.123	-3.134	1.7E-03	2.9E-02
ENSMUSG00000025597	Klhl4	631.696	0.495	0.158	3.133	1.7E-03	2.9E-02
ENSMUSG00000025318	Jph3	3420.653	-0.285	0.091	-3.128	1.8E-03	2.9E-02
ENSMUSG00000027823	Gmps	2354.476	0.309	0.099	3.127	1.8E-03	2.9E-02
ENSMUSG00000053279	Aldh1a1	681.217	-0.501	0.160	-3.126	1.8E-03	3.0E-02
ENSMUSG00000022419	Deptor	760.277	-0.459	0.147	-3.122	1.8E-03	3.0E-02
ENSMUSG00000041670	Rims1	3433.027	-0.417	0.134	-3.120	1.8E-03	3.0E-02
ENSMUSG00000039824	Myl6b	212.823	0.716	0.229	3.120	1.8E-03	3.0E-02
ENSMUSG00000027893	Ahcyl1	8112.722	-0.282	0.090	-3.118	1.8E-03	3.0E-02
ENSMUSG00000048285	Frmd6	411.073	-0.562	0.180	-3.117	1.8E-03	3.0E-02
ENSMUSG00000063434	Sorcs3	686.344	-0.548	0.176	-3.116	1.8E-03	3.0E-02
ENSMUSG00000022426	Josd1	1135.222	-0.376	0.121	-3.114	1.8E-03	3.1E-02
ENSMUSG00000042121	Ssh1	523.382	0.503	0.162	3.114	1.8E-03	3.1E-02
ENSMUSG00000045962	Wnk1	8574.427	0.298	0.096	3.113	1.8E-03	3.1E-02

ENSMUSG00000042680	Garem1	712.235	-0.452	0.145	-3.112	1.9E-03	3.1E-02
ENSMUSG00000042109	Csdc2	868.948	-0.475	0.153	-3.106	1.9E-03	3.1E-02
ENSMUSG00000028541	B4galt2	757.291	-0.420	0.135	-3.105	1.9E-03	3.1E-02
ENSMUSG00000021194	Chga	1418.405	-0.388	0.125	-3.105	1.9E-03	3.1E-02
ENSMUSG00000048826	Dact2	449.224	-0.510	0.164	-3.104	1.9E-03	3.1E-02
ENSMUSG00000058070	Eml1	1356.176	-0.381	0.123	-3.104	1.9E-03	3.1E-02
ENSMUSG00000041329	Atp1b2	6660.211	-0.300	0.097	-3.103	1.9E-03	3.1E-02
ENSMUSG00000048827	Pkd1l3	20.927	2.420	0.781	3.100	1.9E-03	3.2E-02
ENSMUSG00000034926	Dhcr24	987.800	-0.458	0.148	-3.098	1.9E-03	3.2E-02
ENSMUSG00000022641	Bbx	1395.213	0.371	0.120	3.093	2.0E-03	3.2E-02
ENSMUSG00000024050	Wiz	769.981	0.441	0.143	3.091	2.0E-03	3.2E-02
ENSMUSG00000021662	Arhgef28	469.270	0.514	0.166	3.091	2.0E-03	3.3E-02
ENSMUSG00000034007	Scaper	1141.799	0.377	0.122	3.089	2.0E-03	3.3E-02
ENSMUSG00000066129	Kndc1	1482.089	0.373	0.121	3.089	2.0E-03	3.3E-02
ENSMUSG00000024498	Tcerg1	1972.087	0.317	0.103	3.088	2.0E-03	3.3E-02
ENSMUSG00000003948	Mmd	4253.361	-0.285	0.092	-3.086	2.0E-03	3.3E-02
ENSMUSG00000061887	Ssbp3	1790.697	-0.411	0.133	-3.083	2.0E-03	3.3E-02
ENSMUSG00000030259	Rassf8	551.651	0.467	0.152	3.081	2.1E-03	3.3E-02
ENSMUSG00000042807	Hecw2	1428.448	-0.425	0.138	-3.077	2.1E-03	3.4E-02
ENSMUSG00000096764	Gm21985	202.563	0.733	0.238	3.077	2.1E-03	3.4E-02
ENSMUSG00000032261	Sh3bgrl2	765.105	-0.433	0.141	-3.074	2.1E-03	3.4E-02
ENSMUSG00000067629	Syngap1	4079.745	-0.275	0.089	-3.074	2.1E-03	3.4E-02
ENSMUSG00000050052	Tdrp	483.543	-0.498	0.162	-3.069	2.1E-03	3.5E-02
ENSMUSG00000031748	Gnao1	15765.601	-0.240	0.078	-3.064	2.2E-03	3.5E-02
ENSMUSG00000030898	Cckbr	200.965	-0.818	0.267	-3.064	2.2E-03	3.5E-02
ENSMUSG00000046321	Hs3st2	248.087	-0.869	0.284	-3.064	2.2E-03	3.5E-02
ENSMUSG00000078899	Gm4631	3553.141	0.284	0.093	3.062	2.2E-03	3.5E-02
ENSMUSG00000023036	Pcdhgc4	1350.710	-0.474	0.155	-3.060	2.2E-03	3.5E-02
ENSMUSG00000028096	Gpr89	790.038	0.421	0.138	3.056	2.2E-03	3.6E-02
ENSMUSG00000022672	Prkdc	1237.455	0.390	0.128	3.057	2.2E-03	3.6E-02
ENSMUSG00000031246	Sh3bgrl	2466.683	0.316	0.104	3.053	2.3E-03	3.6E-02
ENSMUSG00000028312	Smc2	374.236	0.619	0.203	3.044	2.3E-03	3.7E-02
ENSMUSG00000046844	Vat1l	2128.971	-0.304	0.100	-3.043	2.3E-03	3.7E-02
ENSMUSG00000044365	Cxxc4	1064.808	0.381	0.125	3.040	2.4E-03	3.8E-02
ENSMUSG00000022377	Asap1	1682.296	-0.425	0.140	-3.038	2.4E-03	3.8E-02
ENSMUSG00000040276	Pacsin1	4625.974	-0.286	0.094	-3.035	2.4E-03	3.8E-02
ENSMUSG00000032024	Clmp	740.170	-0.418	0.138	-3.035	2.4E-03	3.8E-02
ENSMUSG00000044813	Shb	247.020	-0.684	0.225	-3.035	2.4E-03	3.8E-02
ENSMUSG00000022353	Mtss1	782.857	0.431	0.142	3.032	2.4E-03	3.8E-02
ENSMUSG00000026411	Tmem9	459.411	0.491	0.162	3.029	2.5E-03	3.9E-02
ENSMUSG00000043671	Dpy19l3	1318.801	-0.425	0.141	-3.028	2.5E-03	3.9E-02
ENSMUSG00000066026	Dhrs3	155.091	-0.797	0.263	-3.028	2.5E-03	3.9E-02
ENSMUSG00000052397	Ezr	1058.298	-0.395	0.131	-3.026	2.5E-03	3.9E-02
ENSMUSG00000047146	Tet1	558.703	0.528	0.175	3.026	2.5E-03	3.9E-02
ENSMUSG00000022389	Tef	5479.922	-0.258	0.085	-3.024	2.5E-03	3.9E-02
ENSMUSG00000038112	AW551984	463.089	1.271	0.421	3.023	2.5E-03	3.9E-02
ENSMUSG00000003585	Sec14l2	453.408	-0.538	0.178	-3.021	2.5E-03	3.9E-02
ENSMUSG00000046999	1110032F04Rik	77.390	-1.156	0.383	-3.020	2.5E-03	3.9E-02
ENSMUSG00000040140	Tdrd6	45.209	1.487	0.493	3.018	2.5E-03	4.0E-02
ENSMUSG00000032737	Inppl1	458.619	-0.502	0.166	-3.018	2.5E-03	4.0E-02
ENSMUSG00000029126	Nsg1	4096.642	-0.268	0.089	-3.017	2.6E-03	4.0E-02
ENSMUSG00000039195	Bbln	314.749	-0.667	0.221	-3.014	2.6E-03	4.0E-02
ENSMUSG00000023236	Scg5	2994.622	0.336	0.111	3.013	2.6E-03	4.0E-02
ENSMUSG00000004347	Pde1c	739.026	-0.416	0.138	-3.011	2.6E-03	4.0E-02
ENSMUSG00000041817	Fam169a	1580.365	0.323	0.107	3.009	2.6E-03	4.0E-02
ENSMUSG00000020733	Slc9a3r1	518.026	-0.475	0.158	-3.010	2.6E-03	4.0E-02

ENSMUSG00000029408	Abcb9	541.661	-0.453	0.150	-3.009	2.6E-03	4.0E-02
ENSMUSG00000022114	Spry2	739.124	-0.416	0.138	-3.008	2.6E-03	4.1E-02
ENSMUSG00000090626	Tex9	453.285	0.617	0.205	3.006	2.6E-03	4.1E-02
ENSMUSG00000004558	Ndrg2	9913.025	-0.295	0.098	-3.006	2.6E-03	4.1E-02
ENSMUSG00000029014	Dnajc2	832.923	-0.408	0.136	-3.004	2.7E-03	4.1E-02
ENSMUSG00000037857	Nufip2	1234.074	0.364	0.121	3.003	2.7E-03	4.1E-02
ENSMUSG00000004846	Plod3	386.341	0.577	0.192	3.001	2.7E-03	4.1E-02
ENSMUSG00000027276	Jag1	200.729	-0.706	0.236	-2.995	2.7E-03	4.2E-02
ENSMUSG00000018481	Appbp2	2623.554	0.288	0.096	2.986	2.8E-03	4.3E-02
ENSMUSG00000033054	Npat	1009.052	0.366	0.123	2.986	2.8E-03	4.3E-02
ENSMUSG00000036977	Anapc10	222.663	0.670	0.225	2.985	2.8E-03	4.3E-02
ENSMUSG00000074340	Ovgp1	60.222	-1.316	0.441	-2.981	2.9E-03	4.4E-02
ENSMUSG00000027660	Skil	2322.275	-0.292	0.098	-2.981	2.9E-03	4.4E-02
ENSMUSG00000044534	Ackr2	36.149	-1.726	0.579	-2.981	2.9E-03	4.4E-02
ENSMUSG00000045098	Kmt5b	1504.250	0.326	0.109	2.979	2.9E-03	4.4E-02
ENSMUSG00000023089	Ndufa5	675.561	-0.432	0.145	-2.978	2.9E-03	4.4E-02
ENSMUSG00000035357	Pdzrn3	247.424	-0.693	0.233	-2.976	2.9E-03	4.4E-02
ENSMUSG00000086477	Gm15506	65.574	1.209	0.406	2.974	2.9E-03	4.5E-02
ENSMUSG00000006611	Hfe	79.092	1.045	0.351	2.973	2.9E-03	4.5E-02
ENSMUSG00000035620	Ric8b	2169.290	-0.326	0.110	-2.971	3.0E-03	4.5E-02
ENSMUSG00000054252	Fgfr3	1211.965	-0.359	0.121	-2.971	3.0E-03	4.5E-02
ENSMUSG00000030835	Nomo1	2102.643	-0.298	0.100	-2.970	3.0E-03	4.5E-02
ENSMUSG00000028871	Rspo1	44.490	-1.597	0.538	-2.970	3.0E-03	4.5E-02
ENSMUSG00000079434	Neu2	77.095	-1.087	0.366	-2.967	3.0E-03	4.5E-02
ENSMUSG00000035863	Palm	2744.431	-0.297	0.100	-2.966	3.0E-03	4.5E-02
ENSMUSG00000037907	Ankrd13b	908.247	-0.397	0.134	-2.965	3.0E-03	4.6E-02
ENSMUSG00000061086	Myl4	38.742	-1.698	0.573	-2.963	3.0E-03	4.6E-02
ENSMUSG00000030231	Plekha5	1897.010	-0.371	0.125	-2.961	3.1E-03	4.6E-02
ENSMUSG00000055675	Kbtbd11	2479.249	-0.300	0.101	-2.959	3.1E-03	4.6E-02
ENSMUSG00000029219	Slc10a4	144.179	-0.804	0.272	-2.958	3.1E-03	4.6E-02
ENSMUSG00000024059	Clip4	1767.068	-0.321	0.108	-2.956	3.1E-03	4.7E-02
ENSMUSG00000042156	Dzip1	1729.411	0.309	0.104	2.956	3.1E-03	4.7E-02
ENSMUSG00000025579	Gaa	4480.353	0.273	0.092	2.955	3.1E-03	4.7E-02
ENSMUSG00000037306	Man1c1	438.027	-0.500	0.169	-2.953	3.1E-03	4.7E-02
ENSMUSG00000024241	Sos1	1667.776	0.338	0.115	2.951	3.2E-03	4.7E-02
ENSMUSG00000034098	Fstl5	853.854	0.377	0.128	2.950	3.2E-03	4.7E-02
ENSMUSG00000038056	Kmt2c	2957.466	0.295	0.100	2.948	3.2E-03	4.8E-02
ENSMUSG00000022141	Nipbl	2011.207	0.312	0.106	2.948	3.2E-03	4.8E-02
ENSMUSG00000040624	Plekhg1	596.007	0.467	0.159	2.944	3.2E-03	4.8E-02
ENSMUSG00000051285	Pcmtd1	4537.439	-0.296	0.100	-2.944	3.2E-03	4.8E-02
ENSMUSG00000052837	Junb	374.608	-0.576	0.196	-2.941	3.3E-03	4.8E-02
ENSMUSG00000024245	Tmem178	289.685	-0.762	0.259	-2.941	3.3E-03	4.8E-02
ENSMUSG00000010721	Lmbr1	767.169	0.390	0.133	2.941	3.3E-03	4.8E-02
ENSMUSG00000006731	B4galnt1	1118.480	-0.360	0.122	-2.942	3.3E-03	4.8E-02
ENSMUSG00000055322	Tns1	1091.874	-0.351	0.119	-2.939	3.3E-03	4.8E-02
ENSMUSG00000036306	Lzts1	1640.298	-0.340	0.116	-2.936	3.3E-03	4.9E-02
ENSMUSG00000038042	Ptpdc1	701.232	0.404	0.138	2.936	3.3E-03	4.9E-02

Supplementary Table S3. Differential splicing of the retina and striatum of 13-15-month-old R6/1 and wt littermates

Genome of reference for *Genomic location* : Mus_musculus.GRCm39.106 (rMATS, SUPPA2) and GRCm38/mm10 v88 (vast-tools)

		RETINA		
Gene	*Event*	*Genomic location*	*Type of event*	*Software*
0610040B10Rik	SE	143316458-143316640-143315081-143315108-143318065-143318456	SE	rMATS
2810403D21Rik	SE	107915077-107915204-107879834-107879961-107945069-107945161	SE	rMATS
4930447C04Rik	SE	72941757-72941819-72939577-72939753-72945260-72945307	SE	rMATS
9430041J12Rik	SE	4122678-4122807-4113189-4113291-4122909-4123727	SE	rMATS
A430035B10Rik	SE	8497407-8497560-8492421-8492804-8508978-8509021	SE	rMATS
Abcc10	RI	46615082-46615401-46615082-46615180-46615255-46615401	RI	rMATS
Ablim1	SE	57036798-57036865-57035356-57035503-57037893-57037941	SE	rMATS
Ablim1	SE	57065845-57065929-57062286-57062334-57068244-57068303	SE	rMATS
Acap3	RI	155987767-155988315-155987767-155987970-155988207-155988315	RI	rMATS
Adck5	RI	76478137-76478647-76478137-76478251-76478352-76478647	RI	rMATS
Add3	SE	53191844-53191906-53135146-53135186-53205231-53205446	SE	rMATS
Ankrd24	SE	81477809-81477882-81476725-81476824-81478134-81478253	SE	rMATS
Atad3aos	SE	155845841-155846234-155845677-155845743-155847826-155848017	SE	rMATS
Atxn7l1	SE	33414383-33414689-33412971-33413093-33416970-33417913	SE	rMATS
Bend4	A5SS	67557403-67557644-67557474-67557644-67549490-67555793	A5	rMATS
Bend4	RI	67549490-67557644-67549490-67555793-67557403-67557644	RI	rMATS
Bend4	RI	67549490-67557644-67549490-67555793-67557474-67557644	RI	rMATS
C330018D20Rik	SE	57091594-57091651-57088904-57090993-57106455-57106556	SE	rMATS
Cacna1g	SE	94314496-94314550-94309644-94309796-94316589-94316803	SE	rMATS
Cacna1g	SE	94305756-94305900-94302264-94302442-94306568-94306925	SE	rMATS
Cenpl	RI	160910477-160913908-160910477-160911020-160913557-160913908	RI	rMATS
Clgn	SE	84134782-84134864-84126577-84126719-84136114-84136307	SE	rMATS
Col20a1	MXE	180641825-180641968-180642157-180642290-180641479-180641609-1	Exc	rMATS
Coq4	A3SS	29685841-29686041-29685947-29686041-29685392-29685522	A3	rMATS
Crem	SE	3287903-3288092-3273421-3273578-3295039-3295182	SE	rMATS
Cstf2	A3SS	132973884-132974026-132973962-132974026-132973163-132973223	A3	rMATS
Diaph1	SE	38039440-38039467-38038056-38038212-38068254-38068434	SE	rMATS
Dlg4	SE	69908611-69908652-69908066-69908202-69921224-69921278	SE	rMATS
Drp2	RI	133341083-133342135-133341083-133341169-133341977-133342135	RI	rMATS
Eef1d	SE	75777734-75777749-75772980-75773104-75781093-75781184	SE	rMATS
Eif4g3	SE	137811567-137811681-137810203-137810299-137823219-137823240	SE	rMATS
Exoc7	SE	116187555-116187624-116186507-116186515-116191091-116191259	SE	rMATS
Fbxo17	A3SS	28436763-28437569-28437089-28437569-28435492-28435628	A3	rMATS
Fbxo17	SE	28436763-28436971-28435492-28435628-28437089-28437563	SE	rMATS
Gm32031	A3SS	46658410-46658756-46658471-46658756-46657830-46658194	A3	rMATS
Gnat2	RI	108002754-108003823-108002754-108002978-108003665-108003823	RI	rMATS
Gpatch8	SE	102422138-102422156-102418919-102418992-102429107-102429182	SE	rMATS
Gpld1	SE	25170874-25170901-25168690-25168851-25174018-25176484	SE	rMATS
Heatr5a	SE	51958987-51959014-51956740-51956876-51961756-51961926	SE	rMATS
Hectd2	A3SS	36578828-36578928-36578831-36578928-36576995-36577119	A3	rMATS
Hfm1	SE	107066242-107066355-107065189-107065493-107070678-107070776	SE	rMATS
Hsd3b7	RI	127400246-127400788-127400246-127400418-127400632-127400788	RI	rMATS
Kcnip2	MXE	45782918-45783026-45783178-45783249-45782637-45782742-45783416	Exc	rMATS
Kdm2b	SE	123019699-123019813-123019082-123019121-123020026-123020287	SE	rMATS
Kif1b	SE	149279929-149279956-149277029-149277144-149282888-149282951	SE	rMATS
Ktn1	SE	47963385-47963457-47962334-47962427-47963682-47963766	SE	rMATS
L3mbtl3	SE	26220074-26220149-26218405-26218565-26220509-26220621	SE	rMATS
Lrp8	SE	107705477-107705600-107704637-107704763-107708504-107708559	SE	rMATS
Lrwd1	RI	136162158-136162904-136162158-136162299-136162787-136162904	RI	rMATS
Man2a2	SE	80005735-80005810-80002909-80003110-80006089-80006226	SE	rMATS
Marchf2	SE	33934916-33935036-33928718-33928946-33937519-33937565	SE	rMATS
Mars1	SE	127132585-127132609-127132409-127132502-127132741-127132813	SE	rMATS
Midn	SE	79989424-79989553-79987494-79987557-79989649-79989702	SE	rMATS
Mtfmt	A5SS	65348894-65349081-65348894-65348970-65351160-65351252	A5	rMATS
Mvk	SE	114582712-114582750-114582329-114582424-114583448-114583540	SE	rMATS
Necap2	A3SS	140798891-140799028-140798891-140799006-140799839-140799948	A3	rMATS
Nfrkb	A3SS	31308089-31308433-31308295-31308433-31307618-31307793	A3	rMATS
Nin	SE	70157845-70158256-70149424-70149628-70158627-70158809	SE	rMATS
Nr1h4	A5SS	89319233-89319386-89319245-89319386-89316352-89316489	A5	rMATS
Oprl1	SE	181359747-181359886-181357482-181357739-181360169-181360525	SE	rMATS
Orai2	SE	136197219-136197328-136190340-136190578-136199391-136199482	SE	rMATS
Pan3	SE	147439894-147440012-147424920-147425068-147458639-147458744	SE	rMATS
Paqr6	RI	88272334-88273109-88272334-88272410-88272981-88273109	RI	rMATS
Pex5	SE	124381091-124381202-124380770-124380866-124381588-124381679	SE	rMATS
Pitpnm3	SE	71953920-71954069-71949663-71949780-71956291-71956462	SE	rMATS
Plekhg3	A5SS	76619168-76619519-76619168-76619432-76620020-76620134	A5	rMATS
Pou2f1	SE	165780349-165780425-165773470-165773571-165830130-165830178	SE	rMATS
Pts	SE	50436452-50436584-50436022-50436079-50438169-50438249	SE	rMATS

Rab28	SE	41784329-41784424-41782318-41783205-41790515-4179059	SE	rMATS
Rapgef6	SE	54452150-54452220-54443615-54443699-54459178-5445932	SE	rMATS
Rbm25	A5SS	83685901-83686149-83685901-83685965-83689175-8368929	A5	rMATS
Rcor3	SE	191792943-191793001-191785687-191786167-191800568-19180064	SE	rMATS
Rims1	SE	22482434-22482518-22474667-22474739-22491964-2249205	SE	rMATS
Rnpepl1	RI	92844868-92845468-92844868-92844982-92845355-9284546	RI	rMATS
Rp1	MXE	4267756-4267864-4276882-4277060-4240427-4240627-4296833-429704	Exc	rMATS
Rpusd3	MXE	113393267-113393407-113393749-113393873-113392279-113392683-1	Exc	rMATS
Sema6a	SE	47403675-47403840-47378320-47382651-47409652-4740967	SE	rMATS
Sez6l	A3SS	112573479-112573594-112573479-112573591-112574591-11257462	A3	rMATS
Slc17a7	SE	44818934-44819009-44818138-44818391-44819240-4481935	SE	rMATS
Slc24a2	SE	87094398-87094584-86990678-86991533-87145119-8714620	SE	rMATS
Slc39a11	A5SS	113354776-113354921-113354797-113354921-113260553-11326072	A5	rMATS
Sp100	MXE	85608806-85608860-85619695-85619770-85606779-85606830-8562197	Exc	rMATS
Ssh3	A3SS	4318572-4318613-4318572-4318610-4319042-431920	A3	rMATS
Stk19	A5SS	35050973-35051128-35050988-35051128-35043726-3504382	A5	rMATS
Syt7	SE	10400148-10400376-10399120-10399252-10403662-1040403	SE	rMATS
Tcf3	SE	80246231-80246458-80245347-80246108-80248660-8024889	SE	rMATS
Tmem234	SE	129495199-129495326-129494775-129495036-129495662-12949575	SE	rMATS
Tpm1	SE	66936945-66937024-66935173-66935449-66938310-6693838	SE	rMATS
Ttc13	SE	125426394-125426460-125422399-125422491-125436641-12543668	SE	rMATS
Wasf3	SE	146402659-146402829-146392418-146392471-146403607-14640387	SE	rMATS
Zbtb20	SE	43397392-43397556-43392096-43392154-43397923-4339811	SE	rMATS
Zfp532	SE	65777263-65777440-65758208-65758405-65815965-6581607	SE	rMATS
Zfp532	SE	65789421-65789703-65756054-65758405-65815965-6581607	SE	rMATS
Zfp810	SE	22191748-22191855-22188043-22190576-22194463-2219455	SE	rMATS
1810009A15Rik	A5	19:8867091-8867367:8866612-8867367:+	A5	SUPPA2
Ache	A5	5:137286319-137288271:137286290-137288271:+	A5	SUPPA2
Agrp	AF	8:106294358-106294848:106294930:106294358-106306282:106306477:	Exc	SUPPA2
Aldoc	SE	11:78216642-78216761:78216960-78217088:+	SE	SUPPA2
Apoe	A3	7:19431572-19432113:19431539-19432113:-	A3	SUPPA2
Arfgap2	SE	2:91098853-91099415:91099456-91099797:+	SE	SUPPA2
Asxl1	SE	2:153194191-153196570:153196572-153198990:+	SE	SUPPA2
Atp1b2	RI	11:69492351:69492449-69493251:69493307:-	RI	SUPPA2
Atp5g3	A5	2:73741404-73741586:73741404-73741596:-	A5	SUPPA2
BC028528	A3	3:95792383-95795453:95792380-95795453:-	A3	SUPPA2
Bex1	SE	X:135115332-135115653:135115729-135116086:-	SE	SUPPA2
Bin1	SE	18:32547343-32547998:32548090-32552896:+	SE	SUPPA2
Bsg	A3	10:79546236-79546459:79546236-79546472:+	A3	SUPPA2
Bsg	RI	10:79546472:79546558-79546674:79546754:+	RI	SUPPA2
Bsg	SE	10:79540418-79544531:79544878-79545505:+	SE	SUPPA2
Bud31	A5	5:145078246-145079258:145078170-145079258:+	A5	SUPPA2
Bud31	SE	5:145079377-145081709:145081831-145083253:+	SE	SUPPA2
Calm2	SE	17:87743362-87750124:87750154-87754221:-	SE	SUPPA2
Camta2	SE	11:70561562-70561717:70561737-70561834:-	SE	SUPPA2
Camta2	SE	11:70573873-70574105:70574176-70574716:-	SE	SUPPA2
Camta2	SE	11:70576992-70577173:70577267-70578712:-	SE	SUPPA2
Ccdc66	SE	14:27204559-27204898:27205147-27205765:-	SE	SUPPA2
Cd302	SE	2:60082835-60085415:60085441-60087377:-	SE	SUPPA2
Cd34	A3	1:194641538-194642088:194641538-194642244:+	A3	SUPPA2
Cdc37	A3	9:21053973-21054243:21053826-21054243:-	A3	SUPPA2
Celf2	RI	2:6544516:6551590-6551853:6552024:-	RI	SUPPA2
Ciart	AL	3:95785815:95786461-95787721:95786822:95787097-95787721:	Exc	SUPPA2
Cox4i1	A3	8:121396121-121399464:121396121-121399468:+	A3	SUPPA2
Cox4i1	A5	8:121395094-121396048:121395090-121396048:+	A5	SUPPA2
Cox8a	SE	19:7192849-7193846:7193914-7194764:-	SE	SUPPA2
Cplx3	AF	9:57512087-57512268:57512361:57512087-57513264:57513564:	Exc	SUPPA2
Cplx3	SE	9:57509744-57512000:57512087-57513264:-	SE	SUPPA2
Cpne8	AF	15:90533486-90533578:90533641:90533486-90563371:90563591:	Exc	SUPPA2
Crbn	A3	6:106772958-106776952:106772955-106776952:-	A3	SUPPA2
Cstpp1	AL	2:91105413:91107479-91109535:91108175:91109302-91109535:	Exc	SUPPA2
Ctsl	A3	13:64514906-64515674:64514738-64515674:-	A3	SUPPA2
D030056L22Rik	A3	19:18691023-18694583:18691023-18694586:+	A3	SUPPA2
Ddx49	A3	8:70747485-70748017:70747411-70748017:-	A3	SUPPA2
Dennd10	A3	19:60800111-60803030:60800111-60803050:+	A3	SUPPA2
Dennd10	SE	19:60803246-60805969:60806048-60809645:+	SE	SUPPA2
Dync2i1	SE	12:116221094-116226159:116226308-116226471:-	SE	SUPPA2
Emp3	AF	7:45569828-45570551:45570586:45569828-45570666:45570828:	Exc	SUPPA2
Fabp12	SE	3:10317417-10326470:10326682-10366185:-	SE	SUPPA2
Fam13c	A3	10:70387620-70388859:70387620-70388862:+	A3	SUPPA2
Gal3st4	A3	5:138269856-138270994:138269642-138270994:-	A3	SUPPA2
Gal3st4	SE	5:138264568-138269030:138269333-138269541:-	SE	SUPPA2
Gm13547	AF	2:29649324:29649537-29653407:29651540:29651818-29653407:-	Exc	SUPPA2

Gpr39	AF	1:125604732:125605929-125800107:125767740:125768063-125800107:	Exc	SUPPA2
Grin3a	A3	4:49665625-49670024:49665565-49670024:-	A3	SUPPA2
Hcls1	SE	16:36769026-36771495:36771549-36775622:+	SE	SUPPA2
Hnrnpm	SE	17:33888150-33889055:33889107-33896248:-	SE	SUPPA2
Hp1bp3	A3	4:137949499-137950801:137949499-137950840:+	A3	SUPPA2
Hp1bp3	AF	4:137943945:137944051-137949400:137944438:137944605-137949400:	Exc	SUPPA2
Hp1bp3	SE	4:137944051-137948837:137949034-137949400:+	SE	SUPPA2
Hspa8	A3	9:40713919-40714035:40713919-40714092:+	A3	SUPPA2
Kif3a	SE	11:53477757-53481544:53481552-53484201:+	SE	SUPPA2
Mier3	SE	13:111840297-111840949:111840985-111841756:+	SE	SUPPA2
Myo6	SE	9:80199799-80200809:80200835-80203925:+	SE	SUPPA2
Naca	SE	10:127872429-127875040:127880955-127882037:+	SE	SUPPA2
Ncor1	AF	11:62329341-62347974:62348158:62329341-62349281:62349367:	Exc	SUPPA2
Nfia	AF	4:97660971:97661148-97671319:97665845:97666231-97671319:-	Exc	SUPPA2
Nfia	AF	4:97660971:97661148-97671319:97665863:97666231-97671319:-	Exc	SUPPA2
Nfia	AF	4:97660971:97661148-97671319:97666056:97666231-97671319:-	Exc	SUPPA2
Nfib	A5	4:82238793-82241782:82238793-82241809:-	A5	SUPPA2
Nfib	SE	4:82215048-82228540:82228628-82238711:-	SE	SUPPA2
Ntan1	RI	16:13644747:13644849-13644924:13644989:+	RI	SUPPA2
Ntan1	SE	16:13637300-13644747:13644849-13644924:+	SE	SUPPA2
Paf1	RI	7:28094843:28094964-28095054:28095120:+	RI	SUPPA2
Pcbp3	A5	10:76617707-76620905:76617707-76620974:-	A5	SUPPA2
Pdss1	SE	2:22825651-22829091:22829218-22829835:+	SE	SUPPA2
Pgk1	A3	X:105243367-105243723:105243367-105243763:+	A3	SUPPA2
Phf23	A3	11:69888664-69889127:69888664-69889328:+	A3	SUPPA2
Pitpnm3	SE	11:71965861-71983354:71983401-72003083:-	SE	SUPPA2
Pllp	AF	8:95406146-95406623:95406710:95406146-95422664:95422906:	Exc	SUPPA2
Ppp1r1a	SE	15:103441921-103442777:103442837-103446222:-	SE	SUPPA2
Pram1	A5	17:33857186-33859438:33857151-33859438:+	A5	SUPPA2
Prkd3	A3	17:79270008-79273604:79270005-79273604:-	A3	SUPPA2
Ptma	A3	1:86457526-86457687:86457526-86457726:+	A3	SUPPA2
Ptpmt1	SE	2:90744519-90747198:90747255-90747773:-	SE	SUPPA2
Rbks	AF	5:31830789-31843313:31843437:31830789-31854854:31854971:	Exc	SUPPA2
Rbl2	A3	8:91822286-91823392:91822286-91823422:+	A3	SUPPA2
Rbpms	SE	8:34274214-34279420:34279523-34285110:-	SE	SUPPA2
Rbpms	SE	8:34285178-34296745:34296878-34324332:-	SE	SUPPA2
Rpl11	A3	4:135779031-135779878:135779027-135779878:-	A3	SUPPA2
Rpl11	AF	4:135780028-135780281:135780401:135780028-135780659:135780739:	Exc	SUPPA2
Rpl19	AF	11:97917536:97917588-97918649:97917750:97917816-97918649:-	Exc	SUPPA2
Rpl22	A3	4:152414585-152416717:152414585-152416737:+	A3	SUPPA2
Rpl23a	RI	11:78071761:78071823-78071980:78072049:-	RI	SUPPA2
Rpl29	A5	9:106306838-106307169:106306775-106307169:+	A5	SUPPA2
Rpl29	A5	9:106307012-106307169:106306775-106307169:+	A5	SUPPA2
Rplp0	A3	5:115697947-115698778:115697947-115698800:+	A3	SUPPA2
Rps27a	RI	11:29496703:29496788-29497206:29497260:-	RI	SUPPA2
Rtn3	SE	19:7412503-7445176:7445232-7460304:-	SE	SUPPA2
Rwdd2a	A5	9:86454270-86454890:86454141-86454890:+	A5	SUPPA2
Scn1a	AF	2:66181570-66240206:66240408:66181570-66271044:66271158:	Exc	SUPPA2
Scn1a	AF	2:66181570-66240206:66240408:66181570-66271044:66271181:	Exc	SUPPA2
Sfpq	A3	4:126917361-126917443:126917361-126917467:+	A3	SUPPA2
Sh2b3	RI	5:121956528:121956742-121956823:121956917:-	RI	SUPPA2
Sh2b3	SE	5:121967139-121970566:121970653-121974613:-	SE	SUPPA2
Skida1	AF	2:18053263-18053498:18053595:18053263-18053759:18053842:	Exc	SUPPA2
Slc22a29	A3	19:8139069-8140013:8138995-8140013:-	A3	SUPPA2
Snip1	A3	4:124960745-124961800:124960745-124961883:+	A3	SUPPA2
Socs6	SE	18:88889042-88905116:88905189-88905333:-	SE	SUPPA2
Spata45	AF	1:190768836:190768909-190771742:190769016:190769071-190771742:	Exc	SUPPA2
Spcs1	A3	14:30722721-30722801:30722717-30722801:-	A3	SUPPA2
Spink8	SE	9:109649764-109653577:109653619-109655440:+	SE	SUPPA2
Sptbn1	AF	11:30109445-30147724:30148257:30109445-30169579:30169772:	Exc	SUPPA2
Srsf3	SE	17:29257598-29258428:29258883-29259749:+	SE	SUPPA2
Tasor	A3	14:27202048-27202599:27202048-27202873:+	A3	SUPPA2
Tektip1	A3	10:81200070-81200376:81199996-81200376:-	A3	SUPPA2
Tektip1	A3	10:81200099-81200376:81200070-81200376:-	A3	SUPPA2
Tet2	AF	3:133219934-133249961:133250151:133219934-133250806:133250900:	Exc	SUPPA2
Tmsb4x	A5	X:165991047-165992004:165991047-165992102:-	A5	SUPPA2
Tpi1	RI	6:124789584:124789668-124789753:124789876:-	RI	SUPPA2
Tpm4	AF	8:72889073:72889297-72892432:72889652:72889764-72892432:-	Exc	SUPPA2
Trio	AF	15:27919377-28024884:28025040:27919377-28025426:28025934:	Exc	SUPPA2
Tsr2	A5	X:149873410-149873592:149873410-149873601:-	A5	SUPPA2
Ttc3	AF	16:94171609:94171720-94184771:94181600:94182312-94184771:-	Exc	SUPPA2
Ttc3	AF	16:94171657:94171720-94184771:94181600:94182312-94184771:-	Exc	SUPPA2
Ubb	AF	11:62441997:62442421-62442966:62442502:62442677-62442966:-	Exc	SUPPA2

Ube2i	AF	17:25488453-25492295:25492508:25488453-25493147:25493284:	Exc	SUPPA2
Ube2j1	SE	4:33043986-33045080:33045199-33049682:+	SE	SUPPA2
Uqcr10	AF	11:4652220-4653904:4654053:4652220-4654157:4654342:-	Exc	SUPPA2
Vasn	AL	16:4458012-4465246:4465423:4458012-4466046:4468666:+	Exc	SUPPA2
Wdr48	A5	9:119745899-119746177:119745857-119746177:+	A5	SUPPA2
Xpa	A5	4:46183235-46184281:46183235-46184288:-	A5	SUPPA2
Xpa	AL	4:46155347:46155826-46183118:46175222:46175415-46183118:	Exc	SUPPA2
Xpa	SE	4:46175415-46180207:46180398-46183118:-	SE	SUPPA2
Ybx1	A3	4:119136408-119138767:119136396-119138767:-	A3	SUPPA2
Ybx1	SE	4:119139645-119139978:119140067-119142306:-	SE	SUPPA2
Ywhaq	SE	12:21441416-21441718:21441800-21444966:-	SE	SUPPA2
Zc3h13	A3	14:75522105-75529009:75522105-75529012:+	A3	SUPPA2
Zc3h13	A3	14:75569658-75573362:75569658-75573365:+	A3	SUPPA2
Zcchc7	SE	4:44929235-44929581:44929645-44931036:+	SE	SUPPA2
Zdhhc7	AF	8:120819740-120827593:120827811:120819740-120827831:120828221:	Exc	SUPPA2
Zfp967	A3	2:176221175-176225042:176221175-176225048:+	A3	SUPPA2
0610009B22Rik	MmuALTD1002719-3/4	chr11:51688548+51688543+51688360+51688207-51688653,51686092	A5	vast-tools
1110020A21Rik	MmuINT1000050	chr17:84957200-84957710=84954967-84955308:-	RI	vast-tools
1300002E11Rik	MmuEX0000218	chr16:21795975,21800715+21800775-21800836,21808247+21808678	SE	vast-tools
2700097O09Rik	MmuEX0001212	chr12:55154822,55053301-55053407,55048977+55048994	SE	vast-tools
4833420G17Rik	MmuINT1001291	chr13:119462759-119463119=119466011-119466304:+	RI	vast-tools
4833420G17Rik	MmuEX0001376	chr13:119463119,119465280-119465387,119466011+119466019+119466	SE	vast-tools
A830010M20Rik	MmuINT1003217	chr5:107496736-107496873=107497721-107497850:+	RI	vast-tools
A930003O13Rik	MmuINT1003258	chr5:22745585-22746887=22742988-22743207:-	RI	vast-tools
A930003O13Rik	MmuINT1003259	chr5:22742988-22743207=22738910-22739341:-	RI	vast-tools
Abhd14a	MmuINT0008920	chr9:106447531-106447563=106445526-106445834:-	RI	vast-tools
Abhd14a	MmuINT1003529	chr9:106445526-106445834=106444090-106444306:-	RI	vast-tools
Abi1	MmuEX0003244	chr2:22953435,22950184-22950270,22946761	SE	vast-tools
Abl2	MmuALTA0000951-1/2	chr1:156640612,156640996+156640993-156641229	A3	vast-tools
Ablim1	MmuEX0003298	chr19:57061253,57051130-57051234,57049509	SE	vast-tools
Ablim1	MmuEX0003302	chr19:57079813,57077414-57077497,57068937	SE	vast-tools
Acad9	MmuINT0009406	chr3:36089332-36089460=36090082-36090154:+	RI	vast-tools
Acrbp	MmuINT1003708	chr6:125053490-125053952=125054673-125054805:+	RI	vast-tools
Actr1b	MmuINT0010514	chr1:36701685-36701901=36701421-36701513:-	RI	vast-tools
Adamts3	MmuEX0003862	chr5:89698204,89696003-89696086,89693202	SE	vast-tools
Adamts3	MmuEX0003863	chr5:89698204,89694750-89694830,89693202	SE	vast-tools
Adcy6	MmuINT0011881	chr15:98596517-98596675=98596040-98596218:-	RI	vast-tools
Adgrl3	MmuEX0026728	chr5:81310079,81329757-81329960,81387775	SE	vast-tools
Ago3	MmuINT0056241	chr4:126346631-126346830=126332732-126345582:-	RI	vast-tools
Akap1	MmuINT0013299	chr11:88844259-88845865=88841169-88841302:-	RI	vast-tools
Alkbh8	MmuINT0014224	chr9:3335478-3335594=3338456-3338591:+	RI	vast-tools
Amn1	MmuINT0014675	chr6:149183394-149183538=149170833-149171050:-	RI	vast-tools
Amt	MmuINT0014777	chr9:108297128-108297295=108297712-108297792:+	RI	vast-tools
Anapc1	MmuINT0014874	chr2:128633337-128633415=128632598-128632672:-	RI	vast-tools
Anapc1	MmuINT0014883	chr2:128623878-128623955=128623496-128623563:-	RI	vast-tools
Anapc1	MmuINT0014884	chr2:128623496-128623563=128622373-128622511:-	RI	vast-tools
Anapc4	MmuINT0014931	chr5:52861243-52861340=52861949-52862010:+	RI	vast-tools
Ank3	MmuINT1004281	chr10:69982116-69982197=69994372-69994446:+	RI	vast-tools
Ank3	MmuEX0004933	chr10:69982161+69982197,69986083+69986089-69988632,69994372	SE	vast-tools
Ank3	MmuEX1003811	chr10:69988632,69993256+69993545-69993762,69994372	SE	vast-tools
Ankrd16	MmuALTA0001609-1/2	chr2:11783749,11784291+11784197-11784452	A3	vast-tools
Anks3	MmuINT0015910	chr16:4942211-4942294=4941899-4941987:-	RI	vast-tools
Ap3s1	MmuEX1004047	chr18:46783799,46788657-46788704,46789897	SE	vast-tools
Arhgap32	MmuINT0018142	chr9:32255174-32256058=32256713-32257698:+	RI	vast-tools
Arhgef11	MmuEX0005959	chr3:87618792+87681892,87683150-87683164,87683486	SE	vast-tools
Arhgef12	MmuEX0005976	chr9:43042591,43040540-43040596,43027295	SE	vast-tools
Arr3	MmuINT0019272	chrX:100613829-100613913=100614131-100614203:+	RI	vast-tools
Arr3	MmuINT0019276	chrX:100617230-100617253=100617425-100617489:+	RI	vast-tools
Arr3	MmuEX0006256	chrX:100614739,100616900-100616983,100617230	SE	vast-tools
Arr3	MmuEX0006257	chrX:100616983,100617230-100617253,100617425	SE	vast-tools
Asph	MmuEX0006460	chr4:9601307,9598733-9598780,9598301	SE	vast-tools
Atp2b4	MmuEX0006834	chr1:133715054,133711632+133711642-133711822,133706996	SE	vast-tools
Atr	MmuEX0007013	chr9:95903778,95905738-95905858,95907289+95907296+95907393	SE	vast-tools
Bag6	MmuEX0007606	chr17:35146353,35146757-35146903,35147141	SE	vast-tools
Baz2b	MmuEX0007715	chr2:59933671,59933406+59933412-59933438,59932170	SE	vast-tools
BC034090	MmuINT1005255	chr1:155217382-155217566=155212471-155213713:-	RI	vast-tools
BC065403	MmuINT1005323	chr9:72409670-72409867=72408644-72409488:-	RI	vast-tools
Bcl9l	MmuINT0024552	chr9:44500745-44501130=44501802-44501921:+	RI	vast-tools
Cacna1g	MmuEX0008787	chr11:94425764+94425785,94423482+94423671-94423724,94418970	SE	vast-tools
Cant1	MmuINT1006021	chr11:118417574-118417654=118410853-118411511:-	RI	vast-tools
Ccdc191	MmuINT0002641	chr16:43944639-43944730=43946669-43946751:+	RI	vast-tools
Ccnl2	MmuEX0010080	chr4:155817987,155818539+155818544+155818587-155818664+155818	SE	vast-tools
Cdon	MmuEX0010675	chr9:35473565,35475730-35475743,35476877	SE	vast-tools

Cecr2	MmuALTD0002958-1/2	chr6:120756551-120756853+120756937,120757566	A5	vast-tools
Cep135	MmuINT0034608	chr5:76638064-76638266=76638922-76639026:+	RI	vast-tools
Cep164	MmuEX0010917	chr9:45809704,45803173-45803328,45802768+45802777	SE	vast-tools
Cep170	MmuALTD0003021-2/3	chr1:176742552+176742501+176742471-176742648,176740089	A5	vast-tools
Cep170	MmuINT0034680	chr1:176742501-176742648=176739919-176740089:-	RI	vast-tools
Cep250	MmuINT0034749	chr2:155961872-155962154=155962900-155962956:+	RI	vast-tools
Cep250	MmuEX1006533	chr2:155962154,155962355-155962437,155962900	SE	vast-tools
Cep44	MmuALTD1039454-1/3	chr8:56550688+56550598+56550545-,56547524	A5	vast-tools
Cep57l1	MmuINT1006779	chr10:41728628-41728714=41723861-41723938:-	RI	vast-tools
Cerkl	MmuEX0011054	chr2:79342303,79342162-79342221,79341512	SE	vast-tools
Chd7	MmuINT0035747	chr4:8839425-8839620=8840389-8840556:+	RI	vast-tools
Ckap5	MmuEX0011484	chr2:91599378,91599604-91599627,91600641	SE	vast-tools
Clasrp	MmuINT1007235	chr7:19584155-19584201=19582232-19582314:-	RI	vast-tools
Colgalt1	MmuINT0069478	chr8:71621764-71621896=71622703-71622830:+	RI	vast-tools
Coq2	MmuINT0041061	chr5:100667892-100668058=100663580-100663701:-	RI	vast-tools
Coro1b	MmuINT0041140	chr19:4148619-4148736=4149342-4149544:+	RI	vast-tools
Coro1b	MmuINT0041149	chr19:4152522-4152579=4153227-4153490:+	RI	vast-tools
Crtc3	MmuINT0042502	chr7:80595610-80595908=80592678-80592878:-	RI	vast-tools
Cryz	MmuEX0012829	chr3:154606531,154611387+154611410-154611573,154613822	SE	vast-tools
Csad	MmuINT0042658	chr15:102178527-102178616=102176998-102177786:-	RI	vast-tools
Cspp1	MmuEX0012960	chr1:10085952,10086576-10086734,10088051+10088104	SE	vast-tools
Ctso	MmuINT0043815	chr3:81942193-81942332=81944862-81945029:+	RI	vast-tools
Cul9	MmuINT0044173	chr17:46502130-46502247=46501753-46501886:-	RI	vast-tools
Cutc	MmuALTA0004876-1/3	chr19:43760724,43762903+43762873+43762858-43763006	A3	vast-tools
D11Wsu47e	MmuEX0013467	chr11:113689221+113689504,113691729-113691833,113692395	SE	vast-tools
Daxx	MmuINT0046707	chr17:33913273-33913726=33913843-33914062:+	RI	vast-tools
Dda1	MmuINT0047323	chr8:71469199-71469325=71472037-71472117:+	RI	vast-tools
Decr2	MmuINT0048086	chr17:26082876-26083093=26081211-26082261:-	RI	vast-tools
Dlat	MmuEX0014769	chr9:50658165,50657845-50657998,50653748	SE	vast-tools
Dlg4	MmuINT0049936	chr11:70018466-70018949=70026923-70026988:+	RI	vast-tools
Dlg4	MmuEX6086898	chr11:70017233+70017376,70017786-70017826,70026923	SE	vast-tools
Dlg4	MmuEX6086899	chr11:70017233+70017376,70017422-70017520,70026923	SE	vast-tools
Dmxl1	MmuEX1008627	chr18:49903028,49903414-49903527,49912607+49912643	SE	vast-tools
Dnajc27	MmuALTD0004351-1/2	chr12:4106802-4106984+4107043,4107159	A5	vast-tools
Doc2b	MmuEX1008819	chr11:75786093,75785517-75785543,75781695	SE	vast-tools
Dock3	MmuINT0052236	chr9:106969753-106969880=106967077-106967223:-	RI	vast-tools
Drd4	MmuINT0053252	chr7:141292006-141292313=141293713-141293825:+	RI	vast-tools
E2f6	MmuEX6080292	chr12:16820384,16822093-16822207,16824570	SE	vast-tools
Edem2	MmuEX0016239	chr2:155713325+155713338,155712712+155712719-155713123,155710	SE	vast-tools
Ehmt1	MmuALTA0006158-2/3	chr2:24877455,24863893-24863905+24863908+24863929	A3	vast-tools
Eif4g1	MmuEX0016609	chr16:20672876,20673599-20673655,20673868	SE	vast-tools
Eif4g3	MmuINT1009498	chr4:138177917-138178010=138180490-138180607:+	RI	vast-tools
Elmo2	MmuEX0016707	chr2:165304900,165303666-165303701,165301991	SE	vast-tools
Epc1	MmuALTD0004926-1/2	chr18:6455193+6455189-6455334,6454393	A5	vast-tools
Eps15l1	MmuALTD0004967-3/4	chr8:72379084+72378991+72378985+72378980-72379155,72373946	A5	vast-tools
Esyt2	MmuINT0059285	chr12:116365555-116365928=116366930-116367028:+	RI	vast-tools
Exoc1	MmuINT0059642	chr5:76554100-76554205=76557819-76557863:+	RI	vast-tools
Faim	MmuEX0017795	chr9:98986512,98990915-98990971,98992101	SE	vast-tools
Fam160a2	MmuEX0018027	chr7:105399648+105399884,105391577-105391667,105390352	SE	vast-tools
Fam161a	MmuINT1009968	chr11:23008465-23008626=23013539-23013647:+	RI	vast-tools
Fam178a	MmuINT0061216	chr19:44941459-44942426=44943449-44943519:+	RI	vast-tools
Fam188a	MmuEX0018166	chr2:12419159,12400023-12400137,12386679	SE	vast-tools
Farsb	MmuINT0062568	chr1:78467971-78468032=78466967-78467018:-	RI	vast-tools
Fbxo22	MmuINT0063237	chr9:55209353-55209491=55213909-55213996:+	RI	vast-tools
Flna	MmuEX0019330	chrX:74233149,74232967-74232990,74230753	SE	vast-tools
Fsd1l	MmuEX0019699	chr4:53686517,53687207-53687239,53693984+53693987	SE	vast-tools
Galk2	MmuINT0067250	chr2:125975260-125975461=125983055-125983587:+	RI	vast-tools
Ganc	MmuEX0020057	chr2:120441600,120444138-120444239,120446299+120446365+120446	SE	vast-tools
Gm26954	MmuEX1012533	chr5:14723524,14713956-14714451,14702198	SE	vast-tools
Gm45106	MmuINT1018370	chr7:38053800-38053952=38050147-38050289:-	RI	vast-tools
Golga4	MmuEX0021439	chr9:118536943,118537242-118537325,118538877	SE	vast-tools
Gpatch8	MmuEX0021550	chr11:102500803,102488747-102488796,102487630	SE	vast-tools
Gpcpd1	MmuEX0021570	chr2:132564627,132558607-132558682,132556976	SE	vast-tools
Gpd1l	MmuINT0072646	chr9:114904775-114905008=114903661-114903767:-	RI	vast-tools
Gps2	MmuINT0073431	chr11:69916198-69916293=69916420-69916591:+	RI	vast-tools
Gramd1a	MmuEX0021779	chr7:31133991,31133220-31133231,31132661+31132865	SE	vast-tools
Gria4	MmuINT0073843	chr9:4427030-4427144=4424320-4424454:-	RI	vast-tools
Gripap1	MmuEX0021927	chrX:7803400,7803594-7803686,7804360	SE	vast-tools
Grm6	MmuINT0074272	chr11:50853190-50853406=50853965-50854100:+	RI	vast-tools
Gse1	MmuEX0022002	chr8:120572906,120574171-120574278,120574934	SE	vast-tools
Gtf2f1	MmuALTD1001106-2/4	chr17:57004151+57004125+57004122+57004118-57004177,57004053	A5	vast-tools
Gtf3c2	MmuINT0074960	chr5:31166361-31166386=31165886-31166015:-	RI	vast-tools
Gtpbp8	MmuINT0075096	chr16:44745395-44745493=44743738-44743868:-	RI	vast-tools

Hcls1	MmuALTD1008958-1/3	chr16:36955260-36955370+36955581+36955604,36955964	A5	vast-tools
Hcn1	MmuEX1014480	chr13:117971731,117971926-117971974,117975252+117975278+11797	SE	vast-tools
Hddc3	MmuINT0076498	chr7:80343716-80343956=80345648-80346714:+	RI	vast-tools
Hira	MmuEX0022940	chr16:18894836,18895903-18896046,18896454	SE	vast-tools
Hsf1	MmuEX0023331	chr15:76500053,76500131-76500196,76500267	SE	vast-tools
Hsf2	MmuINT0079219	chr10:57497497-57497621=57499416-57499491:+	RI	vast-tools
Htra2	MmuINT0079628	chr6:83053921-83054571=83053613-83053817:-	RI	vast-tools
Il18bp	MmuINT0081399	chr7:102017597-102017664=102017287-102017351:-	RI	vast-tools
Impdh2	MmuINT1021314	chr9:108563596-108563691=108564635-108564778:+	RI	vast-tools
Impg2	MmuEX0024103	chr16:56252347,56254163-56254248,56257904+56258145+56258148	SE	vast-tools
Ints13	MmuALTA0000561-1/3	chr6:146556131,146555004-146555052+146555069+146555098	A3	vast-tools
Ints13	MmuINT0004972	chr6:146552372-146552511=146550639-146550774:-	RI	vast-tools
Ints13	MmuINT0004973	chr6:146550639-146550774=146550087-146550164:-	RI	vast-tools
Ints6	MmuINT0082593	chr14:62700631-62700862=62696593-62696955:-	RI	vast-tools
Ipo9	MmuINT0082896	chr1:135406517-135406575=135405821-135405972:-	RI	vast-tools
Irf3	MmuINT0083309	chr7:45000645-45001022=45001673-45001788:+	RI	vast-tools
Katnal2	MmuALTD0007316-1/3	chr18:77011990+77011984+77011971-77012031,77011045	A5	vast-tools
Kcnc3	MmuEX0025027	chr7:44598575,44600674+44600869-44600928,44601869	SE	vast-tools
Kctd9	MmuEX0025240	chr14:67733442,67734141-67734290,67737560+67737606	SE	vast-tools
Kdelc1	MmuINT0086047	chr1:44110735-44110944=44109994-44110083:-	RI	vast-tools
Kif1b	MmuEX0025491	chr4:149206718,149205686-149205724+149205886,149204288+149204	SE	vast-tools
Kif1b	MmuEX0025498	chr4:149198432,149195473-149195499,149192687	SE	vast-tools
Kif1c	MmuEX0025504	chr11:70719093,70723904-70723925,70724048	SE	vast-tools
Kif21b	MmuINT1021721	chr1:136173472-136173651=136174037-136177998:+	RI	vast-tools
Kmt2c	MmuEX0029184	chr5:25304678,25304097-25304119,25302938	SE	vast-tools
Kmt2d	MmuEX0029168	chr15:98837075,98836581-98836628,98835449+98835467	SE	vast-tools
Ktn1	MmuINT0089074	chr14:47724878-47724970=47726226-47726309:+	RI	vast-tools
Ktn1	MmuEX0025962	chr14:47724970,47725929-47726000,47726226	SE	vast-tools
Lap3	MmuINT0089840	chr5:45511108-45511217=45511881-45512691:+	RI	vast-tools
Las1l	MmuINT0090015	chrX:95951310-95951356=95950337-95950446:-	RI	vast-tools
Las1l	MmuINT0090017	chrX:95947770-95947855=95946867-95947073:-	RI	vast-tools
Ldah	MmuINT1022169	chr12:8275808-8275890=8283900-8285759:+	RI	vast-tools
Letm2	MmuINT0090653	chr8:25597120-25597513=25596362-25596445:-	RI	vast-tools
Limk2	MmuINT1022227	chr11:3353277-3353463=3352291-3352377:-	RI	vast-tools
Llgl2	MmuEX0026553	chr11:115854575,115854732+115854812-115854859,115855060+11585	SE	vast-tools
Lnpk	MmuINT1022317	chr2:74554988-74555046=74551061-74551101:-	RI	vast-tools
Lrif1	MmuEX0001851	chr3:106733142,106733337-106733606,106734358	SE	vast-tools
Luc7l2	MmuINT0093874	chr6:38551465-38551908=38570510-38570604:+	RI	vast-tools
Man2a2	MmuEX1016698	chr7:80356342,80355988-80356062,80353362	SE	vast-tools
Map3k12	MmuEX0027710	chr15:102509290,102508328-102508463,102505568	SE	vast-tools
Map7d2	MmuEX0029932	chrX:159490784+159490814,159491768-159491824,159492324+159492	SE	vast-tools
Mapk1ip1	MmuEX0027832	chr7:138845953+138845957,138840577-138840934,138836926+138837	SE	vast-tools
Mapk8ip3	MmuALTD0008199-1/2	chr17:24918088+24917995-24918136,24914705	A5	vast-tools
Mapk8ip3	MmuEX0027856	chr17:24923816,24920472-24920495,24918136+24918139	SE	vast-tools
Mapk9	MmuEX0027861	chr11:49854382,49854737-49854977,49863544+49863561	SE	vast-tools
Mars	MmuEX0027974	chr10:127296873,127296717-127296740,127296626+127296633	SE	vast-tools
Mbnl1	MmuEX0028097	chr3:60613520,60614683-60614736,60615620+60615725	SE	vast-tools
Mettl4	MmuEX0028751	chr17:94747617,94743932-94743994,94740755	SE	vast-tools
Mier2	MmuINT0098752	chr10:79542405-79542481=79541514-79541934:-	RI	vast-tools
Mknk1	MmuINT0099004	chr4:115862977-115863056=115864539-115864612:+	RI	vast-tools
Mknk1	MmuEX0029104	chr4:115863056,115864539-115864612,115866462	SE	vast-tools
Mok	MmuINT0154100	chr12:110809909-110810023=110808205-110808405:-	RI	vast-tools
Mpdz	MmuEX0029495	chr4:81320346,81317594-81317704,81310319	SE	vast-tools
Mpdz	MmuEX0029496	chr4:81320346,81313500-81313598,81310319	SE	vast-tools
Mplkip	MmuINT1023238	chr13:17695413-17695820=17696842-17699105:+	RI	vast-tools
Mycbp2	MmuEX0030304	chr14:103172567,103160841-103160942,103156767	SE	vast-tools
Myh10	MmuEX0030346	chr11:68745419,68745935-68745964,68751942	SE	vast-tools
Myo9a	MmuEX0030580	chr9:59788514,59790036-59790092,59801911+59801914	SE	vast-tools
Nepro	MmuINT0023104	chr16:44732058-44732119=44734541-44734797:+	RI	vast-tools
Nfat5	MmuINT0108001	chr8:107358634-107358766=107361744-107361824:+	RI	vast-tools
Nup205	MmuINT0111809	chr6:35203963-35204013=35205325-35205534:+	RI	vast-tools
Nup85	MmuEX1019020	chr11:115570120,115572736-115572759,115577930	SE	vast-tools
Orc4	MmuALTA0012564-3/3	chr2:48912562,48910186-48910242+48910245+48910294	A3	vast-tools
Osmr	MmuEX6052516	chr15:6836758,6824473-6824652,6821833	SE	vast-tools
Osmr	MmuEX6052517	chr15:6836758,6823552-6823656,6821833	SE	vast-tools
Parp2	MmuEX1019461	chr14:50817470,50818843-50818870,50819210	SE	vast-tools
Parp8	MmuINT0115515	chr13:116868126-116868198=116867036-116867105:-	RI	vast-tools
Pced1a	MmuINT0060512	chr2:130421890-130422136=130419630-130419890:-	RI	vast-tools
Pdcd6	MmuINT0116698	chr13:74317107-74317326=74316330-74316391:-	RI	vast-tools
Peg3	MmuINT0117794	chr7:6716054-6716157=6712673-6712775:-	RI	vast-tools
Peg3	MmuEX0034424	chr7:6716054,6712950-6713012,6712775	SE	vast-tools
Per3	MmuINT0117918	chr4:151031913-151031991=151029136-151029242:-	RI	vast-tools
Pfkfb2	MmuINT0118160	chr1:130698679-130698743=130689182-130691148:-	RI	vast-tools

Phc3	MmuEX0034692	chr3:30936285,30935952-30935999+30936020,30931892	SE	vast-tools
Plk5	MmuINT0122346	chr10:80359410-80359505=80360155-80360353:+	RI	vast-tools
Plod3	MmuINT0122402	chr5:136994933-136995058=136996106-136996648:+	RI	vast-tools
Plscr3	MmuINT0122486	chr11:69848214-69848375=69850012-69850173:+	RI	vast-tools
Pnpo	MmuINT0123227	chr11:96943731-96943986=96942410-96942534:-	RI	vast-tools
Poc1b	MmuINT0123281	chr10:99152801-99152934=99155069-99155137:+	RI	vast-tools
Polr3c	MmuINT0123968	chr3:96716429-96716480=96715634-96715694:-	RI	vast-tools
Pot1b	MmuINT0124260	chr17:55654767-55654872=55652025-55653518:-	RI	vast-tools
Ppip5k1	MmuINT0124823	chr2:121327645-121327770=121326852-121326914:-	RI	vast-tools
Ppip5k1	MmuINT0124824	chr2:121326852-121326914=121321655-121321792:-	RI	vast-tools
Ppip5k1	MmuEX0036605	chr2:121327645,121326852-121326914,121321792	SE	vast-tools
Prdm2	MmuEX0036965	chr4:143161664+143162003+143162056,143145390-143145446,143142	SE	vast-tools
Prkab2	MmuINT0126182	chr3:97666582-97666715=97667359-97667427:+	RI	vast-tools
Prmt9	MmuINT1027453	chr8:77560807-77560976=77562416-77562516:+	RI	vast-tools
Prr14	MmuINT1027506	chr7:127470985-127471119=127471906-127471977:+	RI	vast-tools
Ptprz1	MmuINT0129630	chr6:22987331-22987457=22994518-22994564:+	RI	vast-tools
Qrsl1	MmuINT0130195	chr10:43881473-43881590=43876472-43876677:-	RI	vast-tools
Ranbp2	MmuEX0038718	chr10:58492684,58492802-58493136,58493663	SE	vast-tools
Rbbp6	MmuEX0038994	chr7:122997305,122997596-122997697,122998525	SE	vast-tools
Rbm26	MmuEX0039116	chr14:105140316,105139091-105139162,105132007	SE	vast-tools
Rdh14	MmuINT0133286	chr12:10390772-10391226=10394538-10395562:+	RI	vast-tools
Rfx1	MmuINT0134015	chr8:84087706-84088072=84090111-84090292:+	RI	vast-tools
Rims2	MmuEX0039866	chr15:39462610,39469155-39469202,39472334	SE	vast-tools
Rims2	MmuEX0039872	chr15:39511374,39517806-39517871,39534838	SE	vast-tools
Riok1	MmuINT0135120	chr13:38048880-38048992=38049076-38049156:+	RI	vast-tools
Rnf40	MmuINT1030057	chr7:127590507-127590713=127591315-127591436:+	RI	vast-tools
RP23-273K11.3	MmuINT1028327	chr10:96523860-96523982=96495721-96497628:-	RI	vast-tools
Rpgrip1	MmuEX0040375	chr14:52141240+52141273,52145033+52145038-52145485,52149216	SE	vast-tools
Rpgrip1	MmuEX0040377	chr14:52141240+52141273,52147210+52147215-52147552,52149216	SE	vast-tools
Rpusd1	MmuINT0137257	chr17:25727751-25727847=25728203-25728391:+	RI	vast-tools
Rrh	MmuEX0040578	chr3:129815560,129813250+129813254-129813349,129811779	SE	vast-tools
Rrnad1	MmuINT0023020	chr3:87929856-87930144=87927924-87928048:-	RI	vast-tools
Samd8	MmuALTD0012351-2/3	chr14:-21750780+21750836+21751047,21774963	A5	vast-tools
Samm50	MmuINT0138732	chr15:84211037-84211178=84214126-84214303:+	RI	vast-tools
Scamp2	MmuINT0139192	chr9:57582852-57582953=57587080-57587200:+	RI	vast-tools
Scyl2	MmuALTA0015976-2/2	chr10:89645677,89645462-89645554+89645557	A3	vast-tools
Sec31a	MmuALTD0012543-2/2	chr5:100373425+100373380-100373484,100368352	A5	vast-tools
Sema4g	MmuEX0041614	chr19:44989357+44989679,44991152+44991173-44991358,44992138	SE	vast-tools
Senp3	MmuINT0141345	chr11:69681663-69681846=69680143-69680850:-	RI	vast-tools
Setdb2	MmuINT0142227	chr14:59412091-59412184=59409178-59409518:-	RI	vast-tools
Sgip1	MmuEX0042044	chr4:102928912,102933565-102933630,102956099	SE	vast-tools
Sirt1	MmuINT0143937	chr10:63321749-63322300=63319005-63320964:-	RI	vast-tools
Sirt3	MmuINT0143961	chr7:140876354-140876454=140869489-140869650:-	RI	vast-tools
Slc15a2	MmuINT0144793	chr16:36756050-36756096=36754442-36754552:-	RI	vast-tools
Slc17a7	MmuINT0144968	chr7:45168715-45168967=45169817-45169935:+	RI	vast-tools
Slc17a7	MmuEX1023774	chr7:45168967,45169511-45169585,45169817	SE	vast-tools
Slc24a2	MmuEX0042807	chr4:87226883,87076119-87076157,87073296	SE	vast-tools
Slc25a19	MmuINT0145530	chr11:115623872-115624027=115620877-115621047:-	RI	vast-tools
Slc25a25	MmuINT0145585	chr2:32421303-32421390=32420305-32420416:-	RI	vast-tools
Slc25a25	MmuEX0042866	chr2:32421303,32420656-32420691,32420416	SE	vast-tools
Slc25a33	MmuINT1031407	chr4:149752404-149752470=149749049-149749329:-	RI	vast-tools
Slc4a3	MmuEX1024056	chr1:75549065,75549833-75550053,75550504	SE	vast-tools
Slc4a7	MmuEX0043431	chr14:14746033+14746083,14747733-14747792,14748466+14748500	SE	vast-tools
Slc9a5	MmuEX0043617	chr8:105359453,105361489+105361561-105361638,105363162+105363	SE	vast-tools
Smad4	MmuINT0148509	chr18:73658568-73658684=73657195-73657245:-	RI	vast-tools
Snx32	MmuINT0150167	chr19:5510333-5510489=5498720-5498824:-	RI	vast-tools
Sobp	MmuEX0044324	chr10:43127779,43074593-43074688,43022918	SE	vast-tools
Sos1	MmuINT0150601	chr17:80449258-80449401=80445602-80445712:-	RI	vast-tools
Spsb3	MmuINT0152087	chr17:24886680-24887105=24887780-24887917:+	RI	vast-tools
Spsb3	MmuINT0152088	chr17:24887780-24887917=24890283-24890460:+	RI	vast-tools
Srsf6	MmuEX0044998	chr2:162932142,162932815-162932979+162933082,162933426	SE	vast-tools
Srsf9	MmuEX0045003	chr5:115330658,115331450+115331461-115331539,115332117	SE	vast-tools
Supt20	MmuINT1032477	chr3:54720406-54720461=54722521-54722640:+	RI	vast-tools
Surf2	MmuINT0155262	chr2:26917428-26917531=26918873-26919055:+	RI	vast-tools
Svil	MmuEX0045852	chr18:5082930,5086535-5086624,5086710	SE	vast-tools
Taf1a	MmuALTD1034861-1/2	chr1:183408609-183408724+183408763,183409235	A5	vast-tools
Tctn1	MmuEX0046772	chr5:122258835,122257470-122257621,122254583	SE	vast-tools
Tdg	MmuINT1032868	chr10:82644615-82644709=82647222-82647393:+	RI	vast-tools
Tfpt	MmuALTD0014235-1/2	chr7:3620777+3620747-3620946,3620474	A5	vast-tools
Thoc1	MmuINT0159821	chr18:9968751-9968833=9970255-9970328:+	RI	vast-tools
Tjp2	MmuALTD0014341-2/3	chr19:24111059+24111025+24111021-24111116,24109810	A5	vast-tools
Tm9sf1	MmuINT0160864	chr14:55640385-55640570=55637859-55638132:-	RI	vast-tools
Tmem201	MmuINT0161899	chr4:149729536-149729712=149727902-149728251:-	RI	vast-tools

Tnfaip3	MmuINT1033448	chr10:19015153-19015410=19011481-19011790:-	RI	vast-tools
Tnk2	MmuEX0048311	chr16:32677985,32678669-32678713+32678785,32679459	SE	vast-tools
Trip11	MmuALTD0014820-2/4	chr12:101893777+101893569+101893459+101893431-101893793,10189	A5	vast-tools
Trip12	MmuEX0048991	chr1:84814823,84795690-84795815,84794327	SE	vast-tools
Ttc3	MmuEX0049521	chr16:94384066,94384346-94384388,94384784	SE	vast-tools
Tub	MmuINT0168060	chr7:109029554-109029725=109030124-109034460:+	RI	vast-tools
Tubgcp4	MmuEX0049905	chr2:121184499,121184702-121184793+121184826,121188132	SE	vast-tools
Tubgcp5	MmuINT0168218	chr7:55814860-55815128=55816458-55816656:+	RI	vast-tools
Uggt2	MmuEX0050479	chr14:119034842,119032629+119032691+119032742-119032780+11903	SE	vast-tools
Uhrf2	MmuINT0170215	chr19:30056235-30056494=30071243-30071461:+	RI	vast-tools
Unc119b	MmuINT0170383	chr5:115127011-115127183=115122550-115125500:-	RI	vast-tools
Unc13b	MmuEX1027354	chr4:43250302,43254307-43254345,43255475	SE	vast-tools
Usp16	MmuEX0050848	chr16:87458742,87461965-87462140,87464735	SE	vast-tools
Uspl1	MmuEX0051128	chr5:149184806,149187708-149187834+149187863+149187876,1491883	SE	vast-tools
Vps37a	MmuINT0173992	chr8:40541025-40541093=40543810-40543953:+	RI	vast-tools
Wdr17	MmuEX0051817	chr8:54724288,54703834-54703939,54696397	SE	vast-tools
Wdr43	MmuINT0175288	chr17:71640234-71640320=71641287-71641418:+	RI	vast-tools
Zcchc2	MmuEX0052809	chr1:106004176,106006015-106006086,106011702	SE	vast-tools
Zcchc9	MmuINT1035417	chr13:91800611-91800703=91798823-91798891:-	RI	vast-tools
Zdhhc20	MmuINT0178277	chr14:57840829-57840918=57839090-57839205:-	RI	vast-tools
Zdhhc20	MmuINT0178279	chr14:57837591-57837667=57832702-57836646:-	RI	vast-tools
Zdhhc7	MmuINT0178338	chr8:120087754-120088084=120086629-120086753:-	RI	vast-tools
Zfand3	MmuEX0052966	chr17:30005308,30060810-30060850,30135323	SE	vast-tools
Zfp207	MmuEX0053090	chr11:80391992,80393084-80393176,80394286	SE	vast-tools
Zfp335os	MmuINT1035564	chr2:164911354-164911750=164919224-164919953:+	RI	vast-tools
Zfp512	MmuEX0053395	chr5:31452577+31452589,31453155-31453213+31453311,31465446+314	SE	vast-tools
Zfp930	MmuEX0053690	chr8:69226469,69226672-69226729,69227843	SE	vast-tools
Zfp956	MmuEX0054156	chr6:47956170,47957667-47957724,47962525+47962531	SE	vast-tools
Zfp983	MmuEX0001300	chr17:21650692+21650773+21650872,21651803-21651888+21651894+2	SE	vast-tools
Zkscan17	MmuALTD0016312-1/2	chr11:59503677+59503384-59503763,59503100	A5	vast-tools
Zkscan2	MmuINT0180354	chr7:123484940-123485431=123475384-123480772:-	RI	vast-tools
Zkscan2	MmuEX0053871	chr7:123484940,123482641-123482684,123480772	SE	vast-tools
Zmym3	MmuALTA0020876-2/3	chrX:101417287,101416909-101416975+101416981+101417036	A3	vast-tools
Zmynd8	MmuEX0053988	chr2:165791905,165787164-165787243+165787247,165785738	SE	vast-tools

STRIATUM				
Gene	*Event*	*Genomic location*	*Type of event*	*Software*
2010300C02Rik	MmuEX0000752	chr1:37719570,37676342-37676779,37644640	SE	vast-tools
2210408F21Rik	MmuEX1001513	chr6:31336062,31336804-31336857,31337211+31337230	SE	vast-tools
2310035C23Rik	MmuEX0000915	chr1:105750410,105750973-105751043,105753503	SE	vast-tools
2410089E03Rik	MmuEX0001021	chr15:8223150+8223164,8225339-8225416,8226913	SE	vast-tools
4933431E20Rik	SE	4933431E20Rik,chr3,-,107801904,107801972,107801366,107801531,107	SE	rMATS
9630013A20Rik	A3SS	9630013A20Rik,chr14,-,84709652,84709730,84709652,84709682,847133	A3	rMATS
9630013A20Rik	A3SS	9630013A20Rik,chr14,-,84709652,84709780,84709652,84709682,847133	A3	rMATS
9630013A20Rik	A3SS	9630013A20Rik,chr14,-,84709652,84710144,84709652,84709682,847133	A3	rMATS
Abcc8	RI	Abcc8,chr7,-,45755232,45756112,45755232,45755366,45756008,457561	RI	rMATS
Ablim2	MmuEX0003309	chr5:35841401,35843168-35843266,35848804+35848808	SE	vast-tools
Ache	A5	5:137286319-137288271:137286290-137288271:+	A5	SUPPA2
Ache	AF	5:137285781:137285896-137288271:137286028:137286319-137288271:	Exc	SUPPA2
Ache	AF	5:137286028:137286319-137288271:137286546:137286629-137288271:	Exc	SUPPA2
Acly	MmuEX0003484	chr11:100504203,100503160-100503189,100498762	SE	vast-tools
Adamts3	MmuINT0011386	chr5:89710657-89710740=89708611-89708767:-	RI	vast-tools
Adcy3	MmuALTA0001209-1/2	chr12:4206978,4208580+4208577-4208661	A3	vast-tools
Adgrf5	MmuALTD0006215-1/2	chr17:43403918-43404043+43404097,43410502	A5	vast-tools
Adpgk	RI	Adpgk,chr9,+,59217510,59220519,59217510,59217631,59220322,592205	RI	rMATS
Agap2	SE	10:126925227-126926011:126926070-126926296:+	SE	SUPPA2
Alkbh1	SE	Alkbh1,chr12,-,87488412,87488550,87487075,87487184,87490411,87490	SE	rMATS
Ank3	MmuEX0004910	chr10:69927818,69929447-69929458,69932394	SE	vast-tools
Ankrd40	A3	11:94229347-94230379:94229347-94230459:+	A3	SUPPA2
Arap3	SE	Arap3,chr18,-,38107154,38107193,38105680,38106704,38107417,38107	SE	rMATS
Arhgap26	SE	Arhgap26,chr18,+,39490605,39490716,39439754,39439905,39496169,39	SE	rMATS
Arpp19	MmuINT0019241	chr9:75037907-75037961=75052233-75052355:+	RI	vast-tools
Arpp21	MmuINT0019257	chr9:112206245-112206344=112187590-112187766:-	RI	vast-tools
Asah2	A3SS	Asah2,chr19,-,32038686,32038805,32038686,32038802,32078949,32079	A3	rMATS
Atat1	MmuEX0006557	chr17:35904290,35904108-35904176,35902054	SE	vast-tools
Atg16l1	MmuEX0006621	chr1:87774231,87775081-87775128+87775157,87775757	SE	vast-tools
Atg16l1	MXE	Atg16l1,chr1,+,87702534,87702591,87702802,87702850,87701866,87701	Exc	rMATS
Atm	SE	Atm,chr9,-,53399849,53400021,53399205,53399370,53401410,53401610	SE	rMATS
Atp1b1	AF	1:164270919-164271175:164271288:164270919-164281058:164281346:	Exc	SUPPA2
Banp	A3SS	Banp,chr8,+,122733829,122733901,122733838,122733901,122732290,12	A3	rMATS
Bcor	A3SS	Bcor,chrX,-,11914541,11914886,11914541,11914784,11915233,1191549	A3	rMATS
Bms1	MmuEX0008083	chr6:118418409,118417388-118417578,118416542	SE	vast-tools
Brca2	A5	5:150446555-150446640:150446361-150446640:+	A5	SUPPA2

Brca2	A5	5:150446555-150446640:150446415-150446640:+	A5	SUPPA2
Brd9	MmuALTA0002878-1/3	chr13:73942054,73942694+73942691+73942631-7394280(	A3	vast-tools
Bsg	A3	10:79546236-79546459:79546236-79546472:+	A3	SUPPA2
Btaf1	MmuINT0026075	chr19:36983449-36983652=36986455-36986881:+	RI	vast-tools
C1qtnf6	A3SS	C1qtnf6,chr15,-,78407545,78409598,78407545,78409387,78411427,7841	A3	rMATS
C230004F18Rik	MmuINT0026682	chrX:61122012-61122159=61133366-61133503:+	RI	vast-tools
Cables1	SE	Cables1,chr18,+,12038981,12039059,12021793,12021886,12056416,120	SE	rMATS
Cacna1a	MmuEX0008722	chr8:84594220,84597480-84597485,84601389	SE	vast-tools
Calm2	SE	17:87743362-87750124:87750154-87754221:-	SE	SUPPA2
Calm3	AF	7:16653590-16657622:16657754:16653590-16657766:16658039:	Exc	SUPPA2
Camk2b	MmuEX0008966	chr11:5982442+5982447+5982501,5981070-5981114,5979721	SE	vast-tools
Capzb	SE	4:139015143-139016172:139016284-139018314:+	SE	SUPPA2
Carf	MmuINT0029144	chr1:60141335-60141568=60143994-60144156:+	RI	vast-tools
Carmil2	MmuEX0039940	chr8:105697438,105697561-105697641,105697868	SE	vast-tools
Carmil3	MmuINT0092889	chr14:55501459-55501593=55501830-55501893:+	RI	vast-tools
Ccdc39	MmuINT0030836	chr3:33825391-33825555=33821377-33821514:-	RI	vast-tools
Ccdc62	A3SS	Ccdc62,chr5,+,124089814,124089930,124089863,124089930,124089261,	A3	rMATS
Ccdc88c	MmuEX0009979	chr12:100928827,100924761+100925212-100925650,100923368	SE	vast-tools
Ccnd2	MmuINT0031645	chr6:127148664-127148879=127146023-127146182:-	RI	vast-tools
Ccne2	A3	4:11192717-11192815:11192717-11192818:+	A3	SUPPA2
Ccnl2	MmuINT0031759	chr4:155815243-155815363=155817923-155817987:+	RI	vast-tools
Cdc16	MmuINT0032686	chr8:13768561-13768634=13769377-13769502:+	RI	vast-tools
Cdk11b	SE	4:155709398-155709642:155709796-155709988:+	SE	SUPPA2
Cdk11b	SE	4:155710111-155710184:155710230-155711231:+	SE	SUPPA2
Celf5	MmuALTA0003841-2/3	chr10:81464340,81462669-81462768+81462771+81462812	A3	vast-tools
Cep70	MmuALTD0003052-1/3	chr9:99278110-99278157+99278161+99278198,99281028	A5	vast-tools
Cfap69	MXE	Cfap69,chr5,-,5671919,5672043,5675749,5675927,5669166,5669215,567	Exc	rMATS
Cnot2	MmuALTA0004346-1/4	chr10:116549033,116537221-116537278+116537443+116537446+11653	A3	vast-tools
Cobl	SE	11:12293917-12315105:12315179-12319594:-	SE	SUPPA2
Cog5	MmuINT1007445	chr12:31925645-31925791=31937177-31937630:+	RI	vast-tools
Copg1	MmuINT1007533	chr6:87890190-87890269=87891949-87892024:+	RI	vast-tools
Cpne8	AF	15:90533486-90533578:90533641:90533486-90563371:90563591:	Exc	SUPPA2
Crocc	SE	Crocc,chr4,-,140774082,140774166,140773946,140774008,140778351,14	SE	rMATS
Crtc2	MmuEX0012801	chr3:90262714,90262837+90262914-90262980,90263171	SE	vast-tools
Ctnnal1	SE	Ctnnal1,chr4,-,56838004,56838047,56837731,56837902,56838965,56839	SE	rMATS
Ctsl	A3	13:64514906-64515674:64514738-64515674:-	A3	SUPPA2
D17H6S53E	MmuINT1008182	chr17:35126643-35126785=35126922-35128855:+	RI	vast-tools
D430036J16Rik	SE	D430036J16Rik,chr9,+,81521213,81521236,81513982,81514332,8152459	SE	rMATS
Dbndd1	AL	8:124231457:124232424-124236581:124232426:124233577-124236581:	Exc	SUPPA2
Dbndd1	SE	8:124233577-124235822:124235962-124236581:-	SE	SUPPA2
Ddb2	MmuINT0047368	chr2:91217053-91217230=91216666-91216808:-	RI	vast-tools
Ddb2	RI	2:91064453:91064644-91065160:91065296:-	RI	SUPPA2
Ddb2	SE	2:91047153-91047398:91047575-91047652:-	SE	SUPPA2
Ddb2	SE	2:91047751-91049089:91049234-91064453:-	SE	SUPPA2
Ddx55	MmuEX0014145	chr5:124559317,124560718-124560800,124561856+12456190(	SE	vast-tools
Dnah10	SE	5:124823801-124824667:124824837-124825455:+	SE	SUPPA2
Dnajc13	MmuEX0015182	chr9:104212761,104209869-104209883,104209387	SE	vast-tools
Dop1a	A3SS	Dop1a,chr9,+,86383926,86384036,86383953,86384036,86382848,86382	A3	rMATS
Dopey2	MmuEX0015524	chr16:93780812,93781689-93781724,93782201	SE	vast-tools
Dtna	MmuEX0015806	chr18:23631428,23633218-23633238,23643147	SE	vast-tools
E2f5	A3	3:14653361-14666060:14653361-14666063:+	A3	SUPPA2
E2f6	MmuEX0016112	chr12:16811315,16813847-16813912,16816400	SE	vast-tools
Eif3h	SE	15:51653509-51659784:51659933-51661009:-	SE	SUPPA2
Eif4g3	MmuINT1009498	chr4:138177917-138178010=138180490-138180607:+	RI	vast-tools
Eml1	MmuEX0016781	chr12:108502418,108503183-108503233,108506479	SE	vast-tools
Eml4	MmuEX0016805	chr17:83421735,83425258-83425431,83427223	SE	vast-tools
Enah	MmuEX0016849	chr1:181956383+181956394,181931174-181931185,181930762	SE	vast-tools
Enox2	SE	Enox2,chrX,-,48102009,48102057,48098583,48100511,48102373,481024	SE	rMATS
Epb41l2	SE	Epb41l2,chr10,+,25377475,25377529,25371342,25371489,25377721,253	SE	rMATS
Erc1	SE	Erc1,chr6,-,119802641,119802780,119801346,119802170,119824837,11	SE	rMATS
Erlin2	SE	Erlin2,chr8,+,27514861,27514955,27513893,27514193,27515084,275152	SE	rMATS
Etnk2	SE	Etnk2,chr1,+,133302264,133302348,133300872,133301015,133304643,1	SE	rMATS
Exosc4	A5	15:76211867-76213230:76211863-76213230:+	A5	SUPPA2
Ezh2	RI	Ezh2,chr6,-,47517610,47519340,47517610,47517789,47519299,4751934	RI	rMATS
Ezh2	SE	Ezh2,chr6,-,47518778,47518904,47517610,47517789,47519299,4751934	SE	rMATS
Fam13c	A3	10:70387620-70388859:70387620-70388862:+	A3	SUPPA2
Fam161a	SE	Fam161a,chr11,+,22971493,22971661,22969999,22971133,22973429,22	SE	rMATS
Fam168b	SE	1:34859090-34863101:34863136-34867848:-	SE	SUPPA2
Fam210a	SE	18:68409345-68412338:68412499-68433145:-	SE	SUPPA2
Fam45a	MmuALTA0006911-2/2	chr19:60811673,60814612+60814592-60814761	A3	vast-tools
Fam8a1	MmuALTA1041293-2/2	chr13:46670255,46671213+46671209-46671329	A3	vast-tools
Fancl	MmuEX0018588	chr11:26434500,26459686-26459836,26468335	SE	vast-tools
Fasn	AF	11:120713678-120714052:120714606:120713678-120714856:120715373	Exc	SUPPA2

Fau	A3	19:6108355-6108458:6108355-6108563:+	A3	SUPPA2
Fer	A3SS	Fer,chr17,+,64288474,64288681,64288477,64288681,64280059,6428015	A3	rMATS
Flywch1	MmuEX0019365	chr17:23771415,23770235-23770328,23765905	SE	vast-tools
Gabpb2	RI	Gabpb2,chr3,-,95111041,95112119,95111041,95111236,95111951,95112	RI	rMATS
Galc	MmuINT0067223	chr12:98242642-98242772=98234242-98234397:-	RI	vast-tools
Gigyf1	MmuINT0068843	chr5:137520212-137520376=137520547-137520800:+	RI	vast-tools
Git2	A3SS	Git2,chr5,-,114890339,114890391,114890339,114890388,114891208,114	A3	rMATS
Git2	SE	Git2,chr5,-,114891208,114891214,114890339,114890391,114891339,114	SE	rMATS
Gm1043	SE	Gm1043,chr5,+,37320283,37320829,37317089,37317228,37327246,3732	SE	rMATS
Gm35549	SE	9:122667294-122671781:122671871-122673034:-	SE	SUPPA2
Gmeb1	MmuINT0071653	chr4:132251675-132251832=132245986-132246068:-	RI	vast-tools
Gmppb	RI	Gmppb,chr9,+,107928047,107928441,107928047,107928175,107928258	RI	rMATS
Gpm6b	MmuEX0021614	chrX:166384000,166385391-166385481,166386952	SE	vast-tools
Gpm6b	A3SS	Gpm6b,chrX,+,165168386,165171984,165169947,165171984,165166930	A3	rMATS
Gpm6b	SE	Gpm6b,chrX,+,165168386,165168477,165166930,165166996,165169947	SE	rMATS
Gpr39	AF	1:125604732:125605929-125800107:125767740:125768063-125800107:	Exc	SUPPA2
Gria3	MmuEX0021843	chrX:41654253,41669501-41669615,41672219	SE	vast-tools
Grin1	MmuEX0021891	chr2:25295792,25294438-25294548,25292125+25292128+25292484	SE	vast-tools
Gripap1	MmuEX0021927	chrX:7803400,7803594-7803686,7804360	SE	vast-tools
Grm7	MmuEX0021973	chr6:111495899,111501162+111501510-111501601,111566018	SE	vast-tools
Guf1	MmuINT1020287	chr5:69566361-69566504=69567128-69567261:+	RI	vast-tools
Hecw2	AF	1:54079686-54233063:54233207:54079686-54234135:54234327:	Exc	SUPPA2
Hmgn1	SE	16:95928403-95928511:95928540-95928632:-	SE	SUPPA2
Hnrnpa3	A5	2:75489733-75491810:75489667-75491810:+	A5	SUPPA2
Hps5	RI	Hps5,chr7,-,46424237,46425375,46424237,46424361,46425188,4642537	RI	rMATS
Htra2	MmuEX0023442	chr6:83053001+83053038,83052698-83052726+83052803+83052861,830	SE	vast-tools
Ica1	MmuEX0023542	chr6:8655914+8656362,8653630-8653692+8653770,8653535	SE	vast-tools
Iffo1	A3SS	Iffo1,chr6,+,125129406,125129734,125129576,125129734,125128760,12	A3	rMATS
Ints6	MmuINT0082593	chr14:62700631-62700862=62696593-62696955:-	RI	vast-tools
Iqce	RI	Iqce,chr5,-,140660091,140663261,140660091,140660115,140663170,140	RI	rMATS
Itga7	MXE	Itga7,chr10,+,128777313,128777445,128777690,128777810,128776550,1	Exc	rMATS
Jpx	MmuEX0000698	chrX:103493700+103493947,103496438-103496530+103496593,1035028	SE	vast-tools
Kalrn	MmuALTA0009481-1/2	chr16:34227027,34220056-34220247+34220274	A3	vast-tools
Kcnip2	MmuEX0025056	chr19:45796915+45797091,45796279-45796332,45795704	SE	vast-tools
Kctd17	MmuINT0085965	chr15:78436901-78436994=78438487-78438909:+	RI	vast-tools
Kdm5d	MmuINT0086363	chrY:914025-914183=916641-916760:+	RI	vast-tools
Kdm6a	MmuEX0025354	chrX:18246416,18247567-18247722,18248270	SE	vast-tools
Kif5c	SE	2:49617971-49618554:49618580-49620129:+	SE	SUPPA2
Klc1	MmuINT0087608	chr12:111789325-111789486=111795548-111795678:+	RI	vast-tools
Lmbr1l	RI	Lmbr1l,chr15,-,98806400,98806640,98806400,98806477,98806556,9880	RI	rMATS
Lmtk3	MmuINT0091633	chr7:45783738-45783980=45785569-45785681:+	RI	vast-tools
Lrrfip1	MmuEX0027124	chr1:91068649,91091602-91091667,91100577	SE	vast-tools
Lrrk2	MmuINT0093437	chr15:91779819-91780009=91786966-91787126:+	RI	vast-tools
Madd	MmuEX0027516	chr2:91162028,91160317-91160370,91159305	SE	vast-tools
Map3k4	MmuEX0027731	chr17:12243500,12242650-12242805,12240070	SE	vast-tools
Map7d1	MmuINT0101554	chr4:126237156-126237278=126236896-126237059:-	RI	vast-tools
Mbp	SE	18:82572875-82579903:82579980-82590950:+	SE	SUPPA2
Mbp	SE	18:82593758-82597061:82597183-82602237:+	SE	SUPPA2
Mbtd1	SE	Mbtd1,chr11,+,93782164,93782214,93777847,93777899,93795984,9379	SE	rMATS
Me3	MmuINT0097135	chr7:89833697-89833806=89845795-89845892:+	RI	vast-tools
Med19	A3	2:84516043-84516535:84516043-84516553:+	A3	SUPPA2
Med24	MmuEX0028537	chr11:98717910,98717709-98717765,98716525	SE	vast-tools
Mga	MmuINT0098362	chr2:119921366-119921497=119921738-119921842:+	RI	vast-tools
Mib2	MmuINT0098588	chr4:155656913-155657069=155656646-155656806:-	RI	vast-tools
Mis18bp1	RI	Mis18bp1,chr12,-,65199461,65200667,65199461,65199604,65200579,65	RI	rMATS
Mrpl10	MmuALTD1000586-4/6	chr11:-97041652+97041798+97042005+97042009+97042017+97042143,	A5	vast-tools
Mrpl24	A5	3:87827012-87829124:87826958-87829124:+	A5	SUPPA2
Mrpl24	A5	3:87827135-87829124:87826958-87829124:+	A5	SUPPA2
Mrps18a	A3	17:46433757-46435902:46433757-46436216:+	A3	SUPPA2
Mta1	MmuEX0029871	chr12:113133337+113133576,113134801-113134812,113135797	SE	vast-tools
Mthfsd	RI	Mthfsd,chr8,-,121825446,121825891,121825446,121825510,121825737,	RI	rMATS
Mycbpap	RI	Mycbpap,chr11,-,94403053,94403603,94403053,94403169,94403416,944	RI	rMATS
Myo1b	SE	Myo1b,chr1,-,51807808,51807895,51805980,51806067,51808162,51808	SE	rMATS
Myo6	MX	9:80199799-80200809:80200835-80203925:80199799-80201227:802012	Exc	SUPPA2
Myt1l	MmuALTD0009045-2/2	chr12:29535222-29535229+29535368,29566702	A5	vast-tools
Nars	A3	18:64648479-64649487:64648476-64649487:-	A3	SUPPA2
Ndrg2	SE	14:52148242-52148543:52148584-52148923:-	SE	SUPPA2
Ndufa4	A3	6:11906107-11907323:11906079-11907323:-	A3	SUPPA2
Ndufb8	A5	19:44543496-44543666:44543496-44543756:-	A5	SUPPA2
Ndufs5	A5	4:123609874-123611901:123609874-123611906:-	A5	SUPPA2
Nexn	MmuEX0031533	chr3:152252808,152249660-152249701,152248368	SE	vast-tools
Nfib	A5	4:82238793-82241782:82238793-82241809:-	A5	SUPPA2
Nfib	SE	4:82215048-82228540:82228628-82238711:-	SE	SUPPA2

Nsmaf	MmuINT0111012	chr4:6424879-6424987=6423322-6423498:-	RI	vast-tools
Nup98	MmuINT1024706	chr7:102145557-102145721=102139468-102139650:-	RI	vast-tools
Os9	SE	10:126932611-126932784:126932948-126933801:-	SE	SUPPA2
Osbpl8	MmuEX0033248	chr10:111204840,111224672-111224708,111233553+11123356	SE	vast-tools
Papola	MmuEX0033600	chr12:105829277,105833100-105833162,10583471(	SE	vast-tools
Pard3	MmuEX0033643	chr8:127409617+127409704+127409707,127410729-127410773,127415	SE	vast-tools
Pcbp3	MmuALTD0009991-1/3	chr10:76785140+76785071+76785057-76785214,7678187	A5	vast-tools
Pced1a	MmuINT0060508	chr2:130423412-130424278=130422813-130422892:-	RI	vast-tools
Pcolce	RI	Pcolce,chr5,-,137603984,137605369,137603984,137604062,137605088,1	RI	rMATS
Pde4a	MmuEX1019642	chr9:21194858,21197093-21197178,2119859	SE	vast-tools
Pdgfb	A5	15:79887615-79889670:79887615-79889674:-	A5	SUPPA2
Pdgfb	AF	15:79889766-79897291:79897404:79889766-79898137:79899178:	Exc	SUPPA2
Pdss1	SE	2:22825651-22829091:22829218-22829835:+	SE	SUPPA2
Pkmyt1	RI	Pkmyt1,chr17,+,23951310,23953290,23951310,23951798,23952796,239	RI	rMATS
Plec	MmuEX0035673	chr15:76194751,76194559-76194573,7619443(	SE	vast-tools
Pnpo	A3SS	Pnpo,chr11,-,96830841,96831781,96830841,96830895,96833235,968333	A3	rMATS
Pogz	MmuINT0123390	chr3:94862422-94862530=94864088-94864378:+	RI	vast-tools
Polb	MmuINT0123449	chr8:22639934-22640004=22637068-22637154:-	RI	vast-tools
Ppip5k1	MmuEX0036605	chr2:121327645,121326852-121326914,12132179	SE	vast-tools
Ppp1r16b	SE	2:158593118-158597064:158597189-158597733:+	SE	SUPPA2
Ppp3ca	SE	3:136376662-136486223:136486321-136503487:+	SE	SUPPA2
Ppp3ca	SE	3:136634380-136637772:136637801-136640790:+	SE	SUPPA2
Ppp6r3	MmuINT0125596	chr19:3484831-3484916=3473769-3473866:-	RI	vast-tools
Pradc1	RI	Pradc1,chr6,-,85424917,85425573,85424917,85425027,85425472,85425	RI	rMATS
Prima1	MmuEX0037068	chr12:103202649,103199744+103199783-103199914,10319735	SE	vast-tools
Prpf31	MmuINT1027480	chr7:3630845-3630905=3632668-3632751:+	RI	vast-tools
Prpf39	MmuINT0127086	chr12:65055156-65055320=65055638-65055764:+	RI	vast-tools
Prr14	SE	Prr14,chr7,+,127070876,127071149,127070200,127070291,127071269,1	SE	rMATS
Psd	AF	19:46304515-46305922:46306030:46304515-46306093:46306530:	Exc	SUPPA2
Psmd2	A3	16:20478530-20478707:20478530-20478727:+	A3	SUPPA2
Ptpn2	MmuEX0037818	chr18:67711837,67695624-67695724,6768898	SE	vast-tools
Ptprd	A3	4:76003798-76004249:76003795-76004249:-	A3	SUPPA2
Ptprm	MmuEX0037947	chr17:66919024,66912353-66912427,6689105	SE	vast-tools
Ptrh2	SE	11:86574949-86578862:86578955-86580385:+	SE	SUPPA2
R3hdm1	MmuEX0038208	chr1:128186909,128190638-128190739,12819346(	SE	vast-tools
Rab3il1	SE	Rab3il1,chr19,+,10006837,10006984,10005648,10005867,10007513,1000	SE	rMATS
Rab6b	MmuALTA1024500-1/2	chr9:103162653,103163814+103163810-10316389	A3	vast-tools
Rab7	AF	6:87990680-88021919:88021999:87990680-88022055:88022252:	Exc	SUPPA2
Ralgapb	MmuALTA0014805-2/3	chr2:158448449,158450217+158450189+158450177-15845031(	A3	vast-tools
Ranbp3	RI	Ranbp3,chr17,+,57008022,57009890,57008022,57008088,57009762,5700	RI	rMATS
Raph1	SE	Raph1,chr1,-,60558138,60558219,60549575,60549650,60564750,605652	SE	rMATS
Rbm25	MmuINT0132684	chr12:83674339-83674663=83675044-83675318:+	RI	vast-tools
Rbm28	MmuINT0132749	chr6:29157068-29157139=29155027-29155189:-	RI	vast-tools
Rbms1	MmuEX0039197	chr2:60762053,60759759-60759806+60759922,6075885	SE	vast-tools
Rc3h2	SE	2:37304824-37309444:37309479-37312779:-	SE	SUPPA2
Rcbtb2	MmuEX0039270	chr14:73143188,73151533-73151615,7315431	SE	vast-tools
Reps1	A3SS	Reps1,chr10,+,17983415,17983496,17983418,17983496,17979901,1798	A3	rMATS
Reps2	MmuEX0039430	chrX:162514279,162512679-162512732,16247185	SE	vast-tools
Rims2	MmuEX0039871	chr15:39198538,39275414-39275500,3929210	SE	vast-tools
Rims2	MmuEX0039874	chr15:39437922,39452040-39452107,3945436(	SE	vast-tools
Rnf112	MmuEX0039987	chr11:61453619,61453428-61453496,61452913+61452947+6145297	SE	vast-tools
Robo3	RI	Robo3,chr9,-,37327992,37329097,37327992,37328798,37328986,37329	RI	rMATS
Rpgr	SE	Rpgr,chrX,-,10028840,10028929,10024610,10025088,10030960,1003101	SE	rMATS
Rph3a	AF	5:121147162-121147301:121147601:121147162-121147897:121148143:	Exc	SUPPA2
Rpl3	A3	15:79965412-79965816:79965232-79965816:-	A3	SUPPA2
Rpl35a	A5	16:32877548-32877760:32877535-32877760:+	A5	SUPPA2
Rpl41	A3	10:128384842-128384957:128384840-128384957:-	A3	SUPPA2
Rpn2	SE	2:157163792-157165143:157165190-157167631:+	SE	SUPPA2
Rps12	SE	10:23662778-23662892:23662942-23663054:-	SE	SUPPA2
Rps17	A5	7:80994164-80994605:80994164-80994687:-	A5	SUPPA2
Rps6kb2	MmuINT0137176	chr19:4158829-4158919=4158610-4158717:-	RI	vast-tools
Rpusd1	MmuALTA0015643-4/5	chr17:25727847,25728257+25728252+25728203+25728181+25728164-2	A3	vast-tools
Rreb1	AF	13:38009951:38010028-38072847:38072410:38072656-38072847:-	Exc	SUPPA2
Ryr3	MmuINT0138465	chr2:112640613-112640673=112640140-112640286:-	RI	vast-tools
Scg2	RI	1:79412386:79413648-79413769:79414735:-	RI	SUPPA2
Selenoi	MmuALTD0004973-2/2	chr5:30257712-30257761+30257820,30263071	A5	vast-tools
Senp7	MmuINT0141398	chr16:56179498-56179622=56180526-56180578:+	RI	vast-tools
Sergef	MmuEX0041748	chr7:46621561,46618696-46618758,4661477	SE	vast-tools
Setd5	SE	Setd5,chr6,+,113086384,113086577,113081895,113082082,113086845,1	SE	rMATS
Sgip1	MmuEX0042040	chr4:102860794,102861856-102861939,102863662+10286366	SE	vast-tools
Sgip1	MmuINT0142718	chr4:102918781-102918855=102927461-102927585:+	RI	vast-tools
Sgip1	MmuEX0042041	chr4:102860794,102863662+102863665-102863733,10286764(	SE	vast-tools
Sgip1	SE	Sgip1,chr4,+,102720858,102720930,102717966,102717991,102724836,1(	SE	rMATS

Sgip1	SE	Sgip1,chr4,+,102720861,102720930,102717966,102717991,102724836,1(	SE	rMATS
Slc1a5	RI	7:16529683:16529877-16529949:16530083:+	RI	SUPPA2
Slc25a40	MmuINT1031416	chr5:8442447-8442571=8443612-8443785:+	RI	vast-tools
Smim45	AF	15:82136598:82136949-82143766:82140224:82140586-82143766:-	Exc	SUPPA2
Smim8	SE	Smim8,chr4,-,34771877,34772053,34770887,34771414,34778223,347782	SE	rMATS
Snap25	MX	2:136605541-136611663:136611780-136615815:136605541-136611977:	Exc	SUPPA2
Sorbs2	MmuEX0044378	chr8:45769895,45770546-45770590,4577253	SE	vast-tools
Sorbs2	MmuEX0044385	chr8:45736202,45741487+45741491-45741579,4574542(	SE	vast-tools
Sox5	SE	Sox5,chr6,-,143853112,143853259,143819698,143819876,143887076,14	SE	rMATS
Sox6	A3SS	Sox6,chr7,-,115196730,115196854,115196730,115196851,115258750,11	A3	rMATS
Spats1	SE	Spats1,chr17,-,45763603,45763666,45760360,45760476,45765016,4576!	SE	rMATS
Srrt	RI	Srrt,chr5,-,137299579,137300576,137299579,137299909,137300387,137	RI	rMATS
Srsf11	MmuEX0044989	chr3:158026725,158023243-158023359,15802295(	SE	vast-tools
Ssh3	RI	Ssh3,chr19,-,4316402,4316665,4316402,4316474,4316549,431666!	RI	rMATS
Stau2	MmuEX0045351	chr1:16509342,16486020-16486150+16486165,1646319	SE	vast-tools
Stk35	MmuEX0045458	chr2:129802004,129810488-129811237,12982777	SE	vast-tools
Sumo2	AF	11:115425509-115426605:115426643:115425509-115426924:11542701!	Exc	SUPPA2
Sumo2	AF	11:115425509-115426605:115426643:115425509-115426924:11542710	Exc	SUPPA2
Supt5	MmuEX0045761	chr7:28330580,28330018-28330029,2832965	SE	vast-tools
Svip	AF	7:51653733-51655291:51655446:51653733-51655486:51655766:	Exc	SUPPA2
Sympk	MmuEX0045915	chr7:19024506,19027897-19028022,1902886	SE	vast-tools
Synj1	MmuEX0045984	chr16:90950351,90948066-90948113,9094686	SE	vast-tools
Taf3	MmuEX0046207	chr2:9940927,9921052-9921301,991820(	SE	vast-tools
Taf7l	A5	X:133370920-133371129:133370920-133371237:-	A5	SUPPA2
Taf7l	AF	X:133373716-133375673:133375742:133373716-133376940:133377239:	Exc	SUPPA2
Tceal5	AF	X:135103201-135104263:135104386:135103201-135104387:135104481:	Exc	SUPPA2
Tceal5	AF	X:135103201-135104263:135104386:135103201-135104516:135104625:	Exc	SUPPA2
Tceal5	AF	X:135103201-135104387:135104481:135103201-135104516:135104625:	Exc	SUPPA2
Tcf12	SE	Tcf12,chr9,-,71913982,71914044,71907697,71907800,72016957,7201703	SE	rMATS
Tenm4	AF	7:95820453:95820700-96004563:95858870:95858991-96004563:-	Exc	SUPPA2
Tenm4	AF	7:95820453:95820700-96004563:95859361:95859616-96004563:-	Exc	SUPPA2
Tet2	MmuEX0046991	chr3:133483239,133480883+133481334-133481427,13348034	SE	vast-tools
Tle5	A3	10:81401200-81401306:81401200-81401309:+	A3	SUPPA2
Tma7	AL	9:108903636:108903969-108911395:108911131:108911294-108911395:	Exc	SUPPA2
Tmem18	MmuINT0161713	chr12:30585470-30585590=30587200-30587254:+	RI	vast-tools
Tmem191c	MmuINT0161819	chr16:17277669-17277741=17277851-17277891:+	RI	vast-tools
Tmem209	SE	Tmem209,chr6,-,30506784,30506916,30505731,30505973,30508452,305	SE	rMATS
Tmem63a	MmuINT0162287	chr1:180952132-180952195=180952981-180953018:+	RI	vast-tools
Tmsb4x	A5	X:165991047-165992004:165991047-165992102:-	A5	SUPPA2
Tnrc6c	MmuINT0163576	chr11:117739867-117740009=117741645-117741818:+	RI	vast-tools
Tom1l2	SE	11:60121963-60123724:60123783-60132644:-	SE	SUPPA2
Tom1l2	SE	11:60153609-60161144:60161293-60165750:-	SE	SUPPA2
Trank1	MmuEX1026611	chr9:111373545,111374735+111374905-111374925,11137778	SE	vast-tools
Trank1	MmuEX0048760	chr9:111383162,111385021+111385119-111385204+111385430,111386	SE	vast-tools
Trank1	SE	Trank1,chr9,+,111214186,111214272,111212141,111212230,111215438,	SE	rMATS
Trim9	MmuALTD0014812-1/2	chr12:70251011+70250957-70251260,7024842	A5	vast-tools
Trip12	AF	1:84792690-84816332:84816452:84792690-84816950:84817025:	Exc	SUPPA2
Trmt11	MmuEX0049031	chr10:30590025,30587439-30587595,3056650(	SE	vast-tools
Tssc4	MmuINT1034011	chr7:143069249-143069513=143069755-143069850:+	RI	vast-tools
Ttc3	MmuEX0049521	chr16:94384066,94384346-94384388,9438478	SE	vast-tools
Ttll5	MmuALTA0019117-2/2	chr12:85933674,85939329+85939326-8593951(	A3	vast-tools
Ubb	AF	11:62441997:62442421-62442966:62442502:62442677-62442966:-	Exc	SUPPA2
Ubxn7	SE	16:32178893-32186162:32186227-32188133:+	SE	SUPPA2
Usp28	MmuEX0050910	chr9:49032353,49032509-49032616+49032688,4903559	SE	vast-tools
Usp28	MmuEX0050911	chr9:49032353,49033577-49033672,4903559	SE	vast-tools
Usp34	MmuEX0050974	chr11:23361716,23362577-23362612,2336305	SE	vast-tools
Uspl1	MmuEX0051128	chr5:149184806,149187708-149187834+149187863+149187876,149188	SE	vast-tools
Vav2	SE	Vav2,chr2,-,27158162,27158249,27157241,27157396,27158910,2715903	SE	rMATS
Vcl	MmuINT0172783	chr14:21020479-21020781=21022003-21022127:+	RI	vast-tools
Vmn1r43	A5	6:89848408-89853084:89848408-89853243:-	A5	SUPPA2
Vmn1r43	AF	6:89848408-89849588:89849636:89848408-89853084:89853395:	Exc	SUPPA2
Vmn1r43	AF	6:89848408-89849588:89849636:89848408-89853243:89853395:	Exc	SUPPA2
Vmn1r43	RI	6:89835916:89845523-89848023:89848408:-	RI	SUPPA2
Vmn1r77	A3	7:11774430-11775224:11774430-11775277:+	A3	SUPPA2
Vps13c	SE	Vps13c,chr9,+,67773461,67773524,67768065,67768167,67778457,67778	SE	rMATS
Vps13d	A3SS	Vps13d,chr4,-,144826534,144826640,144826534,144826637,144832368,	A3	rMATS
Wdr83	MmuINT0175801	chr8:85080120-85080225=85079789-85079837:-	RI	vast-tools
Wnk1	SE	6:119920827-119921662:119921761-119923198:-	SE	SUPPA2
Wtap	AF	17:13204840-13210964:13211146:13204840-13211196:13211384:	Exc	SUPPA2
Xpc	RI	Xpc,chr6,-,91473069,91475172,91473069,91473151,91475011,9147517	RI	rMATS
Yipf2	A5	9:21503884-21504023:21503884-21504033:-	A5	SUPPA2
Yipf2	RI	9:21499978:21500854-21500931:21501056:-	RI	SUPPA2
Ywhae	AF	11:75623695:75623917-75642707:75623974:75624071-75642707:-	Exc	SUPPA2

Zbtb49	SE	Zbtb49,chr5,-,38369189,38369432,38367961,38368008,38370651,38370	SE	rMATS
Zcwpw1	SE	Zcwpw1,chr5,+,137815154,137815238,137810263,137810362,13781571	SE	rMATS
Zfc3h1	MmuINT0178470	chr10:115431403-115431442=115431703-115431765:+	RI	vast-tools
Zfp131	RI	13:120237303:120237525-120237628:120237985:-	RI	SUPPA2
Zfp369	MmuINT0179107	chr13:65294396-65294484=65295600-65295726:+	RI	vast-tools
Zfp383	MmuINT1035579	chr7:29912590-29912685=29914617-29916807:+	RI	vast-tools
Zfp672	SE	Zfp672,chr11,-,58210692,58210780,58210149,58210416,58213394,5821	SE	rMATS
Zfp7	RI	Zfp7,chr15,+,76772430,76776595,76772430,76772547,76774206,767765	RI	rMATS
Zfp950	SE	Zfp950,chr19,-,61108756,61108890,61106664,61106785,61115444,6111	SE	rMATS
Zfp956	MmuEX0053739	chr6:47956170,47957476-47957602,47962525+47962531	SE	vast-tools
Zmynd11	MmuALTD0016338-2/2	chr13:9690446+9690278-9690510,9689724	A5	vast-tools
